# Effects of weaning on intestinal crypt epithelial cells in piglets

**DOI:** 10.1038/srep36939

**Published:** 2016-11-10

**Authors:** Huansheng Yang, Xia Xiong, Xiaocheng Wang, Tiejun Li, Yulong Yin

**Affiliations:** 1 Animal Nutrition and Human Health Laboratory, School of Life Sciences, Hunan Normal University, Changsha, China; 2 Chinese Academy of Science, Institute of Subtropical Agriculture, Research Center for Healthy Breeding of Livestock and Poultry, Hunan Engineering and Research Center of Animal and Poultry Science and Key Laboratory for Agroecological Processes in Subtropical Region, Scientific Observation and Experimental Station of Animal Nutrition and Feed Science in South-Central, Ministry of Agriculture, Changsha, China

## Abstract

Intestinal epithelial cells in the crypt proliferate in piglets in response to weaning. However, the underlying mechanism has been unclear. We examined 40 piglets from eight litters (five piglets per litter) that were weaned at the age of 14 d, and one piglet from each litter was randomly selected for closer investigation. Based on the distended intestinal sac method, we isolated crypt epithelial cells from the mid-jejunum on Days 0, 1, 3, 5, and 7 post-weaning. Protein expression was analyzed using either isobaric tags for relative and absolute quantification or western blotting. Proteins related to the cell cycle, organization of the cellular macromolecular complex subunit, localization of cellular macromolecules, Golgi vesicle transport, fatty acid metabolism, oxidative phosphorylation, and translational initiation were mainly down-regulated, while those involved in glycolysis, cell cycle arrest, protein catabolism, and cellular amino acid metabolism were up-regulated. The amount of proteins active in the mTOR signaling pathway was generally decreased over time. These results indicate that weaning influences energy metabolism, cellular macromolecule organization and localization, and protein metabolism, thereby affecting the proliferation of intestinal epithelial cells in weaned piglets. Moreover, those cellular processes are possibly controlled by that signaling pathway.

The small intestine of animals fed with breast milk grows more rapidly during the suckling period when compared with littermates that receive artificial formula. This is because milk contains growth factors that can regulate the proliferation of intestinal epithelial cells postnatally^1^. The expression of various receptors, e.g., c-met [hepatocyte growth factor (HGF) receptor], epidermal growth factor (EGF) receptor, erythropoietin (Epo) receptor, insulin-like growth factor-1 (IGF-1) receptor, glucagon-like polypeptide (GLP)-2 receptor, and feratinocye growth factor (KGF) is detected in the intestine of neonatal animals^2^, and breast milk also has growth factors such as HGF, EGF, Epo, IGF-1, IGF-II, and transforming growth factor-β (TGF-β)^2^,^3^. Moreover, the proliferation of intestinal epithelial cells *in vivo* or *in vitro* is altered by treatment with growth factors^2^,^4^,^5^. Weaning in piglets is an abrupt process that replaces milk feeding with formulated feed that lacks growth factors, which then changes epithelial growth, cell proliferation, and intestinal morphology^6^. For example, in various animal species, the small intestinal villus becomes shorter while the crypt depth increases post-weaning^6^,^7^.

The diet of weaning piglets shifts from high-fat, low-carbohydrate milk to a high-carbohydrate and low-fat feed. When combined with changes in their social and physical environments, the intake of nutrients by these piglets declines significantly in the first few days post-weaning. This lack of sufficient enteral nutrients may lead to reduced proliferation of epithelial cells and enhanced growth of intestinal mucosa, as seen with total parenteral nutrition-fed animals that usually exhibit gut atrophy and a net loss of mucosal protein^8^,^9^. Dudley *et al*. have shown that the synthesis of jejunal mucosal protein is lower in parenterally fed piglets than in those that are enterally fed^10^. Moreover, the synthesis and degradation of proteins in the intestine can be altered when luminal substrate is missing, and enterally administered nutrients can stimulate the secretion of growth factors that have intestinal trophic effects^8^,^11^. In experiments by Burrin *et al*., neonatal piglets were given 0%, 10%, 20%, 40%, 60%, 80%, or 100% of their total nutrient intake enterally, with any remainder provided parenterally. Overall, the intestinal wet weight, protein content, DNA content, villus height, crypt depth, and epithelial cell proliferation were increased as the proportion of enteral nutrients rose^8^. Stoll *et al*. have shown that total parenteral nutrition-fed neonatal pigs experience a loss of intestinal proteins, but that a protein balance occurs at 20% enteral nutrient intake, and protein accretion is stimulated at 60% to 100% enteral nutrient intake^12^. Therefore, all of these results indicate that enteral nutrients play an important role in regulating intestinal protein accretion, epithelial cell proliferation, and mucosa growth.

A highly coordinated process of renewal is followed for intestinal epithelial cells^13^. Most are shed into the intestinal lumen every 3 to 5 d, and the rapid proliferation of cells near the base of the crypt has a key role in supplementing those lost cells and supporting intestinal growth, maintenance, and recovery from tissue damage^13^,^14^. Although epithelial cell proliferation in piglets is affected by weaning^6^,^7^, most studies have focused on measuring rates but have not examined the underlying mechanism^15^,^16^. Therefore, our research objective was to investigate how weaning influences the proliferation of those intestinal epithelial cells.

## Results

### Changes in protein expression in intestinal crypt epithelial cells after weaning

**Figure 1:**
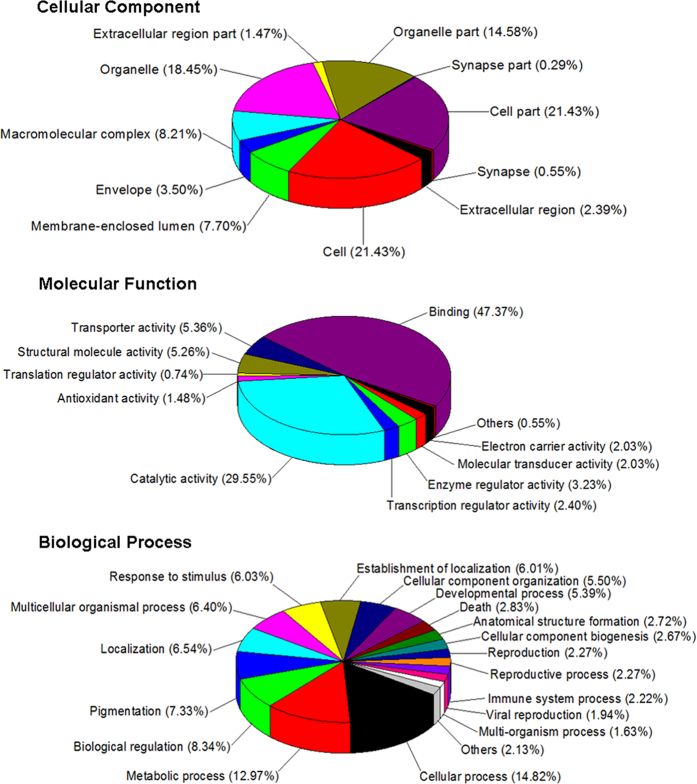
Gene Ontology terms of enrichment (based on WEGO program) for categories of cellular component, molecular function, and biological process for proteins in jejunal crypt epithelial cells of piglets during post-weaning period. Any protein with ≥1.2-fold or ≤0.8-fold difference between w1d, w3d, w5d, or w7d level and w0d level (*P* ≤ 0.05) was considered differentially expressed.

A total of 615 differentially expressed proteins were identified in the crypt epithelial cells from w0d, w1d, w3d, w5d, and w7d piglets (i.e., Days 0, 1, 3, 5, and 7 after abrupt weaning; Supplementary data). Cellular Component Gene Ontology (GO) enrichment analysis showed that these proteins were mainly involved in cell, cell part, organelle, organelle part, macromolecular complex, membrane-enclosed lumen, envelope, extracellular region, and extracellular region part (Fig. 1). Within the category of Molecular Function, these proteins were primarily active in binding, as well as activities associated with catalysis, transporters, structural molecules, enzyme and transcription regulators, molecular transducers, electron carriers, and antioxidants. The Biological Process terms included those for cellular and metabolic processes, biological regulation, pigmentation, localization, multicellular organismal process, response to stimulus, establishment of localization, cellular component organization, and developmental processes. The Kyoto Encyclopedia of Genes and Genomes (KEGG) analysis of pathway enrichment (Fig. S1) showed that these proteins were mainly involved in oxidative phosphorylation, carbon metabolism, ribosome, glycolysis/gluconeogenesis, biosynthesis of amino acids, spliceosome, protein processing in the endoplasmic reticulum, arginine and proline metabolism, the citrate cycle, and phagosomes.

**Figure 2:**
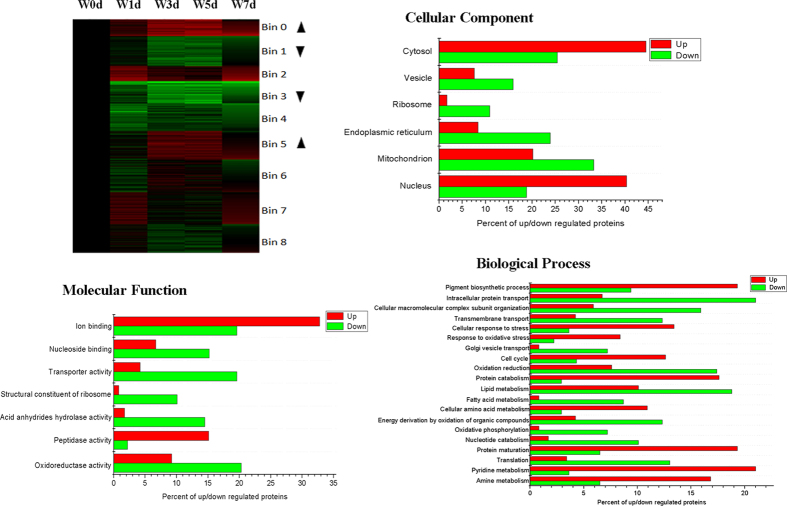
Functional categorization of proteins in jejunal crypt epithelial cells of piglets during post-weaning period. Differentially expressed proteins were grouped using Cluster 3.0 with k-means clustering, and up-regulated (Up arrowhead) and down-regulated (Down arrowhead) protein groups were selected. Gene Ontology for cellular component, molecular function, and biological process categories was determined by WEGO analysis. Terms having *P*-values ≤ 0.05 (Pearson Chi-Square test between numbers of Up and Down proteins) were selected.

These differentially expressed proteins were clustered into nine distinct groups (Bins 0 to 8) through k-means clustering. Whereas the expression of proteins in Bin0 and Bin5 was up-regulated after weaning, that of proteins in Bin1 and Bin3 was down-regulated. We also used the Web Gene Ontology (WEGO) program to analyze GO enrichment of Up proteins and Down proteins, and selected GO terms that were significantly (*P* < 0.05) different between those two kinds of protein (Fig. 2). For the ontology type of Cellular Component, the percent of proteins related to the cytosol and nucleus was significantly greater in the Up group while the percent of proteins related to the vesicle, ribosome, endoplasmic reticulum, and mitochondrion was significantly higher in the Down group. For the ontology type of Molecular Function, the Up group had a significantly higher percent of proteins related to ion binding and peptidase activity but a significantly lower percent of proteins involved in nucleoside binding, transporter activity, structural constituents of the ribosome, acid anhydride hydrolase activity, and oxidoreductase activity. For the ontology type of Biological Process, the percent of proteins related to pigment biosynthesis, cellular response to stress, response to oxidative stress, cell cycle, protein catabolism, cellular amino acid metabolism, protein maturation, pyridine metabolism, and amine metabolism was significantly higher in the Up group while the percent of proteins involved in intracellular protein transport, cellular macromolecular complex submit organization, transmembrane transport, Golgi vesicle transport, oxidation reduction, lipid metabolism, fatty acid metabolism, energy derivation by oxidation of organic compounds, oxidative phosphorylation, nucleotide catabolism, and translation was significantly higher in the Down group.

### Golgi vesicle transport, cellular macromolecular complex subunit organization, and cellular macromolecule localization

**Figure 3:**
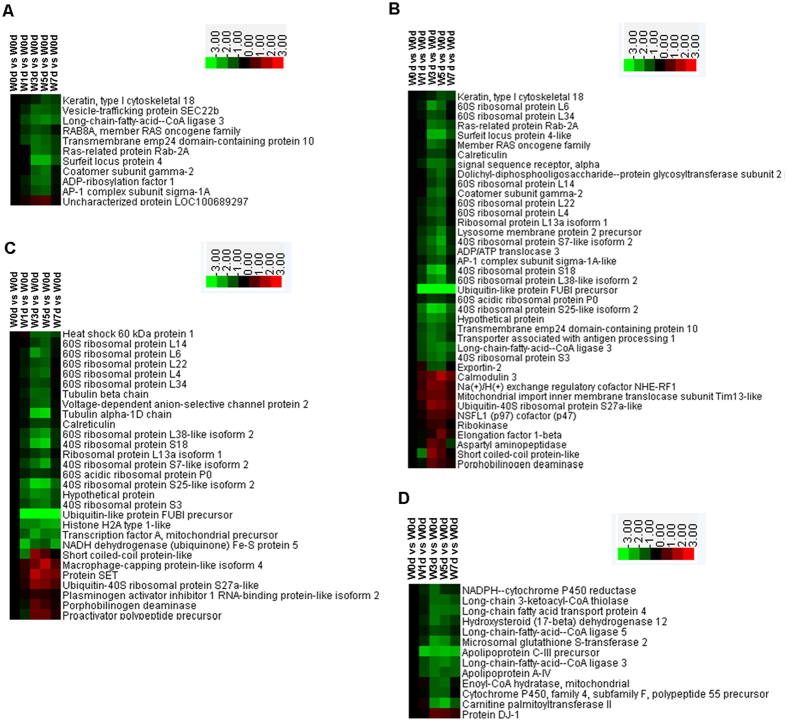
Proteins involved in Golgi vesicle transport (**A**), cellular macromolecule localization (**B**) and organization (**C**), and fatty acid metabolism (**D**) in jejunal crypt epithelial cells of weaning piglets. Proteins enriched in these processes were selected and grouped via Cluster 3.0. Relationships were significant (*P* < 0.05) between Up-regulated and Down-regulated proteins, based on WEGO analysis.

Among the 11 proteins involved in Golgi vesicle transport that were in either the Up or Down groups, the expression of 10 was down-regulated after weaning. These were mainly involved in retrograde vesicle-mediated transport (Golgi to ER), post-Golgi vesicle-mediated transport, and ER to Golgi vesicle-mediated transport (Fig. 3A). In all, 39 proteins were identified as having roles in cellular macromolecule localization, with 29 being down-regulated after weaning. They included proteins primarily related to signal recognition particle (SRP)-dependent cotranslational targeting to membranes and intracellular protein transport (Fig. 3B). Among the 29 proteins related to organization of the cellular macromolecular complex subunit, 22 were down-regulated after weaning and were mainly involved in translational termination and cellular protein complex assembly (Fig. 3C).

### Fatty acid metabolism, glycolysis, and citrate cycle

**Figure 4:**
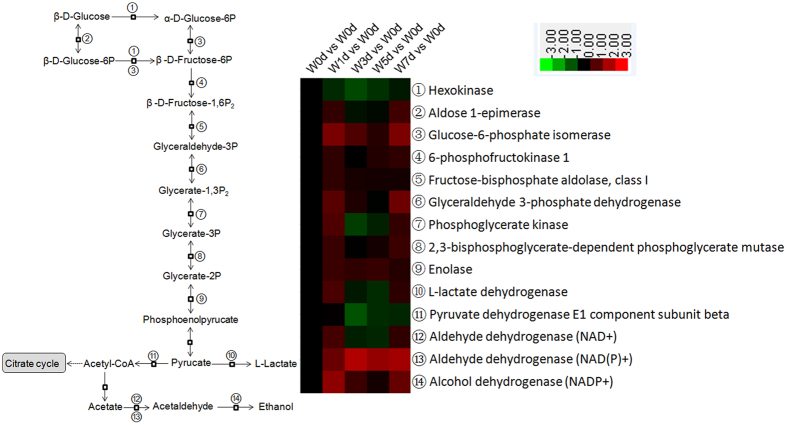
Enrichment of proteins in glycolysis pathway in jejunal crypt epithelial cells of weaning piglets, based on analysis using KEGG database. Any protein with ≥1.2-fold or ≤0.8-fold difference between w1d, w3d, w5d, or w7d level and w0d level (*P* ≤ 0.05) was considered differentially expressed.

**Figure 5:**
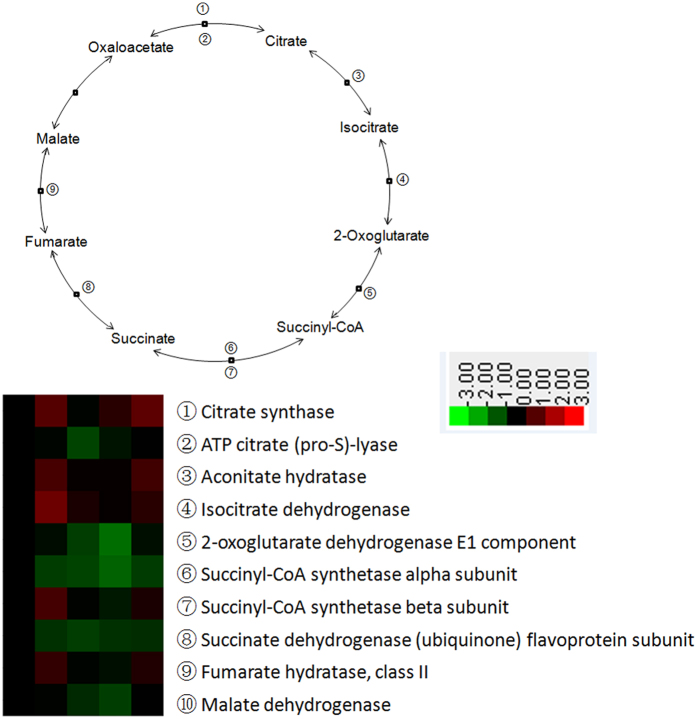
Enrichment of proteins in citrate cycle pathway in jejunal crypt epithelial cells of weaning piglets, based on analysis using KEGG database. Any protein with ≥1.2-fold or ≤0.8-fold difference between w1d, w3d, w5d, or w7d level and w0d level (*P* ≤ 0.05) was considered differentially expressed.

Of the 13 proteins shown to have roles in fatty acid metabolism, 12 were down-regulated in expression after weaning, and were mainly involved in processes of fatty acid beta-oxidation and biosynthesis (Fig. 3D). According to the KEGG database, proteins related to glycolysis were enriched and their expression was generally elevated after weaning (Fig. 4). Proteins involved in the citrate cycle were also enriched, with some being down-regulated while others were up-regulated (Fig. 5).

### Energy derived from oxidation of organic compounds and oxidative phosphorylation

**Figure 6:**
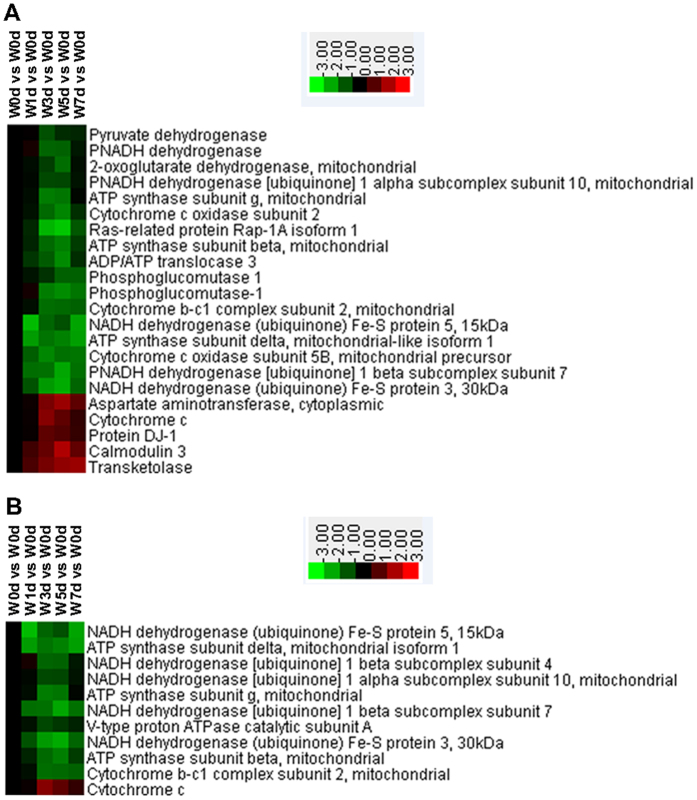
Proteins involved in energy derivation by oxidation of organic compounds (**A**) and oxidative phosphorylation (**B**) in jejunal crypt epithelial cells of weaning piglets. Proteins enriched in these processes were selected and grouped via Cluster 3.0. Relationships were significant (*P* < 0.05) between Up-regulated and Down-regulated proteins, based on WEGO analysis.

We identified 22 proteins related to the derivation of energy via oxidation. Of those, 17 were down-regulated after weaning. They were mainly involved in the respiratory electron transport chain, mitochondrial electron transport (NADH to ubiquinone), and processes of energy reserve metabolism (Fig. 6A). Among the 11 proteins that were involved in oxidative phosphorylation, the expression of 10 was reduced after weaning. These were primarily active in mitochondrial electron transport (NADH to ubiquinone) and ATP synthesis-coupled proton transport (Fig. 6B).

### Translational initiation, cell cycle, mTOR signaling pathway, and cellular responses to DNA damage stimuli

**Figure 7:**
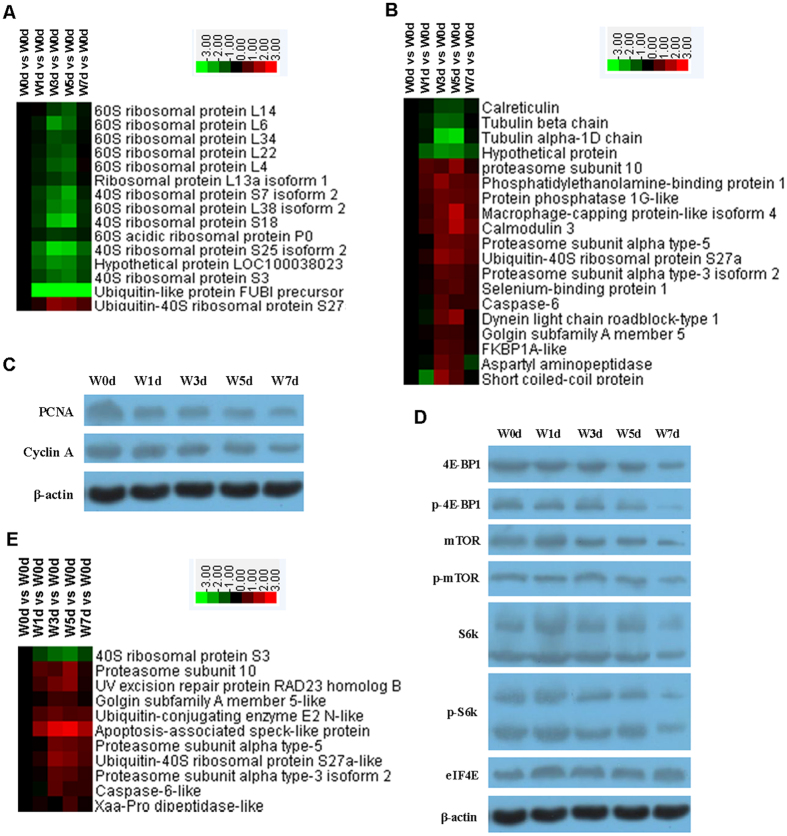
Proteins involved in translational initiation (**A**), mitotic cell cycle (**B**), and mTOR signaling pathway (**E**) in jejunal crypt epithelial cells of weaning piglets. Proteins enriched in these processes were selected and grouped via Cluster 3.0. Relationships were significant (*P* < 0.05) between Up-regulated and Down-regulated proteins, based on WEGO analysis.The expression of proteins in cell cycle (**C**) and mTOR signaling pathway (**D**) was measured using Western blotting. The β-actin band presented in C and D is the same, which is because we used the same samples for measuring these proteins.

Among the 15 proteins that showed roles in translational initiation, the expression of 14 was down-regulated after weaning (Fig. 7A). In all 15 of the 19 proteins related to cell cycle processes were up-regulated. These proteins were mainly involved in DNA damage responses, as well as signal transduction by the p53 class mediator that leads to cell cycle arrest (Fig. 7B). The results of our western blotting showed that expression of proliferating cell nuclear antigen (PCNA) and Cyclin A was reduced after weaning (Fig. 7C; Fig. S2). We also utilized western blots to determine the abundance of proteins in the mTOR signaling pathway and found that 4E-BP1, p-4E-BP1, mTOR, p-mTOR, S6k, and p-S6k, but not eIF4E, were decreased after weaning (Fig. 7D; Fig. S3). Nine out of 10 proteins associated with cellular responses to DNA damage stimuli were up-regulated. These were mainly involved in the DNA damage checkpoint and DNA repairs (Fig. 7E).

### Cellular amino acid metabolism and protein catabolism

**Figure 8:**
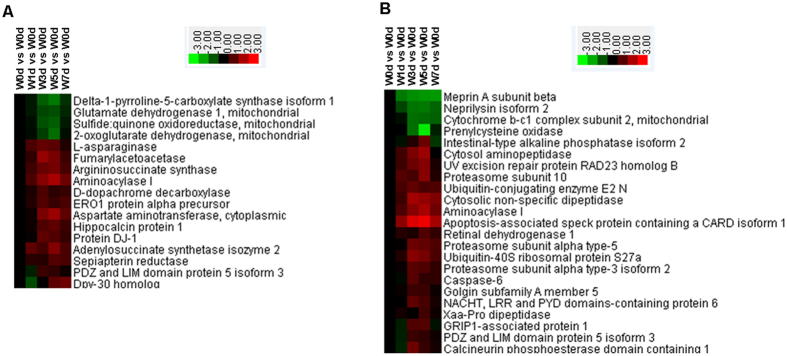
Proteins involved in cellular amino acid metabolism (**A**) and protein catabolism (**B**) in jejunal crypt epithelial cells of weaning piglets. Proteins enriched in these processes were selected and grouped via Cluster 3.0. Relationships were significant (*P* < 0.05) between Up-regulated and Down-regulated proteins, based on WEGO analysis.

Of the 17 proteins related to cellular amino acid metabolism, 13 were up-regulated after weaning and were mainly involved in process for glutamine family amino acid metabolism and aspartate family amino acid catabolism (Fig. 8A). In all, 19 of 23 proteins with a role in protein catabolism were up-regulated. They were mainly involved in proteolysis and the process of proteasomal ubiquitin-dependent protein catabolism (Fig. 8B).

## Discussion

For piglets, weaning is accompanied by changes in intestinal morphology and cell proliferation in the crypt. To investigate the underlying mechanism, which is still not well understood, we isolated jejunal crypt epithelial cells. A total of 628 differentially expressed proteins were identified at 0, 1, 3, 5, and 7 d post-weaning. They are generally involved in cellular and metabolic processes, biological regulation, pigmentation, localization, multicellular organismal processes, response to stimuli, and the establishment of localization, suggesting that many of those biological processes are altered during the post-weaning period. Our analysis of KEGG signaling pathway enrichment also indicated that these differentially expressed proteins are mainly enriched in oxidative phosphorylation, carbon metabolism, ribosome, glycolysis/gluconeogenesis, biosynthesis of amino acids, spliceosome, protein processing in the endoplasmic reticulum, arginine and proline metabolism, and the citrate cycle. These findings will likely contribute to our further research with weaning piglets. We believe that no proteome-wide changes in intestinal epithelial cells, especially the crypt epithelial cells, have previously been reported in piglets during the post-weaning period. Therefore, such insight may lead to the development of new approaches for improving gastrointestinal functions and the health of weaning piglets.

Wang *et al*. have shown that the mRNA expression of genes related to cell proliferation in the jejunum of weaned piglets is decreased in comparison with samples from age-matched suckling piglets^16^. Furthermore, Zhu *et al*. have demonstrated that expression of genes involved in apoptosis and pro-inflammatory signals is enhanced while that of genes related to cell cycle control is lower in weaned piglets than in age-matched suckling piglets. These reports indicate that weaning induces cell cycle arrest and inhibits cell proliferation in the jejunum^7^. Consistent with those from previous studies, our results also provide evidence that the expression of proteins related to the cellular response to DNA damage stimuli and signal transduction by a p53 class mediator causes cell cycle arrest to increase in jejunal crypt epithelial cells during the post-weaning period. In contrast, expression of proteins that function in the cell cycle (PCNA and cyclin A) is down-regulated. These findings indicate that weaning-associated stress induces DNA damage and cell cycle arrest and also reduces the proliferation of those epithelial cells. The mTOR signaling pathway is involved in sensing intracellular and extracellular signals and plays important roles in controlling critical cellular processes such as proliferation, growth, and metabolism^17^,^18^. Here, the abundance of proteins in that pathway decreased over time. Therefore, it appears that weaning affects the proliferation of jejunal epithelial cells via the mTOR pathway. Furthermore, because that pathway regulates cellular processes by coordinating anabolism and catabolism due to nutrient, energy, and growth factor signaling, the decline in mTOR activity in our study cells might have resulted when the animals were deprived of growth factors in breast milk and their intake of nutrients became lower during the post-weaning period^19^.

The reduction in energy intake is a major cause of intestinal dysfunction in weaning piglets^6^,^20^. Different from other organs, the amino acids (glutamate, glutamine, and aspartate) are the major contributors to the generation of oxidative energy in the small intestine^21^,^22^. Glucose and fatty acids also play important roles in providing energy for epithelial cells in that organ^23^,^24^. Our results demonstrated that the expression of proteins related to amino acids is generally up-regulated. They are mainly involved in the metabolism of amino acids within the glutamine and aspartate families. Therefore, it seems that amino acids are critical for providing energy to the jejunal crypt epithelial cells when piglets are weaned. Similarly, the expression of proteins related to glycolysis increases. This response to weaning in the crypt cells differs from that in the jejunal upper and middle villus epithelial cells, where glycolysis-associated proteins is mostly diminished (unpublished data). Such a contrast may result because the proteins are found in different locations along the crypt-villus axis (CVA). Therefore, the villus epithelial cells are more dependent upon luminal nutrients whereas nutrients for the crypt epithelial cells are provided, in part, through the blood^25^. Expression of proteins related to fatty acids is mainly down-regulated in jejunal crypt epithelial cells as well as in the jejunal upper and middle villus epithelial cells (unpublished data). This might be explained because the diets of weaning piglets is converted from high-fat, low-carbohydrate milk to feed that is high-carbohydrate and low-fat^26^.

The citrate cycle has a key role in the oxidation of substrates such as glucose, fatty acids, and amino acids, e.g., glutamine, glutamate, and aspartate^27^,^28^. Our results indicated that expression is inconsistent for related proteins in the jejunal crypt epithelial cells, with some being up-regulated and others down-regulated. The latter response may restrict functioning of the citrate cycle in oxidating substrates to generate energy. The oxidative phosphorylation process is necessary to the transfer of electrons to O_2_ and for synthesizing ATP^29^,^30^. Finally, the expression of proteins that are active in the respiratory electron transport chain and mitochondrial electron transport (NADH to ubiquinone) is mainly down-regulated in the jejunal crypt epithelial cells, demonstrating that processes for oxidative phosphorylation are inhibited in the jejunal crypt of weaning piglets.

Early-weaned piglets commonly experience diarrhea during the first two weeks, perhaps due to dysfunctioning of the gastrointestine that usually leads to intestinal atrophy^6^,^20^. We also found that the expression of proteins related to catabolism was increased in the intestinal epithelial cells, which suggests that protein catabolism leads to intestinal atrophy. Proteins can be catabolized when cells lack specific amino acids that are important for maintaining intestinal functions. They include glutamine, the major metabolic fuel for the intestinal epithelium and a precursor for intense nucleotide biosynthesis^21^,^31^. In fact, Wu *et al*. have shown that dietary supplementation with glutamine prevents jejunal atrophy in weaned piglets. We also noted that amino acid metabolism was increased in jejunal crypt epithelial cells, which implied that they require more amino acids during the post-weaning period^32^. However, the weaning piglets usually have a low feed intake that does not meet the requirements of those intestinal epithelial cells^33^.

In conclusion, our findings are evidence that, during the post-weaning period, expression in the jejunal crypt epithelial cells is mainly decreased for proteins related to the cell cycle, cellular macromolecular complex subunit organization, cellular macromolecule localization, Golgi vesicle transport, fatty acid metabolism, oxidative phosphorylation, and translational initiation. By contrast, expression increases for proteins involved in glycolysis, cell cycle arrest, protein catabolism, and cellular amino acid metabolism. Furthermore, those cellular processes may be regulated by the mTOR signaling pathway. This indicates that weaning may affect the proliferation of intestinal epithelial cells by influencing energy metabolism, cellular macromolecule organization, and localization. These results provide new directions for exploring the mechanism by which weaning has an impact on the proliferation of intestinal epithelial cells and intestinal functioning in young piglets.

## Materials and Methods

### Reagents

The following reagents were used: DL-β-Hydroxybutyrate sodium salt (J&K Chemical Ltd., Beijing, China); trypsin (Promega, Madison, WI, USA); iTRAQ-reagent (Applied Biosystems, Foster City, CA, USA); and bovine serum alumin (BSA fraction V), phenylmethylsulfonyl fluoride, and dithiothreitol (DTT) (all from Sigma-Aldrich, St. Louis, MO, USA).

### Animals and isolation of intestinal crypt epithelial cells

A total of 40 piglets (Duroc × [Landrace × Yorkshire]) from five litters (five piglets per litter) were weaned at the age of 14 d and then fed a creep diet that met the National Research Council nutrient specifications for 5- to 10-kg body weight pigs, as previously described^34^,^35^. Piglets from the same litter were placed together in each pen and all had free access to feed and drinking water at all times throughout the experimental period. At 0 d (w0d), 1 d (w1d), 3 d (w3d), 5 d (w5d), and 7 d (w7d) after weaning, one piglet per litter was maintained under general anesthesia and then sacrificed by an intravenous (jugular vein) injection of 4% sodium pentobarbital solution (40 mg kg^−1^). The intestinal crypt epithelial cells were isolated by the distended intestinal sac method as previously described, but with slight modifications^35^,^36^. Briefly, the divided mid-jejunum segments were rinsed thoroughly with ice-cold physiological saline solution and incubated at 37 °C for 30 min with oxygenated phosphate buffered saline. After incubation with an oxygenated isolation buffer [5 mM Na2EDTA, 10 mM HEPES (pH 7.4), 0.5 mM DTT, 0.25% BSA, 2.5 mM D-glucose, 2.5 mM L-glutamine, and 0.5 mM dl-β-hydroxybutyrate sodium salt], the samples were oxygenated with an O_2_/CO_2_ mixture (19:1, v:v) at 37 °C for 40 min and 50 min, respectively. The segments were then filled with oxygenated isolation buffer and incubated at 37 °C for 60 min to isolate the crypt epithelial cells. The isolation buffers were collected and centrifuged at 400 g for 4 min at 4 °C. Collected cells were washed twice with an oxygenated cell suspension buffer [10 mM HEPES, 1.5 mM CaCl_2_, and 2.0 mM MgCl_2_ (pH 7.4)] and the cells were retained through centrifugation at 400 g for 4 min at 4 °C. They were immediately frozen in liquid nitrogen and stored at −80 °C. This cell fraction procedure was validated by measuring the activity of alkaline phosphatase and the expression of PCNA along CVA, as described previously^35^,^36^. Our experimental design and procedures were in accord with those specified in the Chinese Guidelines for Animal Welfare and Experimental Protocols, and were approved by the Animal Care and Use Committee of the Institute of Subtropical Agriculture at the Chinese Academy of Sciences.

### Sample preparation and isobaric labeling

The harvested cells were re-suspended and disrupted in a lysis buffer composed of 7 M urea, 2 M thiourea, 4% w/v 3-[(3-Cholamidopropyl) dimethylammonio] propanesulfonate, 20 mM tributyl phosphate, and 0.2% Bio-lyte (pH 3–10), and a protease inhibitor cocktail (Roche Diagnostics Ltd, Mannheim, Germany). DNAse I and RNAse A were added to the lysate at final concentrations of 1 mg mL^−1^ and 0.25 mg mL^−1^, respectively. After disruption, the protein solution was separated from the cell debris by centrifugation (12,000 × g, 5 min, 4 °C). The crude protein extracts were further purified using a Ready Prep 2-D Cleanup Kit (Bio-Rad Laboratories, USA) before undergoing a reductive alkylation reaction. The protein concentration was determined using a 2-D Quant Kit (GE Healthcare, USA). Digestion and labeling were performed according to the manufacturer’s protocol (Applied Biosystems). Briefly, 100 μg of total protein in the cell fraction was reduced and alkylated, then digested overnight at 37 °C with trypsin and labeled with iTRAQ-reagents (Applied Biosystems) as follows: w0d, iTRAQ reagent 115; w1d, iTRAQ reagent 116; w3d, iTRAQ reagent 117; w5d, iTRAQ reagent 118; and w7d, iTRAQ reagent 121.

### Peptide fractionation and LC-MS/MS acquisition

The isotopically labeled samples were pooled and then separated into 12 fractions by an Ultremex SCX column containing 5-μm particles (Phenomenex, USA). The eluted fractions were de-salted using a Strata X C18 column (Phenomenex) and dried under vacuum. The final average peptide concentration in each fraction was approximately 0.25 μg μL^−1^. Dried peptides were stored at −80 °C prior to the MS analysis. A nanospray ion source (Waters, USA) system coupled with Triple TOF was used for analytical separations. Microfluidic traps and nanofluidic columns packed with Symmetry C18 (5 μm, 180 μm × 20 mm) were utilized for online trapping and de-salting, and nanofluidic columns packed with BEH130 C18 (1.7 μm, 100 μm × 100 mm) were employed in the separations. Solvents comprised water/acetonitrile/formic acid (A: 98.0/2.0/0.1%; B: 2.0/98.0/0.1%). A portion of a 2.25-μg (9-μL) sample was loaded for trapping and de-salting. At a flow rate of 300 nL min^−1^, this separation was established by maintaining 5% B for 1 min. During the next 64 min, a linear gradient to 35% B occurred over 40 min. After the peptide elution window, the gradient was increased to 80% B in 5 min and then maintained for 5 min. Initial chromatographic conditions were restored in 2 min.

Data were acquired with the Triple TOF 5600 System (AB SCIEX, USA) fitted with a Nanospray III source (AB SCIEX, USA) and a pulled quartz tip as the emitter (New Objectives, USA). Conditions included an ion spray voltage of 2.5 kV, curtain gas at 30 PSI, nebulizer gas at 15 PSI, and an interface heater temperature of 150 °C. The MS was operated with an RP ≥ 30,000 FWHM for the TOF MS scans. For Information Dependent Acquisition, survey scans were acquired in 250 ms and as many as 30 product ion scans were collected if they exceeded a threshold of 120 counts per second, with a 2+ to 5+ charge-state. The total cycle time was fixed to 3.3 s and the Q2 transmission window was 100 Da for 100%.

Four time bins were summed for each scan at a pulser frequency value of 11 kHz. This was achieved by monitoring a 40 GHz multichannel TDC detector with four-anode/channel detection. A sweeping collision energy setting of 35 ± 5 eV coupled with iTRAQ adjust rolling collision energy was applied to all precursor ions for collision-induced dissociation. Dynamic exclusion was set for 1/2 of the peak width (18 s), and the precursor was then refreshed off the exclusion list.

### Database analysis and quantification

Mascot software (version 2.3.02, Matrix Science) was used to identify and quantify proteins simultaneously. Searches were made against the NCBI non-redundant database that contains mammalian proteins (31786 sequences). Spectra from the 12 fractions were combined into one Mascot Generic Format (MGF) file after the raw data were loaded. The MGF file was searched using parameters that included (i) trypsin, chosen as the enzyme with one missed cleavage allowed; (ii) the fixed modifications of carbamidomethylation, set as Cys; and iii) peptide tolerance, set as 0.05 Da with MS/MS tolerance set as 0.1 Da. An automatic decoy database search strategy was employed to estimate the false discovery rate (FDR), which was calculated as the number of false positive matches divided by the total matches. In the final search results, the FDR was below 1.5%. All search results were passed through additional filters before data exportation. For protein identification, the filters were set as follows: significance threshold *P* < 0.05 (with 95% confidence) and an ion score or expected cutoff of <0.05 (with 95% confidence). For protein quantitation, the filters were set as follows: “median” was chosen for the protein ratio type (16), the minimum precursor charge was set to 2+ and minimum peptides were set to 2, and only 2 and >2 unique peptides were used to quantify proteins. The median intensities were set as normalization, and outliers were removed automatically. The peptide threshold was set as described above for identity (Table S1).

### Bioinformatics analysis

Functional annotations of the differentially expressed proteins were conducted using the Blast2GO program against a non-redundant database consisting of proteins from *Sus scrofa*^37^. The pathway analysis was performed using the Kyoto Encyclopedia of Genes and Genomes (KEGG) and can be accessed at http://www.genome.jp/kegg/. The WEGO program were used to classify and group the differentially expressed proteins^38^. Those groupings were done with Cluster 3.0 and k-means clustering^39^. Up-regulated and down-regulated groups of enriched proteins were selected and the WEGO program was utilized to determine the ontologies for the categories of cellular component, molecular function, and biological process. All GO terms with *P*-values ≤ 0.05 were selected (based on Pearson Chi-Square tests between the numbers of Up and Down proteins)^38^.

### Western blot analysis

Total proteins were extracted using ice-cold RIPA buffer [150 mM NaCl, 1% Triton X-100, 0.5% sodium deoxycholate, 0.1% SDS, and 50 mM Tris-HCl (pH 7.4)] (Biyuntian, Shanghai, China), plus the protease inhibitor cocktail and phosphatase inhibitors (Thermo Scientific, Bremen, Germany). After centrifugation (10,000 × g, 4 °C, 10 min), the protein concentration in the supernatant fluid was determined with a Bicinchoninic Acid assay (Beyotime Biotechnology, China). All samples were adjusted to an equal protein concentration and then diluted with 2 × loading buffer [0.63 mL of 0.5 M Tris-HCl (pH 6.8), 0.42 mL of 75% glycerol, 0.125 g of sodium dodecyl sulfate (SDS), 0.25 mL of β-mercaptoethanol, 0.2 mL of 0.05% bromophenol blue solution, and 1 mL of water] to a final volume of 2.5 mL before being heated in boiling water for 5 min. After the soluble proteins were subjected to SDS-PAGE, the products were transferred to polyvinylidene fluoride membranes (Millipore, Billerica, MA, USA), then blocked with 5% nonfat milk in TBS-0.05% Tween-20 for 1 h. Following overnight incubation with primary antibodies, the samples were treated with horseradish peroxidase-linked secondary antibodies (Santa Cruz Biotechnology, Santa Cruz, CA, USA). The bound antibodies were used with enhanced chemiluminescence (Applygen Technologies Inc., Beijing, China) for detection^40^. The western blot analysis utilized antibodies for eIF4E, Cyclin A, and PCNA (Santa Cruz Biotechnology, Santa Cruz, CA, USA); and for S6K, phospho-S6K (Thr389), mTOR, phospho-mTOR (Ser2448), 4EBP1, phospho-4EBP1 (Thr70), and β-actin (Cell Signaling Technology, Cedarlane, ON, Canada).

## Additional Information

**How to cite this article**: Yang, H. *et al*. Effects of weaning on intestinal crypt epithelial cells in piglets. *Sci. Rep*. **6**, 36939; doi: 10.1038/srep36939 (2016).

**Publisher’s note:** Springer Nature remains neutral with regard to jurisdictional claims in published maps and institutional affiliations.

## Supplementary Material

Supplementary Information
