# Supplementary material for: Effects of weaning on intestinal crypt epithelial cells in piglets
Source: Sci Rep. 2016 Nov 10;6:36939. doi: 10.1038/srep36939 (PMC5103268; doi:10.1038/srep36939)
Supplement: Supplementary Information [file srep36939-s1.pdf]

Supplementary Information

Effects of weaning on intestinal crypt epithelial cells in piglets

Huansheng Yang[1,2], Xia Xiong[2], Xiaocheng Wang[2], Tiejun Li[2] & Yulong Yin[1,2,*]

[1]Animal Nutrition and Human Health Laboratory, School of Life Sciences, Hunan Normal University, Changsha, China

[2]Chinese Academy of Science, Institute of Subtropical Agriculture, Research Center for Healthy Breeding of Livestock and Poultry, Hunan Engineering and Research Center of Animal and Poultry Science and Key Laboratory for Agroecological Processes in Subtropical Region, Scientific Observation and Experimental Station of Animal Nutrition and Feed Science in South-Central, Ministry of Agriculture, Changsha, China

[*]Correspondence: Yulong Yin

Chinese Academy of Science, Institute of Subtropical Agriculture

Yuandaer Road #644, 410125, Changsha, China

Tel: (86)-731-84619706

Fax: (86)-731-84612685

E-mail: yinyulong@isa.ac.cn (Y. Yin)

Figure S1. KEGG pathway enrichment of differentially expressed proteins in jejunal crypt epithelial cells of weaning piglets. Any protein with ≥1.2-fold or ≤0.8-fold difference in values measured from w1d, w3d, w5d, or w7d samples versus w0d level, and *P*-value ≤0.05, was considered differentially expressed.

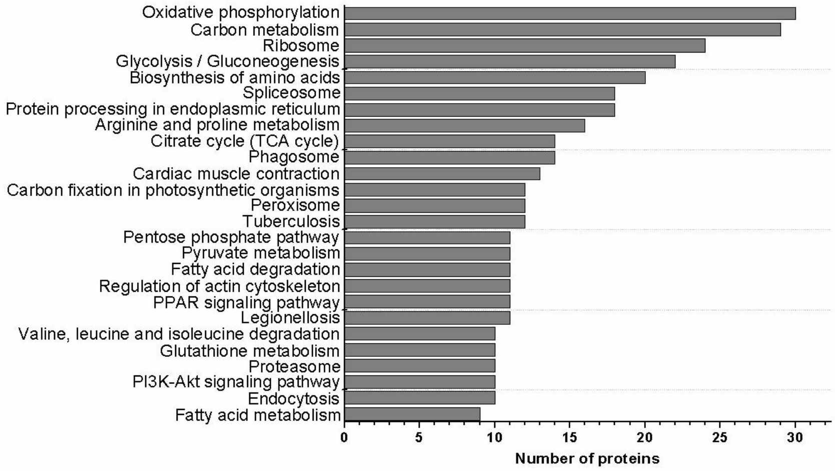

Figure S2. Proteins related to cell cycle in jejunal crypt epithelial cells of weaning piglets. Data are means ± SD; n=5. For each panel, bars not labelled with same superscript indicate values are significantly different at P <0.05.

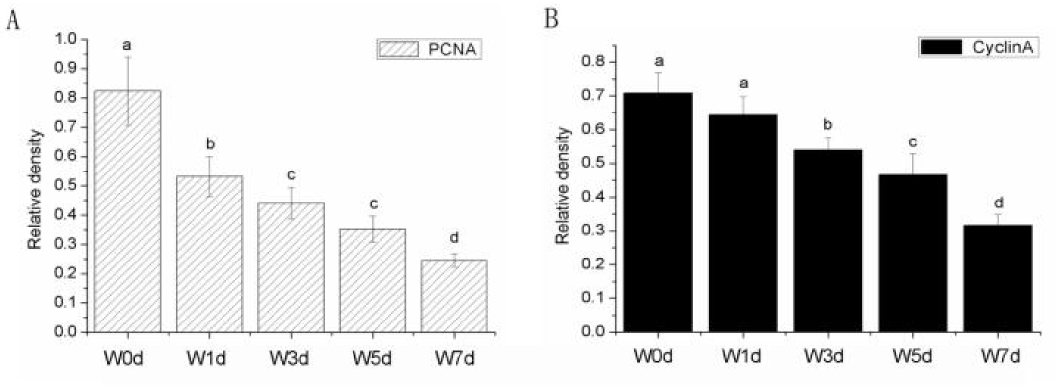

Figure S3. Proteins related to mTOR signaling pathway in jejunal crypt epithelial cells of weaning piglets. Data are means ± SD; n=5. For each panel, bars not labelled with same superscript indicate values are significantly different at $P < 0.05$.

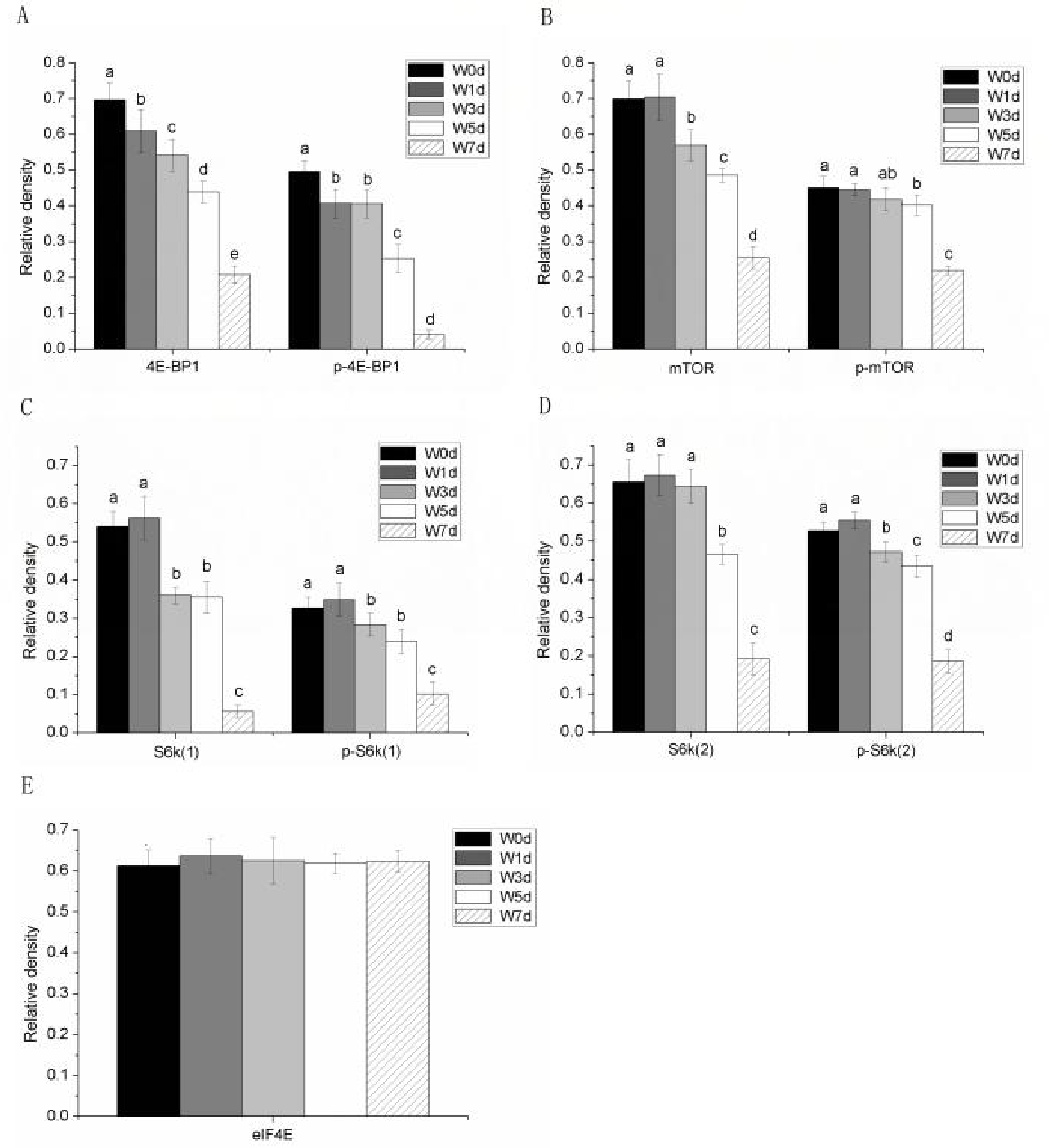

Table S1

Total identified proteins

Accession	Desription	W1d vs W0d	W3d vs W0d	W5d vs W0d	W7d vs W0d
gi|1706761|sp|P49924.1|FABPL_PIG	RecName: Full=Fatty acid-binding protein, liver; AltName: Full=Fatty acid-binding protein 1; AltName: Full=Liver-type fatty acid-binding protein; Short=L-FABP >gi|55742707|ref|NP_001004046.1| fatty acid-binding protein, liver [Sus scrofa] >gi|754910	1.365	1.226	1.187	0.588
gi|71724936|gb|AAZ38898.1|	intestinal fatty acid binding protein [Sus scrofa]	1.479	2.127	2.029	0.869
gi|91206621|sp|Q45KW7.3|FABPI_PIG	RecName: Full=Fatty acid-binding protein, intestinal; AltName: Full=Fatty acid-binding protein 2; AltName: Full=Intestinal-type fatty acid-binding protein; Short=I-FABP >gi|72535172|ref|NP_001026950.1| fatty acid-binding protein, intestinal [Sus scr				
gi|350583843|ref|XP_003126114.3|	PREDICTED: myosin-9 [Sus scrofa]	0.607	0.849	0.925	0.691
gi|1710817|sp|P02632.3|S100G_PIG	RecName: Full=Protein S100-G; AltName: Full=Calbindin-D9k; AltName: Full=S100 calcium-binding protein G; AltName: Full=Vitamin D-dependent calcium-binding protein, intestinal; Short=CABP; Contains: RecName: Full=Protein S100-G, minor isoform >gi|557	1.161	2.371	1.749	1.879
gi|157830532|pdb|1CB1|A	Chain A, Three-Dimensional Solution Structure Of Ca2+-Loaded Porcine Calbindin D9k Determined By Nuclear Magnetic Resonance Spectroscopy				
gi|45269029|gb|AAS55927.1|	cytoskeletal beta actin, partial [Sus scrofa]	1.089	0.585	0.609	0.964
gi|150438831|sp|Q6QAQ1.2|	RecName: Full=Actin, cytoplasmic 1;				

ACTB_PIG	AltName: Full=Beta-actin; Contains: RecName: Full=Actin, cytoplasmic 1, N-terminally processed >gi|311250866|ref|XP_003124328.1| PREDICTED: actin, cytoplasmic 1 [Sus scrofa]				
gi|335297229|ref|XP_003357976.1|	PREDICTED: actin, cytoplasmic 2 [Sus scrofa]				
gi|169139278|gb|ACA48587.1|	liver fatty acid binding protein [Sus scrofa]	0.903	1.628	1.294	0.917
gi|227430407|ref|NP_001153087.1|	keratin, type II cytoskeletal 8 [Sus scrofa]	0.605	0.616	0.767	0.663
gi|194037554|ref|XP_001929445.1|	PREDICTED: ATP synthase subunit beta, mitochondrial [Sus scrofa]	0.748	0.423	0.355	0.607
gi|297591975|ref|NP_001172071.1|	ATP synthase subunit alpha, mitochondrial [Sus scrofa] >gi|187370717|gb|ACD02421.1| cardiac muscle ATP synthase H+ transporting mitochondrial F1 complex alpha subunit 1 [Sus scrofa]	0.768	0.706	0.723	0.799
gi|350585373|ref|XP_003127406.3|	PREDICTED: myosin-14-like [Sus scrofa]	0.667	0.776	0.842	0.605
gi|359811347|ref|NP_001241645.1|	60 kDa heat shock protein, mitochondrial [Sus scrofa] >gi|358009189|gb|AET99216.1| mitochondrial heat shock 60 kDa protein 1 [Sus scrofa]	0.742	0.835	0.735	0.78
gi|358009193|gb|AET99218.1|	prolyl 4-hydroxylase beta polypeptide [Sus scrofa]	0.456	0.471	0.513	0.357
gi|311246557|ref|XP_003122249.1|	PREDICTED: spectrin alpha chain, brain [Sus scrofa]	0.858	1.364	1.308	1.148
gi|47523848|ref|NP_999562.1|	dihydrolipoyllysine-residue succinyltransferase component of 2-oxoglutarate dehydrogenase complex, mitochondrial precursor [Sus scrofa] >gi|18203301|sp|Q9N0F1.1|ODO2_PIG RecName: Full=Dihydrolipoyllysine-residue succinyltransferase component of 2-ox	0.491	0.642	0.769	0.443
gi|350579657|ref|XP_001927830.3|	PREDICTED: 78 kDa glucose-regulated protein [Sus scrofa]	0.613	0.725	0.723	0.64

gi|350593858|ref|XP_003133682.3|	PREDICTED: carbamoyl-phosphate synthase [ammonia], mitochondrial isoform 1 [Sus scrofa]	1.256	0.64	0.507	0.899
gi|350588218|ref|XP_003129667.3|	PREDICTED: nuclear mitotic apparatus protein 1-like [Sus scrofa]	0.867	0.978	1.042	0.815
gi|48675927|ref|NP_001001632.1|	tropomyosin alpha-3 chain [Sus scrofa] >gi|45272586|gb|AAS57724.1| tropomyosin 3 [Sus scrofa]	0.426	1.649	1.82	1.29
gi|346644743|ref|NP_001231138.1|	calmodulin 3 [Sus scrofa] >gi|346644746|ref|NP_001231139.1| calmodulin 1 [Sus scrofa] >gi|311252670|ref|XP_003125211.1| PREDICTED: calmodulin-like [Sus scrofa]	1.687	2.281	4.172	2.23
gi|335289608|ref|XP_003355931.1|	PREDICTED: alpha-actinin-4-like isoform 2 [Sus scrofa]	1.441	1.12	1.304	1.286
gi|335289612|ref|XP_003127168.2|	PREDICTED: alpha-actinin-4-like isoform 1 [Sus scrofa]				
gi|311273061|ref|XP_001925202.2|	PREDICTED: villin 1 [Sus scrofa]	1.437	1.059	0.998	1.99
gi|85701138|sp|Q3ZD69.1|LMNA_PIG	RecName: Full=Prelamin-A/C; Contains: RecName: Full=Lamin-A/C; Flags: Precursor >gi|162139823|ref|NP_001104727.1| prelamin-A/C [Sus scrofa] >gi|66352014|gb|AAY44741.1| lamin A [Sus scrofa]	0.944	0.905	0.832	0.902
gi|46396062|sp|Q865F1.1|MTP_PIG	RecName: Full=Microsomal triglyceride transfer protein large subunit; Flags: Precursor >gi|47523450|ref|NP_999350.1| microsomal triglyceride transfer protein large subunit precursor [Sus scrofa] >gi|28974486|gb|AAO61497.1| microsomal triglyceride tr	0.965	0.461	0.435	0.601
gi|984249|emb|CAA62352.1|	protein kinase [Sus scrofa]	---	---	---	---
gi|17865698|sp|Q29092.3|ENPL_PIG	RecName: Full=Endoplasmin; AltName: Full=94 kDa glucose-regulated protein; Short=GRP-94; AltName: Full=98 kDa protein kinase; Short=PPK 98; Short=ppk98; AltName: Full=Heat shock protein 90 kDa beta member 1;				

	AltName: Full=gp96 homolog; Flags: Precur				
gi|47523016|ref|NP_999268.1|	endoplasmin precursor [Sus scrofa] >gi|2239253|emb|CAA70347.1| gp96/GRP94 [Sus scrofa]	1.336	0.741	1.314	0.855
gi|343962597|ref|NP_001230636.1|	cytosolic non-specific dipeptidase [Sus scrofa]	1.6	3.758	3.289	2.765
gi|332278130|sp|Q29261.2|VILI_PIG	RecName: Full=Villin-1	1.978	1.393	1.137	1.456
gi|350591286|ref|XP_003132317.3|	PREDICTED: filamin-B-like [Sus scrofa]	1.296	1.839	2.223	1.706
gi|225450|prf||1303334A	valosin precursor	1.408	0.778	0.889	1.181
gi|47523628|ref|NP_999442.1|	aminopeptidase N [Sus scrofa] >gi|1703286|sp|P15145.3|AMPN_PIG RecName: Full=Aminopeptidase N; Short=AP-N; Short=pAPN; AltName: Full=Alanyl aminopeptidase; AltName: Full=Aminopeptidase M; Short=AP-M; AltName: Full=Microsomal aminopeptidase; AltName:	0.377	0.514	0.611	0.898
gi|225735709|ref|NP_001139599.1|	clathrin heavy chain 1 [Sus scrofa] >gi|224492556|emb|CAR65329.1| clathrin heavy chain [Sus scrofa]	1.218	0.807	0.717	1.26
gi|311267276|ref|XP_003131485.1|	PREDICTED: keratin, type I cytoskeletal 19-like [Sus scrofa]	0.627	0.634	0.765	0.792
gi|311264042|ref|XP_003129970.1|	PREDICTED: hypoxia up-regulated protein 1 [Sus scrofa]	0.691	0.661	0.727	0.648
gi|164382|gb|AAA31002.1|	Na+, K+-ATPase alpha-subunit precursor [Sus scrofa]	0.797	0.422	0.473	0.725
gi|283443670|gb|ADB19852.1|	Na+/K+ transporting alpha 1 polypeptide [Sus scrofa]				
gi|225173|prf||1210234A	ATPase alpha,Na/K				
gi|393714792|dbj|BAM28740.1|	sodium/potassium-transporting ATPase subunit alpha-1, partial [Sus scrofa]				
gi|194038728|ref|XP_001929104.1|	PREDICTED: pyruvate kinase isozymes M1/M2 isoform 1 [Sus scrofa]	2.09	1.004	1.201	2.004
gi|345441750|ref|NP_001230836.1|	heat shock 70kDa protein 8 [Sus scrofa]	1.383	0.961	0.98	1.398

gi|311273928|ref|XP_003134106.1|	PREDICTED: nucleophosmin-like isoform 2 [Sus scrofa]	0.919	1.022	1.439	0.668
gi|311273930|ref|XP_003134105.1|	PREDICTED: nucleophosmin-like isoform 1 [Sus scrofa]				
gi|350585579|ref|XP_003481996.1|	PREDICTED: alpha-enolase-like, partial [Sus scrofa]	1.597	1.466	1.565	1.341
gi|54873401|gb|AAV40980.1|	heat shock 60 kDa protein 1 [Sus scrofa]	1.229	0.463	0.45	0.587
gi|311252615|ref|XP_003125184.1|	PREDICTED: ubiquitin-40S ribosomal protein S27a-like [Sus scrofa] >gi|311252617|ref|XP_003125185.1| PREDICTED: ubiquitin-40S ribosomal protein S27a-like [Sus scrofa]	1.316	3.017	2.609	1.82
gi|164414678|emb|CAO81735.1|	Alternative Pig Liver Esterase [Sus scrofa]	0.927	1.243	1.579	1.871
gi|47522754|ref|NP_999127.1|	trifunctional enzyme subunit alpha, mitochondrial [Sus scrofa] >gi|7387634|sp|Q29554.1|ECHA_PIG RecName: Full=Trifunctional enzyme subunit alpha, mitochondrial; AltName: Full=78 kDa gastrin-binding protein; AltName: Full=TP-alpha; Includes: RecName:	---	---	---	---
gi|304365428|ref|NP_001182041.1|	protein disulfide-isomerase A3 precursor [Sus scrofa] >gi|301016769|dbj|BAJ11757.1| glucose regulated protein 58 [Sus scrofa]	0.492	1.192	1.152	0.772
gi|187370723|gb|ACD02424.1|	hydroxyacyl-coenzyme A dehydrogenase/3-ketoacyl-coenzyme A thiolase/enoyl-coenzyme A hydratase alpha subunit [Sus scrofa]	---	---	---	---
gi|350593231|ref|XP_003359478.2|	PREDICTED: lactase-phlorizin hydrolase-like [Sus scrofa]	0.691	0.999	0.733	0.7
gi|47522774|ref|NP_999138.1|	heat shock protein HSP 90-alpha [Sus scrofa] >gi|6016267|sp|O02705.3|HS90A_PIG RecName: Full=Heat shock protein HSP 90-alpha >gi|1945447|gb|AAC48718.1| 90-kDa heat shock protein [Sus scrofa]	1.901	1.11	1.112	1.877

gi|297591979|ref|NP_001172070.1|	ornithine aminotransferase, mitochondrial [Sus scrofa] >gi|166244455|gb|ABY86572.1| mitochondrial ornithine aminotransferase [Sus scrofa]	1.701	1.067	0.856	1.434
gi|335287713|ref|XP_003355423.1|	PREDICTED: tubulin alpha-1C chain isoform 1 [Sus scrofa]	---	---	---	---
gi|311267326|ref|XP_003131510.1|	PREDICTED: keratin, type I cytoskeletal 20-like [Sus scrofa]	0.64	0.647	0.745	0.864
gi|350594669|ref|XP_001926037.4|	PREDICTED: ribosome-binding protein 1 [Sus scrofa]	0.571	1.008	0.992	0.8
gi|356460899|ref|NP_999466.2|	catalase [Sus scrofa]	0.83	1.677	1.644	1.541
gi|2494384|sp|Q29550.1|EST1_PIG	RecName: Full=Liver carboxylesterase; AltName: Full=Proline-beta-naphthylamidase; AltName: Full=Retinyl ester hydrolase; Short=REH; Flags: Precursor >gi|363817363|emb|CCF23015.1| unnamed protein product [Sus scrofa] >gi|1931|emb|CAA44929.1| carboxyl	---	---	---	---
gi|218314621|emb|CAV28734.1|	unnamed protein product [Sus sp.]				
gi|297747302|ref|NP_001172115.1|	aspartyl aminopeptidase [Sus scrofa]	0.913	2.217	1.765	0.631
gi|335283403|ref|XP_003123930.2|	PREDICTED: lamin-B1 [Sus scrofa]	0.698	0.956	0.966	0.827
gi|47522618|ref|NP_999099.1|	vinculin [Sus scrofa] >gi|17979613|gb|AAL50327.1| vinculin [Sus scrofa]	1.921	1.133	1.151	1.468
gi|50403675|sp|P26234.4|VINC_PIG	RecName: Full=Vinculin; AltName: Full=Metavinculin				
gi|194043861|ref|XP_001928233.1|	PREDICTED: tubulin alpha-1D chain [Sus scrofa]	0.839	0.202	0.171	0.878
gi|401871556|pdb|4F5C|A	Chain A, Crystal Structure Of The Spike Receptor Binding Domain Of A Porcine Respiratory Coronavirus In Complex With The Pig Aminopeptidase N Ectodomain >gi|401871557|pdb|4F5C|B Chain B, Crystal Structure Of The Spike Receptor Binding Domain Of	---	---	---	---

	A Po				
gi|343790893|ref|NP_001230504.1|	creatine kinase, brain [Sus scrofa]	1.616	0.641	0.774	0.917
gi|47523572|ref|NP_999411.1|	liver carboxylesterase precursor [Sus scrofa] >gi|3831588|gb|AAC70013.1| carboxylesterase [Sus scrofa]	1.484	1.46	2.039	2.172
gi|311250237|ref|XP_003124024.1|	PREDICTED: stress-70 protein, mitochondrial [Sus scrofa]	0.877	0.886	0.721	0.961
gi|350591288|ref|XP_003483241.1|	PREDICTED: filamin-B [Sus scrofa]	1.059	1.552	1.546	1.5
gi|346986466|ref|NP_001231384.1|	proteasome (prosome, macropain) subunit, beta type, 4 [Sus scrofa]	1.495	2.265	3.011	1.72
gi|347300207|ref|NP_001231417.1|	nucleobindin-1 precursor [Sus scrofa]	0.672	1.095	1.007	0.739
gi|47522738|ref|NP_999119.1|	aconitate hydratase, mitochondrial precursor [Sus scrofa] >gi|113159|sp|P16276.1|ACON_PIG RecName: Full=Aconitate hydratase, mitochondrial; Short=Aconitase; AltName: Full=Citrate hydro-lyase; Flags: Precursor >gi|164315|gb|AAA30987.1| heart aconitas	1.77	1.054	1.058	1.686
gi|157837131|pdb|7ACN|A	Chain A, Crystal Structures Of Aconitase With Isocitrate And Nitroisocitrate Bound >gi|157837084|pdb|6ACN|A Chain A, Structure Of Activated Aconitase. Formation Of The (4fe-4s) Cluster In The Crystal >gi|157837014|pdb|5ACN|A Chain A, Structure Of Ac				
gi|335303414|ref|XP_001928370.3|	PREDICTED: tubulin alpha-4A chain [Sus scrofa]	1.239	0.76	0.683	1.233
gi|350591605|ref|XP_003483304.1|	PREDICTED: plastin-1-like [Sus scrofa]	1.46	1.001	0.985	1.418
gi|349501107|ref|NP_001231795.1|	ribosomal protein, large, P2 [Sus scrofa]	0.63	1.484	1.372	0.685
gi|350579435|ref|XP_003122094.3|	PREDICTED: fructose-bisphosphate aldolase B-like, partial [Sus scrofa]	1.468	1.198	1.169	1.175
gi|392513715|ref|NP_001254774.1|	heterogeneous nuclear ribonucleoprotein K [Sus scrofa]	1.038	1.824	1.233	1.272

gi|47523730|ref|NP_999500.1|	tropomyosin alpha-4 chain [Sus scrofa] >gi|54039746|sp|P67937.3|TPM4_PIG RecName: Full=Tropomyosin alpha-4 chain; AltName: Full=Tropomyosin-4 >gi|3661527|gb|AAC61744.1| tropomyosin 4 [Sus scrofa] >gi|262263207|dbj|BAI48106.1| tropomyosin 4 [Sus scro	0.496	0.857	0.634	0.489
gi|311259005|ref|XP_003127890.1|	PREDICTED: myosin regulatory light chain 12B isoform 2 [Sus scrofa] >gi|311259003|ref|XP_003127889.1| PREDICTED: myosin regulatory light chain 12B isoform 1 [Sus scrofa]	0.262	0.4	0.545	0.347
gi|346716324|ref|NP_001231180.1|	myosin regulatory light chain 2 protein isoform 2 [Sus scrofa]				
gi|999620|pdb|1MLD|D	Chain D, Refined Structure Of Mitochondrial Malate Dehydrogenase From Porcine Heart And The Consensus Structure For Dicarboxylic Acid Oxidoreductases >gi|999618|pdb|1MLD|B Chain B, Refined Structure Of Mitochondrial Malate Dehydrogenase From Porcine	2.014	1.057	0.879	1.832
gi|2506849|sp|P00346.2|MDHM_PIG	RecName: Full=Malate dehydrogenase, mitochondrial; Flags: Precursor				
gi|350581138|ref|XP_003124036.3|	PREDICTED: catenin alpha-1 isoform 1 [Sus scrofa]	1.094	0.881	0.907	0.976
gi|335306989|ref|XP_003130411.2|	PREDICTED: ATP-dependent RNA helicase A-like [Sus scrofa]	1.146	0.78	0.777	0.994
gi|350593236|ref|XP_003359477.2|	PREDICTED: lactase-phlorizin hydrolase-like, partial [Sus scrofa]	---	---	---	---
gi|311247536|ref|XP_003122689.1|	PREDICTED: bifunctional ATP-dependent dihydroxyacetone kinase/FAD-AMP lyase (cyclizing)-like [Sus scrofa]	1.863	0.874	0.935	1.222
gi|335280610|ref|XP_003353615.1|	PREDICTED: LOW QUALITY PROTEIN: talin-1 [Sus scrofa]	1.338	1.018	1.273	1.171
gi|268607671|ref|NP_001161267.1|	actin, alpha skeletal muscle [Sus scrofa] >gi|62287931|sp|P68137.1|AC	1.216	0.517	0.418	1.334

<table>
<tr><td></td><td>TS_PIG RecName: Full=Actin, alpha skeletal muscle; AltName: Full=Alpha-actin-1; Flags: Precursor >gi|790202|gb|AAC48692.1| skeletal alpha actin [Sus scrofa]</td><td></td><td></td><td></td><td></td></tr>
<tr><td>gi|281427360|ref|NP_001163988.1|</td><td>actin, alpha cardiac muscle 1 [Sus scrofa] >gi|210077998|emb|CAR82077.1| cardiac muscle alpha actin 1 [Sus scrofa] >gi|210077994|emb|CAR82075.1| cardiac muscle alpha actin 1 [Sus scrofa] >gi|210077996|emb|CAR82076.1| cardiac muscle alpha actin 1 [Su</td><td></td><td></td><td></td><td></td></tr>
<tr><td>gi|56748897|sp|Q6S4N2.1|HS71B_PIG</td><td>RecName: Full=Heat shock 70 kDa protein 1B; AltName: Full=Heat shock 70 kDa protein 2; Short=HSP70.2 >gi|47523308|ref|NP_998931.1| heat shock 70 kDa protein 1B [Sus scrofa] >gi|39777368|gb|AAR30953.1| heat shock protein 70.2 [Sus scrofa] >gi|1487249</td><td>1.002</td><td>1.197</td><td>1.071</td><td>0.58</td></tr>
<tr><td>gi|346986428|ref|NP_001231362.1|</td><td>heat shock 90kD protein 1, beta [Sus scrofa]</td><td>1.86</td><td>1.323</td><td>0.967</td><td>2.418</td></tr>
<tr><td>gi|346986249|ref|NP_001231282.1|</td><td>proteasome subunit beta type-1 [Sus scrofa]</td><td>1.43</td><td>2.595</td><td>2.883</td><td>1.853</td></tr>
<tr><td>gi|392513702|ref|NP_001254763.1|</td><td>protein disulfide isomerase family A, member 4 precursor [Sus scrofa]</td><td>0.946</td><td>1.025</td><td>1.023</td><td>1.005</td></tr>
<tr><td>gi|255683394|ref|NP_001157474.1|</td><td>ornithine carbamoyltransferase, mitochondrial [Sus scrofa]</td><td>0.91</td><td>1.058</td><td>1.682</td><td>1.552</td></tr>
<tr><td>gi|304365440|ref|NP_001182048.1|</td><td>protein disulfide-isomerase A6 precursor [Sus scrofa] >gi|301016767|dbj|BAJ11758.1| protein disulfide isomerase P5 [Sus scrofa]</td><td>1.046</td><td>0.969</td><td>0.942</td><td>1.025</td></tr>
<tr><td>gi|164318|gb|AAA30988.1|</td><td>albumin, partial [Sus scrofa]</td><td>1.129</td><td>0.611</td><td>0.569</td><td>0.712</td></tr>
<tr><td>gi|833798|emb|CAA30970.1|</td><td>albumin [Sus scrofa]</td><td></td><td></td><td></td><td></td></tr>
<tr><td>gi|47522844|ref|NP_999174.1|</td><td>complement C3 precursor [Sus scrofa] >gi|11869931|gb|AAG40565.1|AF154933_1 complement component C3 [Sus scrofa] >gi|27801787|emb|CAD38823.2| complement C3 precursor [Sus scrofa]</td><td>0.64</td><td>0.917</td><td>0.806</td><td>1.028</td></tr>
</table>

gi|311259466|ref|XP_003128111.1|	PREDICTED: putative hydroxypyruvate isomerase-like isoform 1 [Sus scrofa]	1.019	2.107	1.793	1.053
gi|112980811|gb|ABI29187.1|	glyceraldehyde-3-phosphate dehydrogenase [Sus scrofa]	2.033	1.297	0.982	2.414
gi|329744642|ref|NP_001193288.1|	glyceraldehyde-3-phosphate dehydrogenase [Sus scrofa] >gi|2506441|sp|P00355.4|G3P_PIG RecName: Full=Glyceraldehyde-3-phosphate dehydrogenase; Short=GAPDH; AltName: Full=Peptidyl-cysteine S-nitrosylase GAPDH				
gi|47522864|ref|NP_999186.1|	unconventional myosin-VI [Sus scrofa] >gi|75039721|sp|Q29122.1|MYO6_PIG RecName: Full=Unconventional myosin-VI; AltName: Full=Unconventional myosin-6 >gi|516155|emb|CAA84559.1| unconventional myosin [Sus scrofa]	0.847	0.744	0.911	0.63
gi|230506|pdb|2FBP|A	Chain A, Structure Refinement Of Fructose-1,6-Bisphosphatase And Its Fructose 2,6-Bisphosphate Complex At 2.8 Angstroms Resolution >gi|230507|pdb|2FBP|B Chain B, Structure Refinement Of Fructose-1,6-Bisphosphatase And Its Fructose 2,6-Bisphosphate C	1.622	1.109	0.814	0.793
gi|99032141|pdb|2F3H|A	Chain A, Mechanism Of Displacement Of A Catalytically Essential Loop From The Active Site Of Fructose-1,6-Bisphosphatase >gi|99032142|pdb|2F3H|B Chain B, Mechanism Of Displacement Of A Catalytically Essential Loop From The Active Site Of Fructose-1,				
gi|24987483|pdb|1KZ8|A	Chain A, Crystal Structure Of Porcine Fructose-1,6-Bisphosphatase Complexed With A Novel Allosteric-Site Inhibitor >gi|24987484|pdb|1KZ8|F				

	Chain F, Crystal Structure Of Porcine Fructose-1,6-Bisphosphatase Complexed With A Novel Allosteric-Site Inhib				
gi|47522784|ref|NP_999144.1|	fructose-1,6-bisphosphatase 1 [Sus scrofa] >gi|1706735|sp|P00636.4|F16P1_PIG RecName: Full=Fructose-1,6-bisphosphatase 1; Short=FBPase 1; AltName: Full=D-fructose-1,6-bisphosphate 1-phosphohydrolase 1 >gi|300654514|emb|CBV02317.1| unnamed protein pr				
gi|33357497|pdb|1NV1|A	Chain A, Fructose-1,6-Bisphosphatase Complex With Magnesium, Fructose-6- Phosphate, Phosphate And Thallium (5 Mm) >gi|159795376|pdb|2QVV|A Chain A, Porcine Liver Fructose-1,6-Bisphosphatase Cocrystallized With Fru-2,6-P2 And Zn2+, I(T)-State >gi|159				
gi|24987566|pdb|1LEV|F	Chain F, Porcine Kidney Fructose-1,6-Bisphosphatase Complexed With An Amp-Site Inhibitor >gi|24987565|pdb|1LEV|A Chain A, Porcine Kidney Fructose-1,6-Bisphosphatase Complexed With An Amp-Site Inhibitor				
gi|3288991|gb|AAC25597.1|	fructose-1,6-bisphosphatase [Sus scrofa]				
gi|387912908|sp|P28491.3|CALR_PIG	RecName: Full=Calreticulin; AltName: Full=CRP55; AltName: Full=Calregulin; AltName: Full=Endoplasmic reticulum resident protein 60; Short=ERp60; AltName: Full=HACBP; Flags: Precursor >gi|291622246|ref|NP_001167604.1| calreticulin precursor [Sus scro	0.91	0.589	0.581	0.836
gi|343887360|ref|NP_001230	proteasome (prosome, macropain)	1.076	1.677	1.637	1.449

581.1|	subunit, alpha type [Sus scrofa]				
gi|350276134|ref|NP_001231877.1|	LETM1 and EF-hand domain-containing protein 1, mitochondrial [Sus scrofa]	0.568	0.701	0.726	0.558
gi|51701269|sp|Q95334.1|AMPE_PIG	RecName: Full=Glutamyl aminopeptidase; Short=EAP; AltName: Full=Aminopeptidase A; Short=AP-A; AltName: CD_antigen=CD249 >gi|47522856|ref|NP_999182.1| glutamyl aminopeptidase [Sus scrofa] >gi|1518865|gb|AAB07141.1| aminopeptidase A [Sus scrofa]	0.519	0.634	0.513	0.467
gi|347446687|ref|NP_001231527.1|	glucosidase 2 subunit beta precursor [Sus scrofa]	0.456	0.593	0.593	0.549
gi|194037005|ref|XP_001928988.1|	PREDICTED: ribonuclease UK114-like isoform 1 [Sus scrofa]	1.117	2.581	2.299	1.141
gi|335281715|ref|XP_003122673.2|	PREDICTED: L-asparaginase-like [Sus scrofa]	1.844	2.26	2.082	1.32
gi|223046|prf||0410468A	aminotransferase,Asp	1.169	1.787	1.918	1.829
gi|113205892|ref|NP_001038077.1|	tubulin beta chain [Sus scrofa] >gi|75045190|sp|Q767L7.1|TBB5_PIG RecName: Full=Tubulin beta chain; AltName: Full=Tubulin beta-5 chain >gi|81174748|gb|ABB58916.1| beta 5-tubulin [Sus scrofa] >gi|41529175|dbj|BAD08435.1| beta 5-tubulin [Sus scrofa]	0.788	0.442	0.456	0.987
gi|335282386|ref|XP_003354050.1|	PREDICTED: elongation factor 2 [Sus scrofa]	1.833	1.1	0.974	1.955
gi|311254317|ref|XP_001927973.2|	PREDICTED: cingulin-like [Sus scrofa]	0.549	0.991	0.92	0.625
gi|219522018|ref|NP_001137196.1|	Na(+)/H(+) exchange regulatory cofactor NHE-RF1 [Sus scrofa] >gi|217031226|gb|ACJ74160.1| solute carrier family 9 (sodium/hydrogen exchanger) member 3 regulator 1 [Sus scrofa]	1.571	2.608	2.407	1.557
gi|194018702|ref|NP_001123419.1|	tropomyosin beta chain [Sus scrofa] >gi|83778524|gb|ABC47143.1| beta-tropomyosin [Sus scrofa]	---	---	---	---
gi|350595475|ref|XP_003360	PREDICTED: proteasome subunit	0.713	2.004	2.133	1.102

<table>
<tr><td>255.2|</td><td>alpha type-2-like [Sus scrofa]</td><td></td><td></td><td></td><td></td></tr>
<tr><td>gi|335281298|ref|XP_003122400.2|</td><td>PREDICTED: tubulin beta-2C chain-like [Sus scrofa]</td><td>---</td><td>---</td><td>---</td><td>---</td></tr>
<tr><td>gi|330417958|ref|NP_001155225.1|</td><td>phosphoenolpyruvate carboxykinase [GTP], mitochondrial [Sus scrofa] >gi|238863892|gb|ACR66222.1| phosphoenolpyruvate carboxykinase 2 [Sus scrofa]</td><td>2.689</td><td>1.25</td><td>0.996</td><td>1.532</td></tr>
<tr><td>gi|4033507|sp|P08132.2|ANXA4_PIG</td><td>RecName: Full=Annexin A4; AltName: Full=35-beta calcimedin; AltName: Full=Annexin IV; AltName: Full=Annexin-4; AltName: Full=Chromobindin-4; AltName: Full=Endonexin I; AltName: Full=Lipocortin IV; AltName: Full=P32.5; AltName: Full=PP4-X; AltName: F</td><td>0.597</td><td>0.527</td><td>0.325</td><td>0.539</td></tr>
<tr><td>gi|311252000|ref|XP_003124873.1|</td><td>PREDICTED: fumarylacetoacetate hydrolase domain-containing protein 2-like isoform 2 [Sus scrofa]</td><td>1.049</td><td>1.282</td><td>1.147</td><td>0.795</td></tr>
<tr><td>gi|194018698|ref|NP_001123442.1|</td><td>cytochrome c [Sus scrofa] >gi|119388070|sp|P62895.2|CYC_PIG RecName: Full=Cytochrome c >gi|62208258|gb|AAX77008.1| cytochrome c-like protein [Sus scrofa]</td><td>1.081</td><td>2.968</td><td>2.164</td><td>1.501</td></tr>
<tr><td>gi|194039882|ref|XP_001928584.1|</td><td>PREDICTED: histone H2B type 1-H-like [Sus scrofa]</td><td>---</td><td>---</td><td>---</td><td>---</td></tr>
<tr><td>gi|335291884|ref|XP_003356615.1|</td><td>PREDICTED: histone H2B type 1-like [Sus scrofa] >gi|194039814|ref|XP_001927752.1| PREDICTED: histone H2B type 1-like [Sus scrofa]</td><td></td><td></td><td></td><td></td></tr>
<tr><td>gi|335287029|ref|XP_003355256.1|</td><td>PREDICTED: histone H2B type 2-F-like isoform 1 [Sus scrofa]</td><td></td><td></td><td></td><td></td></tr>
<tr><td>gi|335287031|ref|XP_003355257.1|</td><td>PREDICTED: histone H2B type 2-F-like isoform 2 [Sus scrofa]</td><td></td><td></td><td></td><td></td></tr>
<tr><td>gi|335291890|ref|XP_003356617.1|</td><td>PREDICTED: histone H2B type 1-N-like [Sus scrofa]</td><td></td><td></td><td></td><td></td></tr>
<tr><td>gi|343887370|ref|NP_001230585.1|</td><td>histone H2B type 1-D [Sus scrofa] >gi|194039822|ref|XP_001927974.1| PREDICTED: histone H2B</td><td></td><td></td><td></td><td></td></tr>
</table>

	type 1-M-like [Sus scrofa]				
gi|311259875|ref|XP_003128266.1|	PREDICTED: histone H2B type 1-K-like [Sus scrofa]				
gi|544445|sp|P80031.2|GSTP1_PIG	RecName: Full=Glutathione S-transferase P; AltName: Full=GST P1-1; AltName: Full=GST class-pi	1.283	1.047	1.337	1.24
gi|1943419|pdb|2GSR|B	Chain B, Structure Of Porcine Class Pi Glutathione S-Transferase >gi|1943418|pdb|2GSR|A Chain A, Structure Of Porcine Class Pi Glutathione S-Transferase				
gi|253577|gb|AAB22893.1|	myosin light chain isoform LC17a [swine, aorta smooth muscle, Peptide, 150 aa]	0.258	0.611	0.537	0.407
gi|255683363|ref|NP_001157469.1|	myosin light polypeptide 6 [Sus scrofa] >gi|47606435|sp|P60662.2|MYL6_PIG RecName: Full=Myosin light polypeptide 6; AltName: Full=17 kDa myosin light chain; Short=LC17; AltName: Full=Myosin light chain 3; Short=MLC-3; AltName: Full=Myosin light chai				
gi|253578|gb|AAB22894.1|	myosin light chain isoform LC17b [swine, aorta smooth muscle, Peptide, 150 aa]				
gi|335287046|ref|XP_003355261.1|	PREDICTED: histone H2B type 1-N-like [Sus scrofa]	---	---	---	---
gi|350583430|ref|XP_003481516.1|	PREDICTED: histone H2B type 2-E-like [Sus scrofa] >gi|350583421|ref|XP_003355262.2| PREDICTED: histone H2B type 2-E-like [Sus scrofa] >gi|350583436|ref|XP_003481518.1| PREDICTED: histone H2B type 2-E-like [Sus scrofa] >gi|335287018|ref|XP_003355253.				
gi|335310903|ref|XP_003123670.2|	PREDICTED: histone H2B type 1-L-like [Sus scrofa]				
gi|350585077|ref|XP_003481871.1|	PREDICTED: hypothetical protein LOC100739300 [Sus scrofa]	0.914	1.823	1.807	2.044
gi|350585079|ref|XP_003127070.3|	PREDICTED: xaa-Pro dipeptidase [Sus scrofa]				

gi|281500757|pdb|3FX4|A	Chain A, Porcine Aldehyde Reductase In Ternary Complex With Inhibitor >gi|229597975|pdb|3H4G|A Chain A, Structure Of Aldehyde Reductase Holoenzyme In Complex With Potent Aldose Reductase Inhibitor Fidarestat: Implications For Inhibitor Binding And S	3.159	1.61	1.18	2.261
gi|114689|sp|P13618.1|ATP5J_PIG	RecName: Full=ATP synthase-coupling factor 6, mitochondrial; Short=ATPase subunit F6	0.351	0.632	0.768	0.496
gi|335309281|ref|XP_003361573.1|	PREDICTED: ATP synthase-coupling factor 6, mitochondrial-like isoform 1 [Sus scrofa] >gi|335300559|ref|XP_003358937.1| PREDICTED: ATP synthase-coupling factor 6, mitochondrial-like [Sus scrofa]				
gi|335309283|ref|XP_003361574.1|	PREDICTED: ATP synthase-coupling factor 6, mitochondrial-like isoform 2 [Sus scrofa]				
gi|229001074|emb|CAX20316.1|	mannose-6-phosphate receptor binding protein 1 [Sus scrofa]	1.122	1.017	0.952	1.056
gi|350591696|ref|XP_003483317.1|	PREDICTED: sucrase-isomaltase, intestinal-like [Sus scrofa]	0.913	1	0.975	0.943
gi|148222268|ref|NP_001090952.1|	tropomyosin alpha-1 chain [Sus scrofa] >gi|158931149|sp|P42639.2|TPM1_PIG RecName: Full=Tropomyosin alpha-1 chain; AltName: Full=Alpha-tropomyosin; AltName: Full=Tropomyosin-1 >gi|117661180|gb|ABK55659.1| TPM1 [Sus scrofa]	0.641	1.115	1.002	0.688
gi|54020966|ref|NP_001005726.1|	annexin A2 [Sus scrofa] >gi|148876771|sp|P19620.4|ANXA2_PIG RecName: Full=Annexin A2; AltName: Full=Annexin II; AltName: Full=Annexin-2; AltName: Full=Calpactin I heavy chain; AltName: Full=Calpactin-1 heavy chain; AltName:	0.905	0.473	0.453	0.667

	Full=Chromobindin-8; AltN				
gi|350587379|ref|XP_003482398.1|	PREDICTED: putative aminopeptidase C13A11.05-like [Sus scrofa]	2.105	2.722	3.623	0.835
gi|1710093|sp|P50121.2|RET2_PIG	RecName: Full=Retinol-binding protein 2; AltName: Full=Cellular retinol-binding protein II; Short=CRBP-II >gi|55741711|ref|NP_999616.1| retinol-binding protein 2 [Sus scrofa] >gi|455523|emb|CAA54729.1| cellular retinol binding protein II [Sus scrofa	1.386	0.766	0.8	0.777
gi|335284397|ref|XP_003354597.1|	PREDICTED: major vault protein isoform 1 [Sus scrofa]	1.404	1.073	1.002	1.37
gi|335305925|ref|XP_003135119.2|	PREDICTED: LOW QUALITY PROTEIN: ubiquitin-like modifier activating enzyme 1 [Sus scrofa]	1.971	0.991	0.967	1.697
gi|51592135|ref|NP_001004043.1|	cofilin-1 [Sus scrofa] >gi|116850|sp|P10668.3|COF1_PIG RecName: Full=Cofilin-1; AltName: Full=Cofilin, non-muscle isoform >gi|164425|gb|AAA31020.1| cofilin [Sus scrofa]	1.814	1.452	0.952	1.469
gi|340007404|ref|NP_001229990.1|	alpha-actinin-1 [Sus scrofa]	1.027	0.914	0.831	0.877
gi|311251248|ref|XP_003124516.1|	PREDICTED: apoptosis-associated speck-like protein containing a CARD-like isoform 1 [Sus scrofa]	3.139	6.012	6.925	3.814
gi|335284318|ref|XP_003354571.1|	PREDICTED: apoptosis-associated speck-like protein containing a CARD-like isoform 2 [Sus scrofa]				
gi|335299026|ref|XP_003132223.2|	PREDICTED: microtubule-associated protein 4-like [Sus scrofa]	0.901	1.857	1.532	1.377
gi|350583970|ref|XP_003126216.3|	PREDICTED: keratin, type II cytoskeletal 75-like [Sus scrofa]	0.448	0.68	0.76	0.546
gi|51317314|sp|P62802.2|H4_PIG	RecName: Full=Histone H4 >gi|350583428|ref|XP_003481515.1| PREDICTED: histone H4-like [Sus scrofa] >gi|335291880|ref|XP_003356614.1| PREDICTED: histone H4-like [Sus	0.435	0.532	0.445	0.417

	scrofa] >gi|335287016|ref|XP_003355252.1| PREDICTED: histone H4-like [Sus scrofa] >g				
gi|218664491|ref|NP_001136314.1|	galectin-3 [Sus scrofa] >gi|215254098|gb|ACJ64061.1| lectin galactoside-binding soluble 2 protein [Sus scrofa]	1.047	1.249	0.849	0.939
gi|346986388|ref|NP_001231345.1|	profilin-1 [Sus scrofa]	2.107	1.375	1.305	2.004
gi|112976|sp|P00503.3|AATC_PIG	RecName: Full=Aspartate aminotransferase, cytoplasmic; AltName: Full=Glutamate oxaloacetate transaminase 1; AltName: Full=Transaminase A >gi|47522636|ref|NP_999092.1| aspartate aminotransferase, cytoplasmic [Sus scrofa] >gi|300557343|emb|CBV09643.1|	1.113	2.697	3.518	2.144
gi|350584176|ref|XP_003481686.1|	PREDICTED: myosin-Ia-like [Sus scrofa]	0.968	0.862	0.644	1.164
gi|350582932|ref|XP_003125549.2|	PREDICTED: annexin A13 isoform 2 [Sus scrofa]	0.38	0.322	0.384	0.386
gi|350585375|ref|XP_003127408.3|	PREDICTED: myosin-14 [Sus scrofa]	0.739	0.612	0.67	0.696
gi|347300325|ref|NP_001231461.1|	dehydrogenase/reductase SDR family member 11 [Sus scrofa]	2.52	1.354	1.392	1.907
gi|335281609|ref|XP_003353842.1|	PREDICTED: stress-induced-phosphoprotein 1-like [Sus scrofa]	1.48	1.786	1.834	1.491
gi|194044822|ref|XP_001927404.1|	PREDICTED: peroxiredoxin-4 [Sus scrofa]	0.45	1.472	1.707	1.213
gi|311252459|ref|XP_003125105.1|	PREDICTED: poly(rC)-binding protein 1-like [Sus scrofa]	1.293	1.231	1.286	1.261
gi|290463427|sp|Q0QF01.2|DHSA_PIG	RecName: Full=Succinate dehydrogenase [ubiquinone] flavoprotein subunit, mitochondrial; AltName: Full=Flavoprotein subunit of complex II; Short=Fp; Flags: Precursor	0.678	0.605	0.674	0.695
gi|322812294|pdb|3AE8|A	Chain A, Crystal Structure Of Porcine Heart Mitochondrial Complex Ii Bound With				

	N-(3-Isopropoxy-Phenyl)-2-Trifluoromethylbenzamide >gi|345531796|pdb|3SFE|A Chain A, Crystal Structure Of Porcine Mitochondrial Respiratory Complex Ii Bound With Oxaloac				
gi|47523768|ref|NP_999520.1|	epoxide hydrolase 1 precursor [Sus scrofa] >gi|75069447|sp|P79381.1|HYEP_PIG RecName: Full=Epoxide hydrolase 1; AltName: Full=Epoxide hydratase; AltName: Full=Microsomal epoxide hydrolase >gi|1840391|dbj|BAA19200.1| epoxide hydrolase [Sus scrofa]	0.733	0.492	0.311	0.608
gi|194037042|ref|XP_001927136.1|	PREDICTED: cadherin-17 [Sus scrofa]	0.516	0.558	0.827	0.472
gi|350593820|ref|XP_003483769.1|	PREDICTED: isocitrate dehydrogenase [NADP] cytoplasmic isoform 3 [Sus scrofa] >gi|350593816|ref|XP_003483767.1| PREDICTED: isocitrate dehydrogenase [NADP] cytoplasmic isoform 1 [Sus scrofa] >gi|350593818|ref|XP_003483768.1| PREDICTED: isocitrate deh	2.425	1.236	1.05	1.383
gi|350583022|ref|XP_001927263.3|	PREDICTED: 14-3-3 protein zeta/delta [Sus scrofa]	3.308	1.517	1.086	2.726
gi|3913046|sp|O46409.1|APOA4_PIG	RecName: Full=Apolipoprotein A-IV; Short=Apo-AIV; Short=ApoA-IV; AltName: Full=Apolipoprotein A4; Flags: Precursor >gi|47523830|ref|NP_999553.1| apolipoprotein A-IV precursor [Sus scrofa] >gi|2695742|emb|CAA11020.1| apolipoprotein A-IV [Sus scrofa]	0.558	0.39	0.478	0.451
gi|350585075|ref|XP_003481870.1|	PREDICTED: xaa-Pro dipeptidase-like [Sus scrofa]	1.176	1.013	1.704	1.131
gi|146741296|dbj|BAF62303.1|	keratin 1 [Sus scrofa]	0.933	1.375	0.846	0.856
gi|350583977|ref|XP_003481635.1|	PREDICTED: keratin, type II cytoskeletal 1 [Sus scrofa]				
gi|311255413|ref|XP_001927240.2|	PREDICTED: keratin, type II cytoskeletal 79 [Sus scrofa]	0.422	0.675	0.754	0.534

gi|83921635|ref|NP_001033089.1|	FKBP1A-like [Sus scrofa] >gi|61098747|gb|AAX37547.1| FKBP1A-like [Sus scrofa]	1.011	1.849	1.72	1.154
gi|112985|sp|P00506.2|AATM_PIG	RecName: Full=Aspartate aminotransferase, mitochondrial; Short=mAspAT; AltName: Full=Fatty acid-binding protein; Short=FABP-1; AltName: Full=Glutamate oxaloacetate transaminase 2; AltName: Full=Plasma membrane-associated fatty acid-binding protein;	1.009	1.728	2.191	1.62
gi|347658971|ref|NP_001231613.1|	ATP synthase, H+ transporting, mitochondrial Fo complex, subunit d [Sus scrofa]	0.489	0.543	0.446	0.473
gi|311250151|ref|XP_003123979.1|	PREDICTED: heat shock 70 kDa protein 4 [Sus scrofa]	1.453	2.216	1.688	1.224
gi|55583761|sp|Q64L94.1|PSME1_PIG	RecName: Full=Proteasome activator complex subunit 1; AltName: Full=Proteasome activator 28 subunit alpha; Short=PA28a; Short=PA28alpha >gi|34978646|gb|AAQ83574.1| proteasome activator 28 alpha subunit [Sus scrofa]	1.531	1.257	1.253	1.363
gi|350592528|ref|XP_003483480.1|	PREDICTED: endoplasmic reticulum resident protein 29 [Sus scrofa]	0.787	1.304	1.172	0.982
gi|118403904|ref|NP_001072131.1|	protein DJ-1 [Sus scrofa] >gi|67038668|gb|AAY63803.1| DJ-1 protein [Sus scrofa]	1.06	2.156	1.948	1.543
gi|113205888|ref|NP_001038076.1|	aldehyde dehydrogenase, mitochondrial precursor [Sus scrofa] >gi|122138098|sp|Q2XQV4.1|ALDH2_PIG RecName: Full=Aldehyde dehydrogenase, mitochondrial; AltName: Full=ALDH class 2; AltName: Full=ALDH-E2; Flags: Precursor >gi|81295909|gb|ABB70228.1| mit	1.732	0.778	0.734	1.475
gi|187370719|gb|ACD02422.1|	mitochondrial aldehyde dehydrogenase 2 [Sus scrofa]				
gi|350584145|ref|XP_001929467.3|	PREDICTED: nascent polypeptide-associated complex	0.879	1.852	1.608	1.283

	subunit alpha isoform 1 [Sus scrofa]				
gi|350584147|ref|XP_003481679.1|	PREDICTED: nascent polypeptide-associated complex subunit alpha [Sus scrofa]				
gi|350594035|ref|XP_003483822.1|	PREDICTED: UDP-glucuronosyltransferase 1-10 isoform 3 [Sus scrofa]	0.561	0.348	0.404	0.378
gi|213958609|gb|ACJ54701.1|	glutamine:fructose-6-phosphate amidotransferase 1 variant 2 [Sus scrofa]	1.388	0.576	0.609	1.45
gi|303228252|ref|NP_001181908.1|	glucosamine--fructose-6-phosphate aminotransferase [isomerizing] 1 [Sus scrofa] >gi|213958607|gb|ACJ54700.1| glutamine:fructose-6-phosphate amidotransferase 1 variant 1 [Sus scrofa]				
gi|75051712|sp|Q9TUB5.1|CLCA1_PIG	RecName: Full=Calcium-activated chloride channel regulator 1; AltName: Full=Calcium-activated chloride channel family member 1; AltName: Full=pCLCA1; Flags: Precursor >gi|47523388|ref|NP_999313.1| calcium-activated chloride channel regulator 1 precu	1.042	1.503	1.076	1.117
gi|162951821|ref|NP_001106165.1|	heterogeneous nuclear ribonucleoprotein A/B [Sus scrofa] >gi|160858224|dbj|BAF93845.1| CArG-binding factor A [Sus scrofa]	0.998	2.227	1.941	1.736
gi|350276234|ref|NP_001231897.1|	anterior gradient protein 2 homolog precursor [Sus scrofa]	1.123	1.229	1.016	1.215
gi|1364248|emb|CAA82246.1|	glucosephosphate isomerase [Sus scrofa]	2.652	1.874	1.372	2.71
gi|21730305|pdb|1GZD|A	Chain A, Crystal Structure Of Pig Phosphoglucose Isomerase >gi|24987288|pdb|1GZV|A Chain A, The Crystal Structure Of Phosphoglucose Isomerase From Pig Muscle Complexed With 5-Phosphoarabinonate				
gi|47523720|ref|NP_999495.1|	glucose-6-phosphate isomerase [Sus scrofa] >gi|120742|sp|P08059.3|G6PI_PIG RecName:				

	Full=Glucose-6-phosphate isomerase; Short=GPI; AltName: Full=Autocrine motility factor; Short=AMF; AltName: Full=Neuroleukin; Short=NLK; AltName: Full=Phosphoglucose				
gi|350593430|ref|XP_003483683.1|	PREDICTED: glutathione reductase, mitochondrial isoform 1 [Sus scrofa]	0.713	1.184	1.473	1.349
gi|350580243|ref|XP_003123005.3|	PREDICTED: harmonin-like [Sus scrofa]	0.886	2.1	1.767	1.414
gi|194039884|ref|XP_001928592.1|	PREDICTED: histone H2A type 1-like [Sus scrofa]	0.354	0.344	0.267	0.246
gi|335291888|ref|XP_001928674.3|	PREDICTED: histone H2A type 1-F-like [Sus scrofa]				
gi|335291892|ref|XP_003356618.1|	PREDICTED: histone H2A type 1-like [Sus scrofa] >gi|311259877|ref|XP_003128263.1| PREDICTED: histone H2A type 1-like [Sus scrofa] >gi|194039812|ref|XP_001927727.1| PREDICTED: histone H2A type 1-like [Sus scrofa]				
gi|335291882|ref|XP_001928646.3|	PREDICTED: histone H2A type 1-F-like [Sus scrofa]				
gi|335308465|ref|XP_003361239.1|	PREDICTED: histone H2A type 1-like [Sus scrofa] >gi|194039816|ref|XP_001927762.1| PREDICTED: histone H2A type 1-like [Sus scrofa]				
gi|350583438|ref|XP_003481519.1|	PREDICTED: histone H2A type 2-C-like [Sus scrofa]				
gi|350583432|ref|XP_003355263.2|	PREDICTED: histone H2A type 2-A-like [Sus scrofa]				
gi|47523692|ref|NP_999478.1|	thioredoxin [Sus scrofa] >gi|20141821|sp|P82460.3|THIO_PIG RecName: Full=Thioredoxin; Short=Trx >gi|332368598|gb|AEE61373.1| thioredoxin [Sus scrofa] >gi|14326453|gb|AAK60272.1|AF382821_1 thioredoxin [Sus scrofa]	1.007	2.615	2.229	1.258
gi|281427374|ref|NP_001163991.1|	T-complex protein 1 subunit beta [Sus	1.265	0.945	0.666	1.224

	scrofa] >gi|262036935|dbj|BAI47602.1| chaperonin containing TCP1, subunit 2 (beta) [Sus scrofa]				
gi|350596362|ref|XP_003361090.2|	PREDICTED: CD2-associated protein-like [Sus scrofa]	1.357	5.83	3.915	2.031
gi|222136590|ref|NP_001138373.1|	proteasome subunit alpha type-5 [Sus scrofa]	1.041	2.413	2.229	1.879
gi|335285948|ref|XP_003131991.2|	PREDICTED: very long-chain specific acyl-CoA dehydrogenase, mitochondrial-like isoform 1 [Sus scrofa]	1.314	0.725	0.661	0.646
gi|335285950|ref|XP_003354989.1|	PREDICTED: very long-chain specific acyl-CoA dehydrogenase, mitochondrial-like isoform 2 [Sus scrofa]				
gi|2500586|sp|Q29099.1|PTBP1_PIG	RecName: Full=Polypyrimidine tract-binding protein 1; Short=PTB; AltName: Full=Heterogeneous nuclear ribonucleoprotein I; Short=hnRNP I >gi|47523538|ref|NP_999396.1| polypyrimidine tract-binding protein 1 [Sus scrofa] >gi|1122433|emb|CAA63597.1| pol	1.078	2.503	2.591	3.405
gi|350592201|ref|XP_003359029.2|	PREDICTED: hypothetical protein LOC100620809 isoform 1, partial [Sus scrofa]	0.604	0.664	0.798	0.503
gi|335305558|ref|XP_003134901.2|	PREDICTED: heterogeneous nuclear ribonucleoproteins A2/B1 isoform 1 [Sus scrofa]	1.127	0.765	0.745	1.284
gi|346986324|ref|NP_001231316.1|	tumor protein D52 [Sus scrofa]	0.72	0.882	1.075	1.124
gi|47522692|ref|NP_999062.1|	long-chain specific acyl-CoA dehydrogenase, mitochondrial precursor [Sus scrofa] >gi|2829676|sp|P79274.1|ACADL_PIG RecName: Full=Long-chain specific acyl-CoA dehydrogenase, mitochondrial; Short=LCAD; Flags: Precursor >gi|1695729|dbj|BAA13965.1| long	0.734	0.971	0.828	0.632

gi|148747594|ref|NP_001092068.1|	60S acidic ribosomal protein P0 [Sus scrofa] >gi|182705234|sp|Q29214.2|RLA0_PIG RecName: Full=60S acidic ribosomal protein P0; AltName: Full=60S ribosomal protein L10E >gi|83854099|gb|ABC47963.1| ribosomal phosphoprotein large PO subunit [Sus scrofa	0.785	0.612	0.651	0.807
gi|297591965|ref|NP_001172061.1|	leukotriene A-4 hydrolase [Sus scrofa] >gi|262204898|dbj|BAI48029.1| leukotriene A4 hydrolase [Sus scrofa]	1.816	0.956	0.872	1.682
gi|303304923|dbj|BAJ14788.1|	aminopeptidase T [Sus scrofa]				
gi|350586352|ref|XP_003482167.1|	PREDICTED: serpin peptidase inhibitor, clade B (ovalbumin), member 1 [Sus scrofa]	1.608	0.798	0.757	1.136
gi|194033860|ref|XP_001927901.1|	PREDICTED: UV excision repair protein RAD23 homolog B [Sus scrofa]	1.642	2.407	2.937	1.236
gi|347300165|ref|NP_001231397.1|	proteasome subunit alpha type-4 [Sus scrofa]	0.938	1.904	1.898	1.546
gi|299611|gb|AAB26236.1|	cerebroside sulfate activator, CS-Act [swine, kidneys, Peptide, 79 aa]	0.957	0.947	1.163	0.607
gi|17380295|sp|P81405.2|SAP_PIG	RecName: Full=Saposin-B-Val; Contains: RecName: Full=Saposin-B; AltName: Full=Cerebroside sulfate activator; Short=CS-ACT; AltName: Full=Non-specific activator; AltName: Full=Sphingolipid activator protein 1; Short=SAP-1				
gi|311246990|ref|XP_003122442.1|	PREDICTED: mucin-2, partial [Sus scrofa]	1.835	0.865	0.677	1.098
gi|222136592|ref|NP_001138374.1|	proteasome subunit beta type-3 [Sus scrofa]	1.162	1.55	1.872	1.409
gi|68532157|gb|AAY98813.1|	proteasome subunit 10 [Sus scrofa] >gi|68532159|gb|AAY98814.1| proteasome subunit 10 [Sus scrofa]	1.961	1.767	3.009	1.305
gi|113205704|ref|NP_001038030.1|	proteasome subunit beta type-10 [Sus scrofa] >gi|90823108|gb|ABE01081.1| proteasome beta 10 subunit [Sus scrofa]				
gi|194042318|ref|XP_001928	PREDICTED: putative hexokinase	0.716	0.557	0.679	0.825

917.1|	HKDC1 [Sus scrofa]				
gi|350583750|ref|XP_003481579.1|	PREDICTED: hsc70-interacting protein-like isoform 1 [Sus scrofa]	1.648	1.355	1.322	1.35
gi|350583752|ref|XP_003481580.1|	PREDICTED: hsc70-interacting protein-like isoform 2 [Sus scrofa]				
gi|47523764|ref|NP_999518.1|	peptidyl-prolyl cis-trans isomerase A [Sus scrofa] >gi|51702768|sp|P62936.2|PPIA_PIG RecName: Full=Peptidyl-prolyl cis-trans isomerase A; Short=PPIase A; AltName: Full=Cyclophilin A; AltName: Full=Cyclosporin A-binding protein; AltName: Full=Rotamas	2.721	1.273	0.985	2.422
gi|350589126|ref|XP_003482792.1|	PREDICTED: nucleoprotein TPR [Sus scrofa]	0.863	0.852	0.757	0.883
gi|350586779|ref|XP_003482273.1|	PREDICTED: LOW QUALITY PROTEIN: IQ motif containing GTPase activating protein 1 [Sus scrofa]	1.126	0.971	0.793	1.146
gi|311244872|ref|XP_003121596.1|	PREDICTED: creatine kinase U-type, mitochondrial-like isoform 2 [Sus scrofa] >gi|311244870|ref|XP_003121595.1| PREDICTED: creatine kinase U-type, mitochondrial-like isoform 1 [Sus scrofa]	1.676	1.552	1.153	1.306
gi|343791015|ref|NP_001230554.1|	carboxylesterase 3 precursor [Sus scrofa]	0.942	0.662	0.829	0.924
gi|194040624|ref|XP_001929173.1|	PREDICTED: plastin-2 isoform 1 [Sus scrofa]	1.786	1.252	1.164	1.583
gi|162952052|ref|NP_001106151.1|	transketolase [Sus scrofa] >gi|159502444|gb|ABW97521.1| transketolase [Sus scrofa]	1.819	2.627	3.235	3.514
gi|311272935|ref|XP_003133651.1|	PREDICTED: NADH-ubiquinone oxidoreductase 75 kDa subunit, mitochondrial isoform 1 [Sus scrofa]	0.699	0.592	0.38	0.592
gi|335303281|ref|XP_003359671.1|	PREDICTED: NADH-ubiquinone oxidoreductase 75 kDa subunit, mitochondrial isoform 2 [Sus scrofa]				
gi|335302040|ref|XP_001927564.2|	PREDICTED: delta-1-pyrroline-5-carboxylate synthase isoform 1 [Sus scrofa]	0.834	0.466	0.373	0.665

gi|346644799|ref|NP_001231157.1|	thymopoietin [Sus scrofa]	0.723	0.984	0.854	0.716
gi|311252403|ref|XP_003125080.1|	PREDICTED: sepiapterin reductase-like [Sus scrofa]	1.394	1.35	1.971	1.837
gi|1170740|sp|P00339.3|LDHA_PIG	RecName: Full=L-lactate dehydrogenase A chain; Short=LDH-A; AltName: Full=LDH muscle subunit; Short=LDH-M >gi|473571|gb|AAA50436.1| lactate dehydrogenase-A [Sus scrofa domesticus]	1.799	0.825	0.705	1.44
gi|48675953|ref|NP_001001643.1|	rab GDP dissociation inhibitor beta [Sus scrofa] >gi|75043802|sp|Q6Q7J2.1|GDIB_PIG RecName: Full=Rab GDP dissociation inhibitor beta; Short=Rab GDI beta; AltName: Full=Guanosine diphosphate dissociation inhibitor 2; Short=GDI-2 >gi|45758488|gb|AAS76	2.866	1.102	0.916	1.781
gi|335297609|ref|XP_003358079.1|	PREDICTED: synaptic vesicle membrane protein VAT-1 homolog [Sus scrofa]	0.788	0.386	0.515	0.459
gi|350578484|ref|XP_003121491.3|	PREDICTED: NAD-dependent malic enzyme, mitochondrial [Sus scrofa]	1.031	0.947	1.075	1.163
gi|345441771|ref|NP_001230857.1|	aldolase C, fructose-bisphosphate [Sus scrofa]	1.123	0.698	1.148	0.868
gi|71042645|pdb|2BKI|A	Chain A, Myosin Vi Nucleotide-Free (Mdinsert2-Iq) Crystal Structure	---	---	---	---
gi|320089676|pdb|2X51|A	Chain A, M6 Delta Insert1				
gi|73536335|pdb|2BKH|A	Chain A, Myosin Vi Nucleotide-Free (Mdinsert2) Crystal Structure				
gi|162330177|pdb|2VAS|A	Chain A, Myosin Vi (Md-Insert2-Cam, Delta-Insert1) Post-Rigor State >gi|162330179|pdb|2VB6|A Chain A, Myosin Vi (Md-Insert2-Cam, Delta Insert1) Post-Rigor State (Crystal Form 2)				
gi|160877818|pdb|2V26|A	Chain A, Myosin Vi (Md) Pre-Powerstroke State (Mg.Adp.Vo4)				
gi|313754272|pdb|3L9I|A	Chain A, Myosin Vi Nucleotide-Free (Mdinsert2) L310g Mutant Crystal				

	Structure				
gi|122064583|sp|P04178.2|SODC_PIG	RecName: Full=Superoxide dismutase [Cu-Zn] >gi|298677090|ref|NP_001177351.1| superoxide dismutase [Cu-Zn] [Sus scrofa] >gi|297593564|gb|ADI47520.1| superoxide dismutase 1 [Sus scrofa]	0.935	1.54	1.45	1.125
gi|350589456|ref|XP_003357762.2|	PREDICTED: ubiquilin-1 [Sus scrofa]	0.873	1.238	1.248	1.101
gi|47523420|ref|NP_999331.1|	trans-1,2-dihydrobenzene-1,2-diol dehydrogenase [Sus scrofa] >gi|75051805|sp|Q9TV69.1|DHDH_PIG RecName: Full=Trans-1,2-dihydrobenzene-1,2-diol dehydrogenase; AltName: Full=D-xylose 1-dehydrogenase; AltName: Full=D-xylose-NADP dehydrogenase; AltName:	1.382	1.448	1.126	1.422
gi|47522608|ref|NP_999106.1|	dolichyl-diphosphooligosaccharide--protein glycosyltransferase subunit 2 precursor [Sus scrofa] >gi|75056105|sp|Q9GL01.1|RPN2_PIG RecName: Full=Dolichyl-diphosphooligosaccharide--protein glycosyltransferase subunit 2; AltName: Full=Dolichyl-diphosph	0.966	0.55	0.412	0.788
gi|350589336|ref|XP_003130615.3|	PREDICTED: heterogeneous nuclear ribonucleoprotein U [Sus scrofa]	0.796	1.037	0.907	0.885
gi|380509273|gb|AFD64639.1|	peroxisomal acyl-coenzyme A oxidase 1 isoform 1 [Sus scrofa]	1.069	0.86	1.61	0.514
gi|380509275|gb|AFD64640.1|	peroxisomal acyl-coenzyme A oxidase 1 isoform 2 [Sus scrofa]				
gi|350592730|ref|XP_003483523.1|	PREDICTED: heterogeneous nuclear ribonucleoprotein F [Sus scrofa] >gi|335301642|ref|XP_003359253.1| PREDICTED: heterogeneous nuclear ribonucleoprotein F [Sus scrofa] >gi|194042674|ref|XP_001929089.1| PREDICTED: heterogeneous nuclear ribonucleoprotei	1.215	1.29	1.078	1.133

<table>
<tr><td>gi|311254039|ref|XP_003125724.1|</td><td>PREDICTED: mucosal pentraxin-like [Sus scrofa]</td><td>0.42</td><td>0.286</td><td>0.227</td><td>0.291</td></tr>
<tr><td>gi|311248177|ref|XP_003123013.1|</td><td>PREDICTED: proteasome subunit alpha type-1-like [Sus scrofa]</td><td>0.627</td><td>1.485</td><td>1.428</td><td>1.467</td></tr>
<tr><td>gi|347300163|ref|NP_001231396.1|</td><td>succinate-semialdehyde dehydrogenase, mitochondrial [Sus scrofa]</td><td>1.038</td><td>0.391</td><td>0.419</td><td>0.599</td></tr>
<tr><td>gi|347300243|ref|NP_001231430.1|</td><td>glutamate dehydrogenase 1, mitochondrial [Sus scrofa]</td><td>0.826</td><td>0.563</td><td>0.527</td><td>0.727</td></tr>
<tr><td>gi|335309939|ref|XP_003133770.2|</td><td>PREDICTED: nucleolin-like [Sus scrofa]</td><td>1.089</td><td>1.648</td><td>1.607</td><td>1.599</td></tr>
<tr><td>gi|283993079|gb|ADB57036.1|</td><td>L-gulonate 3-dehydrogenase [Sus scrofa]</td><td>2.065</td><td>0.888</td><td>1.278</td><td>1.51</td></tr>
<tr><td>gi|350582175|ref|XP_003481218.1|</td><td>PREDICTED: mitochondrial inner membrane protein, partial [Sus scrofa]</td><td>0.546</td><td>0.513</td><td>0.778</td><td>0.61</td></tr>
<tr><td>gi|1702996|sp|P54612.2|2AAA_PIG</td><td>RecName: Full=Serine/threonine-protein phosphatase 2A 65 kDa regulatory subunit A alpha isoform; AltName: Full=PP2A subunit A isoform PR65-alpha; AltName: Full=PP2A subunit A isoform R1-alpha >gi|47522870|ref|NP_999189.1| serine/threonine-protein ph</td><td>2.623</td><td>1.146</td><td>1.059</td><td>2.451</td></tr>
<tr><td>gi|311270597|ref|XP_003132922.1|</td><td>PREDICTED: 26S proteasome non-ATPase regulatory subunit 9-like [Sus scrofa]</td><td>0.599</td><td>1.217</td><td>1.425</td><td>0.755</td></tr>
<tr><td>gi|47169449|pdb|1VJD|A</td><td>Chain A, Structure Of Pig Muscle Pgk Complexed With Atp >gi|47169448|pdb|1VJC|A Chain A, Structure Of Pig Muscle Pgk Complexed With Mgatp</td><td>1.879</td><td>0.606</td><td>0.764</td><td>1.515</td></tr>
<tr><td>gi|153792027|ref|NP_001093402.1|</td><td>phosphoglycerate kinase 1 [Sus scrofa] >gi|56757507|sp|Q7SIB7.3|PGK1_PIG RecName: Full=Phosphoglycerate kinase 1 >gi|50513041|gb|AAT77773.1| phosphoglycerate kinase 1 [Sus scrofa]</td><td></td><td></td><td></td><td></td></tr>
<tr><td>gi|335281047|ref|XP_003353724.1|</td><td>PREDICTED: LOW QUALITY PROTEIN: long-chain fatty acid transport protein 4 [Sus scrofa]</td><td>0.817</td><td>0.381</td><td>0.401</td><td>0.431</td></tr>
</table>

gi|219521964|ref|NP_001137170.1|	long-chain-fatty-acid--CoA ligase 3 [Sus scrofa] >gi|217314893|gb|ACK36981.1| acyl-CoA synthetase long-chain family member 3 [Sus scrofa]	0.559	0.389	0.335	0.432
gi|47523158|ref|NP_999015.1|	glutathione S-transferase A2 [Sus scrofa] >gi|1185280|emb|CAA93434.1| glutathione S-transferase [Sus scrofa]	2.229	1.726	1.798	3.489
gi|83921643|ref|NP_001033096.1|	serine/arginine-rich splicing factor 1 [Sus scrofa] >gi|114152157|sp|Q3YLA6.3|SRSF1_PIG RecName: Full=Serine/arginine-rich splicing factor 1; AltName: Full=Splicing factor, arginine/serine-rich 1 >gi|73810231|gb|AAZ86089.1| splicing factor arginine/	0.658	0.758	0.713	0.711
gi|311270878|ref|XP_003132994.1|	PREDICTED: AP-1 complex subunit beta-1 isoform 3 [Sus scrofa]	1.174	0.863	0.845	1.105
gi|335301395|ref|XP_003359198.1|	PREDICTED: AP-1 complex subunit beta-1 [Sus scrofa]				
gi|164669|gb|AAA31120.1|	succinyl-CoA synthetase beta-subunit, partial [Sus scrofa]	1.758	0.974	0.824	1.244
gi|90109583|pdb|2FPP|B	Chain B, Crystal Structure Of Pig Gtp-Specific Succinyl-Coa Synthetase From Polyethylene Glycol With Chloride Ions >gi|90109575|pdb|2FPI|B Chain B, Crystal Structure Of Pig Gtp-Specific Succinyl-Coa Synthetase From Polyethylene Glycol >gi|90109573|p				
gi|350591336|ref|XP_003132357.3|	PREDICTED: succinyl-CoA ligase [GDP-forming] subunit beta, mitochondrial isoform 1 [Sus scrofa]				
gi|21264506|sp|P53590.2|SUCB2_PIG	RecName: Full=Succinyl-CoA ligase [GDP-forming] subunit beta, mitochondrial; AltName: Full=GTP-specific succinyl-CoA synthetase subunit beta; AltName: Full=Succinyl-CoA synthetase beta-G chain; Short=SCS-betaG; Flags:				

	Precursor				
gi|9955020|pdb|1EUC|B	Chain B, Crystal Structure Of Dephosphorylated Pig Heart, Gtp-Specific Succinyl-Coa Synthetase				
gi|9955022|pdb|1EUD|B	Chain B, Crystal Structure Of Phosphorylated Pig Heart, Gtp-Specific Succinyl-Coa Synthetase				
gi|120968|sp|P80147.2|GABT_PIG	RecName: Full=4-aminobutyrate aminotransferase, mitochondrial; AltName: Full=(S)-3-amino-2-methylpropionate transaminase; AltName: Full=GABA aminotransferase; Short=GABA-AT; AltName: Full=Gamma-amino-N-butyrate transaminase; Short=GABA transaminase;	1.695	0.783	0.717	1.39
gi|284559|pir||A42481	4-aminobutyrate transaminase (EC 2.6.1.19) precursor - pig				
gi|47523600|ref|NP_999428.1|	4-aminobutyrate aminotransferase, mitochondrial [Sus scrofa] >gi|531154|gb|AAA96981.1| 4-aminobutyrate aminotransferase [Sus scrofa]				
gi|255327|gb|AAB23231.1|	4-aminobutyrate aminotransferase {EC 2.6.1.19} [swine, liver, Peptide, 472 aa] >gi|38492644|pdb|1OHY|C Chain C, 4-Aminobutyrate-Aminotransferase Inactivated By Gamma- Ethynyl Gaba >gi|38492642|pdb|1OHY|A Chain A, 4-Aminobutyrate-Aminotransferase Ina				
gi|346644699|ref|NP_001231019.1|	protein SET [Sus scrofa]	1.404	4.301	2.967	2.469
gi|47522760|ref|NP_999131.1|	long-chain 3-ketoacyl-CoA thiolase [Sus scrofa] >gi|6165556|gb|AAF04612.1|AF028007_1 long-chain 3-ketoacyl-CoA thiolase [Sus scrofa]	0.787	0.43	0.44	0.542
gi|155369760|ref|NP_001094	peroxisomal acyl-coenzyme A	1.021	0.945	1.126	1.452

498.1|	oxidase 1 [Sus scrofa] >gi|112292673|gb|ABI14815.1| palmitoyl acyl-CoA oxidase 1 [Sus scrofa]				
gi|350595285|ref|XP_003484077.1|	PREDICTED: N-alpha-acetyltransferase 38, NatC auxiliary subunit-like [Sus scrofa] >gi|311275562|ref|XP_003134801.1| PREDICTED: N-alpha-acetyltransferase 38, NatC auxiliary subunit-like [Sus scrofa]	2.056	2.91	2.97	2.266
gi|335299041|ref|XP_001926664.3|	PREDICTED: cytochrome b-c1 complex subunit 1, mitochondrial [Sus scrofa]	0.764	0.572	0.812	0.72
gi|61744020|gb|AAX55638.1|	fatty acid synthase [Sus scrofa]	1.431	0.884	0.92	1.488
gi|198443141|pdb|2VZ8|A	Chain A, Crystal Structure Of Mammalian Fatty Acid Synthase >gi|198443142|pdb|2VZ8|B Chain B, Crystal Structure Of Mammalian Fatty Acid Synthase >gi|198443144|pdb|2VZ9|B Chain B, Crystal Structure Of Mammalian Fatty Acid Synthase In Complex With Nad	---	---	---	---
gi|153792600|ref|NP_001093400.1|	fatty acid synthase [Sus scrofa] >gi|148733529|gb|ABR09275.1| fatty acid synthase [Sus scrofa]				
gi|335309408|ref|XP_003361627.1|	PREDICTED: myosin-11-like, partial [Sus scrofa]				
gi|350582036|ref|XP_003481179.1|	PREDICTED: U5 small nuclear ribonucleoprotein 200 kDa helicase [Sus scrofa]	0.686	0.793	0.968	0.662
gi|347800709|ref|NP_001231660.1|	scinderin [Sus scrofa]	1.556	1.156	1.037	1.955
gi|335281857|ref|XP_003122760.2|	PREDICTED: catenin delta-1 [Sus scrofa]	0.533	0.586	0.496	0.613
gi|178056532|ref|NP_001116688.1|	Z-DNA-binding protein 1 [Sus scrofa] >gi|147223427|emb|CAN13344.1| Z-DNA binding protein 1 [Sus scrofa]	0.703	0.731	1.318	1.195
gi|215882147|gb|ACJ70709.1|	DNA-dependent activator of IFN-regulatory factor [Sus scrofa]				
gi|350587231|ref|XP_003482	PREDICTED: LOW QUALITY	1.192	0.769	0.69	0.967

373.1|	PROTEIN: cytoplasmic dynein 1 heavy chain 1 [Sus scrofa]				
gi|311251336|ref|XP_003124563.1|	PREDICTED: elongation factor Tu, mitochondrial-like [Sus scrofa]	0.973	0.501	0.508	0.696
gi|47523726|ref|NP_999498.1|	dolichyl-diphosphooligosaccharide--protein glycosyltransferase subunit 1 precursor [Sus scrofa] >gi|75056164|sp|Q9GMB0.1|RPN1_PIG RecName: Full=Dolichyl-diphosphooligosaccharide--protein glycosyltransferase subunit 1; AltName: Full=Dolichyl-diphosph	0.625	0.627	0.547	0.709
gi|350593062|ref|XP_003133228.3|	PREDICTED: xaa-Pro aminopeptidase 1 isoform 2 [Sus scrofa]	1.117	0.832	0.607	1.023
gi|297591969|ref|NP_001172062.1|	ubiquitin-conjugating enzyme E2 L3 [Sus scrofa] >gi|219563058|gb|ACL27888.1| ubiquitin-conjugating enzyme E2L3 [Sus scrofa]	0.729	1.377	1.106	1.111
gi|311265664|ref|XP_003130763.1|	PREDICTED: LOW QUALITY PROTEIN: kinesin-1 heavy chain [Sus scrofa]	0.823	0.71	1.111	0.887
gi|148234672|ref|NP_001090881.1|	porphobilinogen deaminase [Sus scrofa] >gi|112980813|gb|ABI29188.1| hydroxymethylbilane synthase [Sus scrofa]	0.86	2.163	1.615	1.225
gi|350591926|ref|XP_003358845.2|	PREDICTED: golgin subfamily B member 1 [Sus scrofa]	0.766	0.971	0.959	0.915
gi|113205886|ref|NP_001038075.1|	nucleoside diphosphate kinase B [Sus scrofa] >gi|115311824|sp|Q2EN76.1|NDKB_PIG RecName: Full=Nucleoside diphosphate kinase B; Short=NDK B; Short=NDP kinase B; AltName: Full=Histidine protein kinase NDKB >gi|87047646|gb|ABD18456.1| nm23-H2 [Sus scro	0.829	1.823	1.107	0.757
gi|311249165|ref|XP_003123479.1|	PREDICTED: A-kinase anchor protein 8 [Sus scrofa]	---	---	---	---
gi|47522814|ref|NP_999159.1|	dihydrolipoyllysine-residue acetyltransferase component of	1.069	0.753	0.7	0.863

	pyruvate dehydrogenase complex, mitochondrial precursor [Sus scrofa] >gi|14587786|dbj|BAB61720.1| dihydrolipoamide acetyltransferase [Sus scrofa]				
gi|47523086|ref|NP_999309.1|	peroxiredoxin-5, mitochondrial [Sus scrofa] >gi|10305336|gb|AAG13452.2|AF110735_1 peroxiredoxin 5 [Sus scrofa]	1.866	0.912	0.881	1.432
gi|312062797|ref|NP_001185848.1|	proactivator polypeptide precursor [Sus scrofa] >gi|310789269|gb|ADP24688.1| prosaposin variant 2 [Sus scrofa]	0.731	2.233	2.259	1.324
gi|194036975|ref|XP_001927782.1|	PREDICTED: polyadenylate-binding protein 1 [Sus scrofa]	0.878	1.445	1.228	1.255
gi|1332717|gb|AAB36056.1|	acylamino acid-releasing enzyme, AARE {EC 3.4.19.1} [swine, liver, Peptide, 732 aa] >gi|1583737|prf||2121361A acyl-AA releasing enzyme	1.103	0.791	0.977	0.93
gi|47522644|ref|NP_999088.1|	acylamino-acid-releasing enzyme [Sus scrofa] >gi|1351862|sp|P19205.2|ACPH_PIG RecName: Full=Acylamino-acid-releasing enzyme; Short=AARE; AltName: Full=Acyl-peptide hydrolase; Short=APH; AltName: Full=Acylaminoacyl-peptidase >gi|217672|dbj|BAA00411.1				
gi|47523548|ref|NP_999398.1|	glutaredoxin-1 [Sus scrofa] >gi|121442|sp|P12309.2|GLRX1_PIG RecName: Full=Glutaredoxin-1; AltName: Full=Thioltransferase-1; Short=TTase-1 >gi|332368592|gb|AEE61370.1| glutaredoxin [Sus scrofa] >gi|326632057|gb|ADZ99031.1| glutaredoxin [Sus scrofa]	0.25	0.475	0.406	0.527
gi|157831627|pdb|1KTE|A	Chain A, Crystal Structure Of Thioltransferase At 2.2 Angstrom Resolution				
gi|164359|gb|AAA30992.1|	apolipoprotein A-I [Sus scrofa]	1.067	0.411	0.499	0.666

gi|37787313|gb|AAP69607.1|	nicotinate phosphoribosyltransferase-like protein [Sus scrofa]	1.437	0.862	0.804	1.205
gi|311252547|ref|XP_003125148.1|	PREDICTED: T-complex protein 1 subunit delta-like [Sus scrofa]	2.114	0.936	1.142	1.491
gi|298160970|ref|NP_999423.2|	cAMP-dependent protein kinase type II-alpha regulatory subunit [Sus scrofa] >gi|228008282|dbj|BAH57697.1| cAMP-dependent protein kinase regulatory subunit type II alpha [Sus scrofa]	1.181	0.8	0.707	0.754
gi|325652138|ref|NP_001191702.1|	A-kinase anchor protein 1, mitochondrial [Sus scrofa] >gi|321267428|dbj|BAJ72691.1| A kinase anchor protein 1 [Sus scrofa]	0.686	1.18	1.032	1.126
gi|90200404|gb|ABD92704.1|	triosephosphate isomerase 1 [Sus scrofa]	2.706	1.493	1.114	3.048
gi|262263205|dbj|BAI48105.1|	triosephosphate isomerase 1 [Sus scrofa]				
gi|35384836|gb|AAQ84564.1|	electron transfer flavoprotein alpha subunit precursor [Sus scrofa]	2.734	1.242	1.16	2.173
gi|346986361|ref|NP_001231332.1|	electron-transfer-flavoprotein, alpha polypeptide [Sus scrofa]				
gi|385137156|ref|NP_001245232.1|	kinectin 1 (kinesin receptor) [Sus scrofa]	1.035	1.311	1.263	0.932
gi|350597130|ref|XP_003484362.1|	PREDICTED: V-type proton ATPase subunit B, brain isoform-like [Sus scrofa] >gi|350592249|ref|XP_003483427.1| PREDICTED: V-type proton ATPase subunit B, brain isoform [Sus scrofa]	0.718	0.671	0.6	0.752
gi|350591034|ref|XP_003483187.1|	PREDICTED: glycerol-3-phosphate dehydrogenase 1-like protein-like [Sus scrofa]	1.304	0.697	0.704	0.941
gi|353819|prf||1106188C	cytochrome b5	0.588	0.84	0.666	0.833
gi|48976129|ref|NP_001001770.1|	cytochrome b5 [Sus scrofa] >gi|6166051|sp|P00172.3|CYB5_PIG RecName: Full=Cytochrome b5 >gi|2642486|gb|AAC48779.1| cytochrome b5 [Sus scrofa]				
gi|311277231|ref|XP_003135	PREDICTED: trimethyllysine	1.143	5.553	3.922	1.817

559.1|	dioxygenase, mitochondrial-like [Sus scrofa]				
gi|154147698|ref|NP_001093665.1|	eukaryotic initiation factor 4A-II [Sus scrofa] >gi|85792276|gb|ABC84195.1| eukaryotic translation initiation factor 4A isoform 2 [Sus scrofa]	0.841	0.769	0.49	1.06
gi|311270662|ref|XP_003132938.1|	PREDICTED: phosphatidylethanolamine-binding protein 1-like [Sus scrofa]	1.826	3.013	2.036	1.876
gi|178056588|ref|NP_001116645.1|	ras-related protein Rab-1B [Sus scrofa] >gi|122064946|sp|Q06AU7.1|RAB1B_PIG RecName: Full=Ras-related protein Rab-1B >gi|115394758|gb|ABI97173.1| RAB1B [Sus scrofa]	0.951	0.817	1.011	1.055
gi|62511144|sp|Q764M5.1|STAT1_PIG	RecName: Full=Signal transducer and activator of transcription 1 >gi|47523306|ref|NP_998934.1| signal transducer and activator of transcription 1 [Sus scrofa] >gi|40363629|dbj|BAD06318.1| signal transducer and activator of transcription 1 [Sus scrof	1.669	1.216	1.57	1.391
gi|353468887|gb|AER08620.1|	signal transducer and activator of transcription 1 [Sus scrofa]				
gi|353468891|gb|AER08622.1|	signal transducer and activator of transcription 1 transcript variant 2 [Sus scrofa]				
gi|353468889|gb|AER08621.1|	signal transducer and activator of transcription 1 transcript variant 1 [Sus scrofa]				
gi|194044626|ref|XP_001927289.1|	PREDICTED: 14-3-3 protein beta/alpha isoform 1 [Sus scrofa]	2.234	1.272	0.948	1.724
gi|2947100|gb|AAC39170.1|	15-oxoprostaglandin 13-reductase [Sus scrofa]	1.605	1.793	2.825	3.912
gi|47523824|ref|NP_999550.1|	prostaglandin reductase 1 [Sus scrofa] >gi|2498509|sp|Q29073.1|PTGR1_PIG RecName: Full=Prostaglandin reductase 1; Short=PRG-1; AltName: Full=15-oxoprostaglandin 13-reductase; AltName: Full=NADP-dependent leukotriene				

	B4 12-hydroxydehydrogenase >gi|11				
gi|1845|emb|CAA48565.1|	aminoacylase I [Sus scrofa]	1.855	2.955	3.909	2.438
gi|47522690|ref|NP_999061.1|	aminoacylase-1 [Sus scrofa] >gi|584724|sp|P37111.2|ACY1_PIG RecName: Full=Aminoacylase-1; Short=ACY-1; AltName: Full=N-acyl-L-amino-acid amidohydrolase >gi|533335|dbj|BAA02731.1| N-acylamino acid aminohydrolase (Aminoacylase 1) [Sus scrofa] >gi|4586				
gi|350538593|ref|NP_001233247.1|	phosphoglucomutase-1 [Sus scrofa] >gi|321574209|gb|ADW94631.1| phosphoglucomutase 1 [Sus scrofa]	1.17	0.359	0.319	0.381
gi|194036835|ref|XP_001924895.1|	PREDICTED: 4-trimethylaminobutyraldehyde dehydrogenase [Sus scrofa]	1.684	0.809	0.783	1.223
gi|264681460|ref|NP_001161124.1|	acyl carrier protein, mitochondrial [Sus scrofa] >gi|262204908|dbj|BAI48034.1| NADH dehydrogenase (ubiquinone) 1, alpha/beta subcomplex, 1, 8kDa [Sus scrofa]	0.35	0.688	0.513	0.468
gi|311258000|ref|XP_003127386.1|	PREDICTED: AP-2 complex subunit alpha-1 isoform 1 [Sus scrofa]	1.207	0.648	0.815	1.052
gi|311258002|ref|XP_003127387.1|	PREDICTED: AP-2 complex subunit alpha-1 isoform 2 [Sus scrofa]				
gi|311258004|ref|XP_003127388.1|	PREDICTED: AP-2 complex subunit alpha-1 isoform 3 [Sus scrofa]				
gi|311247353|ref|XP_003122594.1|	PREDICTED: N-acetylated alpha-linked acidic dipeptidase-like 1 [Sus scrofa]	1.445	1.262	2.702	0.96
gi|118675|sp|P09623.1|DLDH_PIG	RecName: Full=Dihydrolipoyl dehydrogenase, mitochondrial; AltName: Full=Dihydrolipoamide dehydrogenase; Flags: Precursor >gi|47522940|ref|NP_999227.1| dihydrolipoyl dehydrogenase, mitochondrial precursor [Sus scrofa] >gi|164539|gb|AAA31069.1| lipoam	0.665	0.684	0.543	0.589
gi|122220|sp|P17630.1|TCO1	RecName: Full=Transcobalamin-1;	1.622	1.801	1.275	2.573

_PIG	Short=TC-1; AltName: Full=Cobalophilin; AltName: Full=Haptocorrin; AltName: Full=Protein R; AltName: Full=Transcobalamin I; Short=TC I; Short=TCI; Flags: Precursor >gi|1964|emb|CAA36800.1| unnamed protein product [Sus				
gi|335306899|ref|XP_003360619.1|	PREDICTED: transcobalamin-1 isoform 1 [Sus scrofa]				
gi|47522620|ref|NP_999100.1|	proteasome subunit beta type-8 [Sus scrofa] >gi|4545092|gb|AAD22390.1|AF059493_1 proteasome subunit LMP7 [Sus scrofa]				
gi|335282599|ref|XP_003123290.2|	PREDICTED: interleukin enhancer-binding factor 3 [Sus scrofa]	1.011	1.226	1.104	1.319
gi|335284501|ref|XP_003124603.2|	PREDICTED: cytochrome b-c1 complex subunit 2, mitochondrial-like [Sus scrofa]	0.886	0.408	0.423	0.451
gi|145559663|gb|ABP73657.1|	MHC class I antigen [Sus scrofa]	0.936	0.687	0.802	1.355
gi|350529387|ref|NP_001231927.1|	basic transcription factor 3 [Sus scrofa]	1.132	3.045	1.918	0.87
gi|264681436|ref|NP_001161113.1|	copper transport protein ATOX1 [Sus scrofa] >gi|262036932|dbj|BAI47601.1| antioxidant protein 1 homolog (yeast) [Sus scrofa]	1.28	2.727	3.209	3.307
gi|350596533|ref|XP_003484288.1|	PREDICTED: copper transport protein ATOX1-like, partial [Sus scrofa]				
gi|335294829|ref|XP_003357324.1|	PREDICTED: acetyl-CoA acetyltransferase, mitochondrial [Sus scrofa]	1.112	0.989	0.72	1.428
gi|350594033|ref|XP_003483821.1|	PREDICTED: UDP-glucuronosyltransferase 1-10 isoform 2 [Sus scrofa]	0.806	0.583	0.609	1.105
gi|350581149|ref|XP_003480971.1|	PREDICTED: matrin-3 [Sus scrofa] >gi|350581147|ref|XP_003480970.1| PREDICTED: matrin-3 [Sus scrofa] >gi|335283551|ref|XP_003354341.1| PREDICTED: matrin-3 [Sus scrofa] >gi|311250254|ref|XP_00312	0.329	0.595	0.655	0.656

	4025.1| PREDICTED: matrin-3 isoform 1 [Sus scrofa]				
gi|350582222|ref|XP_003481226.1|	PREDICTED: LOW QUALITY PROTEIN: dynactin subunit 1 [Sus scrofa]	1.234	2.422	2.124	1.903
gi|47523650|ref|NP_999459.1|	tropomodulin-3 [Sus scrofa] >gi|7288863|gb|AAF45300.1|AF237632_1 ubiquitous tropomodulin U-Tmod [Sus scrofa]	1.732	1.748	1.283	1.247
gi|335292272|ref|XP_003356696.1|	PREDICTED: fumarylacetoacetase-like [Sus scrofa]	1.473	3.38	2.688	1.629
gi|335297524|ref|XP_003358060.1|	PREDICTED: 116 kDa U5 small nuclear ribonucleoprotein component [Sus scrofa] >gi|311267074|ref|XP_003131380.1| PREDICTED: 116 kDa U5 small nuclear ribonucleoprotein component isoform 1 [Sus scrofa]	1.432	1.101	1.086	1.3
gi|335297526|ref|XP_003131381.2|	PREDICTED: 116 kDa U5 small nuclear ribonucleoprotein component isoform 2 [Sus scrofa]				
gi|55977740|sp|Q29387.2|EF1G_PIG	RecName: Full=Elongation factor 1-gamma; Short=EF-1-gamma; AltName: Full=eEF-1B gamma >gi|19111049|gb|AAL85414.1|AF480162_1 eukaryotic elongation factor 1 gamma-like protein [Sus scrofa]	1.33	0.435	0.704	1.36
gi|350580027|ref|XP_003480738.1|	PREDICTED: elongation factor 1-gamma [Sus scrofa]				
gi|113196863|gb|ABI31729.1|	low molecular mass protein 7 [Sus scrofa]	1.128	1.616	1.651	1.131
gi|147225186|emb|CAN13318.1|	proteasome (prosome, macropain) subunit, beta type, 8 (large multifunctional protease 7) [Sus scrofa]				
gi|311260174|ref|XP_001924154.2|	PREDICTED: proteasome subunit beta type-8 [Sus scrofa] >gi|147225184|emb|CAN13316.1| proteasome (prosome, macropain) subunit, beta type, 8 (large multifunctional protease 7) [Sus scrofa]				

gi|72535190|ref|NP_001026957.1|	ras-related protein Rab-1A [Sus scrofa] >gi|75056559|sp|Q52NJ2.3|RAB1A_PIG RecName: Full=Ras-related protein Rab-1A >gi|62868642|gb|AAY17509.1| Ras-related protein Rab-1A [Sus scrofa]	0.891	0.85	0.853	0.998
gi|178056596|ref|NP_001116608.1|	U6 snRNA-associated Sm-like protein LSm2 [Sus scrofa] >gi|147225158|emb|CAN13335.1| LSM2 homolog, U6 small nuclear RNA associated (S. cerevisiae) [Sus scrofa]	0.96	2.495	1.884	1.641
gi|311276332|ref|XP_003135175.1|	PREDICTED: 3-hydroxyacyl-CoA dehydrogenase type-2-like isoform 2 [Sus scrofa]	1.185	0.75	0.745	1.037
gi|311276336|ref|XP_003135176.1|	PREDICTED: 3-hydroxyacyl-CoA dehydrogenase type-2-like isoform 3 [Sus scrofa]				
gi|311276334|ref|XP_003135174.1|	PREDICTED: 3-hydroxyacyl-CoA dehydrogenase type-2-like isoform 1 [Sus scrofa]				
gi|346986418|ref|NP_001231359.1|	DnaJ (Hsp40) homolog, subfamily B, member 1 [Sus scrofa]	1.162	1.428	1.22	1.139
gi|47522680|ref|NP_999069.1|	neutral alpha-glucosidase AB precursor [Sus scrofa] >gi|54037147|sp|P79403.1|GANAB_PIG RecName: Full=Neutral alpha-glucosidase AB; AltName: Full=Alpha-glucosidase 2; AltName: Full=Glucosidase II subunit alpha; Flags: Precursor >gi|1890664|gb|AAB4975	0.917	0.548	0.547	0.771
gi|349585085|ref|NP_001231804.1|	RAB11B, member RAS oncogene family [Sus scrofa]	1.031	0.664	0.52	0.848
gi|335296459|ref|XP_003130769.2|	PREDICTED: vimentin-like [Sus scrofa]	0.816	1.537	1.283	1.234
gi|47523888|ref|NP_999583.1|	ADP/ATP translocase 3 [Sus scrofa] >gi|52000613|sp|Q6QRN9.3|ADT3_PIG RecName: Full=ADP/ATP translocase 3; AltName: Full=ADP,ATP carrier protein 3; AltName: Full=Adenine	0.649	0.471	0.325	0.687

	nucleotide translocator 3; Short=ANT 3; AltName: Full=Solute carrier family 25 m				
gi|1827464|dbj|BAA19181.1|	metallothionein isoform [Sus scrofa]	576.898	5099.604	2186.181	949.007
gi|18202369|sp|P79431.1|MT1E_PIG	RecName: Full=Metallothionein-1E; Short=MT-1E; AltName: Full=Metallothionein-IE; Short=MT-IE >gi|1827462|dbj|BAA19180.1| metallothionein isoform [Sus scrofa]				
gi|335281589|ref|XP_003353840.1|	PREDICTED: 1-phosphatidylinositol-4,5-bisphosphate phosphodiesterase beta-3 isoform 2 [Sus scrofa]	1.089	1.046	1.031	1.203
gi|335281591|ref|XP_003122641.2|	PREDICTED: 1-phosphatidylinositol-4,5-bisphosphate phosphodiesterase beta-3 isoform 1 [Sus scrofa]				
gi|311259696|ref|XP_003128216.1|	PREDICTED: LOW QUALITY PROTEIN: desmoplakin-like [Sus scrofa]	1.077	0.805	1.083	1
gi|350596559|ref|XP_003361362.2|	PREDICTED: ectonucleotide pyrophosphatase/phosphodiesterase family member 3-like, partial [Sus scrofa]	0.727	0.735	0.828	0.662
gi|350580256|ref|XP_003480773.1|	PREDICTED: nucleobindin-2 [Sus scrofa]	0.608	1.207	1.564	1.02
gi|350580258|ref|XP_003122998.3|	PREDICTED: nucleobindin-2 isoform 2 [Sus scrofa]				
gi|311261483|ref|XP_003128742.1|	PREDICTED: LOW QUALITY PROTEIN: golgin subfamily A member 5-like [Sus scrofa]	1.092	1.63	1.604	1.104
gi|342187276|ref|NP_001230112.1|	AP-2 complex subunit beta [Sus scrofa]	0.861	0.815	0.909	1.118
gi|47522662|ref|NP_999076.1|	membrane-associated progesterone receptor component 1 [Sus scrofa] >gi|6647580|sp|Q95250.3|PGRC1_PIG RecName: Full=Membrane-associated progesterone receptor component 1 >gi|1657409|emb|CAA68050.1| steroid membrane binding protein	0.562	0.373	0.608	0.603

	[Sus scrofa]				
gi|335286672|ref|XP_003125720.2|	PREDICTED: transgelin-2-like isoform 1 [Sus scrofa] >gi|335286674|ref|XP_003355153.1| PREDICTED: transgelin-2-like isoform 2 [Sus scrofa]	1.501	0.95	0.996	1.143
gi|311259388|ref|XP_003128071.1|	PREDICTED: cytochrome b-c1 complex subunit 6, mitochondrial-like [Sus scrofa]	0.606	0.434	0.581	0.418
gi|194042189|ref|XP_001924213.1|	PREDICTED: annexin A11 [Sus scrofa]	1.218	0.642	0.78	0.835
gi|346421435|ref|NP_001231091.1|	sorbitol dehydrogenase [Sus scrofa]	1.724	1.163	0.884	1.309
gi|544074|sp|Q99028.1|COMT_PIG	RecName: Full=Catechol O-methyltransferase	1.179	0.618	0.878	1.088
gi|305855180|ref|NP_001182259.1|	catechol O-methyltransferase [Sus scrofa] >gi|285818436|gb|ADC38891.1| catechol-O-methyltransferase [Sus scrofa]				
gi|350586831|ref|XP_003482287.1|	PREDICTED: cytochrome c oxidase subunit 5A, mitochondrial-like isoform 1 [Sus scrofa] >gi|350586833|ref|XP_003482288.1| PREDICTED: cytochrome c oxidase subunit 5A, mitochondrial-like isoform 2 [Sus scrofa]	0.26	0.519	0.473	0.375
gi|350579447|ref|XP_003480607.1|	PREDICTED: protein NipSnap homolog 3A [Sus scrofa]	0.815	1.531	1.298	1.206
gi|335280113|ref|XP_003121789.2|	PREDICTED: LOW QUALITY PROTEIN: 60S ribosomal protein L4 [Sus scrofa]	0.772	0.477	0.419	1.1
gi|311249564|ref|XP_003123691.1|	PREDICTED: heterogeneous nuclear ribonucleoprotein H [Sus scrofa]	0.699	0.928	0.779	0.843
gi|343790906|ref|NP_001230510.1|	calcineurin-like phosphoesterase domain containing 1 [Sus scrofa]	0.755	2.436	1.559	1.194
gi|113205878|ref|NP_001038072.1|	delta(3,5)-Delta(2,4)-dienoyl-CoA isomerase, mitochondrial [Sus scrofa] >gi|90024980|gb|AAZ82038.2| peroxisomal enoyl coenzyme A hydratase 1 [Sus scrofa]	1.299	1.408	0.971	1.065

gi|120564445|gb|ABM30148.1|	peroxisomal enoyl coenzyme A hydratase 1 [Sus scrofa]				
gi|311268173|ref|XP_003131919.1|	PREDICTED: myb-binding protein 1A [Sus scrofa]	0.608	2.053	1.081	1.739
gi|47523406|ref|NP_999324.1|	polymeric immunoglobulin receptor precursor [Sus scrofa] >gi|6863080|dbj|BAA84283.2| poly-Ig receptor [Sus scrofa]	0.825	0.854	0.69	1.094
gi|312283580|ref|NP_999292.2|	superoxide dismutase [Mn], mitochondrial [Sus scrofa]	0.97	1.8	0.677	0.974
gi|335296662|ref|XP_003130891.2|	PREDICTED: 6-phosphofructokinase type C [Sus scrofa]	1.462	0.995	1.326	1.464
gi|335281560|ref|XP_003353830.1|	PREDICTED: splicing factor 1 isoform 2 [Sus scrofa]	1.14	1.611	1.992	0.968
gi|335281562|ref|XP_003353831.1|	PREDICTED: splicing factor 1 isoform 3 [Sus scrofa]				
gi|335281564|ref|XP_003122635.2|	PREDICTED: splicing factor 1 isoform 1 [Sus scrofa]				
gi|346986445|ref|NP_001231372.1|	NSFL1 (p97) cofactor (p47) [Sus scrofa]	1.188	1.88	1.872	1.52
gi|154147660|ref|NP_001093666.1|	eukaryotic initiation factor 4A-I [Sus scrofa] >gi|85792232|gb|ABC84193.1| eukaryotic translation initiation factor 4A isoform 1 [Sus scrofa]	1.139	0.781	0.373	1.323
gi|335308568|ref|XP_003361283.1|	PREDICTED: eukaryotic initiation factor 4A-I-like [Sus scrofa]				
gi|148225750|ref|NP_001090973.1|	heat shock protein 105 kDa [Sus scrofa] >gi|141521428|gb|ABO88027.1| heat shock 105kDa/110kDa protein 1 [Sus scrofa]	0.908	1.023	0.959	1.296
gi|311274155|ref|XP_003134215.1|	PREDICTED: NADH dehydrogenase [ubiquinone] iron-sulfur protein 6, mitochondrial-like [Sus scrofa]	0.891	0.494	1.079	0.589
gi|3746946|gb|AAC64131.1|	phosphoglucomutase 1 [Sus scrofa] >gi|3746944|gb|AAC64130.1| phosphoglucomutase 1 [Sus scrofa]	0.855	0.665	0.452	0.453
gi|350578005|ref|XP_001924268.4|	PREDICTED: ezrin [Sus scrofa]	1.43	1.542	1.413	1.084
gi|343790977|ref|NP_001230537.1|	phospholysine phosphohistidine inorganic pyrophosphate phosphatase [Sus scrofa]	2.963	1.89	1.485	1.259
gi|350577999|ref|XP_001928	PREDICTED: ezrin-like [Sus scrofa]	1.848	0.828	1.314	1.341

755.4|					
gi|350580184|ref|XP_003353965.2|	PREDICTED: reticulocalbin-1-like isoform 2 [Sus scrofa]	0.631	0.986	1.263	0.758
gi|311259408|ref|XP_003128090.1|	PREDICTED: peroxiredoxin-1 isoform 5 [Sus scrofa]	2.943	1.704	1.309	3.046
gi|311259416|ref|XP_003128087.1|	PREDICTED: peroxiredoxin-1 isoform 2 [Sus scrofa] >gi|311259412|ref|XP_003128089.1| PREDICTED: peroxiredoxin-1 isoform 4 [Sus scrofa] >gi|311259414|ref|XP_003128088.1| PREDICTED: peroxiredoxin-1 isoform 3 [Sus scrofa] >gi|311259410|ref|XP_003128086.				
gi|1236646|gb|AAC48775.1|	ch4 and secrete domains of swine IgM [Sus scrofa]	0.779	0.684	0.478	0.807
gi|291202695|dbj|BAI82566.1|	IgM heavy chain constant region [Sus scrofa]				
gi|113205854|ref|NP_001038066.1|	40S ribosomal protein S3 [Sus scrofa] >gi|115502828|sp|Q0Z8U2.1|RS3_PIG RecName: Full=40S ribosomal protein S3 >gi|110006645|gb|ABG48767.1| ribosomal protein S3 [Sus scrofa]	0.59	0.43	0.367	0.585
gi|47523792|ref|NP_999532.1|	catenin beta-1 [Sus scrofa] >gi|167834655|gb|ACA03158.1| beta-catenin [Sus scrofa] >gi|18146999|dbj|BAB82984.1| beta-catenin [Sus scrofa]	0.615	0.612	0.533	0.625
gi|335302729|ref|XP_003359534.1|	PREDICTED: catenin beta-1-like [Sus scrofa]				
gi|311275253|ref|XP_003134650.1|	PREDICTED: single-stranded DNA-binding protein, mitochondrial-like isoform 1 [Sus scrofa]	0.973	1.943	1.71	1.215
gi|47523618|ref|NP_999441.1|	citrate synthase, mitochondrial precursor [Sus scrofa] >gi|116470|sp|P00889.2|CISY_PIG RecName: Full=Citrate synthase, mitochondrial; Flags: Precursor >gi|164419|gb|AAA31017.1| citrate synthase precursor (EC	2.016	0.966	1.408	2.124

	4.1.3.7) [Sus scrofa]				
gi|157834775|pdb|2CTS|A	Chain A, Crystallographic Refinement And Atomic Models Of Two Different Forms Of Citrate Synthase At 2.7 And 1.7 Angstroms Resolution				
gi|253723327|pdb|3ENJ|A	Chain A, Structure Of Pig Heart Citrate Synthase At 1.78 A Resolution >gi|157830720|pdb|1CTS|A Chain A, Crystallographic Refinement And Atomic Models Of Two Different Forms Of Citrate Synthase At 2.7 And 1.7 Angstroms Resolution >gi|230994|pdb|4CTS|				
gi|311247022|ref|XP_003122449.1|	PREDICTED: cadherin-related family member 5 [Sus scrofa]	0.425	0.525	0.89	0.481
gi|335310692|ref|XP_003362151.1|	PREDICTED: protein LZIC-like [Sus scrofa]	1.219	2.177	2.93	1.599
gi|55583765|sp|Q863Z0.3|PSME2_PIG	RecName: Full=Proteasome activator complex subunit 2; AltName: Full=Proteasome activator 28 subunit beta; Short=PA28b; Short=PA28beta >gi|47523632|ref|NP_999444.1| proteasome activator complex subunit 2 [Sus scrofa] >gi|34978648|gb|AAQ83575.1| prote	1.436	0.64	1.096	1.422
gi|342349319|ref|NP_001230139.1|	calnexin precursor [Sus scrofa]	0.685	0.549	0.7	0.759
gi|350590415|ref|XP_003131608.3|	PREDICTED: prohibitin-like [Sus scrofa]	1.62	0.765	0.723	1.186
gi|311258421|ref|XP_003127605.1|	PREDICTED: 6-phosphogluconate dehydrogenase, decarboxylating [Sus scrofa]	1.967	1.556	1.349	1.821
gi|71361861|gb|AAZ30034.1|	Mx1 [Sus scrofa]	0.967	1.069	0.893	1.743
gi|156720190|dbj|BAF76736.1|	Mx1 [Sus scrofa]				
gi|343488474|ref|NP_001230453.1|	elongation factor 1-beta [Sus scrofa]	0.927	1.277	2.426	1.258
gi|347300276|ref|NP_001231441.1|	poly(rC) binding protein 2 [Sus scrofa]	1.094	0.854	0.85	1.302
gi|345090969|ref|NP_001230	septin 2 [Sus scrofa]	1.57	1.505	1.305	1.276

<table>
<tr><td>722.1|</td><td></td><td></td><td></td><td></td><td></td></tr>
<tr><td>gi|448581|prf||1917268B</td><td>pyruvate dehydrogenase:SUBUNIT=beta</td><td>1.03</td><td>0.518</td><td>0.699</td><td>0.734</td></tr>
<tr><td>gi|346986351|ref|NP_001231327.1|</td><td>pyruvate dehydrogenase E1 component subunit beta, mitochondrial [Sus scrofa]</td><td></td><td></td><td></td><td></td></tr>
<tr><td>gi|311268663|ref|XP_003132151.1|</td><td>PREDICTED: 3-ketoacyl-CoA thiolase A, peroxisomal isoform 1 [Sus scrofa]</td><td>2.147</td><td>1.584</td><td>1.713</td><td>1.641</td></tr>
<tr><td>gi|349732238|ref|NP_001231864.1|</td><td>transaldolase [Sus scrofa]</td><td>1.195</td><td>2.121</td><td>1.642</td><td>1.788</td></tr>
<tr><td>gi|213021241|ref|NP_001132944.1|</td><td>proteasome subunit alpha type-6 [Sus scrofa] >gi|210062872|gb|ACJ06406.1| proteasome subunit alpha type 6 [Sus scrofa]</td><td>1.055</td><td>1.229</td><td>1.076</td><td>1.231</td></tr>
<tr><td>gi|335296435|ref|XP_003357777.1|</td><td>PREDICTED: cytoplasmic aconitate hydratase [Sus scrofa]</td><td>2.956</td><td>1.061</td><td>1.068</td><td>1.935</td></tr>
<tr><td>gi|350587882|ref|XP_003129304.3|</td><td>PREDICTED: caspase-6-like [Sus scrofa]</td><td>0.925</td><td>2.035</td><td>1.475</td><td>1.488</td></tr>
<tr><td>gi|335296241|ref|XP_003357722.1|</td><td>PREDICTED: ladinin-1-like [Sus scrofa]</td><td>0.587</td><td>0.68</td><td>0.71</td><td>0.87</td></tr>
<tr><td>gi|311254260|ref|XP_001929592.2|</td><td>PREDICTED: interleukin enhancer-binding factor 2 [Sus scrofa]</td><td>1.471</td><td>1.176</td><td>0.872</td><td>1.452</td></tr>
<tr><td>gi|343887442|ref|NP_001230623.1|</td><td>heterogeneous nuclear ribonucleoprotein C (C1/C2) 2 [Sus scrofa]</td><td>0.738</td><td>0.645</td><td>0.614</td><td>0.827</td></tr>
<tr><td>gi|311261988|ref|XP_003128958.1|</td><td>PREDICTED: phosphoglucomutase-2 [Sus scrofa]</td><td>1.677</td><td>1.345</td><td>1.583</td><td>2.313</td></tr>
<tr><td>gi|2833353|sp|Q29058.1|LEG4_PIG</td><td>RecName: Full=Galectin-4; Short=Gal-4; AltName: Full=L-36 lactose-binding protein; Short=L36LBP; AltName: Full=Lactose-binding lectin 4 >gi|47522788|ref|NP_999146.1| galectin-4 [Sus scrofa] >gi|623346|emb|CAA55884.1| L-36 lactose binding protein [Su</td><td>0.835</td><td>1.744</td><td>1.568</td><td>1.613</td></tr>
<tr><td>gi|350594031|ref|XP_003483820.1|</td><td>PREDICTED: UDP-glucuronosyltransferase 1-10 isoform 1 [Sus scrofa]</td><td>0.773</td><td>0.689</td><td>0.694</td><td>0.597</td></tr>
<tr><td>gi|350591042|ref|XP_003132</td><td>PREDICTED: programmed cell death</td><td>1.284</td><td>0.954</td><td>1.22</td><td>1.59</td></tr>
</table>

145.3|	6-interacting protein [Sus scrofa]				
gi|343478174|ref|NP_001230356.1|	T-complex protein 1 subunit alpha [Sus scrofa]	1.601	0.881	1.189	1.637
gi|335282824|ref|XP_003123501.2|	PREDICTED: ilvB (bacterial acetolactate synthase)-like [Sus scrofa]	0.942	0.825	0.694	0.805
gi|345507389|gb|AEO00192.1|	mucin 13A [Sus scrofa]	0.3	0.499	0.463	0.521
gi|345507392|gb|AEO00194.1|	mucin 13A [Sus scrofa] >gi|345507386|gb|AEO00190.1| mucin 13A [Sus scrofa]				
gi|163310773|ref|NP_001090990.2|	membrane-associated progesterone receptor component 2 [Sus scrofa] >gi|160688702|gb|ABX45133.1| progesterone receptor membrane component 2 [Sus scrofa] >gi|160688700|gb|ABX45132.1| progesterone receptor membrane component 2 [Sus scrofa]	0.546	0.683	0.9	0.82
gi|350586183|ref|XP_003128050.3|	PREDICTED: enoyl-CoA hydratase domain-containing protein 2, mitochondrial-like isoform 1 [Sus scrofa]	1.14	0.512	0.439	0.738
gi|281427370|ref|NP_001163993.1|	T-complex protein 1 subunit eta [Sus scrofa] >gi|262036937|dbj|BAI47603.1| chaperonin containing TCP1, subunit 7 (eta) [Sus scrofa]	1.904	0.901	0.911	1.878
gi|194037681|ref|XP_001926390.1|	PREDICTED: early endosome antigen 1 [Sus scrofa]	0.749	1.226	1.334	0.818
gi|349732201|ref|NP_001231854.1|	dpy-30 homolog [Sus scrofa]	0.546	1.038	2.141	2.438
gi|47523892|ref|NP_999584.1|	epithelial cell adhesion molecule precursor [Sus scrofa] >gi|75045149|sp|Q75QW1.1|EPCAM_PIG RecName: Full=Epithelial cell adhesion molecule; Short=Ep-CAM; AltName: Full=Tumor-associated calcium signal transducer 1; AltName: CD_antigen=CD326; Flags:	0.935	0.504	0.553	0.642
gi|311248247|ref|XP_003123039.1|	PREDICTED: ATP synthase subunit delta, mitochondrial-like isoform 1 [Sus scrofa]	0.28	0.385	0.346	0.274

gi|350580769|ref|XP_003480894.1|	PREDICTED: ATP synthase subunit delta, mitochondrial-like [Sus scrofa]				
gi|350591788|ref|XP_003358782.2|	PREDICTED: hypothetical protein LOC448846 [Sus scrofa]	1.439	0.714	0.869	0.874
gi|311250071|ref|XP_003123947.1|	PREDICTED: solute carrier family 12 member 2 [Sus scrofa]	---	---	---	---
gi|132574|sp|P10775.1|RINI_PIG	RecName: Full=Ribonuclease inhibitor; AltName: Full=Ribonuclease/angiogenin inhibitor 1	1.696	1.691	0.941	1.56
gi|313507173|pdb|1DFJ|I	Chain I, Ribonuclease Inhibitor Complexed With Ribonuclease A >gi|253723219|pdb|2BNH|A Chain A, Porcine Ribonuclease Inhibitor				
gi|48675943|ref|NP_001001640.1|	amine oxidase [flavin-containing] A [Sus scrofa] >gi|62899648|sp|Q6Q2J0.1|AOFA_PIG RecName: Full=Amine oxidase [flavin-containing] A; AltName: Full=Monoamine oxidase type A; Short=MAO-A >gi|45551418|gb|AAS68020.1| monoamine oxidase A [Sus scrofa]	0.908	0.565	0.663	1.069
gi|350592964|ref|XP_003483582.1|	PREDICTED: PDZ and LIM domain protein 1 [Sus scrofa]	1.389	2.467	2.281	1.529
gi|62529273|gb|AAX84942.1|	type III calpastatin [Sus scrofa]	0.743	1.941	2.387	1.443
gi|160425376|gb|ABX39530.1|	calpastatin [Sus scrofa]				
gi|160425375|gb|ABX39529.1|	calpastatin [Sus scrofa]				
gi|47522950|ref|NP_999232.1|	calpastatin [Sus scrofa] >gi|44990121|gb|AAS50179.1| calpastatin [Sus scrofa]				
gi|160425379|gb|ABX39533.1|	calpastatin [Sus scrofa]				
gi|124109|sp|P12675.1|ICAL_PIG	RecName: Full=Calpastatin; AltName: Full=Calpain inhibitor >gi|39545760|gb|AAR27961.1| calpastatin [Sus scrofa] >gi|164407|gb|AAA31012.1| heart calpastatin [Sus scrofa]				

gi|160425377|gb|ABX39531.1|	calpastatin [Sus scrofa]				
gi|160425378|gb|ABX39532.1|	calpastatin [Sus scrofa]				
gi|345091067|ref|NP_001230751.1|	aldo-keto reductase family 7, member A2 (aflatoxin aldehyde reductase) [Sus scrofa]	1.684	1.225	1.113	1.685
gi|350590384|ref|XP_003131576.3|	PREDICTED: importin subunit beta-1-like isoform 1 [Sus scrofa]	1.133	0.693	0.653	0.91
gi|148232214|ref|NP_001090964.1|	ribose-5-phosphate isomerase [Sus scrofa] >gi|158512830|sp|A2TLM1.1|RPIA_PIG RecName: Full=Ribose-5-phosphate isomerase; AltName: Full=Phosphoriboisomerase >gi|124265188|gb|ABM98101.1| ribose 5-phosphate isomerase-like protein [Sus scrofa]	0.938	3.291	3.682	1.253
gi|311275469|ref|XP_003134753.1|	PREDICTED: calumenin isoform 2 [Sus scrofa]	0.129	1.092	0.556	0.512
gi|1346670|sp|P04175.2|NCPR_PIG	RecName: Full=NADPH--cytochrome P450 reductase; Short=CPR; Short=P450R	0.722	0.432	0.709	0.752
gi|194018678|ref|NP_001123431.1|	NADPH--cytochrome P450 reductase [Sus scrofa] >gi|499862|gb|AAA85368.1| NADPH-cytochrome P-450 oxidoreductase [Sus scrofa]				
gi|194035831|ref|XP_001928742.1|	PREDICTED: coatomer subunit alpha isoform 2 [Sus scrofa]	1.561	1.003	0.878	1.562
gi|194035835|ref|XP_001928732.1|	PREDICTED: coatomer subunit alpha isoform 1 [Sus scrofa]				
gi|350579323|ref|XP_003122006.3|	PREDICTED: retinal dehydrogenase 1-like, partial [Sus scrofa]	1.35	1.241	1.953	1.515
gi|194044922|ref|XP_001927475.1|	PREDICTED: ADP/ATP translocase 2 [Sus scrofa]	0.806	0.269	0.464	0.69
gi|350579921|ref|XP_003353801.2|	PREDICTED: dipeptidyl peptidase 3-like isoform 1 [Sus scrofa] >gi|335281448|ref|XP_003353806.1| PREDICTED: dipeptidyl peptidase 3-like [Sus scrofa]	1.859	1.16	1.027	2.216
gi|350579923|ref|XP_003480	PREDICTED: dipeptidyl peptidase				

715.1|	3-like isoform 2 [Sus scrofa]				
gi|18699303|gb|AAL78518.1|AF464056_1	MHC class I antigen [Sus scrofa]	---	---	---	---
gi|29027455|gb|AAO62097.1|	MHC class I antigen [Sus scrofa]				
gi|168208432|gb|ACA21801.1|	MHC class I antigen [Sus scrofa]				
gi|347300323|ref|NP_001231460.1|	thioredoxin-dependent peroxide reductase, mitochondrial [Sus scrofa]	0.606	0.676	0.513	0.331
gi|350588020|ref|XP_003357153.2|	PREDICTED: protein transport protein Sec31A [Sus scrofa]	1.508	0.744	0.93	1.055
gi|194595733|gb|ACF77144.1|	clathrin light chain (CLTA) protein [Sus scrofa]	2.514	2.008	3.157	1.538
gi|47523670|ref|NP_999471.1|	peroxisomal multifunctional enzyme type 2 [Sus scrofa] >gi|499340|emb|CAA55037.1| 17beta-estradiol dehydrogenase [Sus scrofa]	1.407	1.131	1.217	1.501
gi|1702997|sp|P54613.1|2AAB_PIG	RecName: Full=Serine/threonine-protein phosphatase 2A 65 kDa regulatory subunit A beta isoform; AltName: Full=PP2A subunit A isoform PR65-beta; AltName: Full=PP2A subunit A isoform R1-beta >gi|510347|emb|CAA84403.1| protein phosphatase 2A 65 kDa reg	---	---	---	---
gi|335294859|ref|XP_003357335.1|	PREDICTED: serine/threonine-protein phosphatase 2A 65 kDa regulatory subunit A beta isoform isoform 3 [Sus scrofa]				
gi|335294855|ref|XP_003357333.1|	PREDICTED: serine/threonine-protein phosphatase 2A 65 kDa regulatory subunit A beta isoform isoform 1 [Sus scrofa]				
gi|335294861|ref|XP_003357336.1|	PREDICTED: serine/threonine-protein phosphatase 2A 65 kDa regulatory subunit A beta isoform isoform 4 [Sus scrofa]				
gi|335294857|ref|XP_003357334.1|	PREDICTED: serine/threonine-protein phosphatase 2A 65 kDa regulatory subunit A beta isoform isoform 2 [Sus scrofa]				

gi|80971504|ref|NP_001032223.1|	40S ribosomal protein SA [Sus scrofa] >gi|122069666|sp|Q4GWZ2.3|RSSA_PIG RecName: Full=40S ribosomal protein SA; AltName: Full=37 kDa laminin receptor precursor; Short=37LRP; AltName: Full=37/67 kDa laminin receptor; Short=LRP/LR; AltName: Full=67 k	2.076	1.036	0.727	1.462
gi|335302965|ref|XP_001925033.2|	PREDICTED: lupus La protein homolog [Sus scrofa]	1.032	0.677	0.571	1.326
gi|319401915|ref|NP_001188313.1|	rho GDP-dissociation inhibitor 1 [Sus scrofa] >gi|315321426|gb|ADU04840.1| Rho GDP dissociation inhibitor alpha [Sus scrofa]	2.584	0.973	1.127	1.81
gi|350580873|ref|XP_003123770.3|	PREDICTED: ras GTPase-activating-like protein IQGAP2-like [Sus scrofa]	0.937	0.761	0.636	0.979
gi|343780950|ref|NP_001230488.1|	39S ribosomal protein L40, mitochondrial isoform 1 [Sus scrofa]	0.573	0.577	0.466	0.63
gi|343780952|ref|NP_001230489.1|	39S ribosomal protein L40, mitochondrial [Sus scrofa]				
gi|350595262|ref|XP_003484071.1|	PREDICTED: calumenin [Sus scrofa]	0.635	0.71	0.802	0.56
gi|305855176|ref|NP_001182260.1|	cleavage and polyadenylation specificity factor subunit 6 [Sus scrofa] >gi|285818464|gb|ADC38905.1| cleavage and polyadenylation specific factor 6 [Sus scrofa]	0.757	1.403	1.44	0.672
gi|311254645|ref|XP_003125912.1|	PREDICTED: EPS8-like 3 [Sus scrofa]	0.87	0.538	0.696	0.824
gi|311273235|ref|XP_003133777.1|	PREDICTED: intestinal-type alkaline phosphatase-like isoform 2 [Sus scrofa]	0.879	1.307	2.392	0.652
gi|311257294|ref|XP_003127050.1|	PREDICTED: cytochrome b-c1 complex subunit Rieske, mitochondrial-like [Sus scrofa]	0.723	0.545	0.722	0.632
gi|335304027|ref|XP_003134070.2|	PREDICTED: 28S ribosomal protein S36, mitochondrial-like [Sus scrofa]	0.469	0.658	0.771	0.704
gi|350587377|ref|XP_003356918.2|	PREDICTED: cytosol aminopeptidase-like [Sus scrofa]	1.687	2.747	3.579	1.011
gi|335284301|ref|XP_003354567.1|	PREDICTED: T-complex protein 1 subunit zeta-like isoform 2 [Sus	2.957	1.74	1.338	2.699

	scrofa]				
gi|335284299|ref|XP_003124509.2|	PREDICTED: T-complex protein 1 subunit zeta-like isoform 1 [Sus scrofa]				
gi|335281222|ref|XP_003353764.1|	PREDICTED: probable 2-ketogluconate reductase-like isoform 2 [Sus scrofa]	2.964	2.138	1.42	2.895
gi|335281224|ref|XP_001924750.3|	PREDICTED: probable 2-ketogluconate reductase-like isoform 1 [Sus scrofa]				
gi|164543|gb|AAA31072.1|	malate dehydrogenase (EC 1.1.1.37), partial [Sus scrofa]	0.973	0.715	0.601	0.988
gi|6226874|sp|P11708.4|MDHC_PIG	RecName: Full=Malate dehydrogenase, cytoplasmic; AltName: Full=Cytosolic malate dehydrogenase >gi|47523114|ref|NP_999039.1| malate dehydrogenase, cytoplasmic [Sus scrofa] >gi|1469402|gb|AAC48610.1| cytosolic malate dehydrogenase [Sus scrofa]				
gi|5107783|pdb|5MDH|A	Chain A, Crystal Structure Of Ternary Complex Of Porcine Cytoplasmic Malate Dehydrogenase Alpha-Ketomalonate And Tnad At 2.4 Angstroms Resolution >gi|5107784|pdb|5MDH|B Chain B, Crystal Structure Of Ternary Complex Of Porcine Cytoplasmic Malate Dehy				
gi|350596594|ref|XP_003484293.1|	PREDICTED: malate dehydrogenase, cytoplasmic-like [Sus scrofa]				
gi|311247012|ref|XP_003122436.1|	PREDICTED: epidermal growth factor receptor kinase substrate 8-like protein 2-like [Sus scrofa]	0.712	1.308	1.067	0.98
gi|83816981|ref|NP_001033050.1|	proteasome subunit beta type-9 [Sus scrofa] >gi|311260176|ref|XP_003128374.1| PREDICTED: proteasome subunit beta type-9-like [Sus scrofa] >gi|110276961|gb|ABG57117.1| proteasome beta 9 subunit [Sus scrofa] >gi|83320492|gb|ABC02872.1| proteasome beta	1.175	1.396	1.46	1.491

gi|147225181|emb|CAN13313.1|	proteasome (prosome, macropain) subunit, beta type, 9 (large multifunctional protease 2) [Sus scrofa]				
gi|116175259|ref|NP_001070686.1|	heterogeneous nuclear ribonucleoprotein A1 [Sus scrofa] >gi|115371763|gb|ABI96206.1| ROA1 [Sus scrofa]	1.325	0.865	0.712	1.539
gi|45479848|gb|AAS66766.1|	histone H1.3-like protein [Sus scrofa]	0.519	1.101	0.872	1.21
gi|72535198|ref|NP_001026962.1|	histone H1.3-like protein [Sus scrofa] >gi|45479846|gb|AAS66765.1| histone H1.3-like protein [Sus scrofa]				
gi|47522712|ref|NP_999108.1|	pyridoxal kinase [Sus scrofa] >gi|67470293|sp|O46560.1|PDXK_PIG RecName: Full=Pyridoxal kinase; AltName: Full=Pyridoxine kinase >gi|2773404|gb|AAB96794.1| pyridoxal kinase [Sus scrofa]	0.708	0.261	0.275	0.245
gi|194036227|ref|XP_001929678.1|	PREDICTED: selenium-binding protein 1 [Sus scrofa]	1.132	1.756	2.005	1.465
gi|350529411|ref|NP_001231937.1|	proteasome (prosome, macropain) 26S subunit, ATPase, 2 [Sus scrofa]	0.795	0.859	1.186	1.024
gi|462384|sp|P33198.1|IDHP_PIG	RecName: Full=Isocitrate dehydrogenase [NADP], mitochondrial; Short=IDH; AltName: Full=ICD-M; AltName: Full=IDP; AltName: Full=NADP(+)-specific ICDH; AltName: Full=Oxalosuccinate decarboxylase; Flags: Precursor >gi|294223|gb|AAA31089.1| NADPH-specif	1.284	0.588	0.64	0.99
gi|27065481|pdb|1LWD|B	Chain B, Crystal Structure Of Nadp-Dependent Isocitrate Dehydrogenase From Porcine Heart Mitochondria >gi|27065480|pdb|1LWD|A Chain A, Crystal Structure Of Nadp-Dependent Isocitrate Dehydrogenase From Porcine Heart Mitochondria				
gi|255683404|ref|NP_001157479.1|	isocitrate dehydrogenase [NADP], mitochondrial [Sus scrofa]				

gi|311268489|ref|XP_003132080.1|	PREDICTED: fatty aldehyde dehydrogenase-like [Sus scrofa]	0.804	0.424	0.401	0.695
gi|346644882|ref|NP_001231113.1|	reticulocalbin 2, EF-hand calcium binding domain precursor [Sus scrofa]	0.68	0.644	0.763	0.748
gi|350593970|ref|XP_001925937.3|	PREDICTED: 26S proteasome non-ATPase regulatory subunit 1 [Sus scrofa]	0.88	1.075	1.463	1.26
gi|297307133|ref|NP_001171997.1|	protein canopy homolog 2 precursor [Sus scrofa]	0.75	1.048	1.338	0.943
gi|349732227|ref|NP_001231859.1|	heterogeneous nuclear ribonucleoprotein M [Sus scrofa]	0.574	0.507	0.477	0.618
gi|350587641|ref|XP_003129114.3|	PREDICTED: UDP-glucuronosyltransferase 2C1-like [Sus scrofa]	0.934	0.43	0.67	0.942
gi|335282758|ref|XP_003123386.2|	PREDICTED: UV excision repair protein RAD23 homolog A-like [Sus scrofa]	0.577	1.518	1.899	0.832
gi|51493747|gb|AAU04861.1|	enoyl-Coenzyme A hydratase/3-hydroxyacyl Coenzyme A [Sus scrofa]	1.749	1.377	1.622	1.487
gi|351738777|gb|AEQ61487.1|	ribokinase [Sus scrofa]	1.083	1.493	1.603	1.019
gi|311260951|ref|XP_003128591.1|	PREDICTED: dehydrogenase/reductase SDR family member 1-like [Sus scrofa]	0.695	0.789	0.682	1.04
gi|194038973|ref|XP_001929258.1|	PREDICTED: purine nucleoside phosphorylase [Sus scrofa]	1.05	0.89	0.952	0.937
gi|350595461|ref|XP_003134939.3|	PREDICTED: LOW QUALITY PROTEIN: 2-oxoglutarate dehydrogenase, mitochondrial-like [Sus scrofa]	0.912	0.602	0.409	0.891
gi|178056556|ref|NP_001116620.1|	probable aminopeptidase NPEPL1 [Sus scrofa] >gi|147223400|emb|CAN13197.1| aminopeptidase-like 1 [Sus scrofa]	1.378	2.15	2.596	0.755
gi|311253799|ref|XP_001926739.2|	PREDICTED: ras-related protein Rab-2A [Sus scrofa]	1.027	0.39	0.427	0.617
gi|350596433|ref|XP_003484271.1|	PREDICTED: PDZ and LIM domain protein 1-like [Sus scrofa]	0.669	0.927	1.06	0.662
gi|335290692|ref|XP_003356	PREDICTED: UDP-glucose	0.766	0.24	0.268	0.393

250.1|	4-epimerase [Sus scrofa]				
gi|350589440|ref|XP_003130687.3|	PREDICTED: Golgi membrane protein 1-like [Sus scrofa]	0.268	0.679	0.831	0.666
gi|343098455|ref|NP_001230202.1|	ras-related protein Rab-6A [Sus scrofa]	1.156	0.719	0.616	0.97
gi|343488534|ref|NP_001230423.1|	homeobox prox 1 [Sus scrofa]	1.246	1.094	1.062	0.853
gi|335285943|ref|XP_003354987.1|	PREDICTED: eukaryotic translation initiation factor 5A-1-like [Sus scrofa] >gi|311268294|ref|XP_003131981.1| PREDICTED: eukaryotic translation initiation factor 5A-1-like isoform 4 [Sus scrofa] >gi|311268290|ref|XP_003131978.1| PREDICTED: eukaryotic	1.099	1.491	1.183	0.938
gi|335283574|ref|XP_003354345.1|	PREDICTED: transcriptional activator protein Pur-alpha isoform 2 [Sus scrofa] >gi|335283572|ref|XP_003354344.1| PREDICTED: transcriptional activator protein Pur-alpha isoform 1 [Sus scrofa]	0.921	1.144	1.289	0.939
gi|350579350|ref|XP_003353611.2|	PREDICTED: stomatin-like protein 2-like [Sus scrofa]	0.571	0.404	0.611	0.539
gi|350592614|ref|XP_003483500.1|	PREDICTED: splicing factor 3A subunit 1 isoform 1 [Sus scrofa]	0.967	1.291	0.815	1.114
gi|350591864|ref|XP_001926353.4|	PREDICTED: LOW QUALITY PROTEIN: melanotransferrin [Sus scrofa]	0.828	0.796	1.07	0.643
gi|311254575|ref|XP_003125894.1|	PREDICTED: rho-related GTP-binding protein RhoC-like [Sus scrofa]	0.832	0.891	0.934	0.864
gi|346986432|ref|NP_001231366.1|	ras homolog gene family, member A [Sus scrofa]				
gi|75053043|sp|Q6UAQ8.3|ETFB_PIG	RecName: Full=Electron transfer flavoprotein subunit beta; Short=Beta-ETF >gi|328550534|ref|NP_001192208.1| electron transfer flavoprotein subunit beta [Sus scrofa] >gi|35384838|gb|AAQ84565.1| electron transfer flavoprotein beta subunit precursor [S	1.237	0.867	1.028	1.205
gi|350580630|ref|XP_003123	PREDICTED: far upstream	0.981	1.596	1.271	1.194

158.3|	element-binding protein 2-like [Sus scrofa]				
gi|343478210|ref|NP_001230371.1|	syntaxin 12 [Sus scrofa]	0.949	1.837	2.884	1.001
gi|350591495|ref|XP_003132454.3|	PREDICTED: coatomer subunit gamma [Sus scrofa]	1.377	0.929	0.721	1.045
gi|350582722|ref|XP_003481338.1|	PREDICTED: 14-3-3 protein theta isoform 1 [Sus scrofa]	1.696	1.369	1.297	1.659
gi|350582724|ref|XP_003481339.1|	PREDICTED: 14-3-3 protein theta isoform 2 [Sus scrofa]				
gi|217039113|gb|ACJ76845.1|	HDL binding protein [Sus scrofa]	0.968	0.866	0.691	0.902
gi|345090979|ref|NP_001230725.1|	NADH dehydrogenase (ubiquinone) Fe-S protein 3, 30kDa (NADH-coenzyme Q reductase) [Sus scrofa]	0.51	0.285	0.248	0.434
gi|335310868|ref|XP_003362230.1|	PREDICTED: T-complex protein 1 subunit epsilon, partial [Sus scrofa]	1.491	0.598	0.472	1.125
gi|334305778|sp|D2SW95.1|COPB_PIG	RecName: Full=Coatomer subunit beta; AltName: Full=Beta-coat protein; Short=Beta-COP >gi|288860138|ref|NP_001165837.1| coatomer subunit beta [Sus scrofa] >gi|268633765|gb|ACZ15983.1| coatomer protein subunit beta 1 [Sus scrofa]	1.192	0.661	0.623	0.916
gi|12231040|sp|Q29048.2|VATA_PIG	RecName: Full=V-type proton ATPase catalytic subunit A; Short=V-ATPase subunit A; AltName: Full=V-ATPase 69 kDa subunit; AltName: Full=Vacuolar proton pump subunit alpha	0.868	0.558	0.642	0.755
gi|51592133|ref|NP_001004042.1|	V-type proton ATPase catalytic subunit A [Sus scrofa] >gi|1968|emb|CAA44213.1| H+ ATPase [Sus scrofa]				
gi|116047849|gb|ABJ53147.1|	glutathione S-transferase mu 2 [Sus scrofa]	2.29	1.598	1.589	3.519
gi|118403788|ref|NP_001072152.1|	glutathione S-transferase mu 2 [Sus scrofa] >gi|116047847|gb|ABJ53146.1| glutathione S-transferase mu 2 [Sus scrofa]				

gi|350584479|ref|XP_003126643.3|	PREDICTED: bcl-2-like protein 13 [Sus scrofa]	0.678	1.003	0.721	0.739
gi|345100481|pdb|1C9P|A	Chain A, Complex Of Bdellastasin With Porcine Trypsin >gi|3318722|pdb|1AN1|E Chain E, Leech-Derived Tryptase InhibitorTRYPSIN COMPLEX	0.612	0.894	0.765	0.701
gi|311259162|ref|XP_003127965.1|	PREDICTED: far upstream element-binding protein 1 [Sus scrofa]	0.59	0.988	0.965	0.846
gi|350586216|ref|XP_003128039.3|	PREDICTED: thioredoxin domain-containing protein 12-like [Sus scrofa]	0.456	1.098	1.272	1.059
gi|197251946|ref|NP_001127815.1|	actin-related protein 3 [Sus scrofa] >gi|195562215|gb|ACG50179.1| actin-related protein 3-like protein [Sus scrofa]	1.498	1.237	1.031	1.763
gi|136192|sp|P09571.2|TRFE_PIG	RecName: Full=Serotransferrin; Short=Transferrin; AltName: Full=Beta-1 metal-binding globulin; AltName: Full=Siderophilin >gi|18655907|pdb|1H76|A Chain A, The Crystal Structure Of Diferric Porcine Serum Transferrin	0.803	0.46	0.442	0.476
gi|350591529|ref|XP_003483289.1|	PREDICTED: serotransferrin isoform 1 [Sus scrofa]				
gi|347582654|ref|NP_001231582.1|	serotransferrin precursor [Sus scrofa] >gi|350591531|ref|XP_003483290.1| PREDICTED: serotransferrin isoform 2 [Sus scrofa] >gi|189232884|emb|CAQ34904.1| transferrin [Sus scrofa]				
gi|833800|emb|CAA30943.1|	transferrin [Sus scrofa]				
gi|335281104|ref|XP_003353734.1|	PREDICTED: argininosuccinate synthase [Sus scrofa]	1.671	2.308	2.587	1.797
gi|47523456|ref|NP_999349.1|	serine/threonine-protein phosphatase PP1-beta catalytic subunit [Sus scrofa] >gi|47116968|sp|P61292.3|PP1B_PIG RecName: Full=Serine/threonine-protein phosphatase PP1-beta catalytic subunit;	1.843	1.251	1.257	1.937

	Short=PP-1B >gi|89257972|gb|ABD65256.1| protein phosphatase				
gi|113205690|ref|NP_001038024.1|	protein phosphatase 1 catalytic subunit alpha isoform [Sus scrofa] >gi|87621715|gb|ABD38708.1| protein phosphatase 1 catalytic subunit alpha isoform [Sus scrofa]				
gi|113205694|ref|NP_001038025.1|	protein phosphatase 1 catalytic subunit gamma isoform [Sus scrofa] >gi|87621736|gb|ABD38709.1| protein phosphatase 1 catalytic subunit gamma isoform [Sus scrofa]				
gi|335300836|ref|XP_003359054.1|	PREDICTED: ES1 protein homolog, mitochondrial-like [Sus scrofa]	0.485	1.208	0.769	1.133
gi|335280672|ref|XP_003353634.1|	PREDICTED: aldehyde dehydrogenase X, mitochondrial [Sus scrofa]	1.944	0.802	0.591	1.353
gi|311258550|ref|XP_003127676.1|	PREDICTED: EF-hand domain-containing protein D2-like [Sus scrofa]	0.429	0.804	0.768	0.614
gi|335300581|ref|XP_003358947.1|	PREDICTED: T-complex protein 1 subunit theta-like isoform 1 [Sus scrofa]	1.545	0.974	0.794	1.863
gi|335300583|ref|XP_003358948.1|	PREDICTED: T-complex protein 1 subunit theta-like isoform 2 [Sus scrofa]				
gi|335300585|ref|XP_003358949.1|	PREDICTED: T-complex protein 1 subunit theta-like isoform 3 [Sus scrofa]				
gi|47523680|ref|NP_999472.1|	10 kDa heat shock protein, mitochondrial [Sus scrofa] >gi|30525868|gb|AAP32465.1| heat shock 10kD protein [Sus scrofa]	0.739	2.1	0.843	0.766
gi|305855170|ref|NP_001182250.1|	long-chain-fatty-acid--CoA ligase 5 [Sus scrofa] >gi|285818418|gb|ADC38882.1| acyl-CoA synthetase long-chain family member 5 [Sus scrofa]	0.82	0.53	0.574	0.782
gi|350581487|ref|XP_003124444.3|	PREDICTED: 14-3-3 protein gamma-like [Sus scrofa]	2.892	1.76	1.578	2.189
gi|350587966|ref|XP_003482525.1|	PREDICTED: PDZ and LIM domain protein 5 isoform 3 [Sus scrofa]	0.779	1.597	1.479	1.14

gi|311257850|ref|XP_003127328.1|	PREDICTED: 17-beta-hydroxysteroid dehydrogenase 14-like [Sus scrofa]	1.285	0.541	0.462	0.492
gi|194043292|ref|XP_001928111.1|	PREDICTED: 14-3-3 protein eta [Sus scrofa]	3.227	1.275	1.172	2.33
gi|164507|gb|AAA51295.1|	immunoglobulin gamma-chain [Sus scrofa]	0.635	0.488	0.566	0.442
gi|122131841|sp|Q06A98.1|SRSF2_PIG	RecName: Full=Serine/arginine-rich splicing factor 2; AltName: Full=Splicing component, 35 kDa; AltName: Full=Splicing factor SC35; Short=SC-35; AltName: Full=Splicing factor, arginine/serine-rich 2 >gi|116175283|ref|NP_001070697.1| serine/arginine-	1.196	1.38	1.786	1.313
gi|1921|emb|CAA33169.1|	unnamed protein product [Sus scrofa]	0.735	0.558	0.573	0.786
gi|114313|sp|P11607.1|AT2A2_PIG	RecName: Full=Sarcoplasmic/endoplasmic reticulum calcium ATPase 2; Short=SERCA2; Short=SR Ca(2+)-ATPase 2; AltName: Full=Calcium pump 2; AltName: Full=Calcium-transporting ATPase sarcoplasmic reticulum type, slow twitch skeletal muscle isoform; AltN				
gi|47522682|ref|NP_999066.1|	propionyl-CoA carboxylase beta chain, mitochondrial precursor [Sus scrofa] >gi|6093657|sp|P79384.1|PCCB_PIG RecName: Full=Propionyl-CoA carboxylase beta chain, mitochondrial; Short=PCCase subunit beta; AltName: Full=Propanoyl-CoA:carbon dioxide liga	0.717	0.681	1.051	0.758
gi|311272700|ref|XP_003133549.1|	PREDICTED: heterogeneous nuclear ribonucleoprotein A3-like isoform 1 [Sus scrofa]	1.347	0.704	0.642	1.203
gi|335303045|ref|XP_003359613.1|	PREDICTED: heterogeneous nuclear ribonucleoprotein A3-like isoform 2 [Sus scrofa]				
gi|335291231|ref|XP_003356440.1|	PREDICTED: desmoglein-2 [Sus scrofa]	0.805	0.477	0.561	0.564

gi|116551|sp|P04404.2|CMGA_PIG	RecName: Full=Chromogranin-A; Short=CgA; Contains: RecName: Full=Pancreastatin; Contains: RecName: Full=Parastatin; Contains: RecName: Full=WE-14; Flags: Precursor >gi|164417|gb|AAA31016.1| chromogranin A precursor, partial [Sus scrofa]	0.486	0.982	0.552	0.599
gi|341823654|ref|NP_001157477.2|	chromogranin-A precursor [Sus scrofa]				
gi|335298834|ref|XP_003358406.1|	PREDICTED: leucine-rich repeat flightless-interacting protein 2 [Sus scrofa]	0.894	1.546	1.044	1.027
gi|50916342|gb|AAT88086.1|	heat shock protein 27kDa, partial [Sus scrofa]	0.746	1.031	1.13	1.143
gi|55926209|ref|NP_001007519.1|	heat shock protein beta-1 [Sus scrofa] >gi|75062102|sp|Q5S1U1.1|HSPB1_PIG RecName: Full=Heat shock protein beta-1; Short=HspB1; AltName: Full=Heat shock 27 kDa protein; Short=HSP 27 >gi|335284212|ref|XP_003354542.1| PREDICTED: heat shock protein bet				
gi|335284210|ref|XP_003354541.1|	PREDICTED: heat shock protein beta-1-like isoform 1 [Sus scrofa]				
gi|311272309|ref|XP_003133393.1|	PREDICTED: ADP/ATP translocase 1-like isoform 2 [Sus scrofa]	---	---	---	---
gi|251823933|ref|NP_001156532.1|	cadherin-1 precursor [Sus scrofa] >gi|209875157|gb|ACI94878.1| E-cadherin [Sus scrofa]	0.515	1.117	1.208	0.636
gi|350579011|ref|XP_003121807.3|	PREDICTED: acidic leucine-rich nuclear phosphoprotein 32 family member A-like [Sus scrofa]	0.519	0.577	0.573	0.629
gi|45479844|gb|AAS66764.1|	histone H1.3-like protein [Sus scrofa]	---	---	---	---
gi|68534962|ref|NP_001020387.1|	histone H1.2-like protein [Sus scrofa] >gi|66775665|gb|AAY56388.1| histone H1.2-like protein [Sus scrofa]				
gi|45268981|gb|AAS55903.1|	60S ribosomal protein L12, partial [Sus scrofa]	0.878	0.609	0.594	0.987
gi|350579668|ref|XP_003480657.1|	PREDICTED: hypothetical protein LOC414416 [Sus scrofa]				

gi|350590640|ref|XP_003131788.3|	PREDICTED: myosin-Id [Sus scrofa]	0.75	0.444	0.53	0.597
gi|264681430|ref|NP_001161110.1|	3-ketoacyl-CoA thiolase, mitochondrial [Sus scrofa] >gi|262036903|dbj|BAI47587.1| acetyl-Coenzyme A acyltransferase 2 [Sus scrofa]	1.383	0.591	0.719	0.56
gi|335297068|ref|XP_001927259.3|	PREDICTED: CLYBL protein [Sus scrofa]	0.888	1.271	1.636	1.036
gi|350593495|ref|XP_003483700.1|	PREDICTED: LOW QUALITY PROTEIN: glycerol-3-phosphate dehydrogenase, mitochondrial [Sus scrofa]	0.709	0.479	0.534	0.518
gi|350588026|ref|XP_003129409.3|	PREDICTED: heterogeneous nuclear ribonucleoprotein D-like, partial [Sus scrofa]	0.889	1.523	0.997	1.46
gi|47523870|ref|NP_999573.1|	peroxiredoxin-6 [Sus scrofa] >gi|75074817|sp|Q9TSX9.3|PRDX6_PIG RecName: Full=Peroxiredoxin-6; AltName: Full=Non-selenium glutathione peroxidase; Short=NSGPx >gi|6689393|emb|CAB65456.1| non-selenium glutathione phospholipid hydroperoxide peroxidase	1.132	2.008	1.577	1.343
gi|190360593|ref|NP_001121945.1|	interferon-induced guanylate-binding protein 1 [Sus scrofa] >gi|166202346|gb|ABY84597.1| guanylate binding protein 1 [Sus scrofa]	0.933	0.844	0.731	1.258
gi|2500494|sp|Q29308.3|RS19_PIG	RecName: Full=40S ribosomal protein S19	0.747	0.585	0.525	0.846
gi|350585266|ref|XP_003127229.3|	PREDICTED: 40S ribosomal protein S19-like isoform 1 [Sus scrofa] >gi|350585268|ref|XP_003481921.1| PREDICTED: 40S ribosomal protein S19-like [Sus scrofa]				
gi|311267406|ref|XP_003131549.1|	PREDICTED: gasdermin-B-like [Sus scrofa]	1.558	1.337	1.747	2.213
gi|47523724|ref|NP_999497.1|	guanine nucleotide-binding protein subunit beta-2-like 1 [Sus scrofa] >gi|54037165|sp|P63246.3|GB	1.197	0.934	1.038	1.506

	LP_PIG RecName: Full=Guanine nucleotide-binding protein subunit beta-2-like 1; AltName: Full=Receptor for activated C kinase; AltName: Full=Receptor of				
gi|187373263|gb|ACD03295.1|	ISG15 ubiquitin-like modifier [Sus scrofa]	1.661	1.719	3.82	1.158
gi|298104082|ref|NP_001177109.1|	dynein light chain roadblock-type 1 [Sus scrofa]	0.847	2.213	3.05	1.09
gi|311266781|ref|XP_003131241.1|	PREDICTED: galactokinase-like [Sus scrofa]	1.426	0.926	0.906	1.083
gi|350585425|ref|XP_003127517.3|	PREDICTED: isochorismatase domain-containing protein 2, mitochondrial-like isoform 1, partial [Sus scrofa]	0.409	1.462	1.207	0.534
gi|346716265|ref|NP_001231271.1|	omega-amidase NIT2 [Sus scrofa]	1.536	1.113	0.832	1.558
gi|311260243|ref|XP_001927987.2|	PREDICTED: protein CutA isoform 1 [Sus scrofa]	0.621	2.449	1.975	0.951
gi|335292054|ref|XP_003356656.1|	PREDICTED: protein CutA [Sus scrofa]				
gi|335292058|ref|XP_003356658.1|	PREDICTED: protein CutA [Sus scrofa]				
gi|335292056|ref|XP_003356657.1|	PREDICTED: protein CutA [Sus scrofa]				
gi|37962872|gb|AAR05785.1|	cytochrome c oxidase subunit 2 [Sus scrofa] >gi|162423271|gb|ABX89412.1| cytochrome c oxidase subunit 2 [Sus scrofa]	0.868	0.423	0.338	0.684
gi|342316093|gb|ABF56563.2|	cytochrome c oxidase subunit II [Sus scrofa]				
gi|347802433|gb|AEP22017.1|	cytochrome c oxidase subunit II (mitochondrion) [Potamochoerus porcus]				
gi|306485877|gb|ABF49552.2|	cytochrome c oxidase subunit II [Sus scrofa]				
gi|312233367|ref|YP_004021578.1|	cytochrome c oxidase subunit II [Sus scrofa taiwanensis] >gi|317054710|ref|YP_002600780.2| cytochrome c oxidase subunit II [Sus scrofa domesticus] >gi|124358980|ref|YP_0				

	01023766.1| cytochrome c oxidase subunit II [Phacochoerus africanus] >gi|5835866				
gi|157056539|gb|ABV02170.1|	cytochrome c oxidase subunit 2 [Sus scrofa]				
gi|145315754|gb|ABP63291.1|	cytochrome c oxidase subunit II [Sus scrofa]				
gi|157056524|gb|ABV02156.1|	cytochrome c oxidase subunit 2 [Sus scrofa]				
gi|45826173|gb|AAS77694.1|	cytochrome c oxidase subunit 2 [Sus scrofa]				
gi|311252952|ref|XP_003125350.1|	PREDICTED: ketohexokinase-like isoform 3 [Sus scrofa]	0.538	0.556	0.633	0.506
gi|311252954|ref|XP_003125348.1|	PREDICTED: ketohexokinase-like isoform 1 [Sus scrofa]	---	---	---	---
gi|350592835|ref|XP_003483550.1|	PREDICTED: protein transport protein Sec24C isoform 2 [Sus scrofa]				
gi|2497487|sp|Q29561.1|KCY_PIG	RecName: Full=UMP-CMP kinase; AltName: Full=Cytidine monophosphate kinase; AltName: Full=Cytidylate kinase; AltName: Full=Deoxycytidylate kinase; AltName: Full=Uridine monophosphate kinase; AltName: Full=Uridine monophosphate/cytidine monophosphate	0.913	1.002	0.698	0.736
gi|350586231|ref|XP_003128059.3|	PREDICTED: UMP-CMP kinase-like [Sus scrofa]				
gi|335309657|ref|XP_003361720.1|	PREDICTED: 14-3-3 protein epsilon-like [Sus scrofa]	1.488	0.899	0.78	1.393
gi|350579445|ref|XP_003480606.1|	PREDICTED: protein NipSnap homolog 3A-like [Sus scrofa]	0.842	1.427	1.801	0.984
gi|640352|pdb|3MDE|A	Chain A, Crystal Structures Of Medium Chain Acyl-Coa Dehydrogenase From Pig Liver Mitochondria With And Without Substrate >gi|640350|pdb|3MDD|A Chain A, Crystal Structures Of Medium Chain Acyl-Coa Dehydrogenase From Pig Liver Mitochondria With And W	1.391	0.684	0.685	0.883

gi|148872486|sp|P41367.3|ACADM_PIG	RecName: Full=Medium-chain specific acyl-CoA dehydrogenase, mitochondrial; Short=MCAD; Flags: Precursor >gi|56791886|gb|AAW30430.1| medium-chain acyl-coA dehydrogenase [Sus scrofa]				
gi|40889737|pdb|1UDY|D	Chain D, Medium-Chain Acyl-Coa Dehydrogenase With 3-Thiaoctanoyl-Coa >gi|40889735|pdb|1UDY|B Chain B, Medium-Chain Acyl-Coa Dehydrogenase With 3-Thiaoctanoyl-Coa >gi|40889736|pdb|1UDY|C Chain C, Medium-Chain Acyl-Coa Dehydrogenase With 3-Thiaoctanoy				
gi|47522898|ref|NP_999204.1|	medium-chain specific acyl-CoA dehydrogenase, mitochondrial precursor [Sus scrofa] >gi|1127798|gb|AAA83759.1| medium-chain acyl-CoA dehydrogenase [Sus scrofa]				
gi|325652098|ref|NP_001191688.1|	nucleoside diphosphate kinase A [Sus scrofa]	0.73	1.65	1.102	0.703
gi|194037373|ref|XP_001927584.1|	PREDICTED: coatomer subunit zeta-1 isoform 1 [Sus scrofa]	1.781	1.371	1.598	1.578
gi|311244862|ref|XP_001925828.2|	PREDICTED: huntingtin-interacting protein K-like [Sus scrofa]	0.776	1.342	1.28	0.902
gi|311277261|ref|XP_003135572.1|	PREDICTED: 55 kDa erythrocyte membrane protein-like isoform 1 [Sus scrofa]	0.801	0.558	0.535	0.811
gi|47523516|ref|NP_999381.1|	inositol monophosphatase 1 [Sus scrofa] >gi|68568737|sp|O77591.1|IMPA1_PIG RecName: Full=Inositol monophosphatase 1; Short=IMP 1; Short=IMPase 1; AltName: Full=Inositol-1(or 4)-monophosphatase 1; AltName: Full=Lithium-sensitive myo-inositol monophos	1.006	2.592	2.528	1.676
gi|194038734|ref|XP_001929226.1|	PREDICTED: protein transport protein Sec23A isoform 2 [Sus scrofa]	1.655	0.875	0.634	1.202
gi|6435805|pdb|3HDH|B	Chain B, Pig Heart Short Chain	0.962	0.465	0.228	0.846

	L-3-Hydroxyacyl Coa Dehydrogenase Revisited: Sequence Analysis And Crystal Structure Determination >gi|6435806|pdb|3HDH|C Chain C, Pig Heart Short Chain L-3-Hydroxyacyl Coa Dehydrogenase Revisited: Sequence Analysis An				
gi|47523722|ref|NP_999496.1|	hydroxyacyl-coenzyme A dehydrogenase, mitochondrial precursor [Sus scrofa] >gi|7404364|sp|P00348.2|HCDH_PIG RecName: Full=Hydroxyacyl-coenzyme A dehydrogenase, mitochondrial; Short=HCDH; AltName: Full=Medium and short-chain L-3-hydroxyacyl-coenzyme				
gi|311275164|ref|XP_003134601.1|	PREDICTED: amiloride-sensitive amine oxidase [copper-containing] [Sus scrofa]	0.807	0.699	0.984	0.858
gi|164557|gb|AAA31078.1|	MHC PD14 transplantation antigen [Sus scrofa]	2.698	1.722	3.598	1.878
gi|164558|gb|AAA31079.1|	MHC PD14a transplantation antigen [Sus scrofa]				
gi|47523822|ref|NP_999549.1|	glutamate carboxypeptidase 2 [Sus scrofa] >gi|20138100|sp|O77564.1|FOLH1_PIG RecName: Full=Glutamate carboxypeptidase 2; AltName: Full=Folate hydrolase 1; AltName: Full=Folylpoly-gamma-glutamate carboxypeptidase; Short=FGCP; AltName: Full=Glutamate	1.112	2.071	2.577	1.21
gi|116175265|ref|NP_001070688.1|	regucalcin [Sus scrofa] >gi|122131846|sp|Q06AA3.1|RGN_PIG RecName: Full=Regucalcin; Short=RC; AltName: Full=Gluconolactonase; Short=GNL; AltName: Full=Senescence marker protein 30; Short=SMP-30 >gi|115371745|gb|ABI96197.1| SMP-30 [Sus scrofa]	1.624	1.934	2.09	1.639
gi|335305927|ref|XP_003360	PREDICTED: regucalcin-like				

333.1|	isoform 2 [Sus scrofa] >gi|335305929|ref|XP_003135107.2| PREDICTED: regucalcin-like isoform 1 [Sus scrofa]				
gi|311248380|ref|XP_003123108.1|	PREDICTED: cyclic AMP-responsive element-binding protein 3-like protein 3-like isoform 1 [Sus scrofa]	1.073	0.511	0.489	0.485
gi|311248382|ref|XP_003123109.1|	PREDICTED: cyclic AMP-responsive element-binding protein 3-like protein 3-like isoform 2 [Sus scrofa]				
gi|335299760|ref|XP_003358667.1|	PREDICTED: neprilysin isoform 2 [Sus scrofa] >gi|311269573|ref|XP_003132549.1| PREDICTED: neprilysin isoform 1 [Sus scrofa]	0.571	0.385	0.373	0.435
gi|311258794|ref|XP_003127785.1|	PREDICTED: sphingomyelin phosphodiesterase, acid-like 3B [Sus scrofa]	0.849	1.649	1.632	0.918
gi|11641132|gb|AAG38247.1|	cyclophilin [Sus scrofa]	---	---	---	---
gi|335282345|ref|XP_003354038.1|	PREDICTED: mitochondrial import inner membrane translocase subunit Tim13-like [Sus scrofa]	1.78	2.187	2.239	1.616
gi|345441801|ref|NP_001230874.1|	TNF receptor-associated protein 1 [Sus scrofa]	1.932	0.894	0.943	1.689
gi|350539097|ref|NP_001233144.1|	ubiquitin/ISG15-conjugating enzyme E2 L6 [Sus scrofa] >gi|332113317|gb|AEE02035.1| ubiquitin-conjugating enzyme E2L 6 [Sus scrofa]	1.621	1.36	2.399	1.698
gi|194036918|ref|XP_001928194.1|	PREDICTED: thiosulfate sulfurtransferase/rhodanese-like domain-containing protein 1-like [Sus scrofa]	0.678	2.719	2.204	1.842
gi|350584866|ref|XP_003355801.2|	PREDICTED: Golgi apparatus protein 1-like isoform 1 [Sus scrofa]	0.976	0.826	1.083	0.867
gi|350584868|ref|XP_003481837.1|	PREDICTED: Golgi apparatus protein 1-like isoform 2 [Sus scrofa]				
gi|194042126|ref|XP_001928377.1|	PREDICTED: eukaryotic translation initiation factor 3 subunit A [Sus scrofa]	0.9	1.082	0.968	1.434

gi|194034199|ref|XP_001928042.1|	PREDICTED: proteasome subunit alpha type-3 isoform 2 [Sus scrofa]	1.08	2.055	1.906	1.477
gi|194034201|ref|XP_001928025.1|	PREDICTED: proteasome subunit alpha type-3 isoform 1 [Sus scrofa]				
gi|21954744|gb|AAM83105.1|AF525487_1	Ran [Sus scrofa]	1.757	0.822	0.618	1.618
gi|194043605|ref|XP_001925468.1|	PREDICTED: GTP-binding nuclear protein Ran [Sus scrofa]				
gi|194038542|ref|XP_001929208.1|	PREDICTED: methylmalonate-semialdehyde dehydrogenase [acylating], mitochondrial [Sus scrofa]	1.593	0.928	0.929	1.458
gi|322510017|sp|Q007T2.2|CDC42_PIG	RecName: Full=Cell division control protein 42 homolog; Flags: Precursor >gi|269994011|dbj|BAI50642.1| Cell division control protein 42 homolog [Sus scrofa]	1.125	0.764	0.921	1.172
gi|346421347|ref|NP_001231022.1|	glucosamine-6-phosphate isomerase 1 [Sus scrofa]	0.958	1.649	1.438	1.064
gi|335310032|ref|XP_003126353.2|	PREDICTED: dynactin subunit 2-like isoform 3 [Sus scrofa]	1.748	1.035	0.915	1.093
gi|350584186|ref|XP_003126370.2|	PREDICTED: dynactin subunit 2-like [Sus scrofa]				
gi|311247963|ref|XP_003122904.1|	PREDICTED: apoptosis inhibitor 5 isoform 1 [Sus scrofa]	0.895	0.634	0.736	0.863
gi|350594828|ref|XP_003134450.3|	PREDICTED: charged multivesicular body protein 4b-like [Sus scrofa]	1.599	0.904	1.138	0.787
gi|85542092|sp|Q2YGT9.3|RL6_PIG	RecName: Full=60S ribosomal protein L6 >gi|113205608|ref|NP_001038007.1| 60S ribosomal protein L6 [Sus scrofa] >gi|56384243|gb|AAV85770.1| ribosomal protein L6 [Sus scrofa]	0.806	0.295	0.432	0.89
gi|335300802|ref|XP_003359038.1|	PREDICTED: 6-phosphofructokinase, liver type-like [Sus scrofa]	1.026	0.859	0.933	1.51
gi|239977642|sp|Q29183.1|TFF3_PIG	RecName: Full=Trefoil factor 3; AltName: Full=Intestinal trefoil factor; Flags: Precursor	0.853	4.328	3.069	1.667
gi|343478295|ref|NP_001230412.1|	trefoil factor 3 (intestinal) precursor [Sus scrofa]				
gi|350592195|ref|XP_003483	PREDICTED: trefoil factor 3-like,				

413.1|	partial [Sus scrofa]				
gi|262072939|dbj|BAI47777.1|	CCHC-type zinc finger, nucleic acid binding protein [Sus scrofa]	1.38	2.221	1.925	2.168
gi|47523464|ref|NP_999353.1|	dolichyl-diphosphooligosaccharide--protein glycosyltransferase 48 kDa subunit precursor [Sus scrofa] >gi|218511750|sp|Q29381.2|OST48_PIG RecName: Full=Dolichyl-diphosphooligosaccharide--protein glycosyltransferase 48 kDa subunit; Short=DDOST 48 kDa	0.753	0.525	1.151	0.715
gi|113205874|ref|NP_001038071.1|	ATP synthase subunit O, mitochondrial precursor [Sus scrofa] >gi|122145941|sp|Q2EN81.1|ATPO_PIG RecName: Full=ATP synthase subunit O, mitochondrial; AltName: Full=Oligomycin sensitivity conferral protein; Short=OSCP; Flags: Precursor >gi|87047636|gb	1.062	0.495	0.536	0.661
gi|311265228|ref|XP_003130565.1|	PREDICTED: LOW QUALITY PROTEIN: bifunctional aminoacyl-tRNA synthetase [Sus scrofa]	0.783	0.898	0.846	1.048
gi|350591601|ref|XP_003132498.3|	PREDICTED: sodium/potassium-transporting ATPase subunit beta-3-like [Sus scrofa]	0.747	0.364	0.396	0.531
gi|335309396|ref|XP_003361623.1|	PREDICTED: adenylate kinase 2, mitochondrial-like isoform 1 [Sus scrofa]	2.203	1.887	1.877	1.706
gi|385862179|ref|NP_001245364.1|	protein diaphanous homolog 1 [Sus scrofa]	0.625	0.552	0.764	0.564
gi|350587405|ref|XP_003482406.1|	PREDICTED: cytosolic beta-glucosidase-like, partial [Sus scrofa]	1.402	0.973	0.831	1.198
gi|350597193|ref|XP_003484383.1|	PREDICTED: LOW QUALITY PROTEIN: peptidyl-prolyl cis-trans isomerase B [Sus scrofa]	0.394	1.723	0.922	0.958
gi|47523762|ref|NP_999517.1|	plasma membrane calcium-transporting ATPase 1 [Sus scrofa] >gi|114319|sp|P23220.1|AT2B1_PIG RecName: Full=Plasma	0.673	0.627	0.775	0.857

	membrane calcium-transporting ATPase 1; Short=PMCA1; AltName: Full=Plasma membrane calcium pump isoform 1 >gi|2061|emb|CAA37536.1| plasm				
gi|194038540|ref|XP_001929175.1|	PREDICTED: ectonucleoside triphosphate diphosphohydrolase 5 [Sus scrofa]	0.769	0.948	0.509	1.118
gi|350579385|ref|XP_001925894.4|	PREDICTED: glyoxylate reductase/hydroxypyruvate reductase [Sus scrofa]	0.895	0.493	0.623	0.64
gi|343780941|ref|NP_001230485.1|	prohibitin 2 [Sus scrofa]	1.815	0.803	0.787	1.352
gi|311275457|ref|XP_003134747.1|	PREDICTED: filamin-C isoform 1 [Sus scrofa]	---	---	---	---
gi|311275463|ref|XP_003134750.1|	PREDICTED: filamin-C isoform 4 [Sus scrofa]				
gi|335305357|ref|XP_003360191.1|	PREDICTED: filamin-C [Sus scrofa]				
gi|311275461|ref|XP_003134749.1|	PREDICTED: filamin-C isoform 3 [Sus scrofa]				
gi|311275459|ref|XP_003134748.1|	PREDICTED: filamin-C isoform 2 [Sus scrofa]				
gi|164505|gb|AAA51294.1|	immunoglobulin gamma-chain, partial [Sus scrofa]	0.237	0.099	0.217	0.183
gi|433128|gb|AAA52219.1|	Ig gamma 1a chain constant region [Sus scrofa] >gi|2136515|pir||I47161 Ig gamma 3 chain constant region - pig (fragment)				
gi|47523192|ref|NP_998993.1|	IgG heavy chain precursor [Sus scrofa] >gi|5052050|gb|AAD38418.1|AF062384_1 IgG heavy chain precursor [Sus scrofa]				
gi|159138024|gb|ABW89018.1|	immunoglobulin heavy chain gamma constant region [Sus scrofa]				
gi|433122|gb|AAA52216.1|	Ig gamma 1b chain constant region [Sus scrofa] >gi|2136512|pir||I47158 Ig gamma 1 chain constant region - pig (fragment)				
gi|227130|prf||1614425A	chymodenin	0.452	0.325	0.286	0.294
gi|311262177|ref|XP_003129051.1|	PREDICTED: electron transfer flavoprotein-ubiquinone	0.626	0.679	0.626	0.498

<table>
<tr><td></td><td>oxidoreductase, mitochondrial-like [Sus scrofa]</td><td></td><td></td><td></td><td></td></tr>
<tr><td>gi|197251934|ref|NP_001127826.1|</td><td>actin-related protein 2 [Sus scrofa] >gi|195562213|gb|ACG50178.1| actin-related protein 2-like protein [Sus scrofa]</td><td>1.397</td><td>0.5</td><td>0.574</td><td>1.417</td></tr>
<tr><td>gi|78172717|gb|ABB29449.1|</td><td>transporter associated with antigen processing 1 [Sus scrofa]</td><td>0.541</td><td>0.382</td><td>0.447</td><td>0.711</td></tr>
<tr><td>gi|350586535|ref|XP_003128377.3|</td><td>PREDICTED: antigen peptide transporter 1 isoform 1 [Sus scrofa]</td><td></td><td></td><td></td><td></td></tr>
<tr><td>gi|147225183|emb|CAN13315.1|</td><td>transporter 1, ATP-binding cassette, sub-family B (MDR/TAP) [Sus scrofa]</td><td></td><td></td><td></td><td></td></tr>
<tr><td>gi|113205778|ref|NP_001038046.1|</td><td>antigen peptide transporter 1 [Sus scrofa] >gi|78172715|gb|ABB29448.1| transporter associated with antigen processing 1 [Sus scrofa]</td><td></td><td></td><td></td><td></td></tr>
<tr><td>gi|311252239|ref|XP_003124992.1|</td><td>PREDICTED: all-trans-retinol 13,14-reductase [Sus scrofa]</td><td>0.52</td><td>0.2</td><td>0.307</td><td>0.231</td></tr>
<tr><td>gi|335293373|ref|XP_003356947.1|</td><td>PREDICTED: UDP-glucose 6-dehydrogenase-like isoform 1 [Sus scrofa]</td><td>1.734</td><td>1.612</td><td>1.136</td><td>1.981</td></tr>
<tr><td>gi|350580651|ref|XP_003480867.1|</td><td>PREDICTED: scaffold attachment factor B1-like isoform 2 [Sus scrofa]</td><td>0.687</td><td>1.076</td><td>0.762</td><td>0.763</td></tr>
<tr><td>gi|350580653|ref|XP_003354065.2|</td><td>PREDICTED: scaffold attachment factor B1-like isoform 1 [Sus scrofa]</td><td></td><td></td><td></td><td></td></tr>
<tr><td>gi|194041736|ref|XP_001927852.1|</td><td>PREDICTED: fucose mutarotase isoform 1 [Sus scrofa]</td><td>2.396</td><td>1.735</td><td>1.948</td><td>1.845</td></tr>
<tr><td>gi|311272085|ref|XP_003133293.1|</td><td>PREDICTED: fucose mutarotase isoform 2 [Sus scrofa]</td><td></td><td></td><td></td><td></td></tr>
<tr><td>gi|194038586|ref|XP_001926746.1|</td><td>PREDICTED: transmembrane emp24 domain-containing protein 10 [Sus scrofa]</td><td>0.582</td><td>0.399</td><td>0.461</td><td>0.628</td></tr>
<tr><td>gi|72535208|ref|NP_001026966.1|</td><td>2~-5~-oligoadenylate synthase 2 [Sus scrofa] >gi|45594389|gb|AAQ22351.1| 2~-5~ oligoadenylate synthetase 2 [Sus scrofa]</td><td>1.025</td><td>0.661</td><td>0.815</td><td>0.993</td></tr>
<tr><td>gi|85687558|sp|Q4A3R3.1|DMBT1_PIG</td><td>RecName: Full=Deleted in malignant brain tumors 1 protein; AltName: Full=Hensin; Flags: Precursor >gi|73531021|emb|CAJ27171.1| DMBT1 protein [Sus scrofa]</td><td>0.397</td><td>0.523</td><td>0.555</td><td>0.462</td></tr>
</table>

gi|114703738|ref|NP_001041653.1|	deleted in malignant brain tumors 1 protein precursor [Sus scrofa] >gi|110431268|emb|CAJ14977.1| deleted in malignant brain tumors 1 protein [Sus scrofa]				
gi|311246094|ref|XP_001924927.2|	PREDICTED: acidic leucine-rich nuclear phosphoprotein 32 family member B [Sus scrofa]	0.936	0.598	0.775	1.029
gi|298104076|ref|NP_001177104.1|	enoyl-CoA hydratase, mitochondrial [Sus scrofa]	1.176	0.473	0.43	0.891
gi|350596379|ref|XP_003484266.1|	PREDICTED: vesicle-trafficking protein SEC22b-like [Sus scrofa] >gi|350583458|ref|XP_003481524.1| PREDICTED: vesicle-trafficking protein SEC22b [Sus scrofa]	0.749	0.447	0.474	0.538
gi|350582129|ref|XP_003354780.2|	PREDICTED: ig kappa chain V-II region RPMI 6410-like [Sus scrofa]	0.919	1.388	1.364	0.923
gi|350592539|ref|XP_003483482.1|	PREDICTED: translational activator GCN1, partial [Sus scrofa]	---	---	---	---
gi|17432930|sp|Q9N2I1.1|CASP1_PIG	RecName: Full=Caspase-1; Short=CASP-1; AltName: Full=Interleukin-1 beta convertase; Short=IL-1BC; AltName: Full=Interleukin-1 beta-converting enzyme; Short=ICE; Short=IL-1 beta-converting enzyme; AltName: Full=p45; Contains: RecName: Full=Caspase-1	2.442	1.382	1.594	1.486
gi|340007402|ref|NP_001229989.1|	RAB10, member RAS oncogene family [Sus scrofa]	0.916	0.388	0.456	0.797
gi|212549623|ref|NP_001131101.1|	endoplasmic reticulum resident protein 44 precursor [Sus scrofa] >gi|187480160|gb|ACD13000.1| thioredoxin domain-containing 4 [Sus scrofa]	1.194	0.716	0.968	1.059
gi|229620|prf||770227A	dehydrogenase H4,lactate	2.789	2.491	1.866	3.254
gi|56792866|gb|AAW30628.1|	unknown [Sus scrofa]				
gi|282403837|pdb|5LDH|A	Chain A, Structure Of The Active Ternary Complex Of Pig Heart Lactate Dehydrogenase With S-Lac-Nad At 2.7 Angstroms				

<table>
<tr><td></td><td>Resolution >gi|282403838|pdb|5LDH|B Chain B, Structure Of The Active Ternary Complex Of Pig Heart Lactate Dehydrogenase With S-Lac-Na</td><td></td><td></td><td></td><td></td></tr>
<tr><td>gi|56792857|gb|AAW30625.1|</td><td>unknown, partial [Sus scrofa] >gi|56792872|gb|AAW30630.1| unknown [Sus scrofa]</td><td></td><td></td><td></td><td></td></tr>
<tr><td>gi|164518958|ref|NP_001106758.1|</td><td>L-lactate dehydrogenase B chain [Sus scrofa] >gi|1170738|sp|P00336.3|LDHB_PIG RecName: Full=L-lactate dehydrogenase B chain; Short=LDH-B; AltName: Full=LDH heart subunit; Short=LDH-H >gi|473575|gb|AAA50438.1| lactate dehydrogenase-B [Sus scrofa dome</td><td></td><td></td><td></td><td></td></tr>
<tr><td>gi|194044035|ref|XP_001928829.1|</td><td>PREDICTED: splicing factor 3B subunit 1 isoform 1 [Sus scrofa]</td><td>0.537</td><td>0.939</td><td>0.99</td><td>1.072</td></tr>
<tr><td>gi|350582773|ref|XP_003481353.1|</td><td>PREDICTED: 40S ribosomal protein S7-like isoform 2 [Sus scrofa] >gi|350582771|ref|XP_003481352.1| PREDICTED: 40S ribosomal protein S7-like isoform 1 [Sus scrofa]</td><td>0.624</td><td>0.404</td><td>0.255</td><td>0.765</td></tr>
<tr><td>gi|335309199|ref|XP_003361534.1|</td><td>PREDICTED: AP-1 complex subunit sigma-1A-like [Sus scrofa]</td><td>0.824</td><td>0.539</td><td>0.476</td><td>0.893</td></tr>
<tr><td>gi|298160923|ref|NP_001177141.1|</td><td>DDRGK domain-containing protein 1 [Sus scrofa]</td><td>0.358</td><td>0.749</td><td>0.594</td><td>0.461</td></tr>
<tr><td>gi|2498311|sp|Q28943.1|DPYD_PIG</td><td>RecName: Full=Dihydropyrimidine dehydrogenase [NADP(+)]; Short=DHPDHase; Short=DPD; AltName: Full=Dihydrothymine dehydrogenase; AltName: Full=Dihydrouracil dehydrogenase; Flags: Precursor >gi|47522904|ref|NP_999209.1| dihydropyrimidine dehydrogenase</td><td>1.144</td><td>1.171</td><td>1.04</td><td>1.431</td></tr>
<tr><td>gi|20663678|pdb|1GTH|D</td><td>Chain D, Dihydropyrimidine Dehydrogenase (Dpd) From Pig, Ternary Complex With Nadph And 5-Iodouracil >gi|20663676|pdb|1GTH|B Chain B, Dihydropyrimidine</td><td></td><td></td><td></td><td></td></tr>
</table>

	Dehydrogenase (Dpd) From Pig, Ternary Complex With Nadph And 5-Iodouracil >gi|20663644|pdb|1GTE|D				
gi|13399653|pdb|1H7X|C	Chain C, Dihydropyrimidine Dehydrogenase (Dpd) From Pig, Ternary Complex Of A Mutant Enzyme (C671a), Nadph And 5-Fluorouracil >gi|13399651|pdb|1H7X|A Chain A, Dihydropyrimidine Dehydrogenase (Dpd) From Pig, Ternary Complex Of A Mutant Enzyme (C671a				
gi|350591497|ref|XP_003483284.1|	PREDICTED: ras-related protein Rab-7a-like [Sus scrofa]	0.731	0.453	0.527	0.729
gi|190360663|ref|NP_001121949.1|	FYVE and coiled-coil domain-containing protein 1 [Sus scrofa] >gi|41688293|dbj|BAD08647.1| FYVE and coiled-coil domain containing 1 [Sus scrofa]	0.991	0.625	1.214	0.925
gi|194042806|ref|XP_001927837.1|	PREDICTED: annexin A7 isoform 1 [Sus scrofa]	0.478	1.169	1.679	1.453
gi|335301795|ref|XP_003359285.1|	PREDICTED: annexin A7 [Sus scrofa]				
gi|194038359|ref|XP_001929028.1|	PREDICTED: glutaredoxin-related protein 5, mitochondrial-like [Sus scrofa]	1.169	0.703	0.919	0.677
gi|148235351|ref|NP_001090944.1|	NADH dehydrogenase ubiquinone flavoprotein 2 [Sus scrofa] >gi|117660947|gb|ABK55646.1| NDUFV2 [Sus scrofa]	0.501	0.531	0.567	0.591
gi|335287837|ref|XP_001926233.3|	PREDICTED: prefoldin subunit 5-like isoform 1 [Sus scrofa]	1.066	2.467	2.792	1.805
gi|343790920|ref|NP_001230517.1|	fumarate hydratase [Sus scrofa]	1.509	0.966	0.89	1.303
gi|297747368|ref|NP_001172101.1|	ubiquinol-cytochrome c reductase binding protein [Sus scrofa]	0.352	0.887	1.053	0.506
gi|350591701|ref|XP_003132571.3|	PREDICTED: Golgi integral membrane protein 4 [Sus scrofa]	---	---	---	---
gi|350583385|ref|XP_001927905.3|	PREDICTED: tuftelin, partial [Sus scrofa]	0.927	0.974	1.298	0.546
gi|350589959|ref|XP_003482	PREDICTED: LIM domain only	0.679	1.532	1.067	0.786

<table>
<tr><td>961.1|</td><td>protein 7-like [Sus scrofa]</td><td></td><td></td><td></td><td></td></tr>
<tr><td>gi|350585977|ref|XP_003127887.3|</td><td>PREDICTED: erythrocyte membrane protein band 4.1-like 3 [Sus scrofa]</td><td>0.659</td><td>0.795</td><td>0.953</td><td>0.92</td></tr>
<tr><td>gi|335281676|ref|XP_003353867.1|</td><td>PREDICTED: heterogeneous nuclear ribonucleoprotein U-like 2 [Sus scrofa]</td><td>1.432</td><td>0.889</td><td>1.03</td><td>1.367</td></tr>
<tr><td>gi|350578507|ref|XP_003121503.3|</td><td>PREDICTED: asparaginyl-tRNA synthetase, cytoplasmic-like [Sus scrofa]</td><td>1.923</td><td>1.266</td><td>1.484</td><td>1.92</td></tr>
<tr><td>gi|335281552|ref|XP_003122601.2|</td><td>PREDICTED: EH domain-containing protein 1 [Sus scrofa]</td><td>1.348</td><td>2.254</td><td>2.244</td><td>1.547</td></tr>
<tr><td>gi|312062805|ref|NP_001185852.1|</td><td>chloride intracellular channel protein 5 [Sus scrofa] >gi|197361201|gb|ACH70136.1| chloride intracellular channel 5 [Sus scrofa]</td><td>2.034</td><td>0.871</td><td>1</td><td>1.572</td></tr>
<tr><td>gi|346716348|ref|NP_001231189.1|</td><td>arginine--tRNA ligase, cytoplasmic [Sus scrofa]</td><td>1.61</td><td>0.986</td><td>0.841</td><td>1.429</td></tr>
<tr><td>gi|335309827|ref|XP_003361787.1|</td><td>PREDICTED: nuclear autoantigenic sperm protein, partial [Sus scrofa]</td><td>1.435</td><td>2.468</td><td>1.368</td><td>2.088</td></tr>
<tr><td>gi|335288150|ref|XP_003126347.2|</td><td>PREDICTED: prolow-density lipoprotein receptor-related protein 1-like [Sus scrofa]</td><td>0.776</td><td>0.558</td><td>0.53</td><td>0.707</td></tr>
<tr><td>gi|335294978|ref|XP_003357366.1|</td><td>PREDICTED: coatomer subunit delta isoform 1 [Sus scrofa]</td><td>0.933</td><td>0.878</td><td>0.796</td><td>1.047</td></tr>
<tr><td>gi|47523744|ref|NP_999507.1|</td><td>serine/threonine-protein kinase OSR1 [Sus scrofa] >gi|73621342|sp|Q863I2.1|OXSR1_PIG RecName: Full=Serine/threonine-protein kinase OSR1; AltName: Full=Oxidative stress-responsive 1 protein >gi|30525874|gb|AAP32466.1| oxidative-stress responsive 1 [S</td><td>0.723</td><td>0.654</td><td>0.991</td><td>0.473</td></tr>
<tr><td>gi|113205586|ref|NP_001038000.1|</td><td>26S proteasome non-ATPase regulatory subunit 4 [Sus scrofa] >gi|55983054|gb|AAV69969.1| proteasome 26S subunit non-ATPase 4 [Sus scrofa]</td><td>0.572</td><td>0.843</td><td>1.19</td><td>0.746</td></tr>
<tr><td>gi|158514030|sp|A1XQU3.1|RL14_PIG</td><td>RecName: Full=60S ribosomal protein L14 >gi|148237282|ref|NP_001090947.1| 60S ribosomal protein L14 [Sus</td><td>1.034</td><td>0.626</td><td>0.479</td><td>0.855</td></tr>
</table>

	scrofa] >gi|117661006|gb|ABK55649.1| RPL14 [Sus scrofa]				
gi|350586418|ref|XP_001929155.3|	PREDICTED: GMP reductase 1 [Sus scrofa]	---	---	---	---
gi|350587731|ref|XP_003357047.2|	PREDICTED: LPS-responsive vesicle trafficking, beach and anchor containing, partial [Sus scrofa]	0.695	0.477	0.325	0.352
gi|350595424|ref|XP_003484107.1|	PREDICTED: chromobox protein homolog 3-like isoform 2 [Sus scrofa] >gi|350595422|ref|XP_003360239.2| PREDICTED: chromobox protein homolog 3-like isoform 1 [Sus scrofa]	1.138	2.126	2.251	1.606
gi|311255888|ref|XP_003126410.1|	PREDICTED: cullin-associated NEDD8-dissociated protein 1-like [Sus scrofa]	1.43	0.839	0.627	0.959
gi|350584746|ref|XP_003481817.1|	PREDICTED: cullin-associated NEDD8-dissociated protein 1 [Sus scrofa]				
gi|330688410|ref|NP_001193464.1|	actin related protein 2/3 complex, subunit 4 [Sus scrofa] >gi|195562233|gb|ACG50184.1| actin related protein 2/3 complex subunit 4 [Sus scrofa]	1.832	0.682	0.707	1.312
gi|350581241|ref|XP_003124117.3|	PREDICTED: leucyl-tRNA synthetase, cytoplasmic [Sus scrofa]	46.169	0.722	1.086	1.314
gi|335310669|ref|XP_003362140.1|	PREDICTED: hypothetical protein LOC100622909 [Sus scrofa]	0.605	0.902	0.685	0.684
gi|350590115|ref|XP_003131204.3|	PREDICTED: RING finger protein 213-like, partial [Sus scrofa]	1.242	0.866	0.787	1.607
gi|89573899|gb|ABD77175.1|	ribosomal protein L18 [Sus scrofa]	0.92	0.907	0.779	1.498
gi|335289913|ref|XP_003356019.1|	PREDICTED: 60S ribosomal protein L18 isoform 2 [Sus scrofa] >gi|335289911|ref|XP_003356018.1| PREDICTED: 60S ribosomal protein L18 isoform 1 [Sus scrofa]				
gi|335289915|ref|XP_003356020.1|	PREDICTED: 60S ribosomal protein L18 isoform 3 [Sus scrofa]				
gi|22770997|gb|AAN06824.1|	thioredoxin reductase [Sus scrofa]	2.086	1.864	1.576	2.294
gi|255918208|ref|NP_999319.2|	thioredoxin reductase 1, cytoplasmic [Sus scrofa]				
gi|194036302|ref|XP_001926	PREDICTED: histone H2A type	0.779	1.057	1.179	0.893

704.1|	2-B-like [Sus scrofa]				
gi|194039746|ref|XP_001925399.1|	PREDICTED: histone H2A type 1-A-like [Sus scrofa]				
gi|335294990|ref|XP_003129998.2|	PREDICTED: histone H2A.x-like [Sus scrofa]				
gi|350595677|ref|XP_003360362.2|	PREDICTED: GRIP1-associated protein 1 [Sus scrofa]	0.784	1.938	1.963	0.87
gi|311258910|ref|XP_003127843.1|	PREDICTED: splicing factor 3A subunit 3 isoform 1 [Sus scrofa]	0.817	0.532	0.761	1.035
gi|335291009|ref|XP_003356364.1|	PREDICTED: splicing factor 3A subunit 3 isoform 2 [Sus scrofa]				
gi|350594057|ref|XP_003359743.2|	PREDICTED: leucine-rich repeat flightless-interacting protein 2-like [Sus scrofa]	0.725	0.781	1.048	0.688
gi|350596308|ref|XP_003361027.2|	PREDICTED: leucine-rich repeat flightless-interacting protein 2-like [Sus scrofa]				
gi|58801555|ref|NP_001011727.1|	adenosylhomocysteinase [Sus scrofa] >gi|61216107|sp|Q710C4.3|SAHH_PIG RecName: Full=Adenosylhomocysteinase; Short=AdoHcyase; AltName: Full=S-adenosyl-L-homocysteine hydrolase >gi|40644231|emb|CAD20603.1| S-adenosylhomocysteine hydrolase [Sus scrofa]	1.846	1.077	1.022	1.112
gi|262036923|dbj|BAI47597.1|	adenosylhomocysteinase [Sus scrofa] >gi|67513973|dbj|BAD99576.1| S-adenosylhomocysteine hydrolase [Sus scrofa]				
gi|347300360|ref|NP_001231474.1|	serine/arginine repetitive matrix protein 2 [Sus scrofa]	0.351	0.966	0.517	0.477
gi|335279750|ref|XP_003353424.1|	PREDICTED: normal mucosa of esophagus-specific gene 1 protein-like isoform 2 [Sus scrofa] >gi|194034801|ref|XP_001925712.1| PREDICTED: normal mucosa of esophagus-specific gene 1 protein-like isoform 1 [Sus scrofa]	0.512	0.43	0.425	0.466
gi|335282347|ref|XP_003354039.1|	PREDICTED: lamin-B2 [Sus scrofa]	0.704	0.775	0.682	0.731
gi|75054313|sp|Q8WNW3.1|	RecName: Full=Junction	1.027	1.124	0.855	1.047

PLAK_PIG	plakoglobin >gi|47523712|ref|NP_999488.1| junction plakoglobin [Sus scrofa] >gi|218103017|emb|CAT82339.1| unnamed protein product [Sus scrofa] >gi|211637711|emb|CAR97094.1| unnamed protein product [Sus scrofa] >gi|158065996|em				
gi|335294095|ref|XP_003357133.1|	PREDICTED: phosphatidylinositol N-acetylglucosaminyltransferase subunit Y-like isoform 2 [Sus scrofa]	0.198	1.244	0.789	0.539
gi|346644830|ref|NP_001231167.1|	sulfide:quinone oxidoreductase, mitochondrial [Sus scrofa]	0.889	0.519	0.467	0.803
gi|350578685|ref|XP_003480424.1|	PREDICTED: sulfide:quinone oxidoreductase, mitochondrial-like [Sus scrofa]				
gi|311275515|ref|XP_003134775.1|	PREDICTED: NADH dehydrogenase [ubiquinone] 1 alpha subcomplex subunit 5-like [Sus scrofa]	0.491	0.657	0.595	0.425
gi|91208342|sp|Q4FAT7.1|BGLR_PIG	RecName: Full=Beta-glucuronidase; Flags: Precursor >gi|178056516|ref|NP_001116593.1| beta-glucuronidase precursor [Sus scrofa] >gi|69146955|gb|AAZ03639.1| beta-glucuronidase [Sus scrofa]	1.038	1.395	1.756	1.38
gi|147899784|ref|NP_001090887.1|	elongation factor 1-alpha 1 [Sus scrofa] >gi|223019599|emb|CAX36487.1| eukaryotic translation elongation factor 1 alpha 1 [Sus scrofa] >gi|223019597|emb|CAX36486.1| eukaryotic translation elongation factor 1 alpha 1 [Sus scrofa] >gi|110287842|gb|ABG	0.764	0.57	0.438	0.68
gi|350582040|ref|XP_003481180.1|	PREDICTED: U5 small nuclear ribonucleoprotein 200 kDa helicase-like [Sus scrofa]	1.299	0.968	0.982	0.974
gi|311255145|ref|XP_001926338.2|	PREDICTED: thiosulfate sulfurtransferase [Sus scrofa]	1.947	0.819	1.357	1.494
gi|122143720|sp|Q19QT7.1|CGL_PIG	RecName: Full=Cystathionine gamma-lyase; AltName: Full=Cysteine-protein sulfhydrase; AltName: Full=Gamma-cystathionase >gi|1132	1.277	1.871	0.771	1.408

<table>
<tr><td></td><td>05776|ref|NP_001038050.1| cystathionine gamma-lyase [Sus scrofa] >gi|104295139|gb|ABF72039.1| cystathionase [Sus scrofa]</td><td></td><td></td><td></td><td></td></tr>
<tr><td>gi|335307937|ref|XP_003361036.1|</td><td>PREDICTED: golgin subfamily A member 2, partial [Sus scrofa]</td><td>0.688</td><td>0.535</td><td>1.047</td><td>0.64</td></tr>
<tr><td>gi|335281280|ref|XP_003122383.2|</td><td>PREDICTED: apical endosomal glycoprotein [Sus scrofa]</td><td>0.502</td><td>0.431</td><td>0.468</td><td>0.419</td></tr>
<tr><td>gi|350535040|ref|NP_001233182.1|</td><td>eukaryotic translation initiation factor 4 gamma 1 [Sus scrofa] >gi|338784423|gb|AEI98874.1| eukaryotic translation initiation factor 4 gamma 1 [Sus scrofa]</td><td>0.75</td><td>1.018</td><td>0.881</td><td>1.059</td></tr>
<tr><td>gi|335285985|ref|XP_003125470.2|</td><td>PREDICTED: butyrophilin subfamily 1 member A1-like [Sus scrofa]</td><td>0.952</td><td>0.469</td><td>0.543</td><td>0.886</td></tr>
<tr><td>gi|346716222|ref|NP_001231254.1|</td><td>histidine triad nucleotide-binding protein 2, mitochondrial isoform 1 precursor [Sus scrofa]</td><td>2.84</td><td>1.204</td><td>1.571</td><td>1.916</td></tr>
<tr><td>gi|350586921|ref|XP_003482304.1|</td><td>PREDICTED: LOW QUALITY PROTEIN: apoptotic chromatin condensation inducer in the nucleus-like [Sus scrofa]</td><td>0.842</td><td>1.424</td><td>0.894</td><td>1.102</td></tr>
<tr><td>gi|54036319|sp|Q6QAP7.3|RS17_PIG</td><td>RecName: Full=40S ribosomal protein S17 >gi|48675931|ref|NP_001001634.1| 40S ribosomal protein S17 [Sus scrofa] >gi|45269037|gb|AAS55931.1| 40S ribosomal protein S17 [Sus scrofa]</td><td>0.7</td><td>0.663</td><td>0.859</td><td>0.552</td></tr>
<tr><td>gi|389618965|gb|AFK92990.1|</td><td>U2 small nuclear RNA auxiliary factor 2 [Sus scrofa]</td><td>1.303</td><td>1.058</td><td>1.085</td><td>1.315</td></tr>
<tr><td>gi|166796061|ref|NP_001107756.1|</td><td>60S ribosomal protein L5 [Sus scrofa] >gi|164598076|gb|ABY61325.1| ribosomal protein L5 [Sus scrofa]</td><td>1.091</td><td>1.397</td><td>1.374</td><td>1.602</td></tr>
<tr><td>gi|350584106|ref|XP_003481667.1|</td><td>PREDICTED: SWI/SNF complex subunit SMARCC2 [Sus scrofa]</td><td>0.755</td><td>0.898</td><td>0.717</td><td>0.863</td></tr>
<tr><td>gi|350584110|ref|XP_003481668.1|</td><td>PREDICTED: SWI/SNF complex subunit SMARCC2 [Sus scrofa]</td><td></td><td></td><td></td><td></td></tr>
<tr><td>gi|350584134|ref|XP_003355513.2|</td><td>PREDICTED: SWI/SNF complex subunit SMARCC2-like [Sus scrofa]</td><td></td><td></td><td></td><td></td></tr>
<tr><td>gi|350584108|ref|XP_001929203.4|</td><td>PREDICTED: SWI/SNF complex subunit SMARCC2 isoform 2 [Sus</td><td></td><td></td><td></td><td></td></tr>
</table>

	scrofa]				
gi|350582612|ref|XP_003481312.1|	PREDICTED: protein phosphatase 1G-like [Sus scrofa]	1.641	2.347	2.165	1.949
gi|335287568|ref|XP_003126098.2|	PREDICTED: probable ATP-dependent RNA helicase DDX17-like [Sus scrofa]	1.312	0.99	0.748	1.883
gi|350539515|ref|NP_001233132.1|	ATP-dependent RNA helicase DDX3X [Sus scrofa] >gi|332113313|gb|AEE02033.1| X-linked DEAD (Asp-Glu-Ala-Asp) box polypeptide 3 [Sus scrofa]	1.197	0.674	1.417	1.242
gi|47522800|ref|NP_999152.1|	60S ribosomal protein L22 [Sus scrofa] >gi|54039123|sp|P67985.2|RL22_PIG RecName: Full=60S ribosomal protein L22; AltName: Full=Heparin-binding protein HBp15 >gi|409074|dbj|BAA04547.1| heparin binding protein [Sus scrofa]	0.815	0.549	0.479	0.997
gi|122114359|gb|AAY42145.2|	cathepsin D [Sus scrofa]	1.729	1.191	1.125	1.992
gi|311271975|ref|XP_001926332.2|	PREDICTED: short/branched chain specific acyl-CoA dehydrogenase, mitochondrial [Sus scrofa]	1.307	0.714	0.94	1.408
gi|297632426|ref|NP_001172096.1|	signal sequence receptor, alpha [Sus scrofa]	0.756	0.387	0.391	0.624
gi|335293128|ref|XP_003128866.2|	PREDICTED: alpha-adducin [Sus scrofa]	1.111	2.272	1.557	1.839
gi|343790870|ref|NP_001230573.1|	adaptor-related protein complex 2, mu 1 subunit [Sus scrofa]	---	---	---	---
gi|70986480|gb|AAZ16514.1|	hepatic nuclear factor 1 [Sus scrofa]	0.768	1.128	1.78	0.882
gi|239836872|gb|ACS29489.1|	HNF1A, partial [Sus scrofa]				
gi|74024901|ref|NP_001027560.1|	hepatocyte nuclear factor 1-alpha [Sus scrofa] >gi|67866955|gb|AAY82454.1| hepatic nuclear factor 1 [Sus scrofa]				
gi|350592969|ref|XP_003483583.1|	PREDICTED: phosphoglycerate mutase 1-like [Sus scrofa]	1.569	0.987	1.19	1.621
gi|350587465|ref|XP_003128987.3|	PREDICTED: RNA-binding protein 47-like isoform 1 [Sus scrofa]	0.543	0.435	0.259	0.65

gi|350587467|ref|XP_003482419.1|	PREDICTED: RNA-binding protein 47-like [Sus scrofa]				
gi|335302082|ref|XP_001926734.3|	PREDICTED: pyridine nucleotide-disulfide oxidoreductase domain-containing protein 2-like, partial [Sus scrofa]	0.426	0.287	0.465	0.295
gi|146741316|dbj|BAF62313.1|	adaptor-related protein complex 3, delta-1 subunit [Sus scrofa]				
gi|335282242|ref|XP_003354004.1|	PREDICTED: AT-rich interactive domain-containing protein 3A [Sus scrofa]	1.469	1.101	1.576	1.164
gi|345110571|ref|NP_001230773.1|	coproporphyrinogen oxidase [Sus scrofa]	1.109	1.074	1.359	1.315
gi|350529438|ref|NP_001231909.1|	prefoldin subunit 3 [Sus scrofa]	0.853	1.619	1.339	1.117
gi|347582591|ref|NP_001231560.1|	cytoskeleton-associated protein 4 [Sus scrofa]	0.93	1.457	1.466	0.995
gi|350583879|ref|XP_003481608.1|	PREDICTED: LOW QUALITY PROTEIN: cytoskeleton-associated protein 4 [Sus scrofa]				
gi|311247240|ref|XP_003122553.1|	PREDICTED: splicing factor 3B subunit 2 [Sus scrofa]	0.716	1.012	0.897	0.818
gi|347543782|ref|NP_001231554.1|	glutathione synthetase [Sus scrofa]	1.749	1.676	2.554	1.735
gi|335293186|ref|XP_003356895.1|	PREDICTED: grpE protein homolog 1, mitochondrial-like isoform 1 [Sus scrofa]	0.674	0.743	0.608	0.791
gi|350587324|ref|XP_003482387.1|	PREDICTED: grpE protein homolog 1, mitochondrial-like isoform 2 [Sus scrofa]				
gi|194039403|ref|XP_001929632.1|	PREDICTED: CDC5 cell division cycle 5-like [Sus scrofa]	0.541	0.668	0.72	0.847
gi|311247879|ref|XP_003122857.1|	PREDICTED: 26S protease regulatory subunit 6A [Sus scrofa]	1.082	0.958	0.856	1.058
gi|72535182|ref|NP_001026953.1|	ras-related protein Rab-14 [Sus scrofa] >gi|75060237|sp|Q52NJ6.3|RAB14_PIG RecName: Full=Ras-related protein Rab-14 >gi|62868634|gb|AAY17505.1| Ras-related protein Rab-14 [Sus scrofa]	1.13	0.664	0.654	1.221
gi|346421343|ref|NP_001231	ribosomal protein S10 [Sus scrofa]	0.558	0.991	0.938	0.682

035.1|					
gi|311245734|ref|XP_003121939.1|	PREDICTED: hypothetical protein LOC100038023 [Sus scrofa] >gi|311245732|ref|XP_003121938.1| PREDICTED: hypothetical protein LOC100038023 [Sus scrofa]	0.464	0.315	0.345	0.559
gi|311275226|ref|XP_003134622.1|	PREDICTED: glutathione S-transferase kappa 1-like isoform 2 [Sus scrofa]	0.864	0.619	0.577	1.072
gi|311275228|ref|XP_003134621.1|	PREDICTED: glutathione S-transferase kappa 1-like isoform 1 [Sus scrofa]				
gi|47523672|ref|NP_999468.1|	2~-5~-oligoadenylate synthase 1 [Sus scrofa] >gi|6226834|sp|Q29599.3|OAS1_PIG RecName: Full=2~-5~-oligoadenylate synthase 1; Short=(2-5~)oligo(A) synthase 1; Short=2-5A synthase 1; AltName: Full=p42 OAS >gi|3451331|emb|CAA12397.1| 2~-5~ oligoadenyla	0.885	0.462	0.342	0.831
gi|39654572|pdb|1PX5|A	Chain A, Crystal Structure Of The 2~-Specific And Double-Stranded Rna-Activated Interferon-Induced Antiviral Protein 2~-5~-Oligoadenylate Synthetase >gi|39654573|pdb|1PX5|B Chain B, Crystal Structure Of The 2~-Specific And Double-Stranded Rna-Activ				
gi|311247106|ref|XP_003122482.1|	PREDICTED: NADH dehydrogenase [ubiquinone] iron-sulfur protein 8, mitochondrial-like isoform 1 [Sus scrofa]	0.729	0.793	0.549	0.59
gi|311247108|ref|XP_003122483.1|	PREDICTED: NADH dehydrogenase [ubiquinone] iron-sulfur protein 8, mitochondrial-like isoform 2 [Sus scrofa]				
gi|335310373|ref|XP_003362002.1|	PREDICTED: hypothetical protein LOC100628225, partial [Sus scrofa]	0.441	0.79	0.879	0.607
gi|311252247|ref|XP_003124998.1|	PREDICTED: macrophage-capping protein-like isoform 4 [Sus scrofa] >gi|311252243|ref|XP_00312	1.498	2.4	4.476	1.818

<table>
<tr><td></td><td>4996.1| PREDICTED: macrophage-capping protein-like isoform 2 [Sus scrofa] >gi|311252249|ref|XP_003124997.1| PREDICTED: macrophage-capping protein-like isofor</td><td></td><td></td><td></td><td></td></tr>
<tr><td>gi|311254554|ref|XP_003125887.1|</td><td>PREDICTED: bcl-2-like protein 15-like [Sus scrofa]</td><td>0.924</td><td>0.644</td><td>0.797</td><td>0.933</td></tr>
<tr><td>gi|342349346|ref|NP_001230149.1|</td><td>lon peptidase 1, mitochondrial [Sus scrofa]</td><td>0.825</td><td>0.398</td><td>0.342</td><td>0.849</td></tr>
<tr><td>gi|350529356|ref|NP_001231917.1|</td><td>NADH dehydrogenase (ubiquinone) Fe-S protein 5, 15kDa (NADH-coenzyme Q reductase) [Sus scrofa]</td><td>0.217</td><td>0.436</td><td>0.519</td><td>0.262</td></tr>
<tr><td>gi|347582660|ref|NP_001231584.1|</td><td>mesencephalic astrocyte-derived neurotrophic factor precursor [Sus scrofa] >gi|350591263|ref|XP_003483237.1| PREDICTED: mesencephalic astrocyte-derived neurotrophic factor [Sus scrofa] >gi|306563669|gb|ADN03186.1| mesencephalic astrocyte-derived neu</td><td>0.557</td><td>0.812</td><td>0.705</td><td>0.639</td></tr>
<tr><td>gi|311252948|ref|XP_003125347.1|</td><td>PREDICTED: cell growth regulator with EF hand domain protein 1-like isoform 2 [Sus scrofa]</td><td>0.317</td><td>0.344</td><td>0.421</td><td>0.354</td></tr>
<tr><td>gi|311252950|ref|XP_003125346.1|</td><td>PREDICTED: cell growth regulator with EF hand domain protein 1-like isoform 1 [Sus scrofa]</td><td></td><td></td><td></td><td></td></tr>
<tr><td>gi|212549619|ref|NP_001131099.1|</td><td>ERO1-like protein alpha precursor [Sus scrofa] >gi|226741411|sp|B6CVD7.1|ERO1A_PIG RecName: Full=ERO1-like protein alpha; Short=ERO1-L; Short=ERO1-L-alpha; AltName: Full=Endoplasmic oxidoreductin-1-like protein; AltName: Full=Oxidoreductin-1-L-alpha</td><td>1.314</td><td>1.946</td><td>1.577</td><td>1.857</td></tr>
<tr><td>gi|335288894|ref|XP_003126772.2|</td><td>PREDICTED: ubiquitin-conjugating enzyme E2 N-like [Sus scrofa]</td><td>1.78</td><td>2.226</td><td>1.835</td><td>1.902</td></tr>
</table>

gi|350587106|ref|XP_003128729.2|	PREDICTED: protein sel-1 homolog 1-like [Sus scrofa]	---	---	---	---
gi|260593713|ref|NP_001157119.1|	occludin [Sus scrofa] >gi|239934655|emb|CAZ64276.1| occludin [Sus scrofa domesticus]	1.191	---	---	1.157
gi|329744588|ref|NP_001193269.1|	p30 DBC protein [Sus scrofa]	1.715	1.761	0.955	1.182
gi|311245701|ref|XP_003121927.1|	PREDICTED: S-methyl-5~-thioadenosine phosphorylase-like [Sus scrofa]	2.559	2.314	1.457	1.57
gi|84028320|sp|Q9GKX6.1|GALM_PIG	RecName: Full=Aldose 1-epimerase; AltName: Full=Galactose mutarotase >gi|47523866|ref|NP_999571.1| aldose 1-epimerase [Sus scrofa] >gi|11611545|dbj|BAB18973.1| aldose 1-epimerase [Sus scrofa]	1.501	0.864	0.931	1.695
gi|350584800|ref|XP_003355778.2|	PREDICTED: cytochrome c oxidase subunit 4 isoform 1, mitochondrial-like [Sus scrofa]	0.5	0.278	0.305	0.408
gi|335278800|ref|XP_003121142.2|	PREDICTED: afadin [Sus scrofa]	0.568	1.225	1.062	0.765
gi|167908791|ref|NP_001108148.1|	EPLIN-b [Sus scrofa] >gi|86450155|gb|ABC96266.1| EPLIN-b [Sus scrofa]	0.656	0.97	0.799	0.801
gi|335291962|ref|XP_001928015.3|	PREDICTED: E3 ubiquitin-protein ligase TRIM31 [Sus scrofa] >gi|211926941|dbj|BAG82682.1| tripartite motif-containing protein 31 [Sus scrofa]	0.99	0.644	1.319	1.179
gi|350584162|ref|XP_003481682.1|	PREDICTED: serine hydroxymethyltransferase, mitochondrial-like isoform 2 [Sus scrofa]	1.205	0.748	0.778	0.894
gi|350584170|ref|XP_003355531.2|	PREDICTED: serine hydroxymethyltransferase, mitochondrial-like [Sus scrofa] >gi|311255743|ref|XP_003126345.1| PREDICTED: serine hydroxymethyltransferase, mitochondrial-like isoform 1 [Sus scrofa]				
gi|346716298|ref|NP_001231025.1|	heterogeneous nuclear ribonucleoprotein G [Sus scrofa]	0.958	1.657	1.159	1.09

gi|335287882|ref|XP_003126232.2|	PREDICTED: LOW QUALITY PROTEIN: sterol O-acyltransferase 2-like [Sus scrofa]	1.336	0.918	1.402	0.942
gi|335282871|ref|XP_003123529.2|	PREDICTED: epidermal growth factor receptor pathway substrate 15-like 1 [Sus scrofa]	1.048	1.619	1.354	1.24
gi|343478283|ref|NP_001230406.1|	proteasome (prosome, macropain) 26S subunit, non-ATPase, 2 [Sus scrofa]	0.871	1.141	0.897	1.024
gi|311277195|ref|XP_003135533.1|	PREDICTED: host cell factor 1 [Sus scrofa]	0.794	0.801	1.1	0.752
gi|75059260|sp|Q29041.1|FCN2_PIG	RecName: Full=Ficolin-2; AltName: Full=Collagen/fibrinogen domain-containing protein 2; AltName: Full=Ficolin-B; AltName: Full=Ficolin-beta; AltName: Full=L-ficolin; Flags: Precursor >gi|47523126|ref|NP_999033.1| ficolin-2 precursor [Sus scrofa] >gi	0.555	0.353	0.303	0.509
gi|350584750|ref|XP_003126831.3|	PREDICTED: cysteine-rich with EGF-like domain protein 2-like isoform 1 [Sus scrofa]	0.813	1.265	1.285	1.047
gi|350584752|ref|XP_003481818.1|	PREDICTED: cysteine-rich with EGF-like domain protein 2-like [Sus scrofa]				
gi|346986456|ref|NP_001231378.1|	nucleoside diphosphate kinase 3 isoform 1 precursor [Sus scrofa]	0.489	0.953	0.592	0.467
gi|346986458|ref|NP_001231379.1|	nucleoside diphosphate kinase 3 isoform 2 precursor [Sus scrofa]				
gi|238018148|ref|NP_001153899.1|	ADP-ribosylation factor 1 [Sus scrofa] >gi|212725657|gb|ACJ38117.1| ADP-ribosylation factor 1 [Sus scrofa] >gi|237638742|gb|ACR07973.1| ADP-ribosylation factor 1 [Sus scrofa]	0.779	0.435	0.407	0.868
gi|335280985|ref|XP_003122213.2|	PREDICTED: golgin subfamily A member 1 [Sus scrofa]	1.837	1.582	2.493	1.315
gi|350594464|ref|XP_003483904.1|	PREDICTED: LOW QUALITY PROTEIN: clathrin interactor 1 [Sus scrofa]	1.167	0.847	1.137	1.276
gi|2493048|sp|Q95312.1|ATPD_PIG	RecName: Full=ATP synthase subunit delta, mitochondrial;	0.313	0.434	0.45	0.302

	AltName: Full=F-ATPase delta subunit; Flags: Precursor				
gi|356461003|ref|NP_001238977.1|	RNA-binding protein EWS [Sus scrofa] >gi|351738726|gb|AEQ61463.1| EWS [Sus scrofa]	1	1.531	1.38	1.45
gi|350585922|ref|XP_003127872.3|	PREDICTED: adenylyl cyclase-associated protein 1, partial [Sus scrofa]	3.624	2.043	1.696	4.525
gi|350585924|ref|XP_003356378.2|	PREDICTED: adenylyl cyclase-associated protein 1-like [Sus scrofa]				
gi|311249270|ref|XP_003123549.1|	PREDICTED: coiled-coil domain-containing protein 124-like [Sus scrofa]	---	---	---	---
gi|346421386|ref|NP_001231066.1|	ATP synthase subunit g, mitochondrial [Sus scrofa]	0.905	0.362	0.382	0.946
gi|356614808|gb|AET25525.1|	mitochondrial NADH dehydrogenase Fe-S protein 4 [Sus scrofa]	0.432	0.564	0.552	0.513
gi|190360639|ref|NP_001121941.1|	interferon-induced 17 kDa protein [Sus scrofa] >gi|182406743|gb|ACB87600.1| interferon stimulated gene 15 [Sus scrofa]	0.78	2.3	4.581	2.706
gi|335299628|ref|XP_001926139.3|	PREDICTED: coatomer subunit beta~ [Sus scrofa]	0.808	0.654	0.856	0.817
gi|311245810|ref|XP_001924714.2|	PREDICTED: GTP:AMP phosphotransferase, mitochondrial isoform 1 [Sus scrofa]	1.66	0.785	1.152	1.897
gi|345090993|ref|NP_001230731.1|	RAB8A, member RAS oncogene family [Sus scrofa]	0.701	0.514	0.564	0.856
gi|335296120|ref|XP_003357690.1|	PREDICTED: Golgi resident protein GCP60-like [Sus scrofa]	0.636	0.595	0.763	0.857
gi|335292526|ref|XP_001927459.2|	PREDICTED: receptor-interacting serine/threonine-protein kinase 3-like [Sus scrofa]	---	---	---	---
gi|350593418|ref|XP_003359535.2|	PREDICTED: hypothetical protein LOC100627320 [Sus scrofa]	1.522	1.994	2.167	1.535
gi|47522860|ref|NP_999184.1|	dehydrogenase/reductase SDR family member 4 [Sus scrofa] >gi|186972939|pdb|2ZAT|D Chain D, Crystal Structure Of A Mammalian	1.41	1.015	0.948	0.791

	Reductase >gi|186972937|pdb|2ZAT|B Chain B, Crystal Structure Of A Mammalian Reductase >gi|186972938|pdb|2ZAT|C Chain C, Crys				
gi|308153436|sp|Q8WNV7.2|DHRS4_PIG	RecName: Full=Dehydrogenase/reductase SDR family member 4; AltName: Full=NADPH-dependent carbonyl reductase/NADP-retinol dehydrogenase; Short=CR; Short=PHCR; AltName: Full=NADPH-dependent retinol dehydrogenase/reductase; Short=NDRD; AltName: Full=Pe				
gi|305855148|ref|NP_001182266.1|	peptidyl-prolyl cis-trans isomerase FKBP4 [Sus scrofa] >gi|285818414|gb|ADC38880.1| FK506 binding protein 4 [Sus scrofa]	0.978	0.691	0.784	1.009
gi|346644874|ref|NP_001231109.1|	T-complex protein 1 subunit gamma [Sus scrofa]	1.406	0.959	0.898	1.328
gi|350590014|ref|XP_003357929.2|	PREDICTED: serine/threonine-protein kinase 24 [Sus scrofa]	1.139	0.962	1.02	1.073
gi|335289671|ref|XP_003355951.1|	PREDICTED: transcription elongation factor SPT5 isoform 3 [Sus scrofa] >gi|335289667|ref|XP_003355949.1| PREDICTED: transcription elongation factor SPT5 isoform 1 [Sus scrofa] >gi|335289669|ref|XP_003355950.1| PREDICTED: transcription elongation fac	0.921	1.108	1.49	0.892
gi|311249625|ref|XP_003123708.1|	PREDICTED: FAS-associated factor 2 [Sus scrofa]	0.689	0.734	1.36	0.551
gi|350581449|ref|XP_003481037.1|	PREDICTED: guanine nucleotide-binding protein G(I)/G(S)/G(T) subunit beta-2-like [Sus scrofa] >gi|311251041|ref|XP_003124417.1| PREDICTED: guanine	1.102	0.709	0.749	0.867

	nucleotide-binding protein G(I)/G(S)/G(T) subunit beta-2-like isoform 2 [Sus scrofa]				
gi|311259100|ref|XP_003127931.1|	PREDICTED: meprin A subunit beta [Sus scrofa]	0.371	0.316	0.293	0.284
gi|350579827|ref|XP_003480692.1|	PREDICTED: LOW QUALITY PROTEIN: nucleosome assembly protein 1-like 4-like [Sus scrofa]	1.236	1.033	1.196	1.168
gi|356650697|gb|AET34758.1|	nucleosome assembly protein 1-like 4 [Sus scrofa]				
gi|343887420|ref|NP_001230609.1|	transcription elongation factor A protein 1 [Sus scrofa]	0.935	1.433	1.33	0.857
gi|343780936|ref|NP_001230484.1|	heterogeneous nuclear ribonucleoprotein H3 [Sus scrofa]	0.699	0.934	1.088	0.843
gi|194042010|ref|XP_001924773.1|	PREDICTED: gamma-adducin isoform 1 [Sus scrofa]	0.896	0.415	0.659	1.128
gi|194042012|ref|XP_001924864.1|	PREDICTED: gamma-adducin isoform 3 [Sus scrofa] >gi|335302227|ref|XP_003359413.1| PREDICTED: gamma-adducin [Sus scrofa]				
gi|335306217|ref|XP_003360420.1|	PREDICTED: 60S ribosomal protein L9-like [Sus scrofa]	1.077	0.628	0.964	1.517
gi|343478291|ref|NP_001230410.1|	ribosomal protein L9 [Sus scrofa]				
gi|350594715|ref|XP_001927122.3|	PREDICTED: adipocyte plasma membrane-associated protein-like [Sus scrofa]	0.721	0.215	0.2	0.678
gi|335293615|ref|XP_003357006.1|	PREDICTED: UDP-glucuronosyltransferase 2A1 [Sus scrofa]	0.731	0.513	0.642	0.911
gi|127804|sp|P26429.1|SC5A1_PIG	RecName: Full=Sodium/glucose cotransporter 1; Short=Na(+)/glucose cotransporter 1; AltName: Full=High affinity sodium-glucose cotransporter; AltName: Full=Solute carrier family 5 member 1 >gi|164675|gb|AAA31122.1| Na+/glucose cotransporter (SGLT1),	1.227	0.435	0.562	0.89
gi|255683521|ref|NP_001157493.1|	sodium/glucose cotransporter 1 [Sus scrofa]				
gi|335297787|ref|XP_003131	PREDICTED: puromycin-sensitive	1.467	0.977	0.697	1.664

575.2|	aminopeptidase isoform 1 [Sus scrofa]				
gi|350593466|ref|XP_003359556.2|	PREDICTED: UDP-glucose:glycoprotein glucosyltransferase 1 [Sus scrofa]	1.622	0.511	0.758	1.28
gi|1165145|emb|CAA64477.1|	annexin I [Sus scrofa]	0.423	0.578	0.912	0.848
gi|13399613|pdb|1HM6|A	Chain A, X-Ray Structure Of Full-Length Annexin 1 >gi|28948618|pdb|1MCX|A Chain A, Structure Of Full-Length Annexin A1 In The Presence Of Calcium >gi|13399614|pdb|1HM6|B Chain B, X-Ray Structure Of Full-Length Annexin 1				
gi|255683368|ref|NP_001157470.1|	annexin A1 [Sus scrofa] >gi|20141168|sp|P19619.3|ANXA1_PIG RecName: Full=Annexin A1; AltName: Full=Annexin I; AltName: Full=Annexin-1; AltName: Full=Calpactin II; AltName: Full=Calpactin-2; AltName: Full=Chromobindin-9; AltName: Full=Lipocortin I; A				
gi|343183313|ref|NP_001230231.1|	gephyrin [Sus scrofa]	1.148	1.148	1.4	1.115
gi|350584292|ref|XP_003481715.1|	PREDICTED: dynamin 1-like [Sus scrofa]	---	---	---	---
gi|350597090|ref|XP_003484356.1|	PREDICTED: dynamin-1-like protein-like, partial [Sus scrofa]				
gi|194038067|ref|XP_001927944.1|	PREDICTED: protein DEK isoform 1 [Sus scrofa]	0.564	0.737	1.077	0.544
gi|335291757|ref|XP_003356581.1|	PREDICTED: protein DEK [Sus scrofa]				
gi|335291759|ref|XP_003356582.1|	PREDICTED: protein DEK [Sus scrofa]				
gi|350587196|ref|XP_003482363.1|	PREDICTED: tryptophanyl-tRNA synthetase, cytoplasmic-like isoform 2 [Sus scrofa] >gi|350587194|ref|XP_003128776.3| PREDICTED: tryptophanyl-tRNA synthetase, cytoplasmic-like isoform 1 [Sus	1.241	0.79	0.815	0.894

	scrofa]				
gi|350578528|ref|XP_003480383.1|	PREDICTED: peptidyl-prolyl cis-trans isomerase B-like, partial [Sus scrofa]	---	---	---	---
gi|2494023|sp|Q29290.1|CYTB_PIG	RecName: Full=Cystatin-B; AltName: Full=Stefin-B	0.968	1.922	2.05	1.433
gi|311259195|ref|XP_003127983.1|	PREDICTED: plasminogen activator inhibitor 1 RNA-binding protein-like isoform 2 [Sus scrofa]	1.114	1.411	1.334	1.164
gi|311259199|ref|XP_003127984.1|	PREDICTED: plasminogen activator inhibitor 1 RNA-binding protein-like isoform 3 [Sus scrofa]				
gi|311259197|ref|XP_003127982.1|	PREDICTED: plasminogen activator inhibitor 1 RNA-binding protein-like isoform 1 [Sus scrofa]				
gi|335291374|ref|XP_003356484.1|	PREDICTED: plasminogen activator inhibitor 1 RNA-binding protein-like [Sus scrofa]				
gi|72535210|ref|NP_001026967.1|	protein FADD [Sus scrofa] >gi|46397561|gb|AAS91711.1| Fas-associating death domain-containing protein [Sus scrofa]	1.231	0.704	1.654	0.722
gi|350584002|ref|XP_003126228.3|	PREDICTED: keratin, type I cytoskeletal 18 [Sus scrofa]	0.903	0.753	0.534	0.611
gi|335309807|ref|XP_003361778.1|	PREDICTED: delta-aminolevulinic acid dehydratase-like, partial [Sus scrofa]	0.853	1.284	1.316	0.803
gi|194044992|ref|XP_001928105.1|	PREDICTED: sorting nexin-12 [Sus scrofa]	1.055	1.04	1.066	1.481
gi|350586335|ref|XP_003356548.2|	PREDICTED: nuclease-sensitive element-binding protein 1-like [Sus scrofa]	0.357	1.959	0.998	0.852
gi|354549569|gb|AER27829.1|	Y box binding protein 1 [Sus scrofa]				
gi|47523784|ref|NP_999528.1|	40S ribosomal protein S12 [Sus scrofa] >gi|1173191|sp|P46405.2|RS12_PIG RecName: Full=40S ribosomal protein S12 >gi|872315|emb|CAA55946.1| 40S ribosomal protein S12 [Sus scrofa]	0.974	1.068	0.878	1.607

gi|311260817|ref|XP_001925607.2|	PREDICTED: protein PML isoform 1 [Sus scrofa]	0.605	0.668	0.609	0.849
gi|335292411|ref|XP_003356725.1|	PREDICTED: protein PML isoform 2 [Sus scrofa]				
gi|350596898|ref|XP_003361788.2|	PREDICTED: GTP cyclohydrolase 1-like [Sus scrofa]	---	---	---	---
gi|311247118|ref|XP_003122486.1|	PREDICTED: NADH dehydrogenase [ubiquinone] flavoprotein 1, mitochondrial-like isoform 1 [Sus scrofa]	1.053	0.587	0.656	0.836
gi|311247120|ref|XP_003122487.1|	PREDICTED: NADH dehydrogenase [ubiquinone] flavoprotein 1, mitochondrial-like isoform 2 [Sus scrofa]				
gi|311262725|ref|XP_003129324.1|	PREDICTED: inorganic pyrophosphatase 2, mitochondrial-like [Sus scrofa]	1.016	1.01	0.817	0.985
gi|335287403|ref|XP_001927013.3|	PREDICTED: calcium-activated chloride channel regulator 4 [Sus scrofa]	1.181	0.665	0.95	0.821
gi|311253255|ref|XP_003125488.1|	PREDICTED: alanine aminotransferase 1-like [Sus scrofa]	1.108	1.782	2.46	1.848
gi|326633214|ref|NP_001192023.1|	ubiquitin-conjugating enzyme E2 variant 1 [Sus scrofa]	---	---	---	---
gi|335298430|ref|XP_003131953.2|	PREDICTED: LOW QUALITY PROTEIN: mitochondrial 2-oxoglutarate/malate carrier protein [Sus scrofa]	0.922	0.612	0.416	0.502
gi|311250943|ref|XP_003124353.1|	PREDICTED: 28 kDa heat- and acid-stable phosphoprotein-like [Sus scrofa]	0.855	1.997	1.66	1.733
gi|47522750|ref|NP_999125.1|	voltage-dependent anion-selective channel protein 1 [Sus scrofa] >gi|75050406|sp|Q9MZ16.3|VDAC1_PIG RecName: Full=Voltage-dependent anion-selective channel protein 1; Short=VDAC-1 >gi|8745552|gb|AAF78963.1|AF268461_1 voltage-dependent anion channel	0.661	0.45	0.434	0.729
gi|24987656|pdb|1LXE|A	Chain A, Crystal Structure Of The Cathelicidin Motif Of Protegrins >gi|24987482|pdb|1KWI|A	0.329	1.16	0.826	2.176

	Chain A, Crystal Structure Analysis Of The Cathelicidin Motif Of Protegrins				
gi|159162733|pdb|1N5P|A	Chain A, Solution Structure Of The Cathelin-Like Domain Of Protegrins (All Amide Bonds Involving Proline Residues Are In Trans Conformation) >gi|159162732|pdb|1N5H|A Chain A, Solution Structure Of The Cathelin-Like Domain Of Protegrins (The R87-P88				
gi|52000924|sp|Q29238.2|CLIC1_PIG	RecName: Full=Chloride intracellular channel protein 1; AltName: Full=Nuclear chloride ion channel 27	1.73	0.733	0.748	1.761
gi|194036322|ref|XP_001928206.1|	PREDICTED: Na(+)/H(+) exchange regulatory cofactor NHE-RF3 isoform 1 [Sus scrofa] >gi|194036324|ref|XP_001928212.1| PREDICTED: Na(+)/H(+) exchange regulatory cofactor NHE-RF3 isoform 2 [Sus scrofa]	1.081	1.545	1.68	1.125
gi|156120152|ref|NP_001095294.1|	complement factor B precursor [Sus scrofa] >gi|148724909|emb|CAN87697.1| B-factor, properdin [Sus scrofa]	1.303	0.829	0.753	1.237
gi|162138242|gb|ABX82825.1|	complement factor B [Sus scrofa]				
gi|335290029|ref|XP_003356052.1|	PREDICTED: nuclear pore glycoprotein p62 [Sus scrofa]	0.611	0.516	0.685	0.524
gi|335294212|ref|XP_003129427.2|	PREDICTED: 60S ribosomal protein L27a-like isoform 1 [Sus scrofa]	1.376	0.517	0.659	0.736
gi|350588060|ref|XP_003482544.1|	PREDICTED: 60S ribosomal protein L27a-like [Sus scrofa]				
gi|56404320|sp|Q6PWT7.1|CCS_PIG	RecName: Full=Copper chaperone for superoxide dismutase; AltName: Full=Superoxide dismutase copper chaperone >gi|49274647|ref|NP_001001866.1| copper chaperone for superoxide dismutase [Sus scrofa] >gi|46395042|gb|AAS91658.1| superoxide dismutase cop	1.032	1.247	1.516	1.198
gi|262399411|ref|NP_001161062.1|	uncharacterized protein C14orf142 homolog [Sus scrofa] >gi|212288175|sp|P0C8B5.1|	1.721	2.291	2.179	1.65

	CN142_PIG RecName: Full=Uncharacterized protein C14orf142 homolog				
gi|335293826|ref|XP_003357065.1|	PREDICTED: LOW QUALITY PROTEIN: short coiled-coil protein-like [Sus scrofa]	0.372	3.119	1.888	0.975
gi|329663948|ref|NP_001192334.1|	ras GTPase-activating protein-binding protein 1 [Sus scrofa]	0.664	0.971	1.069	0.932
gi|350587586|ref|XP_003129077.3|	PREDICTED: hypothetical protein LOC100522154 [Sus scrofa]	1.027	1.932	2.369	1.379
gi|350586208|ref|XP_003128041.3|	PREDICTED: epidermal growth factor receptor substrate 15 [Sus scrofa]	0.871	1.063	0.837	0.912
gi|555827|gb|AAA65943.1|	immunoglobulin alpha heavy chain constant region, partial [Sus scrofa] >gi|2136551|pir||I47175 Ig alpha chain C region - pig (fragment)	0.72	0.817	1.044	1.378
gi|290578621|gb|ADD51207.1|	immunoglobulin IgA heavy chain constant region [Sus scrofa]				
gi|311250752|ref|XP_003124284.1|	PREDICTED: NACHT, LRR and PYD domains-containing protein 6 [Sus scrofa]	1	2.178	1.782	1.213
gi|311249251|ref|XP_003123542.1|	PREDICTED: 6-phosphogluconolactonase-like [Sus scrofa]	1.405	0.796	0.756	1.009
gi|197251940|ref|NP_001127829.1|	actin-related protein 2/3 complex subunit 5 [Sus scrofa] >gi|195562237|gb|ACG50185.1| actin related protein 2/3 complex subunit 5 [Sus scrofa]	0.9	1.278	0.982	1.803
gi|311260144|ref|XP_003128362.1|	PREDICTED: butyrophilin subfamily 1 member A1-like [Sus scrofa]	---	---	---	---
gi|350586520|ref|XP_003128365.3|	PREDICTED: butyrophilin subfamily 1 member A1-like [Sus scrofa]				
gi|350586522|ref|XP_003482204.1|	PREDICTED: butyrophilin subfamily 1 member A1-like [Sus scrofa]				
gi|350581286|ref|XP_003124146.3|	PREDICTED: hypothetical protein LOC100516425 [Sus scrofa]	1.102	1.883	1.285	1.111
gi|350580953|ref|XP_003354274.2|	PREDICTED: 60S acidic ribosomal protein P1-like [Sus scrofa]	0.421	1.608	1.595	0.92
gi|47522916|ref|NP_999215.1|	glutathione S-transferase omega-1 [Sus	1.41	0.791	0.625	1.755

	scrofa] >gi|20141290|sp|Q9N1F5.2|GSTO1_PIG RecName: Full=Glutathione S-transferase omega-1; Short=GSTO-1; AltName: Full=Glutathione S-transferase omega 1-1; Short=GSTO 1-1; AltName: Full=Glutathione-dependent d				
gi|335293690|ref|XP_003129163.2|	PREDICTED: general vesicular transport factor p115-like [Sus scrofa]	0.836	2.522	2.176	1.516
gi|47523794|ref|NP_999534.1|	voltage-dependent anion-selective channel protein 2 [Sus scrofa] >gi|75050405|sp|Q9MZ15.1|VDAC2_PIG RecName: Full=Voltage-dependent anion-selective channel protein 2; Short=VDAC-2 >gi|8745554|gb|AAF78964.1|AF268462_1 voltage-dependent anion channel	0.872	0.478	0.433	0.922
gi|343432604|ref|NP_001230320.1|	ubiquitin-conjugating enzyme E2 variant 2 [Sus scrofa]	---	---	---	---
gi|335291035|ref|XP_003127861.2|	PREDICTED: polyadenylate-binding protein 4 isoform 1 [Sus scrofa]	---	---	---	---
gi|350585908|ref|XP_003482078.1|	PREDICTED: polyadenylate-binding protein 4 isoform 2 [Sus scrofa]				
gi|350586828|ref|XP_003482286.1|	PREDICTED: paired amphipathic helix protein Sin3a-like isoform 2 [Sus scrofa] >gi|335292401|ref|XP_003356722.1| PREDICTED: paired amphipathic helix protein Sin3a-like isoform 1 [Sus scrofa]	1.135	1.157	2.238	1.077
gi|5542425|pdb|1QPW|D	Chain D, Crystal Structure Determination Of Porcine Hemoglobin At 1.8a Resolution >gi|5542423|pdb|1QPW|B Chain B, Crystal Structure Determination Of Porcine Hemoglobin At 1.8a Resolution >gi|809285|pdb|2PGH|D Chain D, Structure Determination Of Aquo	0.842	0.728	0.606	0.619
gi|402550453|pdb|4F4O|H	Chain H, Structure Of The Haptoglobin-Haemoglobin				

	Complex >gi|402550447|pdb|4F4O|B Chain B, Structure Of The Haptoglobin-Haemoglobin Complex >gi|402550456|pdb|4F4O|K Chain K, Structure Of The Haptoglobin-Haemoglobin Complex >gi|402550450|pdb|4F4O|E				
gi|261245058|ref|NP_001138313.1|	hemoglobin subunit beta [Sus scrofa] >gi|3041678|sp|P02067.3|HBB_PIG RecName: Full=Hemoglobin subunit beta; AltName: Full=Beta-globin; AltName: Full=Hemoglobin beta chain >gi|806381|emb|CAA60490.1| beta-globin [Sus scrofa]				
gi|120564455|gb|ABM30153.1|	beta-globin [Sus scrofa]				
gi|1900|emb|CAA27575.1|	unnamed protein product [Sus scrofa]	1.005	0.503	0.769	1.152
gi|335892232|pdb|3N2F|B	Chain B, Crystal Structure Of The Sodium-Potassium Pump >gi|335892235|pdb|3N2F|D Chain D, Crystal Structure Of The Sodium-Potassium Pump >gi|319443575|pdb|3N23|D Chain D, Crystal Structure Of The High Affinity Complex Between Ouabain And The E2p For				
gi|48374077|ref|NP_001001542.1|	sodium/potassium-transporting ATPase subunit beta-1 [Sus scrofa] >gi|164380|gb|AAA31001.1| Na+, K+-ATPase [Sus scrofa]				
gi|1703469|sp|P05027.2|AT1B1_PIG	RecName: Full=Sodium/potassium-transporting ATPase subunit beta-1; AltName: Full=Sodium/potassium-dependent ATPase subunit beta-1 >gi|225192|prf||1211232A ATPase beta,Na/K >gi|1902|emb|CAA28301.1| unnamed protein product [Sus scrofa]				
gi|288965538|pdb|3KDP|D	Chain D, Crystal Structure Of The Sodium-Potassium				

	Pump >gi|288965535|pdb|3KDP|B Chain B, Crystal Structure Of The Sodium-Potassium Pump				
gi|114326183|ref|NP_001041537.1|	ADP-ribosylation factor 4 [Sus scrofa] >gi|221327932|gb|ACM17702.1| ADP-ribosylation factor 4 [Sus scrofa] >gi|203283175|gb|ACH96700.1| ADP-ribosylation factor 4 [Sus scrofa] >gi|62208269|gb|AAX77012.1| ADP-ribosylation factor family-like protein [S	0.686	0.442	0.301	0.822
gi|350582454|ref|XP_003125231.3|	PREDICTED: LOW QUALITY PROTEIN: leucine-rich PPR motif-containing protein, mitochondrial [Sus scrofa]	1.183	0.968	0.808	1.206
gi|311248919|ref|XP_003123379.1|	PREDICTED: protein Hook homolog 2 [Sus scrofa]	---	---	---	---
gi|311259611|ref|XP_003128182.1|	PREDICTED: 40S ribosomal protein S5-like isoform 2 [Sus scrofa] >gi|311259613|ref|XP_003128181.1| PREDICTED: 40S ribosomal protein S5-like isoform 1 [Sus scrofa]	1.156	0.55	0.608	1.144
gi|335290571|ref|XP_003356212.1|	PREDICTED: UPF0485 protein C1orf144 homolog isoform 2 [Sus scrofa]	0.702	1.491	1.375	0.757
gi|47522846|ref|NP_999175.1|	ATP-binding cassette sub-family G member 2 [Sus scrofa] >gi|68051987|sp|Q8MIB3.1|ABCG2_PIG RecName: Full=ATP-binding cassette sub-family G member 2; AltName: Full=Brain multidrug resistance protein; AltName: CD_antigen=CD338 >gi|21530795|emb|CAD1278	1.247	0.674	0.582	0.785
gi|350592328|ref|XP_001926837.4|	PREDICTED: kinesin family member 13B [Sus scrofa]	1.331	1.467	1.406	1.294
gi|103472127|ref|NP_001033715.2|	aldo-keto reductase family 1, member C-like 1 [Sus scrofa] >gi|94421332|gb|ABF18834.1| putative aldo-keto reductase family 1 member C4 [Sus scrofa]	1.037	1.744	2.546	3.783
gi|158148957|dbj|BAF82012.	aldo-keto reductase [Sus				

1|	scrofa] >gi|61741954|gb|AAX54862.1| prostaglandin F synthase [Sus scrofa]				
gi|178056496|ref|NP_001116547.1|	aldo-keto reductase family 1 member C4 [Sus scrofa] >gi|94962086|gb|ABF48390.1| aldo-keto reductase family 1 member C4 [Sus scrofa]				
gi|350586674|ref|XP_001929500.4|	PREDICTED: exportin-5 [Sus scrofa]	0.936	0.699	0.653	1.018
gi|335292368|ref|XP_001926489.3|	PREDICTED: calcium and integrin-binding protein 1 [Sus scrofa]	0.454	0.7	0.817	0.648
gi|38569723|gb|AAR24382.1|	GDP dissociation inhibitor 2 [Sus scrofa]	---	---	---	---
gi|350276172|ref|NP_001231880.1|	leucine-rich repeat-containing protein 59 [Sus scrofa]	0.495	0.761	0.604	0.665
gi|89573851|gb|ABD77151.1|	succinate dehydrogenase complex subunit B [Sus scrofa]	0.734	0.37	0.483	0.563
gi|322812295|pdb|3AE8|B	Chain B, Crystal Structure Of Porcine Heart Mitochondrial Complex Ii Bound With N-(3-Isopropoxy-Phenyl)-2-Trifluoromethylbenzamide >gi|322812327|pdb|3AEG|B Chain B, Crystal Structure Of Porcine Heart Mitochondrial Complex Ii Bound With N-Biphenyl-3-				
gi|345531797|pdb|3SFE|B	Chain B, Crystal Structure Of Porcine Mitochondrial Respiratory Complex Ii Bound With Oxaloacetate And Thiabendazole >gi|345531793|pdb|3SFD|B Chain B, Crystal Structure Of Porcine Mitochondrial Respiratory Complex Ii Bound With Oxaloacetate And Pent				
gi|157279735|ref|NP_001098423.1|	succinate dehydrogenase [ubiquinone] iron-sulfur subunit, mitochondrial precursor [Sus scrofa] >gi|122131481|sp|Q007T0.1|DHSB_PIG RecName: Full=Succinate dehydrogenase [ubiquinone] iron-sulfur subunit,				

	mitochondrial; AltName: Full=Iron-sulfur subuni				
gi|50262041|gb|AAT72764.1|	fatty acid binding protein 3, partial [Sus scrofa]	1.966	1.67	1.37	2.192
gi|374637318|gb|AEZ54788.1|	heart fatty acid-binding protein [Sus scrofa]				
gi|198250386|gb|ACH85195.1|	fatty acid binding protein 3 [Sus scrofa]				
gi|148792972|gb|ABR12603.1|	heart fatty acid-binding protein [Sus scrofa]				
gi|172073181|ref|NP_001093401.1|	fatty acid-binding protein, heart [Sus scrofa] >gi|2811020|sp|O02772.3|FABPH_PIG RecName: Full=Fatty acid-binding protein, heart; AltName: Full=Fatty acid-binding protein 3; AltName: Full=Heart-type fatty acid-binding protein; Short=H-FABP >gi|30237				
gi|224994935|gb|ACN76574.1|	fatty acid-binding protein 3 [Sus scrofa]				
gi|266018|gb|AAB25484.1|	calmodulin=low affinity vasoactive intestinal polypeptide binding protein [swine, liver, Peptide Partial, 34 aa]	1.397	2.473	2.726	4.203
gi|343790897|ref|NP_001230506.1|	eukaryotic translation initiation factor [Sus scrofa]	---	---	---	---
gi|335284027|ref|XP_003354495.1|	PREDICTED: LOW QUALITY PROTEIN: brain-specific angiogenesis inhibitor 1-associated protein 2-like protein 1-like [Sus scrofa]	---	---	---	---
gi|311259030|ref|XP_003127902.1|	PREDICTED: small nuclear ribonucleoprotein Sm D1-like [Sus scrofa]	1.41	1.528	1.301	0.682
gi|47522948|ref|NP_999231.1|	D-amino-acid oxidase [Sus scrofa] >gi|129305|sp|P00371.2|OXDA_PIG RecName: Full=D-amino-acid oxidase; Short=DAAO; Short=DAMOX; Short=DAO >gi|164306|gb|AAA30985.1| D-amino acid oxidase [Sus scrofa] >gi|48425898|pdb|1VE9|A Chain A, Porcine Kidney D-Am	1.109	0.677	0.555	0.816
gi|335310745|ref|XP_003125	PREDICTED: hypothetical protein	1.353	1.643	1.34	1.558

987.2|	LOC100515435, partial [Sus scrofa]				
gi|350582854|ref|XP_003481372.1|	PREDICTED: elongation factor 1-delta-like isoform 1 [Sus scrofa]				
gi|350582856|ref|XP_003481373.1|	PREDICTED: elongation factor 1-delta-like isoform 2 [Sus scrofa]				
gi|335279372|ref|XP_003121421.2|	PREDICTED: LOW QUALITY PROTEIN: myristoylated alanine-rich C-kinase substrate-like [Sus scrofa]	0.352	0.423	0.313	0.457
gi|356582293|ref|NP_001239141.1|	DEAH (Asp-Glu-Ala-His) box polypeptide 15 [Sus scrofa]	0.875	0.801	0.869	1.207
gi|335286637|ref|XP_001927783.3|	PREDICTED: nitrilase homolog 1 isoform 2 [Sus scrofa]	1.142	1.595	1.118	0.932
gi|335290948|ref|XP_003356342.1|	PREDICTED: uncharacterized protein KIAA1522-like [Sus scrofa]				
gi|335293906|ref|XP_003129266.2|	PREDICTED: annexin A5-like [Sus scrofa]	0.335	0.364	0.348	0.325
gi|350586356|ref|XP_003482168.1|	PREDICTED: ribosyldihydronicotinamide dehydrogenase [quinone]-like [Sus scrofa]	1.234	0.977	1.294	0.558
gi|311246132|ref|XP_003122091.1|	PREDICTED: 39S ribosomal protein L50, mitochondrial-like [Sus scrofa]	0.428	0.347	0.475	0.429
gi|350536531|ref|NP_001233199.1|	phosphatidylinositol-binding clathrin assembly protein [Sus scrofa] >gi|338784421|gb|AEI98873.1| phosphatidylinositol-binding clathrin assembly protein [Sus scrofa]	1.507	2.488	4.681	1.808
gi|350596835|ref|XP_003361703.2|	PREDICTED: phosphatidylinositol-binding clathrin assembly protein-like [Sus scrofa]				
gi|335310931|ref|XP_003362256.1|	PREDICTED: vacuolar protein sorting-associated protein 35 [Sus scrofa]	2.403	0.722	1.158	1.45
gi|335307273|ref|XP_003360776.1|	PREDICTED: mitogen-activated protein kinase 3, partial [Sus scrofa]	4.06	0.684	0.408	0.44
gi|164664456|ref|NP_001106915.1|	F-actin-capping protein subunit beta [Sus scrofa] >gi|148613359|gb|ABQ96223.1| F-actin capping protein beta subunit [Sus scrofa] >gi|148613353|gb|ABQ96220.1| F-actin capping protein beta	2.198	0.855	0.819	1.465

	subunit [Sus scrofa]				
gi|270000214|gb|ACZ57956.1|	F-actin capping protein beta subunit variant I [Sus scrofa]				
gi|270000216|gb|ACZ57957.1|	F-actin capping protein beta subunit variant II [Sus scrofa]				
gi|148222609|ref|NP_001090925.1|	F-actin-capping protein subunit beta [Sus scrofa] >gi|134035396|sp|A0PFK7.1|CAPZB_PIG RecName: Full=F-actin-capping protein subunit beta; AltName: Full=CapZ beta >gi|118627576|emb|CAL69437.1| F-actin capping protein subunit beta 1 [Sus scrofa]				
gi|346986241|ref|NP_001231394.1|	neurocalcin delta [Sus scrofa] >gi|346986243|ref|NP_001231395.1| neurocalcin delta [Sus scrofa]	1.329	2.765	2.675	1.757
gi|335294002|ref|XP_003357106.1|	PREDICTED: 3-hydroxybutyrate dehydrogenase type 2-like isoform 2 [Sus scrofa] >gi|335294000|ref|XP_003129333.2| PREDICTED: 3-hydroxybutyrate dehydrogenase type 2-like isoform 1 [Sus scrofa]	2.36	0.622	0.791	1.663
gi|335300663|ref|XP_003358983.1|	PREDICTED: trifunctional purine biosynthetic protein adenosine-3-like [Sus scrofa]	1.521	1.571	0.85	1.601
gi|335289705|ref|XP_003355965.1|	PREDICTED: flavin reductase-like [Sus scrofa]	1.05	1.11	1.107	1.542
gi|311261216|ref|XP_001924364.2|	PREDICTED: c-1-tetrahydrofolate synthase, cytoplasmic [Sus scrofa]	1.698	0.908	0.953	1.733
gi|311271203|ref|XP_001925518.2|	PREDICTED: ras-related protein Rab-4A-like [Sus scrofa]	---	---	---	---
gi|350585214|ref|XP_003481903.1|	PREDICTED: ras-related protein Rab-4B-like [Sus scrofa] >gi|311257582|ref|XP_003127192.1| PREDICTED: ras-related protein Rab-4B-like isoform 2 [Sus scrofa]				
gi|106073274|gb|ABF81975.1|	hypothetical protein [Sus scrofa]	1.521	1.032	1.234	1.545
gi|172072661|ref|NP_001116458.1|	tRNA-splicing ligase RtcB homolog [Sus				

	scrofa] >gi|118573909|sp|Q19PY3.1| RTCB_PIG RecName: Full=tRNA-splicing ligase RtcB homolog >gi|106073298|gb|ABF81976.1| hypothetical protein [Sus scrofa]				
gi|335289428|ref|XP_003127055.2|	PREDICTED: hypothetical protein LOC100523791 [Sus scrofa]	1.451	1.827	2.253	1.323
gi|347349312|gb|AEO80316.1|	programmed cell death protein 5 [Sus scrofa]				
gi|329663924|ref|NP_001192332.1|	tubulin-folding cofactor B-like [Sus scrofa] >gi|24528346|emb|CAD56044.1| cytoskeleton-associated protein 1 [Sus scrofa]	1.001	1.31	1.194	1.124
gi|350584424|ref|XP_003126577.3|	PREDICTED: chromodomain-helicase-DNA-binding protein 4 [Sus scrofa]	0.602	0.885	0.588	0.978
gi|178056560|ref|NP_001116630.1|	phosphoenolpyruvate carboxykinase, cytosolic [Sus scrofa] >gi|238863890|gb|ACR66221.1| phosphoenolpyruvate carboxykinase 1 [Sus scrofa] >gi|147223435|emb|CAN13128.1| phosphoenolpyruvate carboxykinase 1 (soluble) [Sus scrofa]	2.378	0.556	0.615	0.866
gi|350595271|ref|XP_003134762.3|	PREDICTED: hypothetical protein LOC100521760 [Sus scrofa]	1.787	1.246	1.2	1.484
gi|335310107|ref|XP_003361890.1|	PREDICTED: non-POU domain-containing octamer-binding protein [Sus scrofa]	1.266	0.594	0.636	0.877
gi|194043017|ref|XP_001928354.1|	PREDICTED: MGC159817 protein isoform 1 [Sus scrofa]	2.079	1.832	2.726	2.978
gi|350591430|ref|XP_003483266.1|	PREDICTED: protein SEC13 homolog [Sus scrofa]	0.852	0.702	0.715	0.613
gi|311270318|ref|XP_001925732.2|	PREDICTED: zinc transporter ZIP14 [Sus scrofa]	1.034	0.639	0.68	0.687
gi|311271598|ref|XP_001925392.2|	PREDICTED: bifunctional 3~-phosphoadenosine 5~-phosphosulfate synthase 2-like [Sus scrofa]	1.47	1.492	1.161	2.034
gi|47522660|ref|NP_999079.1|	fatty-acid amide hydrolase 1 [Sus scrofa] >gi|21542039|sp|Q9TUI8.1|FAAH1_PIG RecName:	1.107	0.598	1.183	0.743

	Full=Fatty-acid amide hydrolase 1; AltName: Full=Anandamide amidohydrolase 1; AltName: Full=Oleamide hydrolase 1 >gi|6451573|dbj|BAA86917.1| fatty acid amide hydro				
gi|350581741|ref|XP_003124660.3|	PREDICTED: phosphomannomutase 2-like, partial [Sus scrofa]	1.863	1.052	1.59	1.141
gi|311267482|ref|XP_003131586.1|	PREDICTED: chromobox protein homolog 1-like [Sus scrofa]	---	---	---	---
gi|130759|sp|P23687.1|PPCE_PIG	RecName: Full=Prolyl endopeptidase; Short=PE; AltName: Full=Post-proline cleaving enzyme >gi|51592147|ref|NP_001004050.1| prolyl endopeptidase [Sus scrofa] >gi|387766256|pdb|4AN1|A Chain A, Prolyl Oligopeptidase From Porcine Brain With A Covalently	2.184	0.997	1.054	1.385
gi|5107662|pdb|1QFM|A	Chain A, Prolyl Oligopeptidase From Porcine Muscle				
gi|50514023|pdb|1VZ3|A	Chain A, Prolyl Oligopeptidase From Porcine Brain, T597c Mutant				
gi|157879457|pdb|1O6F|A	Chain A, Prolyl Oligopeptidase From Porcine Brain, D641a Mutant With Bound Peptide Ligand Suc-Gly-Pro				
gi|334878488|pdb|1H2Z|A	Chain A, Prolyl Oligopeptidase From Porcine Brain, S554a Mutant With Bound Peptide Ligand Suc-Gly-Pro >gi|13096640|pdb|1E8M|A Chain A, Prolyl Oligopeptidase From Porcine Brain, Mutant, Complexed With Inhibitor >gi|37928243|pdb|1UOQ|A Chain A, Prolyl				
gi|157879458|pdb|1O6G|A	Chain A, Prolyl Oligopeptidase From Porcine Brain, D641n Mutant With Bound Peptide Ligand Suc-Gly-Pro				
gi|10835490|pdb|1E5T|A	Chain A, Prolyl Oligopeptidase From Porcine Brain, Mutant				
gi|27065054|pdb|1H2X|A	Chain A, Prolyl Oligopeptidase From Porcine Brain, Y473f Mutant >gi|27065055|pdb|1H2Y|A Chain A, Prolyl Oligopeptidase From				

	Porcine Brain, Y473f Mutant With Covalently Bound Inhibitor Z-Pro-Prolinal				
gi|50514022|pdb|1VZ2|A	Chain A, Prolyl Oligopeptidase From Porcine Brain, Y73cV427CC255T Mutant				
gi|350584879|ref|XP_003126924.3|	PREDICTED: alanyl-tRNA synthetase, cytoplasmic [Sus scrofa]	1.61	0.915	1.097	1.114
gi|56711362|ref|NP_001008689.1|	GTP-binding protein SAR1b [Sus scrofa] >gi|75061519|sp|Q5PYH3.1|SAR1B_PIG RecName: Full=GTP-binding protein SAR1b >gi|55977172|gb|AAV68380.1| Sar1b protein [Sus scrofa]	0.806	0.515	0.613	0.785
gi|62948008|gb|AAY23007.1|	GTP-binding protein SAR1b [Sus scrofa]				
gi|122058965|gb|ABM66369.1|	Sara2 protein [Sus scrofa]				
gi|350581244|ref|XP_003354387.2|	PREDICTED: transcription elongation regulator 1-like [Sus scrofa]	1.157	0.827	1.12	0.839
gi|311256485|ref|XP_003126672.1|	PREDICTED: protein SCAF11 [Sus scrofa]	0.564	0.884	0.534	0.89
gi|335301749|ref|XP_001925115.3|	PREDICTED: inorganic pyrophosphatase [Sus scrofa]	0.845	1.353	0.98	1.4
gi|311257919|ref|XP_003127356.1|	PREDICTED: aldehyde dehydrogenase family 16 member A1-like isoform 1 [Sus scrofa]	---	---	---	---
gi|311257921|ref|XP_003127357.1|	PREDICTED: aldehyde dehydrogenase family 16 member A1-like isoform 2 [Sus scrofa]				
gi|350590070|ref|XP_003131163.3|	PREDICTED: paraspeckle component 1 [Sus scrofa]	1.256	0.892	0.844	1.179
gi|343791007|ref|NP_001230549.1|	dynein, cytoplasmic 1, light intermediate chain 2 [Sus scrofa]	0.937	0.602	0.787	0.595
gi|194044484|ref|XP_001929185.1|	PREDICTED: eukaryotic translation initiation factor 2 subunit 2 [Sus scrofa]	0.878	0.871	0.883	0.824
gi|350583407|ref|XP_003481511.1|	PREDICTED: cathepsin S [Sus scrofa]	0.807	3.109	2.841	1.171
gi|298104126|ref|NP_001177126.1|	NADH dehydrogenase [ubiquinone] 1 alpha subcomplex subunit 8 [Sus	0.475	0.386	0.242	0.436

	scrofa]				
gi|350582355|ref|XP_003354851.2|	PREDICTED: exportin-1 [Sus scrofa]	2.597	2.292	1.444	1.821
gi|148237564|ref|NP_001090977.1|	adenylosuccinate synthetase isozyme 2 [Sus scrofa] >gi|189031715|sp|A4Z6H1.1|PURA2_PIG RecName: Full=Adenylosuccinate synthetase isozyme 2; Short=AMPSase 2; Short=AdSS 2; AltName: Full=Adenylosuccinate synthetase, acidic isozyme; AltName: Full=Adeny	2.151	1.756	2.876	1.842
gi|147225159|emb|CAN13336.1|	valyl-tRNA synthetase 2 [Sus scrofa]	0.936	0.861	1.053	1.209
gi|305855130|ref|NP_001182307.1|	valyl-tRNA synthetase [Sus scrofa] >gi|162138236|gb|ABX82822.1| valyl-tRNA synthetase [Sus scrofa]				
gi|347300500|ref|NP_001231502.1|	pre-mRNA-processing factor 40 homolog A [Sus scrofa]	0.486	0.417	0.497	0.543
gi|350592272|ref|XP_003483433.1|	PREDICTED: LOW QUALITY PROTEIN: PDZ and LIM domain protein 2-like [Sus scrofa]	0.472	0.971	1.074	0.77
gi|343887450|ref|NP_001230627.1|	acireductone dioxygenase 1 [Sus scrofa]	---	---	---	---
gi|335310208|ref|XP_003361930.1|	PREDICTED: NADH dehydrogenase [ubiquinone] 1 beta subcomplex subunit 4-like [Sus scrofa]	1.201	0.436	0.449	0.822
gi|297307137|ref|NP_001171999.1|	ran-specific GTPase-activating protein [Sus scrofa]	1.286	1.053	1.001	1.898
gi|237681312|ref|NP_001153744.1|	protein S100-A12 [Sus scrofa] >gi|126302600|sp|P80310.2|S10AC_PIG RecName: Full=Protein S100-A12; AltName: Full=Calgranulin-C; Short=CAGC; AltName: Full=Extracellular newly identified RAGE-binding protein; Short=EN-RAGE; AltName: Full=S100 calcium-b	0.787	1.875	1.494	2.476
gi|311275455|ref|XP_003134746.1|	PREDICTED: V-type proton ATPase subunit F-like [Sus scrofa]	1.032	1.05	1.458	1.027
gi|270047494|ref|NP_001161	fructose-1,6-bisphosphatase isozyme	1.007	0.651	0.666	0.64

104.2|	2 [Sus scrofa]				
gi|335300686|ref|XP_003358992.1|	PREDICTED: carbonyl reductase [NADPH] 1-like [Sus scrofa]	3.339	1.314	1.332	3.405
gi|350588305|ref|XP_003482618.1|	PREDICTED: methylosome subunit pICln-like [Sus scrofa]	---	---	---	---
gi|311245238|ref|XP_003121750.1|	PREDICTED: lisH domain and HEAT repeat-containing protein KIAA1468-like [Sus scrofa]	---	---	---	---
gi|219522002|ref|NP_001137188.1|	dual specificity mitogen-activated protein kinase kinase 1 [Sus scrofa] >gi|217314899|gb|ACK36984.1| mitogen-activated protein kinase kinase 1 [Sus scrofa]	---	---	---	---
gi|350580571|ref|XP_003480850.1|	PREDICTED: dual specificity mitogen-activated protein kinase kinase 2-like [Sus scrofa]				
gi|347300370|ref|NP_001231479.1|	mitogen-activated protein kinase kinase 2 [Sus scrofa]				
gi|350579773|ref|XP_001925384.4|	PREDICTED: U2 small nuclear ribonucleoprotein A~ [Sus scrofa]	0.703	0.733	0.655	0.683
gi|178056212|ref|NP_001116678.1|	tubulin polymerization-promoting protein [Sus scrofa] >gi|170178280|gb|ACB10579.1| tubulin polymerization promoting protein p25 alpha [Sus scrofa]	---	---	---	---
gi|350584885|ref|XP_003126937.3|	PREDICTED: splicing factor 3B subunit 3-like [Sus scrofa]	1.029	0.697	0.616	1.068
gi|62082662|gb|AAX62160.1|	glycogen synthase 1 [Sus scrofa]	---	---	---	---
gi|306029976|gb|ADM83431.1|	glycogen synthase 1 [Sus scrofa] >gi|306782595|ref|NP_001182437.1| glycogen [starch] synthase, muscle [Sus scrofa]				
gi|178056623|ref|NP_001116685.1|	vesicle-associated membrane protein-associated protein B [Sus scrofa] >gi|160210890|sp|A5GFS8.1|VAPB_PIG RecName: Full=Vesicle-associated membrane protein-associated protein B; Short=VAMP-B; Short=VAMP-associated protein B; Short=VAP-B >gi|147223393	0.473	0.849	0.623	0.782
gi|132566481|gb|ABO34136.	bactericidal permeability increasing	1.007	0.321	0.341	0.863

1|	protein [Sus scrofa]				
gi|226533693|ref|NP_001152779.1|	bactericidal permeability-increasing protein precursor [Sus scrofa] >gi|225922170|gb|ACO37155.1| bactericidal/permeability increasing protein [Sus scrofa]				
gi|347543763|ref|NP_001231548.1|	calmodulin-like 4 [Sus scrofa]	0.596	0.478	0.608	0.471
gi|240851536|ref|NP_001155875.1|	ubiquitin thioesterase OTUB1 [Sus scrofa] >gi|167888450|gb|ACA09613.1| OTUB1 [Sus scrofa]	1.916	1.458	1.235	1.685
gi|298231139|ref|NP_001177220.1|	pancreatic lipase-related protein 2 precursor [Sus scrofa] >gi|204307484|gb|ACI00231.1| pancreatic lipase-related protein 2 [Sus scrofa] >gi|204307482|gb|ACI00230.1| pancreatic lipase-related protein 2 [Sus scrofa]	1.485	1.001	0.449	0.797
gi|350580438|ref|XP_003480823.1|	PREDICTED: NADH dehydrogenase [ubiquinone] 1 beta subcomplex subunit 7-like [Sus scrofa]	0.407	0.396	0.258	0.394
gi|335310428|ref|XP_003362028.1|	PREDICTED: surfeit locus protein 4-like, partial [Sus scrofa]	0.968	0.242	0.24	0.501
gi|311259825|ref|XP_001924399.2|	PREDICTED: leucine-rich repeat-containing protein 16A [Sus scrofa]	1.062	1.41	1.161	1.191
gi|346986364|ref|NP_001231333.1|	serine/threonine-protein phosphatase 6 catalytic subunit [Sus scrofa]	---	---	---	---
gi|148887343|sp|Q9MYT8.4|ATP5I_PIG	RecName: Full=ATP synthase subunit e, mitochondrial; Short=ATPase subunit e	0.728	0.624	0.575	0.535
gi|311268019|ref|XP_003131834.1|	PREDICTED: carboxypeptidase D [Sus scrofa]	0.829	0.626	0.85	0.993
gi|148230268|ref|NP_001090970.1|	galectin-3 [Sus scrofa] >gi|124830340|gb|ABN15099.1| lectin galactoside-binding soluble 3 [Sus scrofa]	0.987	0.842	0.859	0.77
gi|157427728|ref|NP_001098772.1|	ATP-citrate synthase isoform 2 [Sus scrofa] >gi|380509279|gb|AFD64642.1| ATP citrate lyase short isoform [Sus scrofa] >gi|156140098|gb|ABU51323	0.96	0.574	0.853	0.991

	.1| ATP citrate lyase [Sus scrofa]				
gi|381140346|ref|NP_001244205.1|	ATP-citrate synthase isoform 1 [Sus scrofa] >gi|380509277|gb|AFD64641.1| ATP citrate lyase long isoform [Sus scrofa]				
gi|341942463|gb|AEL12455.1|	ATP citrate lyase [Sus scrofa]				
gi|335289328|ref|XP_003355850.1|	PREDICTED: ubiquinone biosynthesis protein COQ9, mitochondrial-like [Sus scrofa]	0.95	0.625	0.737	0.889
gi|335307327|ref|XP_003127745.2|	PREDICTED: heterogeneous nuclear ribonucleoprotein R [Sus scrofa]	---	---	---	---
gi|343488538|ref|NP_001230424.1|	cytoplasmic dynein 1 intermediate chain 2 [Sus scrofa]	0.686	1.581	1.663	1.087
gi|343790888|ref|NP_001230502.1|	uncharacterized protein LOC100627352 [Sus scrofa]	0.852	0.333	0.465	0.797
gi|311265230|ref|XP_003130552.1|	PREDICTED: 3~(2~),5~-bisphosphate nucleotidase 1-like isoform 2 [Sus scrofa]	1.739	1.093	0.851	2.146
gi|311265232|ref|XP_003130551.1|	PREDICTED: 3~(2~),5~-bisphosphate nucleotidase 1-like isoform 1 [Sus scrofa]				
gi|350597093|ref|XP_003362096.2|	PREDICTED: pyruvate kinase isozymes R/L-like [Sus scrofa]	---	---	---	---
gi|311269055|ref|XP_003132321.1|	PREDICTED: monoacylglycerol lipase ABHD6 [Sus scrofa]	0.846	0.276	0.256	0.585
gi|346421372|ref|NP_001231060.1|	ferritin, light polypeptide [Sus scrofa]	0.84	1.455	2.659	1.513
gi|311261863|ref|XP_003128900.1|	PREDICTED: cell growth-regulating nucleolar protein [Sus scrofa]	1.193	1.164	1.529	1.079
gi|346644844|ref|NP_001231177.1|	phosphotriesterase-related protein [Sus scrofa]	1.138	2.16	2.467	1.396
gi|7288152|dbj|BAA92850.1|	esterase D [Sus scrofa]	3.122	1.562	2.008	3.194
gi|47522936|ref|NP_999225.1|	S-formylglutathione hydrolase [Sus scrofa] >gi|62900112|sp|Q9GJT2.1|ESTD_PIG RecName: Full=S-formylglutathione hydrolase; Short=FGH; AltName: Full=Esterase D >gi|10443892|gb|AAG17630.1|AF254785_1 esterase D [Sus scrofa] >gi|9857260|dbj|BAB11922.1|e				

gi|348605151|ref|NP_001231720.1|	hydroxysteroid (17-beta) dehydrogenase 3 [Sus scrofa]	---	---	---	---
gi|350589482|ref|XP_003357775.2|	PREDICTED: BAG family molecular chaperone regulator 1-like isoform 1 [Sus scrofa]				
gi|347300398|ref|NP_001231491.1|	core histone macro-H2A.1 isoform 1 [Sus scrofa]	1.416	0.411	0.92	1.318
gi|347300400|ref|NP_001231492.1|	core histone macro-H2A.1 isoform 2 [Sus scrofa]				
gi|350588303|ref|XP_003129732.3|	PREDICTED: serine/threonine-protein kinase PAK 1-like [Sus scrofa]	1.086	0.799	0.761	0.84
gi|45268967|gb|AAS55896.1|	40S ribosomal protein S28, partial [Sus scrofa]	0.812	1.156	1.082	1.173
gi|48597082|ref|NP_001001587.1|	40S ribosomal protein S28 [Sus scrofa] >gi|51316555|sp|Q6QAT1.2|RS28_PIG RecName: Full=40S ribosomal protein S28				
gi|13878316|sp|P79273.1|ACADS_PIG	RecName: Full=Short-chain specific acyl-CoA dehydrogenase, mitochondrial; Short=SCAD; AltName: Full=Butyryl-CoA dehydrogenase; Flags: Precursor >gi|47522686|ref|NP_999063.1| short-chain specific acyl-CoA dehydrogenase, mitochondrial precursor [Sus s	1.815	0.506	0.361	0.938
gi|311252326|ref|XP_003125038.1|	PREDICTED: mannosyl-oligosaccharide glucosidase [Sus scrofa]	---	---	---	---
gi|166796059|ref|NP_001107755.1|	aminoacyl tRNA synthase complex-interacting multifunctional protein 1 [Sus scrofa] >gi|164598078|gb|ABY61326.1| small inducible cytokine subfamily E member 1 [Sus scrofa]	1.039	0.894	0.937	1.423
gi|297747346|ref|NP_001177097.1|	S100 calcium binding protein A14 [Sus scrofa]	0.868	1.211	1.289	0.925
gi|346716331|ref|NP_001231183.1|	ras-related protein Rab-5C [Sus scrofa]	0.994	1.048	0.475	0.811
gi|122142619|sp|Q19S50.1|STAT3_PIG	RecName: Full=Signal transducer and activator of transcription 3 >gi|113205774|ref|NP_001038045.	0.697	0.816	0.729	0.586

	1| signal transducer and activator of transcription 3 [Sus scrofa] >gi|315273320|gb|ADU03227.1| signal transducer and activator of transcription 3 isofor				
gi|315273324|gb|ADU03228.1|	signal transducer and activator of transcription 3 isoform 2 [Sus scrofa]				
gi|335284676|ref|XP_003124691.2|	PREDICTED: coronin-7-like [Sus scrofa]	2.172	1.572	1.581	1.798
gi|350582437|ref|XP_003481270.1|	PREDICTED: multiple coagulation factor deficiency protein 2-like [Sus scrofa]	---	---	---	---
gi|56788496|gb|AAW29969.1|	unknown [Sus scrofa] >gi|56792880|gb|AAW30632.1| unknown [Sus scrofa]	0.647	1.212	0.768	1.437
gi|311268139|ref|XP_003131895.1|	PREDICTED: sedoheptulokinase [Sus scrofa]	---	---	---	---
gi|335310769|ref|XP_003362184.1|	PREDICTED: THO complex subunit 4-like, partial [Sus scrofa]	0.639	0.688	0.742	0.587
gi|350589921|ref|XP_003482951.1|	PREDICTED: olfactomedin-4-like [Sus scrofa]				
gi|335284655|ref|XP_003124675.2|	PREDICTED: putative oxidoreductase GLYR1 isoform 1 [Sus scrofa]				
gi|335284657|ref|XP_003354668.1|	PREDICTED: putative oxidoreductase GLYR1 isoform 2 [Sus scrofa]				
gi|335284659|ref|XP_003354669.1|	PREDICTED: putative oxidoreductase GLYR1 isoform 3 [Sus scrofa]				
gi|319738636|ref|NP_001188360.1|	selenoprotein O [Sus scrofa]	---	---	---	---
gi|311256987|ref|XP_003126905.1|	PREDICTED: telomeric repeat-binding factor 2-interacting protein 1 [Sus scrofa]	1.368	2.792	2.63	1.525
gi|350591252|ref|XP_003132269.3|	PREDICTED: RNA-binding protein 5 [Sus scrofa]	---	---	---	---
gi|335281184|ref|XP_003353752.1|	PREDICTED: WD repeat-containing protein 5 [Sus scrofa]	0.726	1.433	1.209	1.175
gi|345199288|ref|NP_001230831.1|	protein phosphatase 1, regulatory (inhibitor) subunit 7 [Sus scrofa]	1.888	1.653	0.854	2.001
gi|311276634|ref|XP_003135	PREDICTED: mitochondrial import	0.885	1.602	1.803	0.802

287.1|	inner membrane translocase subunit Tim8 A-like [Sus scrofa]				
gi|350584903|ref|XP_003126945.3|	PREDICTED: AP-1 complex subunit gamma-1 isoform 1 [Sus scrofa]	1.115	0.685	0.787	1.059
gi|350584905|ref|XP_003481845.1|	PREDICTED: AP-1 complex subunit gamma-1 isoform 2 [Sus scrofa]				
gi|178056464|ref|NP_001116693.1|	beta-hexosaminidase subunit alpha precursor [Sus scrofa] >gi|169117926|gb|ACA43012.1| hexosaminidase A alpha polypeptide [Sus scrofa]	0.957	0.757	0.809	0.847
gi|385648284|ref|NP_001245315.1|	topoisomerase (DNA) II beta 180kDa [Sus scrofa]	0.719	0.554	0.635	0.674
gi|311260926|ref|XP_001924849.2|	PREDICTED: sec1 family domain-containing protein 1 [Sus scrofa]	1.944	0.808	1.404	1.612
gi|311248720|ref|XP_003123296.1|	PREDICTED: eukaryotic translation initiation factor 3 subunit G-like [Sus scrofa]	0.707	1.429	1.503	1.273
gi|335285938|ref|XP_003131986.2|	PREDICTED: Y-box-binding protein 2-like [Sus scrofa]	---	---	---	---
gi|350590843|ref|XP_003483149.1|	PREDICTED: Y-box-binding protein 2-like [Sus scrofa]				
gi|57528035|ref|NP_001009582.1|	stathmin [Sus scrofa] >gi|75043336|sp|Q6DUB7.3|STMN1_PIG RecName: Full=Stathmin >gi|49615355|gb|AAT66936.1| stathmin-1 [Sus scrofa]	0.699	1.231	1.255	1.081
gi|350578790|ref|XP_003121632.3|	PREDICTED: calcium-binding protein p22-like [Sus scrofa]	0.862	0.931	1.199	0.729
gi|350596649|ref|XP_003361456.2|	PREDICTED: calcium-binding protein p22-like [Sus scrofa]				
gi|350578792|ref|XP_003480452.1|	PREDICTED: calcium-binding protein p22-like isoform 1 [Sus scrofa]				
gi|4432942|dbj|BAA21081.1|	ribosomal protein S4 [Sus scrofa]	1.354	1.001	0.784	1.355
gi|324021713|ref|NP_001191212.1|	ribosomal protein S4 [Sus scrofa]				
gi|350590315|ref|XP_003483030.1|	PREDICTED: eukaryotic translation initiation factor 1-like [Sus scrofa]	0.736	1.146	1.26	1.239
gi|194043083|ref|XP_001928729.1|	PREDICTED: malectin-like [Sus scrofa]	0.792	0.388	0.53	0.878

gi|335309994|ref|XP_003130977.2|	PREDICTED: hypothetical protein LOC100522509, partial [Sus scrofa]	---	---	---	---
gi|89216|pir||PT0220	Ig lambda chain V-C region PLC3 - pig (fragment)	0.534	0.84	0.664	0.541
gi|164511|gb|AAA03572.1|	immunoglobulin lambda-chain, partial [Sus scrofa]				
gi|343183368|ref|NP_001230248.1|	immunoglobulin lambda-like polypeptide 5 precursor [Sus scrofa]				
gi|125947|sp|P01846.1|LAC_PIG	RecName: Full=Ig lambda chain C region				
gi|350592642|ref|XP_003483508.1|	PREDICTED: T-cell receptor beta chain T17T-22-like [Sus scrofa]				
gi|311271023|ref|XP_003133034.1|	PREDICTED: immunoglobulin lambda-like polypeptide 5-like [Sus scrofa]				
gi|350584919|ref|XP_003481848.1|	PREDICTED: IST1 homolog [Sus scrofa]	---	---	---	---
gi|335290688|ref|XP_003356248.1|	PREDICTED: hydroxymethylglutaryl-CoA lyase, mitochondrial-like isoform 1 [Sus scrofa]	0.92	1.174	0.679	0.763
gi|335310870|ref|XP_003362231.1|	PREDICTED: tumor protein D54-like, partial [Sus scrofa]	---	---	---	---
gi|19883961|sp|P35750.3|CAN1_PIG	RecName: Full=Calpain-1 catalytic subunit; AltName: Full=Calcium-activated neutral proteinase 1; Short=CANP 1; AltName: Full=Calpain mu-type; AltName: Full=Calpain-1 large subunit; AltName: Full=Micromolar-calpain; Short=muCANP >gi|8132993|gb|AAF734	1.292	1.074	1.2	1.214
gi|47522772|ref|NP_999137.1|	calpain-1 catalytic subunit [Sus scrofa] >gi|8132991|gb|AAF73443.1|AF263609_1 micromolar calcium-activated neutral protease 1 isoform B [Sus scrofa]				
gi|335282905|ref|XP_003354187.1|	PREDICTED: 60S ribosomal protein L18a-like isoform 4 [Sus scrofa] >gi|335282901|ref|XP_003354185.1| PREDICTED: 60S ribosomal protein L18a-like isoform 2 [Sus	0.673	0.483	0.4	0.83

	scrofa] >gi|335282903|ref|XP_003354186.1| PREDICTED: 60S ribosomal protein L18a-like isofor				
gi|2275401|gb|AAB63894.1|	ovarian sterol carrier protein 2, partial [Sus scrofa]	1.491	0.833	1.117	1.32
gi|350586177|ref|XP_003482126.1|	PREDICTED: non-specific lipid-transfer protein-like [Sus scrofa]				
gi|350587510|ref|XP_003482430.1|	PREDICTED: pre-mRNA 3~-end-processing factor FIP1-like [Sus scrofa]	0.645	0.841	1.135	1.033
gi|113205600|ref|NP_001038003.1|	guanine nucleotide-binding protein subunit alpha-11 [Sus scrofa] >gi|122142902|sp|Q2XSV9.2|GNA11_PIG RecName: Full=Guanine nucleotide-binding protein subunit alpha-11; Short=G alpha-11; Short=G-protein subunit alpha-11 >gi|84688557|gb|ABB71823.2| gu	1.305	0.455	0.502	0.734
gi|146741292|dbj|BAF62301.1|	guanine nucleotide-binding protein, alpha-11 [Sus scrofa]				
gi|350587233|ref|XP_003482374.1|	PREDICTED: cytoplasmic dynein 1 heavy chain 1-like [Sus scrofa]	---	---	---	---
gi|335310309|ref|XP_003361972.1|	PREDICTED: 39S ribosomal protein L12, mitochondrial-like, partial [Sus scrofa]	---	---	---	---
gi|121149|sp|P20735.1|GGT1_PIG	RecName: Full=Gamma-glutamyltranspeptidase 1; Short=GGT 1; AltName: Full=Gamma-glutamyltransferase 1; AltName: Full=Glutathione hydrolase 1; AltName: Full=Leukotriene-C4 hydrolase; AltName: CD_antigen=CD224; Contains: RecName: Full=Gamma-glutamyltra	0.92	0.941	1.029	0.802
gi|146741348|dbj|BAF62329.1|	gamma-glutamyltranspeptidase 1 [Sus scrofa]				
gi|193788671|ref|NP_001123277.1|	carnitine O-palmitoyltransferase 1, liver isoform [Sus scrofa] >gi|14579225|gb|AAK69171.1|AF288789_1 carnitine palmitoyltransferase I [Sus scrofa] >gi|222090415|gb|ACM42415	1.518	0.619	1.096	0.742

	.1| liver type carnitine palmitoyltransferase I [Sus scrofa]				
gi|312062795|ref|NP_001185851.1|	mitogen-activated protein kinase 1 [Sus scrofa] >gi|310789265|gb|ADP24686.1| extracellular signal-regulated kinase-2 [Sus scrofa]	---	---	---	---
gi|335310941|ref|XP_003362261.1|	PREDICTED: 40S ribosomal protein S2-like [Sus scrofa]	0.619	0.566	0.74	0.944
gi|124857|sp|P00998.1|ISK1_PIG	RecName: Full=Pancreatic secretory trypsin inhibitor; AltName: Full=Serine protease inhibitor Kazal-type 1 >gi|230351|pdb|1TGS|I Chain I, Three-Dimensional Structure Of The Complex Between Pancreatic Secretory Inhibitor (Kazal Type) And Trypsinogen	0.546	1.059	0.297	0.98
gi|229311|prf||700704A	inhibitor II,trypsin				
gi|335283733|ref|XP_003354394.1|	PREDICTED: pancreatic secretory trypsin inhibitor-like isoform 1 [Sus scrofa]				
gi|343432640|ref|NP_001230334.1|	39S ribosomal protein L44, mitochondrial [Sus scrofa]	1.053	0.959	0.789	0.981
gi|311252785|ref|XP_003125258.1|	PREDICTED: serine/arginine-rich splicing factor 7-like isoform 1 [Sus scrofa]	0.925	1.059	1.163	1.091
gi|350586583|ref|XP_003482220.1|	PREDICTED: serine/arginine-rich splicing factor 3-like [Sus scrofa]				
gi|350596827|ref|XP_003128414.2|	PREDICTED: serine/arginine-rich splicing factor 3-like [Sus scrofa]				
gi|311252787|ref|XP_003125259.1|	PREDICTED: serine/arginine-rich splicing factor 7-like isoform 2 [Sus scrofa]				
gi|311264036|ref|XP_003129968.1|	PREDICTED: 40S ribosomal protein S25-like isoform 2 [Sus scrofa] >gi|311264034|ref|XP_003129967.1| PREDICTED: 40S ribosomal protein S25-like isoform 1 [Sus scrofa]	0.408	0.174	0.194	0.439
gi|75054309|sp|Q8WNQ7.1|GALNS_PIG	RecName: Full=N-acetylgalactosamine-6-sulfatase; AltName: Full=Chondroitinsulfatase;	1.114	1.206	1.362	0.958

	Short=Chondroitinase; AltName: Full=Galactose-6-sulfate sulfatase; AltName: Full=N-acetylgalactosamine-6-sulfate sulfatase; Short=GalNAc6S sulfatase; Flags: Precurs				
gi|298160993|ref|NP_001177169.1|	glycerol-3-phosphate dehydrogenase [NAD+], cytoplasmic [Sus scrofa]	0.658	0.354	0.279	0.313
gi|350583346|ref|XP_003125776.3|	PREDICTED: ubiquitin associated protein 2-like isoform 3 [Sus scrofa]	0.946	1.471	1.696	1.033
gi|350596813|ref|XP_003484323.1|	PREDICTED: ubiquitin-associated protein 2-like [Sus scrofa]				
gi|347922148|ref|NP_001231673.1|	charged multivesicular body protein 5 [Sus scrofa]	0.442	0.792	1.053	0.549
gi|335289683|ref|XP_003355956.1|	PREDICTED: LOW QUALITY PROTEIN: mitochondrial import inner membrane translocase subunit TIM50-like [Sus scrofa]	0.891	1.032	0.715	0.748
gi|114326218|ref|NP_001041535.1|	hematological and neurological expressed 1-like protein [Sus scrofa] >gi|54114954|tpg|DAA01824.1| TPA_inf: HN1-like protein [Sus scrofa]	0.682	1.098	0.968	1.471
gi|346986315|ref|NP_001231313.1|	alpha-soluble NSF attachment protein [Sus scrofa]	1.129	0.696	0.769	0.769
gi|350592026|ref|XP_003132761.3|	PREDICTED: TBC1 domain family member 23-like [Sus scrofa]	0.957	0.835	1.06	0.95
gi|311255134|ref|XP_003126086.1|	PREDICTED: 2-amino-3-ketobutyrate coenzyme A ligase, mitochondrial-like isoform 1 [Sus scrofa] >gi|311255118|ref|XP_003126081.1| PREDICTED: 2-amino-3-ketobutyrate coenzyme A ligase, mitochondrial-like isoform 1 [Sus scrofa]	0.922	0.418	---	0.493
gi|350589397|ref|XP_003357727.2|	PREDICTED: aminopeptidase B-like [Sus scrofa]	1.773	1.562	1.107	1.839
gi|47716872|gb|AAT37622.1|	galectin-1 [Sus scrofa]	0.635	0.693	0.67	0.513
gi|311259452|ref|XP_003128108.1|	PREDICTED: 40S ribosomal protein S8-like [Sus scrofa]	1.064	0.634	0.671	1.375
gi|189043876|sp|A1XQU9.1|	RecName: Full=40S ribosomal	0.872	0.66	0.567	0.93

RS20_PIG	protein S20 >gi|194018718|ref|NP_001123426.1| 40S ribosomal protein S20 [Sus scrofa] >gi|117661119|gb|ABK55655.1| RPS20 [Sus scrofa]				
gi|113205666|ref|NP_001038019.1|	NADH dehydrogenase 1 beta subcomplex 6 [Sus scrofa] >gi|87047638|gb|ABD18452.1| NADH dehydrogenase 1 beta subcomplex 6 [Sus scrofa]	---	---	---	---
gi|311270922|ref|XP_003133013.1|	PREDICTED: up-regulated during skeletal muscle growth protein 5-like [Sus scrofa] >gi|311271791|ref|XP_003133221.1| PREDICTED: up-regulated during skeletal muscle growth protein 5-like [Sus scrofa]	---	---	---	---
gi|256838109|ref|NP_001157984.1|	phosphate carrier protein, mitochondrial [Sus scrofa] >gi|255964672|gb|ACU44653.1| solute carrier family 25 member 3 [Sus scrofa]	0.704	0.387	0.381	0.659
gi|343790856|ref|NP_001230567.1|	glutathione transferase zeta 1 [Sus scrofa]	1.644	0.741	0.595	1.333
gi|195972993|ref|NP_001124446.1|	stromal interaction molecule 1 precursor [Sus scrofa] >gi|157929328|gb|ABW03922.1| stromal interaction molecule 1 [Sus scrofa]				
gi|178056753|ref|NP_001116644.1|	glycogen phosphorylase, liver form [Sus scrofa] >gi|167834153|gb|ACA02827.1| glycogen storage disease type VI-related protein [Sus scrofa]	---	---	---	---
gi|350589278|ref|XP_003130571.3|	PREDICTED: melanoma inhibitory activity protein 3-like, partial [Sus scrofa]	0.752	1.073	0.757	0.72
gi|335287593|ref|XP_003355390.1|	PREDICTED: ras-related C3 botulinum toxin substrate 2-like [Sus scrofa]	---	---	---	---
gi|342672022|ref|NP_001230144.1|	exportin-2 [Sus scrofa]	1.359	0.529	0.497	1.03
gi|311274576|ref|XP_003134	PREDICTED: small nuclear	---	---	---	---

384.1|	ribonucleoprotein-associated protein B~-like [Sus scrofa]				
gi|343887452|ref|NP_001230628.1|	small nuclear ribonucleoprotein polypeptide N [Sus scrofa]				
gi|381352839|gb|AFG25597.1|	SNRPN [Sus scrofa]				
gi|335292262|ref|XP_001927373.3|	PREDICTED: isocitrate dehydrogenase [NAD] subunit alpha, mitochondrial [Sus scrofa]	0.941	0.61	1.02	0.702
gi|335281115|ref|XP_001926630.3|	PREDICTED: nuclear pore complex protein Nup214 [Sus scrofa]	0.588	1.43	0.797	0.893
gi|350587460|ref|XP_003128981.3|	PREDICTED: sister chromatid cohesion protein PDS5 homolog A-like [Sus scrofa]	---	---	---	---
gi|219521990|ref|NP_001137182.1|	granzyme B precursor [Sus scrofa] >gi|216408333|gb|ACJ72857.1| granzyme B [Sus scrofa]	1.167	3.528	4.173	3.057
gi|335299970|ref|XP_003358743.1|	PREDICTED: DCN1-like protein 1-like [Sus scrofa]	---	---	---	---
gi|194040450|ref|XP_001927992.1|	PREDICTED: lactoylglutathione lyase isoform 1 [Sus scrofa]	1.245	1.591	3.199	2.25
gi|350586543|ref|XP_003482212.1|	PREDICTED: glutamate--cysteine ligase catalytic subunit isoform 2 [Sus scrofa]	2.588	1.351	1.762	2.034
gi|51702802|sp|P62901.1|RL31_PIG	RecName: Full=60S ribosomal protein L31 >gi|350582083|ref|XP_003481192.1| PREDICTED: 60S ribosomal protein L31-like isoform 4 [Sus scrofa] >gi|350582079|ref|XP_003481190.1| PREDICTED: 60S ribosomal protein L31-like isoform 2 [Sus scrofa] >gi|3505820	0.573	0.991	0.69	0.994
gi|349585075|ref|NP_001231802.1|	small acidic protein [Sus scrofa]	---	---	---	---
gi|47524464|gb|AAT34965.1|	2~-5~ oligoadenylate synthetase 1 [Sus scrofa]	---	---	---	---
gi|740987|prf||2006263A	Arg Gly amidinotransferase	0.768	0.399	0.54	0.432
gi|190360601|ref|NP_001121914.1|	glycine amidinotransferase, mitochondrial [Sus scrofa] >gi|308153447|sp|P50441.2|GATM_PIG RecName: Full=Glycine				

	amidinotransferase, mitochondrial; AltName: Full=L-arginine:glycine amidinotransferase; AltName: Full=Transamidinase; Flags: Precursor >g				
gi|350594954|ref|XP_003360054.2|	PREDICTED: adenosine deaminase [Sus scrofa]	0.473	0.933	0.854	1.307
gi|350582315|ref|XP_003481243.1|	PREDICTED: NFU1 iron-sulfur cluster scaffold homolog, mitochondrial-like [Sus scrofa]	0.809	1.818	1.689	1.055
gi|311276040|ref|XP_003135024.1|	PREDICTED: LOW QUALITY PROTEIN: acyl-coenzyme A thioesterase 9, mitochondrial-like [Sus scrofa]	1.339	0.598	0.958	0.858
gi|75074354|sp|Q9GL51.3|LIS1_PIG	RecName: Full=Platelet-activating factor acetylhydrolase IB subunit alpha; AltName: Full=Lissencephaly-1 protein; Short=LIS-1; AltName: Full=PAF acetylhydrolase 45 kDa subunit; Short=PAF-AH 45 kDa subunit; AltName: Full=PAF-AH alpha; Short=PAFAH alp	0.992	0.695	0.644	0.883
gi|350590470|ref|XP_003131623.3|	PREDICTED: canalicular multispecific organic anion transporter 2 [Sus scrofa]	0.962	0.719	0.732	0.732
gi|47522764|ref|NP_999133.1|	integrin beta-1 precursor [Sus scrofa] >gi|75050024|sp|Q9GLP0.1|ITB1_PIG RecName: Full=Integrin beta-1; AltName: Full=Fibronectin receptor subunit beta; AltName: Full=VLA-4 subunit beta; AltName: CD_antigen=CD29; Flags: Precursor >gi|10336839|gb|AAG	---	---	---	---
gi|335281543|ref|XP_003122613.2|	PREDICTED: protein fat-free homolog [Sus scrofa]	---	---	---	---
gi|350587133|ref|XP_001926860.4|	PREDICTED: thyroid receptor-interacting protein 11 [Sus scrofa]	0.712	1.283	0.892	1.203
gi|350595681|ref|XP_003135172.3|	PREDICTED: structural maintenance of chromosomes protein 1A-like [Sus scrofa]	---	---	---	---
gi|346716326|ref|NP_001231	SWI/SNF-related matrix-associated	0.627	0.547	0.905	0.991

181.1|	actin-dependent regulator of chromatin subfamily D member 2 [Sus scrofa]				
gi|145279215|ref|NP_001077410.1|	angiotensin-converting enzyme isoform 2 precursor [Sus scrofa] >gi|119381511|gb|ABL73884.1| angiotensin I converting enzyme [Sus scrofa]	1.814	1.096	2.012	1.498
gi|311273877|ref|XP_003134081.1|	PREDICTED: 28S ribosomal protein S27, mitochondrial-like [Sus scrofa]	1.338	0.635	0.986	1.095
gi|350587174|ref|XP_003356842.2|	PREDICTED: serpin A3-6 [Sus scrofa]	1.228	0.874	1.477	0.914
gi|335295413|ref|XP_003130208.2|	PREDICTED: calcium-binding mitochondrial carrier protein Aralar2 [Sus scrofa]	0.922	0.459	0.572	0.493
gi|400294561|gb|AFP81702.1|	solute carrier family 27 member 4, partial [Sus scrofa]	0.627	0.518	0.399	0.655
gi|183228163|gb|ACC59785.1|	PRP19/PSO4 pre-mRNA processing factor 19-like protien [Sus scrofa]	1.04	1.003	0.467	0.837
gi|190360605|ref|NP_001121929.1|	pre-mRNA-processing factor 19 [Sus scrofa] >gi|356995561|dbj|BAL14717.1| nuclear matrix protein 200 [Sus scrofa] >gi|171920012|gb|ACB59178.1| pre-mRNA processing factor 19 [Sus scrofa]				
gi|55926217|ref|NP_001007518.1|	cytochrome c oxidase subunit 5B, mitochondrial precursor [Sus scrofa] >gi|75042739|sp|Q5S3G4.1|COX5B_PIG RecName: Full=Cytochrome c oxidase subunit 5B, mitochondrial; AltName: Full=Cytochrome c oxidase polypeptide Vb; Flags: Precursor >gi|55583747|g	0.467	0.331	0.397	0.379
gi|350580071|ref|XP_003122734.3|	PREDICTED: oxysterol-binding protein 1 [Sus scrofa]	0.815	1.016	0.753	0.838
gi|148226851|ref|NP_001090933.1|	ATP synthase subunit f, mitochondrial [Sus scrofa] >gi|117660512|gb|ABK55624.1| mitochondrial Atp5j2 [Sus scrofa]	0.81	0.22	0.278	0.434
gi|158518654|sp|Q95339.4|ATPK_PIG	RecName: Full=ATP synthase subunit f, mitochondrial				
gi|251764755|sp|A6M931.1|I	RecName: Full=Eukaryotic initiation	1.469	0.813	0.904	1.401

F4A3_PIG	factor 4A-III; Short=eIF-4A-III; Short=eIF4A-III; AltName: Full=ATP-dependent RNA helicase DDX48; AltName: Full=ATP-dependent RNA helicase eIF4A-3; AltName: Full=DEAD box protein 48; AltName: Full=Eukaryotic trans				
gi|350586361|ref|XP_003356557.2|	PREDICTED: serine/threonine-protein kinase PRP4 homolog [Sus scrofa]	0.523	0.604	0.573	0.522
gi|335290248|ref|XP_003127516.2|	PREDICTED: hsp70-binding protein 1-like [Sus scrofa]	---	---	---	---
gi|342349338|ref|NP_001230145.1|	elaC homolog 2 [Sus scrofa]	---	---	---	---
gi|350590206|ref|XP_003131326.3|	PREDICTED: protein kinase C alpha type, partial [Sus scrofa]	---	---	---	---
gi|49274643|ref|NP_001001864.1|	amine oxidase [flavin-containing] B [Sus scrofa] >gi|62899647|sp|Q6PLK3.3|AOFB_PIG RecName: Full=Amine oxidase [flavin-containing] B; AltName: Full=Monoamine oxidase type B; Short=MAO-B >gi|46909483|gb|AAT06259.1| monoamine oxidase B [Sus scrofa]	0.901	0.405	0.432	0.776
gi|335280279|ref|XP_001928782.2|	PREDICTED: 26S protease regulatory subunit 10B [Sus scrofa]	1.174	0.812	0.717	1.112
gi|350583020|ref|XP_003481419.1|	PREDICTED: zinc finger protein 706-like [Sus scrofa]	1.271	1.945	2.395	1.9
gi|195539476|ref|NP_001124208.1|	bifunctional purine biosynthesis protein PURH [Sus scrofa] >gi|192383834|gb|ACF04802.1| 5-aminoimidazole-4-carboxamide ribonucleotide formyltransferase/IMP cyclohydrolase [Sus scrofa]	1.674	0.855	1.284	1.949
gi|113205898|ref|NP_001038078.1|	microtubule-associated protein RP/EB family member 1 [Sus scrofa] >gi|81174752|gb|ABB58918.1| microtubule-associated protein RP/EB family member 1 [Sus scrofa]	---	---	---	---
gi|335308271|ref|XP_003361	PREDICTED: pre-mRNA-processing	---	---	---	---

162.1|	factor 6-like [Sus scrofa]				
gi|60394813|sp|Q29201.4|RS16_PIG	RecName: Full=40S ribosomal protein S16 >gi|212549659|ref|NP_001131092.1| 40S ribosomal protein S16 [Sus scrofa] >gi|335289675|ref|XP_003355953.1| PREDICTED: 40S ribosomal protein S16-like isoform 2 [Sus scrofa] >gi|335289677|ref|XP_003355954.1| PRE	1.01	0.711	0.563	1.304
gi|350588052|ref|XP_003357166.2|	PREDICTED: importin-7, partial [Sus scrofa]	1.07	1.011	0.855	0.961
gi|222090420|gb|ACM42418.1|	carnitine palmitoyltransferase II [Sus scrofa]	1.253	0.307	0.223	0.492
gi|229358270|gb|ACQ57802.1|	mitochondrial carnitine palmitoyltransferase II [Sus scrofa]				
gi|350536125|ref|NP_001233172.1|	carnitine O-palmitoyltransferase 2, mitochondrial precursor [Sus scrofa] >gi|294805368|gb|ADF42518.1| mitochondrial carnitine palmitoyltransferase II [Sus scrofa]				
gi|113205668|ref|NP_001038021.1|	microsomal glutathione S-transferase 3 [Sus scrofa] >gi|120564453|gb|ABM30152.1| microsomal glutathione S-transferase 3 [Sus scrofa] >gi|87047644|gb|ABD18455.1| microsomal glutathione S-transferase 3 [Sus scrofa]	1.689	0.87	0.986	0.95
gi|335309593|ref|XP_003361696.1|	PREDICTED: 39S ribosomal protein L12, mitochondrial-like, partial [Sus scrofa]	0.419	0.93	0.562	0.587
gi|346716090|ref|NP_001231205.1|	thioredoxin-like protein 1 [Sus scrofa]	1.061	0.842	0.83	0.965
gi|12055551|emb|CAC21172.1|	diazepam binding inhibitor [Sus scrofa]	1.667	2.489	2.516	1.486
gi|110825776|sp|P12026.2|ACBP_PIG	RecName: Full=Acyl-CoA-binding protein; Short=ACBP; AltName: Full=Diazepam-binding inhibitor; Short=DBI; AltName: Full=Endozepine; Short=EP; Contains: RecName: Full=DBI(32-86) >gi|47523046|ref|N				

	P_999284.1| acyl-CoA-binding protein [Sus scrofa] >gi|1				
gi|281427368|ref|NP_001163992.1|	steroid hormone receptor ERR1 [Sus scrofa] >gi|229609707|gb|ACQ83469.1| ERRa [Sus scrofa]	---	---	---	---
gi|297632416|ref|NP_001172092.1|	enhancer of rudimentary homolog [Sus scrofa]	0.36	0.948	1.154	0.569
gi|118403852|ref|NP_001072139.1|	UBC1 [Sus scrofa] >gi|115371727|gb|ABI96188.1| UBC1 [Sus scrofa]	1.67	1.155	1.259	1.448
gi|343887409|ref|NP_001230603.1|	deoxynucleotidyltransferase terminal-interacting protein 2 [Sus scrofa]	---	---	---	---
gi|347300356|ref|NP_001231473.1|	uncharacterized protein LOC100689297 [Sus scrofa]	1.224	1.686	1.919	1.106
gi|311247993|ref|XP_003122918.1|	PREDICTED: probable methylthioribulose-1-phosphate dehydratase-like [Sus scrofa]	1.308	1.874	2.593	1.715
gi|349732262|ref|NP_001231870.1|	3-hydroxyanthranilate 3,4-dioxygenase [Sus scrofa]	0.477	0.418	0.343	0.525
gi|311252389|ref|XP_003125056.1|	PREDICTED: STAM-binding protein [Sus scrofa]	---	---	---	---
gi|343790890|ref|NP_001230503.1|	acyl-CoA dehydrogenase family, member 8 [Sus scrofa]	1.868	1.044	1.462	1.699
gi|343780946|ref|NP_001230486.1|	D-dopachrome decarboxylase [Sus scrofa]	1.248	1.873	1.422	1.566
gi|335289112|ref|XP_003126904.2|	PREDICTED: lysyl-tRNA synthetase isoform 1 [Sus scrofa]	1.504	0.655	0.639	1.189
gi|335289114|ref|XP_003355791.1|	PREDICTED: lysyl-tRNA synthetase isoform 2 [Sus scrofa]				
gi|350587855|ref|XP_003129278.3|	PREDICTED: protein transport protein Sec24D [Sus scrofa]	---	---	---	---
gi|335309819|ref|XP_003361783.1|	PREDICTED: charged multivesicular body protein 2a-like, partial [Sus scrofa]	---	---	---	---
gi|113205716|ref|NP_001038033.1|	1,5-anhydro-D-fructose reductase [Sus scrofa] >gi|94421324|gb|ABF18830.1| putative aldo-keto reductase family 1 member CL2 [Sus scrofa]	1.44	1.224	0.906	1.522
gi|239938601|sp|P82125.2|AKCL2_PIG	RecName: Full=1,5-anhydro-D-fructose				

	reductase; Short=AF reductase; AltName: Full=Aldo-keto reductase family 1 member C-like protein 2; Short=Aldo-keto reductase family 1 member CL2; AltName: Full=Aldo-keto reductase family 1 member E2				
gi|350589668|ref|XP_003482894.1|	PREDICTED: 1,5-anhydro-D-fructose reductase-like [Sus scrofa]				
gi|311258972|ref|XP_003127870.1|	PREDICTED: adenylyl cyclase-associated protein 1-like [Sus scrofa]	---	---	---	---
gi|266686|sp|P29804.1|ODPA_PIG	RecName: Full=Pyruvate dehydrogenase E1 component subunit alpha, somatic form, mitochondrial; AltName: Full=PDHE1-A type I; Flags: Precursor >gi|1851|emb|CAA37180.1| pyruvate dehydrogenase (lipoamide) [Sus scrofa domesticus]	1.493	0.329	0.515	0.96
gi|448580|prf||1917268A	pyruvate dehydrogenase:SUBUNIT=alpha				
gi|350595555|ref|XP_003360292.2|	PREDICTED: pyruvate dehydrogenase E1 component subunit alpha, somatic form, mitochondrial isoform 1 [Sus scrofa]				
gi|350595557|ref|XP_003484129.1|	PREDICTED: pyruvate dehydrogenase E1 component subunit alpha, somatic form, mitochondrial isoform 2 [Sus scrofa]				
gi|350539577|ref|NP_001233167.1|	coatomer subunit gamma-2 [Sus scrofa] >gi|301087607|gb|ADK56184.1| coatomer protein complex subunit gamma 2 [Sus scrofa]	1.059	0.472	0.381	0.867
gi|1262435|emb|CAA61864.1|	put. 26S protease subunit [Sus scrofa]	0.765	0.82	0.599	0.929
gi|47522792|ref|NP_999148.1|	26S protease regulatory subunit 8 [Sus scrofa] >gi|49065781|sp|P62197.1|PRS8_PIG RecName: Full=26S protease regulatory subunit 8; AltName: Full=26S proteasome AAA-ATPase				

	subunit RPT6; AltName: Full=Proteasome 26S subunit ATPase 5; AltName: Full=Prot				
gi|311252444|ref|XP_003125100.1|	PREDICTED: small nuclear ribonucleoprotein G-like [Sus scrofa]	0.638	0.44	0.527	0.508
gi|194036463|ref|XP_001928684.1|	PREDICTED: ras-related protein Rap-1A isoform 1 [Sus scrofa] >gi|335287176|ref|XP_003355289.1| PREDICTED: ras-related protein Rap-1A isoform 2 [Sus scrofa]	0.726	0.228	0.197	0.487
gi|350584241|ref|XP_003481702.1|	PREDICTED: ras-related protein Rap-1b [Sus scrofa] >gi|350596871|ref|XP_003484329.1| PREDICTED: ras-related protein Rap-1b-like [Sus scrofa]				
gi|51512143|gb|AAU05316.1|	hepatitis B virus x interacting protein [Sus scrofa]	---	---	---	---
gi|187471167|sp|Q66X52.2|HBXIP_PIG	RecName: Full=Hepatitis B virus X-interacting protein homolog; Short=HBV X-interacting protein homolog; Short=HBX-interacting protein homolog				
gi|335307770|ref|XP_003360968.1|	PREDICTED: hepatitis B virus X-interacting protein homolog, partial [Sus scrofa]				
gi|350595155|ref|XP_003360146.2|	PREDICTED: maltase-glucoamylase, intestinal-like, partial [Sus scrofa]	0.458	0.608	0.571	0.338
gi|350597052|ref|XP_003361964.2|	PREDICTED: maltase-glucoamylase, intestinal-like, partial [Sus scrofa]				
gi|350580871|ref|XP_003123769.3|	PREDICTED: ras GTPase-activating-like protein IQGAP2-like [Sus scrofa]	0.962	0.63	0.649	1.002
gi|345199274|ref|NP_001230825.1|	glutaredoxin 3 [Sus scrofa]	1.323	0.863	0.929	1.587
gi|335283420|ref|XP_003123942.2|	PREDICTED: protein PRRC1-like, partial [Sus scrofa]	1.596	1.527	0.935	1.343
gi|335301657|ref|XP_001929317.3|	PREDICTED: coiled-coil domain-containing protein 6 [Sus scrofa]	---	---	---	---
gi|335308509|ref|XP_003361258.1|	PREDICTED: microsomal glutathione S-transferase 2-like,	0.711	0.282	0.335	0.585

	partial [Sus scrofa]				
gi|346421419|ref|NP_001231084.1|	lysosome membrane protein 2 precursor [Sus scrofa]	0.696	0.52	0.332	0.768
gi|163915141|ref|NP_001106518.1|	carnitine O-acetyltransferase [Sus scrofa] >gi|162852671|emb|CAP49176.1| carnitine acetyl transferase [Sus scrofa]	1.806	1.038	1.109	1.343
gi|239504558|ref|NP_001155112.1|	caspase-10 [Sus scrofa] >gi|229365449|dbj|BAH57972.1| caspase 10 [Sus scrofa]	1.116	0.668	0.738	0.988
gi|350590094|ref|XP_003357986.2|	PREDICTED: putative sodium-coupled neutral amino acid transporter 10 isoform 1 [Sus scrofa]	---	---	---	---
gi|350590096|ref|XP_003482987.1|	PREDICTED: putative sodium-coupled neutral amino acid transporter 10 isoform 2 [Sus scrofa]				
gi|62510530|sp|Q8MJ30.1|DHPR_PIG	RecName: Full=Dihydropteridine reductase; AltName: Full=HDHPR; AltName: Full=Quinoid dihydropteridine reductase >gi|47523746|ref|NP_999508.1| dihydropteridine reductase [Sus scrofa] >gi|22094813|gb|AAM91996.1|AF526879_1 quinoid dihydropteridine redu	---	---	---	---
gi|164023822|ref|NP_001106688.1|	60S ribosomal protein L7 [Sus scrofa] >gi|56384247|gb|AAV85772.1| ribosomal protein L7 [Sus scrofa]	0.808	0.736	0.535	1.166
gi|47522730|ref|NP_999115.1|	60S ribosomal protein L29 [Sus scrofa] >gi|51704218|sp|Q95281.4|RL29_PIG RecName: Full=60S ribosomal protein L29 >gi|4586439|dbj|BAA76404.1| ribosomal protein L29/heparin/heparan sulfate interacting protein [Sus scrofa] >gi|4586433|dbj|BAA76401.1|r	0.323	0.44	0.287	0.306
gi|350591380|ref|XP_003358554.2|	PREDICTED: ADP-ribosylation factor-like protein 8B-like, partial [Sus scrofa]	1.719	1.334	---	1.377
gi|356582297|ref|NP_001239143.1|	ADP-ribosylation factor-like 8B [Sus scrofa]				

gi|194474046|ref|NP_001124006.1|	1-acyl-sn-glycerol-3-phosphate acyltransferase beta precursor [Sus scrofa] >gi|262036915|dbj|BAI47593.1| 1-acylglycerol-3-phosphate O-acyltransferase 2 [Sus scrofa] >gi|216408319|gb|ACJ72850.1| 1-acylglycerol-3-phosphate O-acyltransferase 2 [Sus scr	0.835	0.385	0.563	0.422
gi|311256096|ref|XP_003126504.1|	PREDICTED: epidermal growth factor receptor kinase substrate 8 [Sus scrofa]	1.005	1.468	1.663	1.18
gi|350578257|ref|XP_001927626.3|	PREDICTED: heterogeneous nuclear ribonucleoprotein Q isoform 1 [Sus scrofa]	---	---	---	---
gi|319401911|ref|NP_001188310.1|	adenosylhomocysteinase-like protein 1 [Sus scrofa] >gi|315321422|gb|ADU04838.1| adenosylhomocysteinase-like protein 1 [Sus scrofa]	---	---	---	---
gi|335293809|ref|XP_003129222.2|	PREDICTED: SWI/SNF-related matrix-associated actin-dependent regulator of chromatin subfamily A member 5 [Sus scrofa]	---	---	---	---
gi|21263966|sp|O97580.1|SUCB1_PIG	RecName: Full=Succinyl-CoA ligase [ADP-forming] subunit beta, mitochondrial; AltName: Full=ATP-specific succinyl-CoA synthetase subunit beta; AltName: Full=Succinyl-CoA synthetase beta-A chain; Short=SCS-betaA; Flags: Precursor >gi|3808237|gb|AAC697	1.603	0.886	0.685	1.279
gi|311266259|ref|XP_003131022.1|	PREDICTED: succinyl-CoA ligase [ADP-forming] subunit beta, mitochondrial [Sus scrofa]				
gi|350584784|ref|XP_003481823.1|	PREDICTED: acyl-CoA synthetase family member 3, mitochondrial-like [Sus scrofa]	---	---	---	---
gi|311272155|ref|XP_003133324.1|	PREDICTED: activated RNA polymerase II transcriptional coactivator p15-like [Sus scrofa]	1.272	0.887	1.028	0.92
gi|164664444|ref|NP_001106907.1|	alpha-2-macroglobulin receptor-associated protein precursor [Sus	0.444	0.486	0.435	0.456

	scrofa] >gi|162946612|gb|ABY21262.1| alpha-2-macroglobulin receptor-associated protein [Sus scrofa]				
gi|1709973|sp|P53027.3|RL10A_PIG	RecName: Full=60S ribosomal protein L10a	1.019	0.786	0.75	1.14
gi|350584352|ref|XP_001926213.3|	PREDICTED: putative deoxyribose-phosphate aldolase-like [Sus scrofa]	1.499	0.84	1.311	1.266
gi|345091011|ref|NP_001230734.1|	PCTP-like protein [Sus scrofa]	1.458	0.724	0.904	1.656
gi|335286933|ref|XP_003355223.1|	PREDICTED: 39S ribosomal protein L9, mitochondrial-like isoform 1 [Sus scrofa]	---	---	---	---
gi|335286935|ref|XP_003355224.1|	PREDICTED: 39S ribosomal protein L9, mitochondrial-like isoform 2 [Sus scrofa]				
gi|335286937|ref|XP_003355225.1|	PREDICTED: 39S ribosomal protein L9, mitochondrial-like isoform 3 [Sus scrofa]				
gi|335285324|ref|XP_003354827.1|	PREDICTED: prenylcysteine oxidase-like [Sus scrofa]	1.016	0.403	0.147	0.77
gi|350580104|ref|XP_003353925.2|	PREDICTED: mitochondrial carrier homolog 2 [Sus scrofa]	0.732	---	0.78	1.032
gi|335297415|ref|XP_003358038.1|	PREDICTED: 60S ribosomal protein L38-like isoform 2 [Sus scrofa] >gi|311266862|ref|XP_003131289.1| PREDICTED: 60S ribosomal protein L38-like isoform 1 [Sus scrofa]	0.659	0.354	0.29	0.677
gi|350589263|ref|XP_003130553.2|	PREDICTED: isoleucyl-tRNA synthetase, mitochondrial [Sus scrofa]	---	---	---	---
gi|350589265|ref|XP_003482824.1|	PREDICTED: isoleucyl-tRNA synthetase, mitochondrial-like [Sus scrofa]				
gi|311258593|ref|XP_003127690.1|	PREDICTED: adaptin ear-binding coat-associated protein 2-like [Sus scrofa]	---	---	---	---
gi|350590733|ref|XP_003131874.3|	PREDICTED: pre-mRNA-processing-splicing factor 8, partial [Sus scrofa]	1.245	0.745	0.327	1.182

gi|171465894|gb|ACB46190.1|	solute carrier family 3 member 2 [Sus scrofa]	1.19	1.317	1.335	1.619
gi|335281655|ref|XP_003353857.1|	PREDICTED: 4F2 cell-surface antigen heavy chain [Sus scrofa]				
gi|335290721|ref|XP_003356259.1|	PREDICTED: platelet-activating factor acetylhydrolase 2, cytoplasmic-like [Sus scrofa]	---	---	---	---
gi|350589961|ref|XP_003482962.1|	PREDICTED: LIM domain only protein 7-like [Sus scrofa]	---	---	---	---
gi|350594202|ref|XP_003133916.3|	PREDICTED: threonyl-tRNA synthetase, cytoplasmic-like [Sus scrofa]	1.321	1.138	1.101	1.8
gi|121118|sp|P20305.1|GELS_PIG	RecName: Full=Gelsolin; AltName: Full=Actin-depolymerizing factor; Short=ADF; AltName: Full=Brevin; Flags: Precursor >gi|164472|gb|AAA31042.1| plasma gelsolin precursor, partial [Sus scrofa] >gi|758306|emb|CAA32077.1| gelsolin [Sus scrofa]	0.313	0.654	0.571	0.497
gi|350587978|ref|XP_003129373.3|	PREDICTED: protein FAM13A-like [Sus scrofa]	0.659	0.866	0.618	0.781
gi|311260959|ref|XP_003128594.1|	PREDICTED: magnesium-dependent phosphatase 1-like [Sus scrofa]	---	---	---	---
gi|335308200|ref|XP_003361139.1|	PREDICTED: ADP-sugar pyrophosphatase-like [Sus scrofa]	0.954	0.904	0.3	0.59
gi|350589627|ref|XP_003482885.1|	PREDICTED: ADP-sugar pyrophosphatase-like isoform 2 [Sus scrofa] >gi|350589625|ref|XP_003482884.1| PREDICTED: ADP-sugar pyrophosphatase-like isoform 1 [Sus scrofa]				
gi|147832425|emb|CAN59677.1|	HLA-B associated transcript 3 [Sus scrofa]	0.548	0.744	0.786	0.679
gi|147832426|emb|CAN59678.1|	HLA-B associated transcript 3 [Sus scrofa]				
gi|162138209|gb|ABX82810.1|	HLA-B-associated protein 3 [Sus scrofa]				
gi|223950621|ref|NP_001138854.1|	large proline-rich protein BAG6 [Sus scrofa] >gi|317374853|sp|A5D9M6.1|BAG6_PIG RecName: Full=Large				

<table>
<tr><td></td><td>proline-rich protein BAG6; AltName: Full=BCL2-associated athanogene 6; AltName: Full=HLA-B-associated transcript 3 >gi|147832424|emb|CAN59676.1| HLA-</td><td></td><td></td><td></td><td></td></tr>
<tr><td>gi|350580973|ref|XP_003123871.3|</td><td>PREDICTED: alpha-mannosidase 2 [Sus scrofa]</td><td>1.133</td><td>0.658</td><td>0.917</td><td>0.809</td></tr>
<tr><td>gi|350583728|ref|XP_003126027.3|</td><td>PREDICTED: protein kinase C and casein kinase substrate in neurons protein 2-like [Sus scrofa]</td><td>---</td><td>---</td><td>---</td><td>---</td></tr>
<tr><td>gi|350588987|ref|XP_003130351.3|</td><td>PREDICTED: protein PRRC2C [Sus scrofa]</td><td>---</td><td>---</td><td>---</td><td>---</td></tr>
<tr><td>gi|359465576|ref|NP_001240753.1|</td><td>A-kinase anchor protein 9 [Sus scrofa]</td><td>0.772</td><td>1.137</td><td>0.982</td><td>0.663</td></tr>
<tr><td>gi|311254196|ref|XP_003125793.1|</td><td>PREDICTED: double-stranded RNA-specific adenosine deaminase, partial [Sus scrofa]</td><td>0.75</td><td>1.18</td><td>1.736</td><td>1.162</td></tr>
<tr><td>gi|311270046|ref|XP_003132745.1|</td><td>PREDICTED: 60S ribosomal protein L24-like isoform 1 [Sus scrofa]</td><td>0.42</td><td>0.371</td><td>0.263</td><td>0.418</td></tr>
<tr><td>gi|57527982|ref|NP_001009576.1|</td><td>radixin [Sus scrofa] >gi|131821|sp|P26044.1|RADI_PIG RecName: Full=Radixin; AltName: Full=Moesin-B >gi|164586|gb|AAB02865.1| moesin B [Sus scrofa]</td><td>---</td><td>---</td><td>---</td><td>---</td></tr>
<tr><td>gi|335292025|ref|XP_003128369.2|</td><td>PREDICTED: butyrophilin subfamily 1 member A1-like [Sus scrofa]</td><td>0.82</td><td>0.746</td><td>0.935</td><td>0.779</td></tr>
<tr><td>gi|350586526|ref|XP_003482206.1|</td><td>PREDICTED: butyrophilin-like protein 1-like [Sus scrofa]</td><td></td><td></td><td></td><td></td></tr>
<tr><td>gi|311248954|ref|XP_003123394.1|</td><td>PREDICTED: methylthioribose-1-phosphate isomerase [Sus scrofa]</td><td>1.08</td><td>0.841</td><td>0.697</td><td>1.123</td></tr>
<tr><td>gi|335290012|ref|XP_003356047.1|</td><td>PREDICTED: protein arginine N-methyltransferase 1 isoform 1 [Sus scrofa]</td><td>1.589</td><td>0.772</td><td>0.747</td><td>1.434</td></tr>
<tr><td>gi|335290016|ref|XP_003356049.1|</td><td>PREDICTED: protein arginine N-methyltransferase 1 isoform 3 [Sus scrofa]</td><td></td><td></td><td></td><td></td></tr>
<tr><td>gi|350581874|ref|XP_003124716.3|</td><td>PREDICTED: rab11 family-interacting protein 3-like [Sus scrofa]</td><td>0.509</td><td>0.444</td><td>0.527</td><td>0.474</td></tr>
</table>

gi|47523066|ref|NP_999296.1|	caspase-3 [Sus scrofa] >gi|54035961|sp|Q95ND5.1|CASP3_PIG RecName: Full=Caspase-3; Short=CASP-3; Contains: RecName: Full=Caspase-3 subunit p17; Contains: RecName: Full=Caspase-3 subunit p12; Flags: Precursor >gi|14090240|dbj|BAB55544.1| caspase-3 [S	1.672	1.439	1.284	1.702
gi|335290940|ref|XP_003356338.1|	PREDICTED: histone-binding protein RBBP4-like [Sus scrofa]	---	---	---	---
gi|346986460|ref|NP_001231381.1|	AP-2 complex subunit sigma isoform 1 [Sus scrofa]	---	---	---	---
gi|194038455|ref|XP_001928714.1|	PREDICTED: arginase-2, mitochondrial [Sus scrofa]	1.045	1.8	1.849	1.528
gi|350579579|ref|XP_001925536.4|	PREDICTED: 26S proteasome non-ATPase regulatory subunit 5 [Sus scrofa]	2.285	0.861	0.75	1.323
gi|346421378|ref|NP_001231061.1|	serpin H1 precursor [Sus scrofa]	0.667	0.74	0.49	0.32
gi|347543818|ref|NP_001231565.1|	cytochrome P450, family 4, subfamily F, polypeptide 55 precursor [Sus scrofa]	0.959	0.472	0.472	0.986
gi|349585046|ref|NP_001231798.1|	uncharacterized protein LOC100513647 [Sus scrofa]	1.129	1.102	1.159	1.01
gi|346421403|ref|NP_001231076.1|	translocon-associated protein subunit gamma [Sus scrofa]	---	---	---	---
gi|350580262|ref|XP_003123007.3|	PREDICTED: pleckstrin homology domain-containing family A member 7 [Sus scrofa]	0.79	0.737	0.979	0.755
gi|350596440|ref|XP_003484272.1|	PREDICTED: elongation factor Tu GTP-binding domain-containing protein 1 [Sus scrofa]	---	---	---	---
gi|335288532|ref|XP_001925819.3|	PREDICTED: ubiquitin carboxyl-terminal hydrolase 5 isoform 1 [Sus scrofa]	0.985	1.005	1.02	1.252
gi|335288534|ref|XP_003355644.1|	PREDICTED: ubiquitin carboxyl-terminal hydrolase 5 isoform 2 [Sus scrofa]				
gi|350593631|ref|XP_003359617.2|	PREDICTED: LOW QUALITY PROTEIN: alkyldihydroxyacetonephosphate	1.415	1.103	1.169	1.625

	synthase, peroxisomal [Sus scrofa]				
gi|311274849|ref|XP_001925742.2|	PREDICTED: regulation of nuclear pre-mRNA domain-containing protein 1B [Sus scrofa]	0.672	1.313	0.928	0.755
gi|311247401|ref|XP_003122628.1|	PREDICTED: peptidyl-prolyl cis-trans isomerase FKBP2-like isoform 1 [Sus scrofa]	0.659	0.769	0.771	0.931
gi|335281587|ref|XP_003353839.1|	PREDICTED: peptidyl-prolyl cis-trans isomerase FKBP2-like isoform 4 [Sus scrofa] >gi|335281583|ref|XP_003353837.1| PREDICTED: peptidyl-prolyl cis-trans isomerase FKBP2-like isoform 2 [Sus scrofa] >gi|335281585|ref|XP_003353838.1| PREDICTED: peptidyl				
gi|335308437|ref|XP_003361229.1|	PREDICTED: nodal modulator 1, partial [Sus scrofa]	---	---	---	---
gi|171986635|gb|ACB59309.1|	intelectin 2 [Sus scrofa]	1.352	0.631	0.358	0.858
gi|350583241|ref|XP_003481467.1|	PREDICTED: intelectin-2-like [Sus scrofa]				
gi|194042631|ref|XP_001925414.1|	PREDICTED: ATP-binding cassette sub-family B member 10, mitochondrial-like [Sus scrofa]	---	---	---	---
gi|346986378|ref|NP_001231341.1|	heme oxygenase 2 [Sus scrofa]	0.834	0.975	0.848	0.916
gi|335305501|ref|XP_003134862.2|	PREDICTED: E3 ubiquitin-protein ligase ZNRF2-like [Sus scrofa]	0.453	1.019	1.358	0.815
gi|164302|gb|AAA30983.1|	alpha-1 acid glycoprotein, partial [Sus scrofa]	0.263	0.696	0.494	0.699
gi|350579533|ref|XP_003480630.1|	PREDICTED: alpha-1-acid glycoprotein-like [Sus scrofa] >gi|194033903|ref|XP_001928762.1| PREDICTED: alpha-1-acid glycoprotein [Sus scrofa]				
gi|297747284|ref|NP_001172109.1|	prefoldin subunit 6 [Sus scrofa] >gi|297747282|ref|NP_001172108.1| prefoldin subunit 6 [Sus scrofa]	1.246	1.047	1.771	1.188
gi|343887362|ref|NP_001230582.1|	small nuclear ribonucleoprotein Sm D2 [Sus scrofa]	0.577	0.588	0.599	0.648

gi|335280302|ref|XP_003353540.1|	PREDICTED: actin-related protein 10-like [Sus scrofa]	---	---	---	---
gi|52001458|sp|P22411.3|DPP4_PIG	RecName: Full=Dipeptidyl peptidase 4; AltName: Full=Dipeptidyl peptidase IV; Short=DPP IV; AltName: Full=T-cell activation antigen CD26; AltName: CD_antigen=CD26; Contains: RecName: Full=Dipeptidyl peptidase 4 membrane form; AltName: Full=Dipeptidyl	0.986	1.192	1.335	1.094
gi|90108717|pdb|2AJC|D	Chain D, Porcine Dipeptidyl Peptidase Iv (Cd26) In Complex With 4-(2- Aminoethyl)-Benzene Sulphonyl Fluoride (Aebsf) >gi|90108715|pdb|2AJC|B Chain B, Porcine Dipeptidyl Peptidase Iv (Cd26) In Complex With 4-(2- Aminoethyl)-Benzene Sulphonyl Fluoride				
gi|350588982|ref|XP_003130335.2|	PREDICTED: thiamin pyrophosphokinase 1-like [Sus scrofa]	---	---	---	---
gi|194035181|ref|XP_001925325.1|	PREDICTED: sphingomyelin phosphodiesterase 2 [Sus scrofa]	---	---	---	---
gi|350593416|ref|XP_003483680.1|	PREDICTED: BAG family molecular chaperone regulator 4-like [Sus scrofa]	0.782	1.77	1.183	0.995
gi|311274558|ref|XP_003134376.1|	PREDICTED: vacuolar protein sorting-associated protein 16 homolog isoform 1 [Sus scrofa]				
gi|311274560|ref|XP_003134377.1|	PREDICTED: vacuolar protein sorting-associated protein 16 homolog isoform 2 [Sus scrofa]				
gi|297632389|ref|NP_001172080.1|	mitochondrial ribosomal protein L52 [Sus scrofa]	0.14	0.446	0.448	0.294
gi|347658999|ref|NP_001231625.1|	uncharacterized protein LOC100512671 [Sus scrofa]	0.634	1.694	2.208	1.089
gi|350588410|ref|XP_003482644.1|	PREDICTED: ester hydrolase C11orf54 homolog [Sus scrofa]				
gi|350582160|ref|XP_003124976.3|	PREDICTED: threonine synthase-like 2-like [Sus scrofa]	---	---	---	---
gi|148234110|ref|NP_001087	selenide, water dikinase 2 [Sus	---	---	---	---

204.1|	scrofa] >gi|171769747|sp|A1YIZ1.2|SPS2_PIG RecName: Full=Selenide, water dikinase 2; AltName: Full=Selenium donor protein 2; AltName: Full=Selenophosphate synthase 2 >gi|120988510|gb|ABM46855.1| selenophosphate synthe				
gi|343432623|ref|NP_001230325.1|	bolA-like protein 1 [Sus scrofa]	1.648	1.679	1.528	1.58
gi|335284508|ref|XP_003124605.2|	PREDICTED: mu-crystallin homolog [Sus scrofa]	---	---	---	---
gi|178057067|ref|NP_001116568.1|	hsp90 co-chaperone Cdc37 [Sus scrofa] >gi|51870491|emb|CAG15149.1| CDC37 cell division cycle 37 protein [Sus scrofa]	0.935	0.666	0.807	0.831
gi|297591959|ref|NP_001172060.1|	farnesyl pyrophosphate synthase precursor [Sus scrofa] >gi|262072800|dbj|BAI47708.1| farnesyl diphosphate synthase [Sus scrofa]	---	---	---	---
gi|2498644|sp|Q29075.1|NKL_PIG	RecName: Full=Antimicrobial peptide NK-lysin; Short=NKL; Flags: Precursor >gi|736317|emb|CAA59720.1| NK-lysin [Sus scrofa]	---	---	---	---
gi|311252221|ref|XP_003124987.1|	PREDICTED: antimicrobial peptide NK-lysin [Sus scrofa]				
gi|1352666|sp|P11493.2|PP2AB_PIG	RecName: Full=Serine/threonine-protein phosphatase 2A catalytic subunit beta isoform; Short=PP2A-beta >gi|164298|gb|AAA30982.1| protein phosphatase 2A beta subunit, partial [Sus scrofa]	2.065	1.224	0.936	1.719
gi|47523790|ref|NP_999531.1|	serine/threonine-protein phosphatase 2A catalytic subunit alpha isoform [Sus scrofa] >gi|54038797|sp|P67776.1|PP2AA_PIG RecName: Full=Serine/threonine-protein phosphatase 2A catalytic subunit alpha isoform; Short=PP2A-alpha >gi|164296|gb|AA				

	A30981.1|				
gi|311272409|ref|XP_003133430.1|	PREDICTED: serine/threonine-protein phosphatase 2A catalytic subunit alpha isoform [Sus scrofa]				
gi|194034423|ref|XP_001926853.1|	PREDICTED: l-2-hydroxyglutarate dehydrogenase, mitochondrial [Sus scrofa]	0.722	0.257	0.397	0.518
gi|347658980|ref|NP_001231616.1|	ribosomal protein L36 [Sus scrofa] >gi|350580660|ref|XP_003480870.1| PREDICTED: 60S ribosomal protein L36 isoform 3 [Sus scrofa] >gi|350580656|ref|XP_003480868.1| PREDICTED: 60S ribosomal protein L36 isoform 1 [Sus scrofa] >gi|350580658|ref|XP_00348	0.476	0.379	0.238	0.385
gi|350581899|ref|XP_003124791.3|	PREDICTED: LOW QUALITY PROTEIN: putative N-acetylglucosamine-6-phosphate deacetylase-like [Sus scrofa]	---	---	---	---
gi|60097959|ref|NP_001012408.1|	dystrophin [Sus scrofa] >gi|75052798|sp|Q5GN48.1|DMD_PIG RecName: Full=Dystrophin >gi|58416122|emb|CAI26302.1| dystrophin [Sus scrofa]	1.086	0.933	1.591	0.858
gi|325652134|ref|NP_001191701.1|	bromodomain-containing protein 4 [Sus scrofa] >gi|321172828|gb|ADW77216.1| bromodomain-containing protein 4 [Sus scrofa]	0.575	1.145	0.883	0.932
gi|347300394|ref|NP_001231489.1|	vesicle-fusing ATPase [Sus scrofa]	0.525	0.58	0.503	0.645
gi|350596345|ref|XP_003484261.1|	PREDICTED: vesicle-fusing ATPase-like [Sus scrofa]				
gi|350594565|ref|XP_003134235.3|	PREDICTED: acid ceramidase-like [Sus scrofa]	0.498	1.252	1.005	0.73
gi|160420271|ref|NP_001038047.1|	zinc finger Ran-binding domain-containing protein 2 [Sus scrofa] >gi|118578026|sp|Q19QU3.1|ZRAB2_PIG RecName: Full=Zinc finger Ran-binding domain-containing protein 2; AltName: Full=Zinc finger protein	0.664	1.344	0.97	0.958

	265 >gi|104295127|gb|ABF72033.1| zinc finger pr				
gi|335306763|ref|XP_003360560.1|	PREDICTED: ubiquitin-like protein 4A-like isoform 1 [Sus scrofa]	0.8	0.778	0.894	0.843
gi|335306765|ref|XP_003360561.1|	PREDICTED: ubiquitin-like protein 4A-like isoform 2 [Sus scrofa]				
gi|311255898|ref|XP_003126418.1|	PREDICTED: LOW QUALITY PROTEIN: nuclear pore complex protein Nup107-like [Sus scrofa]	---	---	---	---
gi|335310331|ref|XP_003361983.1|	PREDICTED: poly(U)-binding-splicing factor PUF60-like [Sus scrofa]	1.293	1.065	0.792	1.76
gi|178056703|ref|NP_001116542.1|	angiotensin-converting enzyme 2 precursor [Sus scrofa] >gi|170066580|gb|ACB06678.1| angiotensin converting enzyme 2 [Sus scrofa]	0.613	0.88	0.945	0.81
gi|254546340|gb|ACT66265.1|	angiotensin I converting enzyme 2 [Sus scrofa domesticus]				
gi|335310935|ref|XP_003362258.1|	PREDICTED: NADH dehydrogenase [ubiquinone] 1 beta subcomplex subunit 10-like [Sus scrofa]	0.74	0.563	0.475	0.74
gi|346644697|ref|NP_001231015.1|	eukaryotic translation initiation factor 3 subunit L [Sus scrofa]	2.308	1.055	1.577	1.845
gi|194041975|ref|XP_001929465.1|	PREDICTED: STE20-like kinase isoform 2 [Sus scrofa]	---	---	---	---
gi|346227222|ref|NP_001230997.1|	ribosomal protein L13a isoform 1 [Sus scrofa]	0.802	0.636	0.5	0.943
gi|346227224|ref|NP_001230998.1|	ribosomal protein L13a isoform 2 [Sus scrofa]				
gi|58618083|gb|AAW80612.1|	P2X7 receptor [Sus scrofa]	0.605	0.439	0.663	0.308
gi|311270616|ref|XP_001926839.2|	PREDICTED: P2X purinoceptor 7 [Sus scrofa]				
gi|58826311|gb|AAW82880.1|	P2X7 purinergic receptor [Sus scrofa]				
gi|122064241|sp|Q06AT0.3|HPCL1_PIG	RecName: Full=Hippocalcin-like protein 1 >gi|178056468|ref|NP_001116598.1| hippocalcin-like protein 1 [Sus scrofa] >gi|115394792|gb|ABI97190.1| HPCL1 [Sus scrofa]	1.122	1.907	2.156	1.642

gi|343478230|ref|NP_001230382.1|	enhancer of yellow 2 transcription factor homolog isoform 1 [Sus scrofa] >gi|350582977|ref|XP_003481405.1| PREDICTED: enhancer of yellow 2 transcription factor homolog isoform 1 [Sus scrofa]	---	---	---	---
gi|343478234|ref|NP_001230383.1|	enhancer of yellow 2 transcription factor homolog isoform 2 [Sus scrofa] >gi|350582979|ref|XP_003481406.1| PREDICTED: enhancer of yellow 2 transcription factor homolog isoform 2 [Sus scrofa]				
gi|585657|sp|P37109.1|ISK4_PIG	RecName: Full=Serine protease inhibitor Kazal-type 4; AltName: Full=Peptide PEC-60; Flags: Precursor >gi|47523134|ref|NP_999029.1| serine protease inhibitor Kazal-type 4 precursor [Sus scrofa] >gi|5705936|gb|AAB23691.2| PEC-60 [Sus scrofa] >gi|2034|	---	---	---	---
gi|159162822|pdb|1PCE|A	Chain A, Solution Structure And Dynamics Of Pec-60, A Protein Of The Kazal Type Inhibitor Family, Determined By Nuclear Magnetic Resonance Spectroscopy				
gi|350595064|ref|XP_003134565.3|	PREDICTED: dnaJ homolog subfamily B member 6-like [Sus scrofa]	1.018	1.082	1.298	1.144
gi|54039304|sp|P62279.2|RS13_PIG	RecName: Full=40S ribosomal protein S13	0.681	0.401	0.457	0.918
gi|348605266|ref|NP_001231758.1|	ribosomal protein S13 [Sus scrofa]				
gi|164525|gb|AAA31059.1|	lactoferrin [Sus scrofa]	---	1.107	---	0.8
gi|17467354|gb|AAL40161.1|L77887_1	lactoferrin, partial [Sus scrofa]				
gi|3915882|sp|P14632.3|TRFL_PIG	RecName: Full=Lactotransferrin; Short=Lactoferrin; Flags: Precursor				
gi|47523782|ref|NP_999527.1|	lactotransferrin precursor [Sus scrofa] >gi|164614|gb|AAA31102.1| lactoferrin [Sus scrofa]				
gi|116488296|gb|ABJ98718.1|	lactoferrin [Sus scrofa]				
gi|41688298|dbj|BAD08651.1	lactotransferrin [Sus scrofa]				

|					
gi|32130549|gb|AAP70487.1|	lactoferrin [Sus scrofa]				
gi|194043274|ref|XP_001927288.1|	PREDICTED: developmentally-regulated GTP-binding protein 1 isoform 1 [Sus scrofa]	---	---	---	---
gi|311257796|ref|XP_003127291.1|	PREDICTED: SUMO-activating enzyme subunit 1-like isoform 1 [Sus scrofa]	---	---	---	---
gi|335289862|ref|XP_003356004.1|	PREDICTED: SUMO-activating enzyme subunit 1-like isoform 2 [Sus scrofa]				
gi|2258465|gb|AAB94003.1|	succinyl-CoA synthetase alpha subunit [Sus scrofa]	0.61	0.582	0.448	0.616
gi|12621910|gb|AAB94002.2|	succinyl-CoA synthetase [Sus scrofa]				
gi|9955019|pdb|1EUC|A	Chain A, Crystal Structure Of Dephosphorylated Pig Heart, Gtp-Specific Succinyl-Coa Synthetase				
gi|90109582|pdb|2FPP|A	Chain A, Crystal Structure Of Pig Gtp-Specific Succinyl-Coa Synthetase From Polyethylene Glycol With Chloride Ions >gi|90109574|pdb|2FPI|A Chain A, Crystal Structure Of Pig Gtp-Specific Succinyl-Coa Synthetase From Polyethylene Glycol >gi|90109554|p				
gi|90109572|pdb|2FPG|A	Chain A, Crystal Structure Of Pig Gtp-Specific Succinyl-Coa Synthetase In Complex With Gdp				
gi|223634702|sp|O19069.2|SUCA_PIG	RecName: Full=Succinyl-CoA ligase [ADP/GDP-forming] subunit alpha, mitochondrial; AltName: Full=Succinyl-CoA synthetase subunit alpha; Short=SCS-alpha; Flags: Precursor				
gi|9955021|pdb|1EUD|A	Chain A, Crystal Structure Of Phosphorylated Pig Heart, Gtp-Specific Succinyl-Coa Synthetase				
gi|311263942|ref|XP_003129926.1|	PREDICTED: oligoribonuclease, mitochondrial-like [Sus scrofa]	---	---	---	---

gi|194035009|ref|XP_001925846.1|	PREDICTED: ras-related protein Rab-27B-like [Sus scrofa]	0.921	0.767	0.639	0.911
gi|17933431|gb|AAL48289.1|AF452448_1	beta-2-microglobulin protein [Sus scrofa]	0.551	1.527	1.177	1.363
gi|47522782|ref|NP_999143.1|	beta-2-microglobulin precursor [Sus scrofa] >gi|1168611|sp|Q07717.1|B2MG_PIG RecName: Full=Beta-2-microglobulin; AltName: Full=Lactollin; Flags: Precursor >gi|350578710|ref|XP_003480435.1| PREDICTED: beta-2-microglobulin-like [Sus scrofa] >gi|350578				
gi|365813031|pdb|3QQ3|E	Chain E, Crystal Structure Of Swine Major Histocompatibility Complex Class I Sla-1 0401 And Identification Of 2009 Pandemic Swine-Origin Influenza A H1n1 Virus Cytotoxic T Lymphocyte Epitope Peptides >gi|365813034|pdb|3QQ4|B Chain B, Crystal Structu				
gi|77176666|gb|ABA64451.1|	beta-2-microglobulin protein, partial [Sus scrofa]				
gi|350578712|ref|XP_003480436.1|	PREDICTED: beta-2-microglobulin-like [Sus scrofa]				
gi|311248023|ref|XP_003122934.1|	PREDICTED: eukaryotic translation initiation factor 3 subunit M [Sus scrofa]	1.029	0.97	1.863	1.341
gi|346986292|ref|NP_001231303.1|	uncharacterized protein LOC100155717 isoform 1 [Sus scrofa] >gi|346986290|ref|NP_001231302.1| uncharacterized protein LOC100155717 isoform 1 [Sus scrofa]	1.313	0.813	0.747	0.706
gi|346986294|ref|NP_001231304.1|	uncharacterized protein LOC100155717 isoform 2 [Sus scrofa]				
gi|350589017|ref|XP_003482769.1|	PREDICTED: 28S ribosomal protein S14, mitochondrial-like [Sus scrofa]	---	---	---	---
gi|45268975|gb|AAS55900.1|	60S ribosomal protein L11, partial [Sus scrofa]	2.079	1.202	2.07	1.478
gi|51704216|sp|Q29205.3|RL	RecName: Full=60S ribosomal				

11_PIG	protein L11 >gi|48675939|ref|NP_001001638.1| 60S ribosomal protein L11 [Sus scrofa] >gi|45239006|gb|AAS55632.1| ribosomal protein L11 [Sus scrofa]				
gi|433126|gb|AAA52218.1|	Ig gamma 2b chain constant region, partial [Sus scrofa] >gi|2136514|pir||I47160 Ig gamma 2b chain constant region - pig (fragment)	---	---	---	---
gi|350591483|ref|XP_003358588.2|	PREDICTED: acyl-CoA dehydrogenase family member 9, mitochondrial-like [Sus scrofa]	---	---	---	---
gi|311253264|ref|XP_001925395.2|	PREDICTED: zinc transporter ZIP4-like [Sus scrofa]	---	---	---	---
gi|311260258|ref|XP_003128394.1|	PREDICTED: LEM domain-containing protein 2 [Sus scrofa]	---	---	---	---
gi|335288032|ref|XP_001929448.3|	PREDICTED: prostaglandin E synthase 3 isoform 1 [Sus scrofa]	---	---	---	---
gi|335288038|ref|XP_003355506.1|	PREDICTED: prostaglandin E synthase 3 isoform 4 [Sus scrofa]				
gi|335288034|ref|XP_003355504.1|	PREDICTED: prostaglandin E synthase 3 isoform 2 [Sus scrofa]				
gi|47522718|ref|NP_999109.1|	dolichyl-diphosphooligosaccharide--protein glycosyltransferase subunit DAD1 [Sus scrofa] >gi|3023621|sp|Q29036.3|DAD1_PIG RecName: Full=Dolichyl-diphosphooligosaccharide--protein glycosyltransferase subunit DAD1; Short=Oligosaccharyl transferase sub	0.875	0.615	0.56	0.973
gi|350585844|ref|XP_003356353.2|	PREDICTED: histone deacetylase 1-like, partial [Sus scrofa]	---	---	---	---
gi|350585862|ref|XP_003127820.3|	PREDICTED: histone deacetylase 1-like [Sus scrofa]				
gi|311247551|ref|XP_003122699.1|	PREDICTED: DNA damage-binding protein 1-like isoform 1 [Sus scrofa]	1.254	0.422	0.742	0.802
gi|335281341|ref|XP_003122446.2|	PREDICTED: mitochondrial glutamate carrier 1-like [Sus scrofa]	---	---	---	---
gi|194042439|ref|XP_001928	PREDICTED: ATPase family AAA	0.481	1.189	1.064	0.8

012.1|	domain-containing protein 1 isoform 1 [Sus scrofa]				
gi|335301975|ref|XP_003359338.1|	PREDICTED: ATPase family AAA domain-containing protein 1 isoform 2 [Sus scrofa]				
gi|194033654|ref|XP_001928369.1|	PREDICTED: ubiquinone biosynthesis protein COQ4 homolog, mitochondrial [Sus scrofa]	---	---	---	---
gi|47117721|sp|P83884.2|RL36A_PIG	RecName: Full=60S ribosomal protein L36a; AltName: Full=60S ribosomal protein L44 >gi|47522650|ref|NP_999082.1| 60S ribosomal protein L36a [Sus scrofa] >gi|335303998|ref|XP_003134062.2| PREDICTED: 60S ribosomal protein L36a-like [Sus scrofa] >gi|335	1.132	0.672	0.743	1.439
gi|194035409|ref|XP_001926511.1|	PREDICTED: syntaxin-7 [Sus scrofa]	0.461	0.897	0.68	0.715
gi|365796119|dbj|BAL43002.1|	toll-interacting protein [Sus scrofa]	0.769	1.115	1.202	0.594
gi|194033405|ref|XP_001928380.1|	PREDICTED: acetyl-CoA acetyltransferase, cytosolic [Sus scrofa]	0.884	0.927	0.657	0.802
gi|311248940|ref|XP_003123387.1|	PREDICTED: growth arrest and DNA damage-inducible proteins-interacting protein 1-like [Sus scrofa]	0.625	1.146	1.178	0.724
gi|335289143|ref|XP_003126912.2|	PREDICTED: probable D-lactate dehydrogenase, mitochondrial [Sus scrofa]	1.998	1.083	2.112	1.394
gi|311246098|ref|XP_003122082.1|	PREDICTED: coronin-2A-like [Sus scrofa]	0.864	0.93	0.766	1.106
gi|178056567|ref|NP_001116632.1|	prefoldin subunit 4 [Sus scrofa] >gi|147225098|emb|CAN13185.1| prefoldin 4 [Sus scrofa]	1.299	2.181	1.298	1.388
gi|148232463|ref|NP_001090940.1|	methylmalonyl-CoA epimerase, mitochondrial [Sus scrofa] >gi|117660706|gb|ABK55633.1| MCEE [Sus scrofa]	---	---	---	---
gi|359807141|ref|NP_001240850.1|	mannose-6-phosphate isomerase [Sus scrofa]	---	---	---	---
gi|350536495|ref|NP_001233	probable ATP-dependent RNA	1.051	1.216	1.219	1.395

197.1|	helicase DDX46 [Sus scrofa] >gi|336092215|gb|AEI00731.1| DEAD box polypeptide 46 [Sus scrofa]				
gi|298228972|ref|NP_001177201.1|	autophagy-related protein 16-1 isoform 1 [Sus scrofa] >gi|296874478|gb|ADH81744.1| autophagy related 16-like protein 1 transcript variant 1 [Sus scrofa]	---	---	---	---
gi|298231102|ref|NP_001177202.1|	autophagy-related protein 16-1 isoform 2 [Sus scrofa] >gi|296874480|gb|ADH81745.1| autophagy related 16-like protein 1 transcript variant 2 [Sus scrofa]				
gi|343403779|ref|NP_001230274.1|	ribosomal protein L13 [Sus scrofa]	1.041	0.385	0.387	1.096
gi|335298292|ref|XP_003358241.1|	PREDICTED: phosphatidylinositol transfer protein alpha isoform-like [Sus scrofa]	---	---	---	---
gi|350579520|ref|XP_003353667.2|	PREDICTED: U4/U6 small nuclear ribonucleoprotein Prp4-like [Sus scrofa]	1.08	0.968	1.519	0.658
gi|350582982|ref|XP_003481407.1|	PREDICTED: tetratricopeptide repeat protein 35-like [Sus scrofa]	---	---	---	---
gi|311252708|ref|XP_003125227.1|	PREDICTED: LOW QUALITY PROTEIN: protein phosphatase 1B-like [Sus scrofa]	1.755	0.953	0.884	0.89
gi|343790912|ref|NP_001230514.1|	ras-related C3 botulinum toxin substrate 1 (rho family, small GTP binding protein Rac1) [Sus scrofa]	---	---	---	---
gi|213021237|ref|NP_001132943.1|	DNA-(apurinic or apyrimidinic site) lyase [Sus scrofa] >gi|210062866|gb|ACJ06403.1| APEX nuclease 1 [Sus scrofa]	1.59	0.705	0.937	0.827
gi|47523060|ref|NP_999293.1|	serine/threonine-protein phosphatase 2B catalytic subunit alpha isoform [Sus scrofa] >gi|14209665|gb|AAK56844.1|AF193515_1 calcineurin catalytic subunit delta isoform [Sus scrofa]	---	---	---	---
gi|311250082|ref|XP_003123949.1|	PREDICTED: isochorismatase domain-containing protein 1-like [Sus scrofa]	0.95	0.901	1.01	0.792

gi|350529419|ref|NP_001231941.1|	inositol polyphosphate-1-phosphatase [Sus scrofa]	---	---	---	---
gi|350593719|ref|XP_003483752.1|	PREDICTED: inositol polyphosphate 1-phosphatase-like, partial [Sus scrofa]				
gi|347543777|ref|NP_001231552.1|	protein LYRIC [Sus scrofa]	0.777	1.226	1.046	1.089
gi|350590001|ref|XP_003357925.2|	PREDICTED: multidrug resistance-associated protein 4-like, partial [Sus scrofa]	0.814	0.461	0.545	0.631
gi|350580632|ref|XP_003123159.3|	PREDICTED: general transcription factor IIF subunit 1-like [Sus scrofa]	---	---	---	---
gi|350585533|ref|XP_003356179.2|	PREDICTED: leucine-rich repeat-containing protein 47-like [Sus scrofa]	---	0.923	1.23	0.852
gi|335290197|ref|XP_003356098.1|	PREDICTED: LOW QUALITY PROTEIN: 40S ribosomal protein S9-like, partial [Sus scrofa]	0.594	0.672	0.735	0.801
gi|311273092|ref|XP_001925601.2|	PREDICTED: protein FAM134A [Sus scrofa]	---	---	---	---
gi|335293289|ref|XP_003356924.1|	PREDICTED: O-phosphoseryl-tRNA(Sec) selenium transferase-like [Sus scrofa]	---	---	---	---
gi|350596387|ref|XP_003361109.2|	PREDICTED: O-phosphoseryl-tRNA(Sec) selenium transferase-like [Sus scrofa]				
gi|47523704|ref|NP_999484.1|	double stranded RNA-dependent protein kinase [Sus scrofa] >gi|29292936|dbj|BAC66439.1| double stranded RNA-dependent protein kinase [Sus scrofa]	0.76	1.035	1.042	1.077
gi|311252821|ref|XP_003125284.1|	PREDICTED: interferon-induced, double-stranded RNA-activated protein kinase-like [Sus scrofa]				
gi|219521982|ref|NP_001137178.1|	erlin-2 precursor [Sus scrofa] >gi|217314887|gb|ACK36978.1| ER lipid raft-associated 2 isoform 2 [Sus scrofa]	---	---	---	---
gi|239504580|ref|NP_001155120.1|	selenoprotein M precursor [Sus scrofa] >gi|237930353|gb|ACR33808.1| selenoprotein M [Sus scrofa]	---	---	---	---
gi|311257625|ref|XP_003127	PREDICTED: platelet-activating	---	---	---	---

213.1|	factor acetylhydrolase IB subunit gamma-like [Sus scrofa] >gi|311257641|ref|XP_003127220.1| PREDICTED: platelet-activating factor acetylhydrolase IB subunit gamma-like [Sus scrofa]				
gi|350592333|ref|XP_003359097.2|	PREDICTED: mitochondrial peptide methionine sulfoxide reductase [Sus scrofa]	---	---	---	---
gi|335295539|ref|XP_003130253.2|	PREDICTED: multidrug resistance protein 1 isoform 2 [Sus scrofa]	---	---	---	---
gi|350588870|ref|XP_003130261.3|	PREDICTED: multidrug resistance protein 1-like, partial [Sus scrofa]				
gi|350582950|ref|XP_003481396.1|	PREDICTED: double-strand-break repair protein rad21 homolog [Sus scrofa]	0.699	0.672	1.136	0.806
gi|350597023|ref|XP_003361918.2|	PREDICTED: double-strand-break repair protein rad21 homolog [Sus scrofa]				
gi|93359412|gb|ABF13285.1|	proliferating cell nuclear antigen [Sus scrofa]	---	---	---	---
gi|335304393|ref|XP_003359931.1|	PREDICTED: proliferating cell nuclear antigen [Sus scrofa]				
gi|60389430|sp|Q8MIZ3.1|DCPS_PIG	RecName: Full=m7GpppX diphosphatase; AltName: Full=DCS-1; AltName: Full=Hint-related 7meGMP-directed hydrolase; AltName: Full=Histidine triad protein member 5; Short=HINT-5; AltName: Full=Scavenger mRNA-decapping enzyme DcpS >gi|47523260|ref|NP_9989	---	---	---	---
gi|194042142|ref|XP_001929035.1|	PREDICTED: BAG family molecular chaperone regulator 3 [Sus scrofa]	0.803	0.732	0.843	0.588
gi|326632513|gb|ADZ99177.1|	BCL2-associated athanogene 3, partial [Sus scrofa]				
gi|335282832|ref|XP_003354166.1|	PREDICTED: protein Wiz isoform 2 [Sus scrofa]	---	---	---	---
gi|335282838|ref|XP_003354168.1|	PREDICTED: protein Wiz isoform 4 [Sus scrofa]				

gi|335282836|ref|XP_003123504.2|	PREDICTED: protein Wiz isoform 1 [Sus scrofa]				
gi|335282840|ref|XP_003354169.1|	PREDICTED: protein Wiz isoform 5 [Sus scrofa]				
gi|350595384|ref|XP_003134854.3|	PREDICTED: glycyl-tRNA synthetase-like [Sus scrofa]	---	---	---	---
gi|350585317|ref|XP_003127269.3|	PREDICTED: poliovirus receptor-like [Sus scrofa]	---	---	---	---
gi|349732213|ref|NP_001231857.1|	LSM3 homolog, U6 small nuclear RNA associated [Sus scrofa]	1.066	1.554	1.348	1.109
gi|350582471|ref|XP_003125244.3|	PREDICTED: echinoderm microtubule associated protein like 4 [Sus scrofa]	0.952	1.303	1.407	1.152
gi|350593526|ref|XP_003359584.2|	PREDICTED: 26S proteasome non-ATPase regulatory subunit 14 [Sus scrofa]	---	---	---	---
gi|51338616|sp|P62831.1|RL23_PIG	RecName: Full=60S ribosomal protein L23; AltName: Full=Ribosomal protein L17 >gi|194018720|ref|NP_001123427.1| 60S ribosomal protein L23 [Sus scrofa] >gi|117661026|gb|ABK55650.1| RPL23 [Sus scrofa]	0.6	0.498	0.522	0.903
gi|350589072|ref|XP_003357606.2|	PREDICTED: sulfhydryl oxidase 1-like isoform 1 [Sus scrofa]	---	---	---	---
gi|350589074|ref|XP_003357607.2|	PREDICTED: sulfhydryl oxidase 1-like isoform 2 [Sus scrofa]				
gi|335304552|ref|XP_001927148.2|	PREDICTED: acetyl-coenzyme A synthetase 2-like, mitochondrial-like [Sus scrofa]	1.29	0.748	0.638	1.105
gi|285818412|gb|ADC38879.1|	periostin, osteoblast specific factor [Sus scrofa]	0.836	1.196		1.034
gi|329744611|ref|NP_001193280.1|	periostin, osteoblast specific factor isoform 2 precursor [Sus scrofa]				
gi|329744601|ref|NP_001193276.1|	periostin, osteoblast specific factor isoform 1 precursor [Sus scrofa]				
gi|350579947|ref|XP_003122565.3|	PREDICTED: U4/U6.U5 tri-snRNP-associated protein 1 [Sus scrofa]	---	---	---	---
gi|194037097|ref|XP_001927840.1|	PREDICTED: carbonic anhydrase 2 [Sus scrofa]	0.798	1.38	1.495	0.798
gi|350582678|ref|XP_003354	PREDICTED: HCLS1-binding	---	---	---	---

961.2|	protein 3-like [Sus scrofa]				
gi|350590394|ref|XP_003131590.3|	PREDICTED: pyridoxine-5~-phosphate oxidase-like [Sus scrofa]	1.599	2.451	2.121	1.531
gi|335285134|ref|XP_003354782.1|	PREDICTED: charged multivesicular body protein 3-like isoform 2 [Sus scrofa]	0.402	0.867	0.704	0.463
gi|335285138|ref|XP_003125011.2|	PREDICTED: charged multivesicular body protein 3-like isoform 1 [Sus scrofa]				
gi|335285136|ref|XP_003354783.1|	PREDICTED: charged multivesicular body protein 3-like isoform 3 [Sus scrofa]				
gi|335290686|ref|XP_003356247.1|	PREDICTED: tissue alpha-L-fucosidase-like [Sus scrofa]	1.253	0.498	0.564	0.97
gi|336455302|gb|AEI59128.1|	fucosidase alpha-L-1 [Sus scrofa]				
gi|347543811|ref|NP_001231562.1|	cytochrome P450, family 2, subfamily J, polypeptide 34 [Sus scrofa]	0.682	0.669	0.73	0.888
gi|194041937|ref|XP_001928912.1|	PREDICTED: alpha-centractin [Sus scrofa]	1.787	1.406	1.284	1.802
gi|335286356|ref|XP_001927203.3|	PREDICTED: copine-3 [Sus scrofa]	0.525	0.444	0.348	0.456
gi|346716148|ref|NP_001231230.1|	importin-5 [Sus scrofa]	1.188	1.243	1.296	1.211
gi|311271339|ref|XP_003133112.1|	PREDICTED: sphingosine-1-phosphate lyase 1 isoform 2 [Sus scrofa]	0.583	0.569	0.74	0.661
gi|311271341|ref|XP_003133111.1|	PREDICTED: sphingosine-1-phosphate lyase 1 isoform 1 [Sus scrofa]				
gi|345091002|ref|NP_001230732.1|	nuclear distribution gene C homolog [Sus scrofa]	---	---	---	---
gi|194042094|ref|XP_001925670.1|	PREDICTED: shootin-1-like isoform 1 [Sus scrofa]	0.813	0.553	0.594	0.553
gi|113205762|ref|NP_001038043.1|	granulins precursor [Sus scrofa] >gi|55247591|gb|AAV48633.1| granulin precursor [Sus scrofa]	1.234	1.344	1.056	0.679
gi|350585670|ref|XP_003127678.3|	PREDICTED: filamin-binding LIM protein 1-like isoform 1 [Sus scrofa]	---	---	---	---
gi|350585672|ref|XP_003482022.1|	PREDICTED: filamin-binding LIM protein 1-like isoform 2 [Sus scrofa]				

gi|350594224|ref|XP_003359804.2|	PREDICTED: LOW QUALITY PROTEIN: nipped-B-like protein [Sus scrofa]	---	---	---	---
gi|335302969|ref|XP_001925494.2|	PREDICTED: Golgi reassembly-stacking protein 2 isoform 1 [Sus scrofa]	1.023	1.281	0.907	1.224
gi|335302971|ref|XP_003359599.1|	PREDICTED: Golgi reassembly-stacking protein 2 isoform 2 [Sus scrofa]				
gi|311258558|ref|XP_003127668.1|	PREDICTED: protein DDI1 homolog 2 [Sus scrofa]	---	---	---	---
gi|346716195|ref|NP_001231246.1|	nucleosome assembly protein 1-like 1 [Sus scrofa] >gi|346716193|ref|NP_001231245.1| nucleosome assembly protein 1-like 1 [Sus scrofa]	---	---	---	---
gi|335310655|ref|XP_003362133.1|	PREDICTED: hypothetical protein LOC100621842 [Sus scrofa]	---	---	---	---
gi|335283905|ref|XP_003354455.1|	PREDICTED: BET1-like protein-like isoform 2 [Sus scrofa] >gi|311250744|ref|XP_003124272.1| PREDICTED: BET1-like protein-like isoform 1 [Sus scrofa]	---	---	---	---
gi|178056852|ref|NP_001116541.1|	ATP-binding cassette sub-family F member 1 [Sus scrofa] >gi|56404330|sp|Q767L0.1|ABCF1_PIG RecName: Full=ATP-binding cassette sub-family F member 1 >gi|41529180|dbj|BAD08439.1| ATP-binding cassette, sub-family F (GCN20), member 1 [Sus scrofa]	---	---	---	---
gi|350579945|ref|XP_003480721.1|	PREDICTED: barrier-to-autointegration factor-like [Sus scrofa] >gi|335281491|ref|XP_003353819.1| PREDICTED: barrier-to-autointegration factor-like [Sus scrofa] >gi|335281487|ref|XP_003353817.1| PREDICTED: barrier-to-autointegration factor-like [Sus	0.942	1.351	1.123	0.909

gi|335298830|ref|XP_001925616.3|	PREDICTED: golgin subfamily A member 4 [Sus scrofa]	0.735	1.062	1.363	0.765
gi|347300222|ref|NP_001231422.1|	basic leucine zipper and W2 domain-containing protein 1 [Sus scrofa]	---	---	---	---
gi|350590801|ref|XP_003483139.1|	PREDICTED: phospholipase D2-like [Sus scrofa]	---	---	---	---
gi|350595116|ref|XP_003360118.2|	PREDICTED: NEDD8 ultimate buster 1 [Sus scrofa]	---	---	---	---
gi|116175275|ref|NP_001070689.1|	transformer-2 protein homolog beta [Sus scrofa] >gi|115371737|gb|ABI96193.1| TRA2B [Sus scrofa]	0.879	1.049	1.069	1.067
gi|6094365|sp|O02799.1|STAT2_PIG	RecName: Full=Signal transducer and activator of transcription 2 >gi|47522708|ref|NP_999054.1| signal transducer and activator of transcription 2 [Sus scrofa] >gi|315273316|gb|ADU03225.1| signal transducer and activator of transcription 2 [Sus scrof	---	---	---	---
gi|346644866|ref|NP_001231105.1|	coiled-coil-helix-coiled-coil-helix domain-containing protein 3, mitochondrial [Sus scrofa]	---	---	---	---
gi|311273314|ref|XP_003133809.1|	PREDICTED: COP9 signalosome complex subunit 8-like isoform 1 [Sus scrofa]	---	---	---	---
gi|4538854|emb|CAB39532.1|	54 kDa vacuolar H(+)-ATPase subunit [Sus scrofa]	---	---	---	---
gi|47523554|ref|NP_999405.1|	V-type proton ATPase subunit H [Sus scrofa] >gi|4538856|emb|CAB39533.1| 54 kDa vacuolar H(+)-ATPase subunit [Sus scrofa]				
gi|18206257|sp|Q9TVC1.1|VATH_PIG	RecName: Full=V-type proton ATPase subunit H; Short=V-ATPase subunit H; AltName: Full=V-ATPase 50/57 kDa subunits; AltName: Full=Vacuolar proton pump subunit H; AltName: Full=Vacuolar proton pump subunit SFD >gi|23956412|emb|CAB39537.1| 54 kDa vacuo				
gi|311263676|ref|XP_003129	PREDICTED: dipeptidyl peptidase	---	---	---	---

789.1|	1-like [Sus scrofa]				
gi|350586576|ref|XP_001929450.4|	PREDICTED: LOW QUALITY PROTEIN: peptidyl-prolyl cis-trans isomerase FKBP5 [Sus scrofa]	0.774	0.52	0.632	0.547
gi|194035744|ref|XP_001928514.1|	PREDICTED: protein Dr1-like [Sus scrofa]	---	---	---	---
gi|346986339|ref|NP_001231321.1|	small glutamine-rich tetratricopeptide repeat-containing protein alpha [Sus scrofa]	1.912	1.27	2.541	2.032
gi|3023862|sp|P81140.1|GCDH_PIG	RecName: Full=Glutaryl-CoA dehydrogenase, mitochondrial; Short=GCD; Flags: Precursor >gi|260168|gb|AAB24225.1| glutaryl-CoA dehydrogenase, GCDH [swine, liver, Peptide Partial, 408 aa]	---	---	---	---
gi|311248913|ref|XP_003123376.1|	PREDICTED: glutaryl-CoA dehydrogenase, mitochondrial-like isoform 1 [Sus scrofa]				
gi|311248915|ref|XP_003123377.1|	PREDICTED: glutaryl-CoA dehydrogenase, mitochondrial-like isoform 2 [Sus scrofa]				
gi|385648282|ref|NP_001245314.1|	glucosidase, beta (bile acid) 2 [Sus scrofa]	0.636	0.76	0.737	0.977
gi|210062464|gb|ABY85807.2|	immunoglobulin gamma chain 6a [Sus scrofa]	---	---	---	---
gi|311275995|ref|XP_003135004.1|	PREDICTED: histone-binding protein RBBP7-like isoform 1 [Sus scrofa]	1.042	0.65	0.931	1.266
gi|350585895|ref|XP_003127841.3|	PREDICTED: MAP7 domain-containing protein 1 [Sus scrofa]	0.378	0.548	0.646	0.417
gi|350592486|ref|XP_001929589.3|	PREDICTED: acyl-CoA dehydrogenase family member 10 [Sus scrofa]	---	---	---	---
gi|350587337|ref|XP_003128893.2|	PREDICTED: peroxisomal acyl-coenzyme A oxidase 3 [Sus scrofa]	---	---	---	---
gi|47116966|sp|P61288.1|TCTP_PIG	RecName: Full=Translationally-controlled tumor protein; Short=TCTP >gi|47523802|ref|NP_99	0.76	0.917	0.893	0.853

	9538.1| translationally-controlled tumor protein [Sus scrofa] >gi|18482460|gb|AAL68965.1| translationally controlled tumor protein [Sus scrofa]				
gi|335281686|ref|XP_003353872.1|	PREDICTED: UBX domain-containing protein 1-like [Sus scrofa]	0.843	1.119	0.973	0.796
gi|75053356|sp|Q764M6.1|IRF3_PIG	RecName: Full=Interferon regulatory factor 3; Short=IRF-3 >gi|47523300|ref|NP_998935.1| interferon regulatory factor 3 [Sus scrofa] >gi|40363627|dbj|BAD06317.1| interferon regulatory factor 3 [Sus scrofa]	---	---	---	---
gi|317016904|gb|ADU85980.1|	interferon regulatory factor 3 [Sus scrofa] >gi|163141076|gb|ABY26589.1| interferon regulatory factor 3 [Sus scrofa]				
gi|261490800|ref|NP_001159781.1|	survival of motor neuron-related-splicing factor 30 [Sus scrofa] >gi|190333253|gb|ACE73645.1| survival motor neuron domain-containing protein 1 [Sus scrofa] >gi|190576653|gb|ACE73646.1| survival motor neuron domain-containing protein 1 [Sus scrofa]	0.834	1.322	1.148	1.092
gi|335282050|ref|XP_003122930.2|	PREDICTED: caprin-1 isoform 1 [Sus scrofa]	---	---	---	---
gi|335282052|ref|XP_003353957.1|	PREDICTED: caprin-1 isoform 2 [Sus scrofa]				
gi|148235477|ref|NP_001090932.1|	protein QIL1 precursor [Sus scrofa] >gi|148839601|sp|A1XQR7.1|QIL1_PIG RecName: Full=Protein QIL1 >gi|117660492|gb|ABK55623.1| QIL1 [Sus scrofa]	---	---	---	---
gi|335301140|ref|XP_003359135.1|	PREDICTED: transmembrane emp24 domain-containing protein 2-like isoform 1 [Sus scrofa]	1.466	1.188	1.206	1.585
gi|335301142|ref|XP_003359136.1|	PREDICTED: transmembrane emp24 domain-containing protein 2-like				

	isoform 2 [Sus scrofa]				
gi|148231223|ref|NP_001090898.1|	mitochondrial antiviral-signaling protein [Sus scrofa] >gi|156144883|gb|ABU53000.1| virus-induced signaling adapter [Sus scrofa] >gi|119507309|dbj|BAF42542.1| mitochondrial antiviral signaling protein [Sus scrofa]	0.771	1.333	1.416	1.219
gi|238176|gb|AAB20199.1|	3,4-dihydroxyphenylalanine (Dopa) decarboxylase [swine, kidney, Peptide, 485 aa]	---	---	---	---
gi|126513274|gb|ABO15741.1|	L-aromatic dopa decarboxylase [Sus scrofa]				
gi|47523148|ref|NP_999019.1|	aromatic-L-amino-acid decarboxylase [Sus scrofa] >gi|2829681|sp|P80041.2|DDC_PIG RecName: Full=Aromatic-L-amino-acid decarboxylase; Short=AADC; AltName: Full=DOPA decarboxylase; Short=DDC >gi|1839555|gb|AAB47157.1| dopa decarboxylase [Sus scrofa] >g				
gi|126513279|gb|ABO15742.1|	L-aromatic dopa decarboxylase splice variant 1 [Sus scrofa]				
gi|350580560|ref|XP_003123249.3|	PREDICTED: transcription activator BRG1 [Sus scrofa]	1.009	0.858	1.256	0.901
gi|311262372|ref|XP_003129135.1|	PREDICTED: N-acylethanolamine-hydrolyzing acid amidase-like [Sus scrofa]	---	---	---	---
gi|335292565|ref|XP_003128593.2|	PREDICTED: charged multivesicular body protein 4a-like [Sus scrofa]	---	---	---	---
gi|343403828|ref|NP_001230291.1|	serine beta-lactamase-like protein LACTB, mitochondrial [Sus scrofa]	0.703			0.932
gi|194036296|ref|XP_001926524.1|	PREDICTED: splicing factor 3B subunit 4 [Sus scrofa]	0.863	1.231	1.161	0.76
gi|297632428|ref|NP_001172097.1|	28S ribosomal protein S28, mitochondrial [Sus scrofa]	0.837	0.477	0.428	0.701
gi|350582501|ref|XP_003481284.1|	PREDICTED: atlastin-2-like isoform 2 [Sus scrofa]	1.596	1.146	0.929	1.273

<table>
<tr><td>gi|350582505|ref|XP_003125271.2|</td><td>PREDICTED: atlastin-2-like isoform 1 [Sus scrofa]</td><td></td><td></td><td></td><td></td></tr>
<tr><td>gi|350582503|ref|XP_003481285.1|</td><td>PREDICTED: atlastin-2-like isoform 3 [Sus scrofa]</td><td></td><td></td><td></td><td></td></tr>
<tr><td>gi|298104120|ref|NP_001177123.1|</td><td>V-type proton ATPase subunit G 1 [Sus scrofa]</td><td>0.329</td><td>0.557</td><td>0.485</td><td>0.429</td></tr>
<tr><td>gi|118403868|ref|NP_001072136.1|</td><td>N-acetylglucosaminyltransferase I [Sus scrofa] >gi|112821182|dbj|BAF03221.1| N-acetylglucosaminyltransferase I [Sus scrofa]</td><td>---</td><td>---</td><td>---</td><td>---</td></tr>
<tr><td>gi|194045136|ref|XP_001927417.1|</td><td>PREDICTED: immunoglobulin-binding protein 1 isoform 2 [Sus scrofa]</td><td>0.871</td><td>0.641</td><td>0.531</td><td>0.896</td></tr>
<tr><td>gi|194045138|ref|XP_001927405.1|</td><td>PREDICTED: immunoglobulin-binding protein 1 isoform 1 [Sus scrofa]</td><td></td><td></td><td></td><td></td></tr>
<tr><td>gi|311264357|ref|XP_003130126.1|</td><td>PREDICTED: suppressor of tumorigenicity 14 protein homolog [Sus scrofa]</td><td>---</td><td>---</td><td>---</td><td>---</td></tr>
<tr><td>gi|147899011|ref|NP_001090950.1|</td><td>40S ribosomal protein S26 [Sus scrofa] >gi|187606917|sp|P49171.4|RS26_PIG RecName: Full=40S ribosomal protein S26 >gi|117661156|gb|ABK55657.1| RPS26 [Sus scrofa]</td><td>---</td><td>---</td><td>---</td><td>---</td></tr>
<tr><td>gi|335307625|ref|XP_003360911.1|</td><td>PREDICTED: 40S ribosomal protein S26-like [Sus scrofa]</td><td></td><td></td><td></td><td></td></tr>
<tr><td>gi|350588024|ref|XP_003482537.1|</td><td>PREDICTED: heterogeneous nuclear ribonucleoprotein D0-like [Sus scrofa]</td><td>---</td><td>---</td><td>---</td><td>---</td></tr>
<tr><td>gi|311254587|ref|XP_003125898.1|</td><td>PREDICTED: CTTNBP2 N-terminal like [Sus scrofa]</td><td>---</td><td>---</td><td>---</td><td>---</td></tr>
<tr><td>gi|147899312|ref|NP_001090923.1|</td><td>F-actin capping protein subunit alpha 1 [Sus scrofa] >gi|148613357|gb|ABQ96222.1| F-actin capping protein alpha 1 subunit [Sus scrofa] >gi|118627572|emb|CAL69435.1| F-actin capping protein subunit alpha 1 [Sus scrofa]</td><td>---</td><td>---</td><td>---</td><td>---</td></tr>
<tr><td>gi|350583525|ref|XP_003481537.1|</td><td>PREDICTED: hypothetical protein LOC100737138 [Sus scrofa]</td><td></td><td></td><td></td><td></td></tr>
</table>

gi|311268984|ref|XP_003132296.1|	PREDICTED: glycerate kinase-like [Sus scrofa]	1.015	0.491	0.604	0.683
gi|350592016|ref|XP_003358886.2|	PREDICTED: LOW QUALITY PROTEIN: protein TFG [Sus scrofa]	---	---	---	---
gi|229626|prf||770504A	hemoglobin alpha	---	---	---	---
gi|402550455|pdb|4F4O|J	Chain J, Structure Of The Haptoglobin-Haemoglobin Complex >gi|402550449|pdb|4F4O|D Chain D, Structure Of The Haptoglobin-Haemoglobin Complex >gi|402550452|pdb|4F4O|G Chain G, Structure Of The Haptoglobin-Haemoglobin Complex >gi|402550446|pdb|4F4O|A				
gi|350581852|ref|XP_003481137.1|	PREDICTED: hemoglobin subunit alpha-like isoform 6 [Sus scrofa] >gi|350581848|ref|XP_003481135.1| PREDICTED: hemoglobin subunit alpha-like isoform 4 [Sus scrofa] >gi|350581844|ref|XP_003481133.1| PREDICTED: hemoglobin subunit alpha-like isoform 2 [S				
gi|178056781|ref|NP_001116594.1|	histone H2A.Z [Sus scrofa] >gi|166407422|gb|ABY87518.1| H2A histone family member Z [Sus scrofa]	---	---	---	---
gi|350595451|ref|XP_003134922.3|	PREDICTED: histone H2A.V-like [Sus scrofa]				
gi|348605274|ref|NP_001231762.1|	alcohol dehydrogenase 5 (class III), chi polypeptide [Sus scrofa]	1.887	0.872	0.792	1.618
gi|335310819|ref|XP_003362206.1|	PREDICTED: ankyrin repeat and LEM domain-containing protein 2, partial [Sus scrofa]	---	---	---	---
gi|335296651|ref|XP_003130877.2|	PREDICTED: tubulin alpha chain-like 3-like [Sus scrofa]	---	---	---	---
gi|350589654|ref|XP_003357831.2|	PREDICTED: tubulin alpha chain-like 3, partial [Sus scrofa]				
gi|311245655|ref|XP_003121912.1|	PREDICTED: nesprin-2-like [Sus scrofa]	---	---	---	---
gi|330340461|ref|NP_001193383.1|	2-hydroxyacyl-CoA lyase 1 [Sus scrofa]	---	---	---	---
gi|335278852|ref|XP_003353	PREDICTED: sorting nexin-9 [Sus	1.186	0.908	0.906	0.666

<table>
<tr><td>219.1|</td><td>scrofa]</td><td></td><td></td><td></td><td></td></tr>
<tr><td>gi|308081427|ref|NP_001183950.1|</td><td>hydroxysteroid dehydrogenase-like protein 2 [Sus scrofa]</td><td>0.789</td><td>0.489</td><td>0.551</td><td>0.593</td></tr>
<tr><td>gi|160333766|ref|NP_001103898.1|</td><td>protein canopy homolog 3 precursor [Sus scrofa] >gi|166216082|sp|A5GFQ5.1|CNPY3_PIG RecName: Full=Protein canopy homolog 3; AltName: Full=Trinucleotide repeat-containing gene 5 protein; Flags: Precursor >gi|147223347|emb|CAN13167.1| trinucleotide re</td><td>0.942</td><td>0.367</td><td>0.762</td><td>0.836</td></tr>
<tr><td>gi|346986322|ref|NP_001231315.1|</td><td>peptidyl-prolyl cis-trans isomerase D [Sus scrofa]</td><td>0.891</td><td>1.065</td><td>0.833</td><td>1.246</td></tr>
<tr><td>gi|350583831|ref|XP_003355400.2|</td><td>PREDICTED: pyridoxal phosphate phosphatase-like, partial [Sus scrofa]</td><td>---</td><td>---</td><td>---</td><td>---</td></tr>
<tr><td>gi|335284421|ref|XP_003354609.1|</td><td>PREDICTED: ataxin 2-like [Sus scrofa]</td><td>---</td><td>---</td><td>---</td><td>---</td></tr>
<tr><td>gi|335284425|ref|XP_003354611.1|</td><td>PREDICTED: ataxin 2-like [Sus scrofa]</td><td></td><td></td><td></td><td></td></tr>
<tr><td>gi|335284427|ref|XP_003354612.1|</td><td>PREDICTED: ataxin 2-like [Sus scrofa]</td><td></td><td></td><td></td><td></td></tr>
<tr><td>gi|335284423|ref|XP_003354610.1|</td><td>PREDICTED: ataxin 2-like [Sus scrofa]</td><td></td><td></td><td></td><td></td></tr>
<tr><td>gi|335284429|ref|XP_003124559.2|</td><td>PREDICTED: ataxin 2-like isoform 1 [Sus scrofa]</td><td></td><td></td><td></td><td></td></tr>
<tr><td>gi|350585179|ref|XP_003481897.1|</td><td>PREDICTED: seryl-tRNA synthetase, mitochondrial-like [Sus scrofa]</td><td>1.123</td><td>0.969</td><td>1.234</td><td>1.477</td></tr>
<tr><td>gi|311266963|ref|XP_003131335.1|</td><td>PREDICTED: putative rRNA methyltransferase 3 [Sus scrofa]</td><td>---</td><td>---</td><td>---</td><td>---</td></tr>
<tr><td>gi|350586678|ref|XP_003482243.1|</td><td>PREDICTED: DNA-directed RNA polymerases I and III subunit RPAC1 [Sus scrofa]</td><td>---</td><td>---</td><td>---</td><td>---</td></tr>
<tr><td>gi|311247599|ref|XP_003122720.1|</td><td>PREDICTED: vacuolar protein sorting-associated protein 37C-like [Sus scrofa]</td><td>---</td><td>---</td><td>---</td><td>---</td></tr>
<tr><td>gi|311262785|ref|XP_003129351.1|</td><td>PREDICTED: ragulator complex protein LAMTOR3-like isoform 3 [Sus scrofa] >gi|311262781|ref|XP_003129349.1| PREDICTED: ragulator</td><td>0.677</td><td>0.447</td><td>0.638</td><td>0.591</td></tr>
</table>

	complex protein LAMTOR3-like isoform 1 [Sus scrofa] >gi|311262783|ref|XP_003129350.1| PREDICTED: ragulator complex protei				
gi|342349340|ref|NP_001230146.1|	carnitine O-octanoyltransferase [Sus scrofa]	---	---	---	---
gi|311253960|ref|XP_001927701.2|	PREDICTED: NADH dehydrogenase [ubiquinone] iron-sulfur protein 2, mitochondrial [Sus scrofa]	---	---	---	---
gi|343790970|ref|NP_001230536.1|	translin-associated protein X [Sus scrofa]	---	---	1.122	---
gi|335303755|ref|XP_003359787.1|	PREDICTED: Golgi phosphoprotein 3-like [Sus scrofa]	---	---	---	---
gi|311272290|ref|XP_003133380.1|	PREDICTED: CDKN2A-interacting protein isoform 1 [Sus scrofa]	---	---	---	---
gi|335302635|ref|XP_003359511.1|	PREDICTED: CDKN2A-interacting protein isoform 2 [Sus scrofa]				
gi|47522786|ref|NP_999145.1|	UTP--glucose-1-phosphate uridylyltransferase [Sus scrofa] >gi|6136110|sp|P79303.3|UGPA_PIG RecName: Full=UTP--glucose-1-phosphate uridylyltransferase; AltName: Full=UDP-glucose pyrophosphorylase; Short=UDPGP; Short=UGPase >gi|1752677|emb|CAA67690.1|	1.821	1.319	1.126	2.247
gi|298104080|ref|NP_001177107.1|	ribosomal protein L30 [Sus scrofa]	---	---	---	---
gi|47522710|ref|NP_999050.1|	mannosyl-oligosaccharide 1,2-alpha-mannosidase IA [Sus scrofa] >gi|41017248|sp|O02773.1|MA1A1_PIG RecName: Full=Mannosyl-oligosaccharide 1,2-alpha-mannosidase IA; AltName: Full=Man(9)-alpha-mannosidase; Short=Man9-mannosidase; AltName: Full=Mannosid	---	---	---	---
gi|156120140|ref|NP_001095287.1|	helicase SKI2W [Sus scrofa] >gi|148724903|emb|CAN87691.1| superkiller viralicidic activity 2-like (S. cerevisiae) [Sus scrofa]	0.943	1.061	1.13	0.702

gi|162138264|gb|ABX82836.1|	putative superkiller viralicidic activity 2 protein [Sus scrofa]				
gi|335284313|ref|XP_003124517.2|	PREDICTED: RNA-binding protein FUS-like isoform 1 [Sus scrofa]	0.713	0.899	0.633	1.032
gi|335284315|ref|XP_003354570.1|	PREDICTED: RNA-binding protein FUS-like isoform 2 [Sus scrofa]				
gi|351738724|gb|AEQ61462.1|	FUS [Sus scrofa]				
gi|311260957|ref|XP_003128592.1|	PREDICTED: NEDD8-like [Sus scrofa]	1.32	2.052	1.478	1.848
gi|335292563|ref|XP_003356757.1|	PREDICTED: NEDD8-like [Sus scrofa]				
gi|194038855|ref|XP_001928049.1|	PREDICTED: hypothetical protein LOC100154068 [Sus scrofa]	---	---	---	---
gi|350591669|ref|XP_003483310.1|	PREDICTED: GMP synthase [glutamine-hydrolyzing]-like [Sus scrofa]	---	---	---	---
gi|335299136|ref|XP_003358504.1|	PREDICTED: poly [ADP-ribose] polymerase 3 [Sus scrofa]	0.853	0.943		0.951
gi|116175255|ref|NP_001070683.1|	regulator of differentiation 1 [Sus scrofa] >gi|115371761|gb|ABI96205.1| ROD1 [Sus scrofa]	---	---	---	---
gi|47523818|ref|NP_999546.1|	calcium/calmodulin-dependent protein kinase type II subunit delta [Sus scrofa] >gi|75073672|sp|Q95266.1|KCC2D_PIG RecName: Full=Calcium/calmodulin-dependent protein kinase type II subunit delta; Short=CaM kinase II subunit delta; Short=CaMK-II subun	1.253	1.013	0.852	1.523
gi|307078155|ref|NP_001182495.1|	splicing factor, arginine/serine-rich 9 [Sus scrofa]	---	---	---	---
gi|349732254|ref|NP_001231868.1|	glutathione S-transferase Mu 3-like [Sus scrofa]	---	---	---	---
gi|335293678|ref|XP_003357027.1|	PREDICTED: ras GTPase-activating protein-binding protein 2 isoform 1 [Sus scrofa]	---	---	---	---
gi|335293682|ref|XP_003357029.1|	PREDICTED: ras GTPase-activating protein-binding protein 2 isoform 3 [Sus scrofa] >gi|335293680|ref|XP_00335				

	7028.1| PREDICTED: ras GTPase-activating protein-binding protein 2 isoform 2 [Sus scrofa]				
gi|335293613|ref|XP_003357005.1|	PREDICTED: UDP-glucuronosyltransferase 2C1-like [Sus scrofa]	---	---	---	---
gi|335308671|ref|XP_003361329.1|	PREDICTED: 60S ribosomal protein L23a-like [Sus scrofa] >gi|311267953|ref|XP_003131820.1| PREDICTED: 60S ribosomal protein L23a-like [Sus scrofa]	0.436	0.214	0.141	0.499
gi|350593534|ref|XP_003483707.1|	PREDICTED: grancalcin-like [Sus scrofa]	1.5	0.974	1.667	1.364
gi|350593538|ref|XP_003483709.1|	PREDICTED: grancalcin-like [Sus scrofa]				
gi|311271760|ref|XP_003133218.1|	PREDICTED: nucleolar and coiled-body phosphoprotein 1 [Sus scrofa]	---	---	---	---
gi|350587063|ref|XP_001924746.3|	PREDICTED: YLP motif-containing protein 1 [Sus scrofa]	0.939	1.091	1.197	0.542
gi|311258899|ref|XP_003127836.1|	PREDICTED: poly(ADP-ribose) glycohydrolase ARH3-like [Sus scrofa]	---	---	---	---
gi|350585235|ref|XP_003481909.1|	PREDICTED: coiled-coil domain-containing protein 97-like [Sus scrofa] >gi|350585231|ref|XP_003127200.3| PREDICTED: coiled-coil domain-containing protein 97-like [Sus scrofa]	0.869	1.668	0.938	0.981
gi|335286349|ref|XP_001927382.3|	PREDICTED: regulator of microtubule dynamics protein 1 [Sus scrofa]	---	---	---	---
gi|350590765|ref|XP_003358260.2|	PREDICTED: zinc finger ZZ-type and EF-hand domain-containing protein 1-like, partial [Sus scrofa]	---	---	---	---
gi|346227226|ref|NP_001230999.1|	40S ribosomal protein S11 [Sus scrofa]	0.559	0.52	0.403	1.019
gi|346644805|ref|NP_001231160.1|	pinin [Sus scrofa]	0.459	0.95	0.861	0.838
gi|350589580|ref|XP_003130823.3|	PREDICTED: dnaJ homolog subfamily C member 1 [Sus scrofa]	---	---	---	---

gi|350580945|ref|XP_003480928.1|	PREDICTED: endoplasmic reticulum aminopeptidase 2-like [Sus scrofa]	0.852	0.642	0.618	0.901
gi|297632397|ref|NP_001172084.1|	high mobility group protein HMG-I/HMG-Y isoform 2 [Sus scrofa] >gi|294494645|gb|ADE93010.1| high mobility group AT-hook protein 1 [Sus scrofa]	---	---	---	---
gi|75050051|sp|Q9GLV6.1|DDX58_PIG	RecName: Full=Probable ATP-dependent RNA helicase DDX58; AltName: Full=DEAD box protein 58; AltName: Full=RHIV-1; AltName: Full=RIG-I-like receptor 1; Short=RLR-1; AltName: Full=RNA helicase induced by virus; AltName: Full=Retinoic acid-inducible ge	---	---	---	---
gi|350596885|ref|XP_003484332.1|	PREDICTED: probable ATP-dependent RNA helicase DDX58-like, partial [Sus scrofa]				
gi|157272171|gb|ABV26717.1|	DEAD/H (Asp-Glu-Ala-Asp/His) box polypeptide [Sus scrofa]				
gi|356460981|ref|NP_998969.2|	probable ATP-dependent RNA helicase DDX58 [Sus scrofa] >gi|224176124|dbj|BAH23574.1| DEAD box polypeptide 58 [Sus scrofa]				
gi|349732258|ref|NP_001231869.1|	uroporphyrinogen decarboxylase [Sus scrofa]	---	---	---	---
gi|350579853|ref|XP_003480698.1|	PREDICTED: liprin-alpha-1-like [Sus scrofa]	1.347	1.462	1.395	0.919
gi|350596914|ref|XP_003361801.2|	PREDICTED: dihydroorotate dehydrogenase (quinone), mitochondrial-like [Sus scrofa]	---	---	---	---
gi|350582270|ref|XP_003125088.3|	PREDICTED: zinc finger protein 638-like [Sus scrofa]	0.733	0.873	1.286	0.906
gi|346716375|ref|NP_001231201.1|	integrin alpha-2 precursor [Sus scrofa]	0.908	0.498	0.769	0.679
gi|335297522|ref|XP_003131393.2|	PREDICTED: LOW QUALITY PROTEIN: glycylpeptide N-tetradecanoyltransferase 1 [Sus scrofa]	---	---	---	---
gi|350590362|ref|XP_003483042.1|	PREDICTED: protein phosphatase 1 regulatory subunit 1B-like [Sus scrofa]	0.988	0.887	0.978	1.135

gi|47522648|ref|NP_999086.1|	beta-hexosaminidase subunit beta precursor [Sus scrofa] >gi|1041667|emb|CAA63123.1| 65 kDa epididymal boar protein [Sus scrofa]	---	---	---	---
gi|262072808|dbj|BAI47712.1|	hexosaminidase B (beta polypeptide) [Sus scrofa]				
gi|78100037|sp|Q29548.2|HEXB_PIG	RecName: Full=Beta-hexosaminidase subunit beta; AltName: Full=65 kDa epididymal boar protein; AltName: Full=Beta-N-acetylhexosaminidase subunit beta; Short=Hexosaminidase subunit B; AltName: Full=N-acetyl-beta-glucosaminidase subunit beta; Flags: Pr				
gi|311248396|ref|XP_003123114.1|	PREDICTED: endophilin-A2-like [Sus scrofa]	---	---	---	---
gi|113205798|ref|NP_001038052.1|	serine/arginine-rich splicing factor 11 [Sus scrofa] >gi|104295153|gb|ABF72046.1| splicing factor, arginine/serine-rich 11 [Sus scrofa]	---	---	---	---
gi|311276941|ref|XP_003135418.1|	PREDICTED: apoptosis-inducing factor 1, mitochondrial isoform 1 [Sus scrofa]	0.848	0.56	0.777	0.636
gi|311276943|ref|XP_003135419.1|	PREDICTED: apoptosis-inducing factor 1, mitochondrial isoform 2 [Sus scrofa]				
gi|335306508|ref|XP_003360488.1|	PREDICTED: apoptosis-inducing factor 1, mitochondrial [Sus scrofa]				
gi|335299816|ref|XP_003358692.1|	PREDICTED: latexin-like [Sus scrofa]	---	---	---	---
gi|335289036|ref|XP_003355772.1|	PREDICTED: adenine phosphoribosyltransferase-like [Sus scrofa] >gi|311256890|ref|XP_003126852.1| PREDICTED: adenine phosphoribosyltransferase-like [Sus scrofa]	1.231	1.138	1.085	0.799
gi|343072|gb|AAA32030.1|	ATPase 8, partial (mitochondrion) [Sus scrofa]	0.683	0.285	0.431	0.745
gi|223976083|ref|YP_002600781.1|	ATP synthase F0 subunit 8 [Sus scrofa domesticus] >gi|5835867|ref|NP_008				

	638.1|ATP8_15069 ATP synthase F0 subunit 8 [Sus scrofa] >gi|347448714|gb|AEO93028.1| ATP synthase F0 subunit 8 (mitochondrion) [Sus scrofa] >gi|347448658|gb|AEO92976.1| ATP synt				
gi|209166273|gb|ACI41640.1|	ATP synthase F0 subunit 8 [Sus scrofa]				
gi|262072813|dbj|BAI47714.1|	hydroxysteroid (17-beta) dehydrogenase 12 [Sus scrofa]	0.728	0.376	0.498	0.648
gi|335281993|ref|XP_003353940.1|	PREDICTED: estradiol 17-beta-dehydrogenase 12 [Sus scrofa]				
gi|8569334|pdb|1EIO|A	Chain A, Ileal Lipid Binding Protein In Complex With Glycocholate	1.497	1.823	0.959	0.734
gi|356460931|ref|NP_999380.2|	gastrotropin [Sus scrofa]				
gi|157830915|pdb|1EAL|A	Chain A, Nmr Study Of Ileal Lipid Binding Protein				
gi|125151|sp|P00571.1|KAD1_PIG	RecName: Full=Adenylate kinase isoenzyme 1; Short=AK 1; AltName: Full=ATP-AMP transphosphorylase 1; AltName: Full=Myokinase >gi|350579686|ref|XP_003122225.3| PREDICTED: adenylate kinase isoenzyme 1 isoform 1 [Sus scrofa] >gi|229498|prf||742496A kina	---	---	---	---
gi|313507265|pdb|3ADK|A	Chain A, Refined Structure Of Porcine Cytosolic Adenylate Kinase At 2.1 Angstroms Resolution				
gi|350579688|ref|XP_003480663.1|	PREDICTED: adenylate kinase isoenzyme 1 [Sus scrofa]				
gi|194035478|ref|XP_001924964.1|	PREDICTED: 60S ribosomal protein L8 [Sus scrofa]	---	---	---	---
gi|87133208|gb|ABD24291.1|	Tax1 binding protein 3 [Sus scrofa]	---	---	---	---
gi|311268143|ref|XP_003131896.1|	PREDICTED: tax1-binding protein 3-like [Sus scrofa]				
gi|350589935|ref|XP_003131063.3|	PREDICTED: tax1-binding protein 3-like, partial [Sus scrofa]				
gi|194036902|ref|XP_001927	PREDICTED: prefoldin subunit	---	---	---	---

358.1|	2-like [Sus scrofa]				
gi|350595023|ref|XP_003484029.1|	PREDICTED: brefeldin A-inhibited guanine nucleotide-exchange protein 2 [Sus scrofa]	---	---	---	---
gi|194044826|ref|XP_001927474.1|	PREDICTED: [Pyruvate dehydrogenase [lipoamide]] kinase isozyme 3, mitochondrial isoform 1 [Sus scrofa]	---	---	---	---
gi|335305794|ref|XP_003360296.1|	PREDICTED: [Pyruvate dehydrogenase [lipoamide]] kinase isozyme 3, mitochondrial isoform 2 [Sus scrofa]				
gi|311263751|ref|XP_003129801.1|	PREDICTED: protein CWC15 homolog isoform 2 [Sus scrofa]	---	---	---	---
gi|335294720|ref|XP_003129800.2|	PREDICTED: protein CWC15 homolog isoform 1 [Sus scrofa]				
gi|90903287|gb|ABE02211.1|	MX2 [Sus scrofa]	---	---	---	---
gi|206557741|sp|A7VK00.1|MX2_PIG	RecName: Full=Interferon-induced GTP-binding protein Mx2; AltName: Full=Myxovirus resistance protein 2 >gi|210050415|dbj|BAG80705.1| Mx2 protein [Sus scrofa] >gi|156720189|dbj|BAF76735.1| Mx2 [Sus scrofa]				
gi|148235632|ref|NP_001090885.1|	interferon-induced GTP-binding protein Mx2 [Sus scrofa] >gi|58701898|gb|AAW81693.1| MX2 [Sus scrofa]				
gi|311253020|ref|XP_003125380.1|	PREDICTED: putative peptidyl-tRNA hydrolase PTRHD1-like [Sus scrofa]	0.406	0.392	0.647	0.521
gi|311258562|ref|XP_003127670.1|	PREDICTED: agmatinase, mitochondrial-like [Sus scrofa]	1.979	0.893	1.312	1.357
gi|1016321|gb|AAA79108.1|	cytochrome P450 2C35, partial [Sus scrofa]	---	---	---	---
gi|47523894|ref|NP_999585.1|	cytochrome P450 2C49 precursor [Sus scrofa] >gi|19071840|dbj|BAB85664.1| cytochrome P450 2C49 [Sus scrofa]				
gi|311248306|ref|XP_003123072.1|	PREDICTED: U6 snRNA-associated Sm-like protein LSm7-like [Sus	0.751	0.974	1.158	1.071

	scrofa]				
gi|335288048|ref|XP_001927642.3|	PREDICTED: partner of Y14 and mago-like [Sus scrofa]	---	---	---	---
gi|37934198|gb|AAP68768.1|	2,4-dienoyl-CoA reductase [Sus scrofa]	1.33	0.905	0.922	0.844
gi|346421316|ref|NP_001177161.2|	2,4-dienoyl-CoA reductase, mitochondrial [Sus scrofa]				
gi|295442674|gb|ADG21261.1|	2,4-dienoyl-CoA reductase 1 [Sus scrofa]				
gi|350582325|ref|XP_003354840.2|	PREDICTED: calcineurin subunit B type 1-like [Sus scrofa] >gi|335306878|ref|XP_003125129.2| PREDICTED: calcineurin subunit B type 1-like [Sus scrofa]	0.823	0.982	0.989	1.117
gi|311259157|ref|XP_003127967.1|	PREDICTED: interferon-induced protein 44-like [Sus scrofa]	0.829	0.924	0.904	1.383
gi|350586528|ref|XP_003482207.1|	PREDICTED: butyrophilin-like protein 2 [Sus scrofa] >gi|350586524|ref|XP_003482205.1| PREDICTED: butyrophilin-like protein 2-like [Sus scrofa]	---	---	---	---
gi|1346657|sp|P48762.1|SL9A1_PIG	RecName: Full=Sodium/hydrogen exchanger 1; AltName: Full=Na(+)/H(+) exchanger 1; Short=NHE-1; AltName: Full=Solute carrier family 9 member 1 >gi|164596|gb|AAA31092.1| Na+-H+ exchanger [Sus scrofa]	---	---	---	---
gi|55741758|ref|NP_001007104.1|	sodium/hydrogen exchanger 1 [Sus scrofa] >gi|240707|gb|AAB20633.1| Na(+)-H+ exchanger [Sus scrofa]				
gi|311264699|ref|XP_003130289.1|	PREDICTED: mitochondrial-processing peptidase subunit beta [Sus scrofa]	---	---	---	---
gi|335308134|ref|XP_003361114.1|	PREDICTED: mitochondrial-processing peptidase subunit beta-like [Sus scrofa]				
gi|350583174|ref|XP_001927278.4|	PREDICTED: acyl-protein thioesterase 1 [Sus scrofa]	2.18	0.982	0.968	1.35
gi|110832818|sp|Q29221.3|CAZA2_PIG	RecName: Full=F-actin-capping protein subunit alpha-2; AltName:	---	---	---	---

	Full=CapZ alpha-2 >gi|148613355|gb|ABQ96221.1| F-actin capping protein alpha 2 subunit [Sus scrofa] >gi|38322751|gb|AAR16302.1| capping protein (actin filament) muscle Z-line, alpha 2 [				
gi|350596747|ref|XP_003484313.1|	PREDICTED: F-actin-capping protein subunit alpha-2-like [Sus scrofa]				
gi|147903595|ref|NP_001090924.1|	F-actin-capping protein subunit alpha-2 [Sus scrofa] >gi|118627574|emb|CAL69436.1| F-actin capping protein subunit alpha 2 [Sus scrofa]				
gi|350595294|ref|XP_003134809.3|	PREDICTED: F-actin-capping protein subunit alpha-2-like [Sus scrofa]				
gi|350591814|ref|XP_003483340.1|	PREDICTED: lipoma-preferred partner-like [Sus scrofa]	0.669	0.732	0.815	0.863
gi|335290435|ref|XP_003356182.1|	PREDICTED: rho guanine nucleotide exchange factor 16 [Sus scrofa]	1.187	0.883	0.642	1.232
gi|311250341|ref|XP_003124106.1|	PREDICTED: transcription initiation factor TFIID subunit 7 [Sus scrofa]	1.001	1.809	1.608	1.345
gi|113205780|ref|NP_001038049.1|	quinone oxidoreductase [Sus scrofa] >gi|104295137|gb|ABF72038.1| zeta-crystallin [Sus scrofa]	---	---	---	---
gi|115502567|sp|Q0MVN8.1|QOR_PIG	RecName: Full=Quinone oxidoreductase; AltName: Full=NADPH:quinone reductase; AltName: Full=Zeta-crystallin >gi|111434264|gb|ABH10011.1| zeta-crystallin [Sus scrofa]				
gi|311249297|ref|XP_003123564.1|	PREDICTED: peptidyl-prolyl cis-trans isomerase FKBP8 [Sus scrofa]	---	---	---	---
gi|339895863|ref|NP_001229960.1|	coiled-coil and C2 domain-containing 1A [Sus scrofa]	---	---	---	---
gi|311247589|ref|XP_003122716.1|	PREDICTED: coiled-coil domain-containing protein 86-like [Sus scrofa]	---	---	---	---
gi|350594682|ref|XP_003134	PREDICTED: D-tyrosyl-tRNA(Tyr)	---	---	---	---

325.3|	deacylase 1-like [Sus scrofa]				
gi|5739517|gb|AAD50507.1|AF176246_1	macrophage migration inhibitory factor, partial [Sus scrofa]	0.505	0.499	0.353	0.26
gi|262204902|dbj|BAI48031.1|	macrophage migration inhibitory factor [Sus scrofa]				
gi|116175251|ref|NP_001070681.1|	macrophage migration inhibitory factor [Sus scrofa] >gi|124075357|sp|P80928.3|MIF_PIG RecName: Full=Macrophage migration inhibitory factor; Short=MIF; AltName: Full=Glycosylation-inhibiting factor; Short=GIF; AltName: Full=L-dopachrome isomerase; Al				
gi|350594292|ref|XP_003134012.3|	PREDICTED: superkiller viralicidic activity 2-like 2 isoform 1 [Sus scrofa]	---	---	---	---
gi|350580668|ref|XP_003123154.3|	PREDICTED: scaffold attachment factor B2 [Sus scrofa]	---	---	---	---
gi|335310888|ref|XP_003362240.1|	PREDICTED: importin subunit alpha-4-like, partial [Sus scrofa]	---	---	---	---
gi|345199331|ref|NP_001230852.1|	CWC27 spliceosome-associated protein homolog [Sus scrofa]	---	---	---	---
gi|350592457|ref|XP_001929016.4|	PREDICTED: CAP-Gly domain-containing linker protein 1 [Sus scrofa]	1.419	1.214	1.321	1.129
gi|350579992|ref|XP_003122608.3|	PREDICTED: EH domain binding protein 1-like 1 [Sus scrofa]	---	---	---	---
gi|356582360|ref|NP_001239162.1|	ADP-ribosylation factor interacting protein 1 [Sus scrofa]	---	---	---	---
gi|194043071|ref|XP_001928539.1|	PREDICTED: gatC-like protein-like [Sus scrofa]	---	---	---	---
gi|227121291|gb|ACP19336.1|	claudin 3 variant 1 [Sus scrofa]	---	---	---	---
gi|229892832|ref|NP_001153547.1|	claudin-3 [Sus scrofa] >gi|227121293|gb|ACP19337.1| claudin 3 variant 2 [Sus scrofa]				
gi|346986400|ref|NP_001231351.1|	kunitz-type protease inhibitor 1 precursor [Sus scrofa]	0.573	0.736	0.762	0.681
gi|350581652|ref|XP_003354625.2|	PREDICTED: cytochrome b-c1 complex subunit 2, mitochondrial-like [Sus scrofa]	---	---	---	---

gi|350593477|ref|XP_003483694.1|	PREDICTED: myosin-VIIb [Sus scrofa]	0.821	0.831	1.173	0.709
gi|335287187|ref|XP_003355292.1|	PREDICTED: ATP synthase subunit b, mitochondrial [Sus scrofa]	---	---	---	---
gi|311275507|ref|XP_003134773.1|	PREDICTED: neural Wiskott-Aldrich syndrome protein-like [Sus scrofa]	0.716	0.861	0.847	0.958
gi|350589312|ref|XP_003130599.3|	PREDICTED: LOW QUALITY PROTEIN: protein ELYS-like [Sus scrofa]	---	---	---	---
gi|344259276|gb|AEN03335.1|	proteasome subunit beta type 6 [Sus scrofa] >gi|344259274|gb|AEN03334.1| proteasome subunit beta type 6 [Sus scrofa]	2.594	0.743	1.107	0.517
gi|346716138|ref|NP_001138398.2|	proteasome subunit beta type-6 [Sus scrofa]				
gi|350592919|ref|XP_001924466.4|	PREDICTED: neutral ceramidase [Sus scrofa]	0.821	1.269	1.808	1.064
gi|311246780|ref|XP_003122339.1|	PREDICTED: haloacid dehalogenase-like hydrolase domain-containing protein 2-like [Sus scrofa]	0.868	1.466	1.718	1.043
gi|350596685|ref|XP_003361498.2|	PREDICTED: haloacid dehalogenase-like hydrolase domain-containing protein 2-like isoform 1 [Sus scrofa]				
gi|311273575|ref|XP_003133933.1|	PREDICTED: NAD kinase domain-containing protein 1 isoform 1 [Sus scrofa]	---	---	---	---
gi|335303800|ref|XP_003359802.1|	PREDICTED: NAD kinase domain-containing protein 1 isoform 2 [Sus scrofa]				
gi|335286747|ref|XP_001924678.3|	PREDICTED: hepatoma-derived growth factor-like [Sus scrofa]	1.35	1.402	1.013	1.412
gi|350594009|ref|XP_003483813.1|	PREDICTED: PERQ amino acid-rich with GYF domain-containing protein 2-like, partial [Sus scrofa]	0.465	0.784	0.856	0.665
gi|311267458|ref|XP_003131579.1|	PREDICTED: 39S ribosomal protein L10, mitochondrial-like [Sus scrofa]	---	---	---	---
gi|335310432|ref|XP_003362030.1|	PREDICTED: splicing factor 3B subunit 3-like [Sus scrofa]	0.927	1.004	0.927	1.065
gi|311269853|ref|XP_003132	PREDICTED: choline-phosphate	0.689	0.563	0.584	0.844

661.1|	cytidylyltransferase A [Sus scrofa]				
gi|311262619|ref|XP_003129271.1|	PREDICTED: mitotic spindle assembly checkpoint protein MAD2A-like [Sus scrofa]	---	---	---	---
gi|335307137|ref|XP_003360720.1|	PREDICTED: BRISC and BRCA1-A complex member 1-like [Sus scrofa]	---	---	---	---
gi|335286799|ref|XP_003355186.1|	PREDICTED: FAD synthase [Sus scrofa] >gi|335286797|ref|XP_001929410.3| PREDICTED: FAD synthase isoform 1 [Sus scrofa]	---	---	---	---
gi|264681454|ref|NP_001161122.1|	S-adenosylmethionine synthase isoform type-2 [Sus scrofa] >gi|262204900|dbj|BAI48030.1| methionine adenosyltransferase II alpha [Sus scrofa]	---	---	---	---
gi|335297289|ref|XP_003357996.1|	PREDICTED: septin-9-like [Sus scrofa]	---	---	---	---
gi|311276184|ref|XP_003135086.1|	PREDICTED: FUN14 domain-containing protein 1-like [Sus scrofa]	0.718	1.337	1.354	1.308
gi|297307135|ref|NP_001171998.1|	protein unc-45 homolog A [Sus scrofa]	---	---	---	---
gi|350582906|ref|XP_001925367.4|	PREDICTED: gasdermin-C-like [Sus scrofa]	---	---	---	---
gi|298160958|ref|NP_001177156.1|	NHP2 non-histone chromosome protein 2-like 1 [Sus scrofa] >gi|298160960|ref|NP_001177155.1| NHP2 non-histone chromosome protein 2-like 1 [Sus scrofa]	---	---	---	---
gi|335306662|ref|XP_003360532.1|	PREDICTED: NHP2-like protein 1-like [Sus scrofa]				
gi|349732194|ref|NP_001231852.1|	armadillo repeat containing 10 [Sus scrofa] >gi|350588904|ref|XP_003482738.1| PREDICTED: armadillo repeat-containing protein 10-like isoform 1 [Sus scrofa]	---	---	---	---
gi|350588908|ref|XP_003482740.1|	PREDICTED: armadillo repeat-containing protein 10-like isoform 3 [Sus scrofa]				
gi|350588906|ref|XP_003482739.1|	PREDICTED: armadillo repeat-containing protein 10-like				

	isoform 2 [Sus scrofa]				
gi|350588910|ref|XP_003482741.1|	PREDICTED: armadillo repeat-containing protein 10-like isoform 4 [Sus scrofa]				
gi|347300383|ref|NP_001231485.1|	aminoacyl tRNA synthase complex-interacting multifunctional protein 2 isoform 1 [Sus scrofa]	---	---	---	---
gi|347300385|ref|NP_001231486.1|	aminoacyl tRNA synthase complex-interacting multifunctional protein 2 isoform 2 [Sus scrofa]				
gi|311272660|ref|XP_003133533.1|	PREDICTED: secernin-3-like [Sus scrofa]	---	---	---	---
gi|343478220|ref|NP_001230376.1|	eukaryotic translation initiation factor 4H 1 [Sus scrofa]	---	---	---	---
gi|343478222|ref|NP_001230377.1|	eukaryotic translation initiation factor 4H 2 [Sus scrofa]				
gi|350597032|ref|XP_001925921.4|	PREDICTED: aldehyde dehydrogenase family 1 member A3, partial [Sus scrofa]	2.31	4.2	3.338	3.713
gi|21703165|gb|AAM76080.1|AF334742_1	immunoglobulin kappa light chain VJ region [Sus scrofa]	---	---	---	---
gi|41323521|gb|AAR99918.1|	immunoglobulin kappa variable region [Sus scrofa]				
gi|41323519|gb|AAR99917.1|	immunoglobulin kappa variable region [Sus scrofa]				
gi|41323567|gb|AAR99941.1|	immunoglobulin kappa variable region [Sus scrofa]				
gi|41323635|gb|AAR99975.1|	immunoglobulin kappa variable region [Sus scrofa]				
gi|41323673|gb|AAR99994.1|	immunoglobulin kappa variable region [Sus scrofa]				
gi|41323593|gb|AAR99954.1|	immunoglobulin kappa variable region [Sus scrofa] >gi|41323525|gb|AAR99920.1| immunoglobulin kappa variable region [Sus scrofa]				
gi|41323677|gb|AAR99996.1|	immunoglobulin kappa variable region [Sus scrofa]				
gi|41323583|gb|AAR99949.1|	immunoglobulin kappa variable region [Sus scrofa]				
gi|41323535|gb|AAR99925.1|	immunoglobulin kappa variable region [Sus scrofa]				

gi|41323653|gb|AAR99984.1|	immunoglobulin kappa variable region [Sus scrofa] >gi|41323645|gb|AAR99980.1| immunoglobulin kappa variable region [Sus scrofa] >gi|41323561|gb|AAR99938.1| immunoglobulin kappa variable region [Sus scrofa] >gi|41323557|gb|AAR99936.1| immunoglobulin				
gi|41323671|gb|AAR99993.1|	immunoglobulin kappa variable region [Sus scrofa]				
gi|41323661|gb|AAR99988.1|	immunoglobulin kappa variable region [Sus scrofa] >gi|41323597|gb|AAR99956.1| immunoglobulin kappa variable region [Sus scrofa] >gi|41323553|gb|AAR99934.1| immunoglobulin kappa variable region [Sus scrofa] >gi|41323541|gb|AAR99928.1| immunoglobulin				
gi|41323619|gb|AAR99967.1|	immunoglobulin kappa variable region [Sus scrofa]				
gi|41323539|gb|AAR99927.1|	immunoglobulin kappa variable region [Sus scrofa]				
gi|41323643|gb|AAR99979.1|	immunoglobulin kappa variable region [Sus scrofa]				
gi|41323633|gb|AAR99974.1|	immunoglobulin kappa variable region [Sus scrofa]				
gi|41323563|gb|AAR99939.1|	immunoglobulin kappa variable region [Sus scrofa]				
gi|41323627|gb|AAR99971.1|	immunoglobulin kappa variable region [Sus scrofa]				
gi|41323509|gb|AAR99912.1|	immunoglobulin kappa variable region [Sus scrofa] >gi|41323551|gb|AAR99933.1| immunoglobulin kappa variable region [Sus scrofa]				
gi|41323665|gb|AAR99990.1|	immunoglobulin kappa variable region [Sus scrofa]				
gi|41323629|gb|AAR99972.1|	immunoglobulin kappa variable region [Sus scrofa]				

gi|41323579|gb|AAR99947.1|	immunoglobulin kappa variable region [Sus scrofa]				
gi|41323569|gb|AAR99942.1|	immunoglobulin kappa variable region [Sus scrofa]				
gi|41323581|gb|AAR99948.1|	immunoglobulin kappa variable region [Sus scrofa]				
gi|41323639|gb|AAR99977.1|	immunoglobulin kappa variable region [Sus scrofa]				
gi|41323681|gb|AAR99998.1|	immunoglobulin kappa variable region [Sus scrofa]				
gi|41323685|gb|AAS00001.1|	immunoglobulin kappa variable region [Sus scrofa]				
gi|41323641|gb|AAR99978.1|	immunoglobulin kappa variable region [Sus scrofa]				
gi|41323683|gb|AAR99999.1|	immunoglobulin kappa variable region [Sus scrofa]				
gi|41323547|gb|AAR99931.1|	immunoglobulin kappa variable region [Sus scrofa]				
gi|41323523|gb|AAR99919.1|	immunoglobulin kappa variable region [Sus scrofa]				
gi|41323623|gb|AAR99969.1|	immunoglobulin kappa variable region [Sus scrofa]				
gi|41323675|gb|AAR99995.1|	immunoglobulin kappa variable region [Sus scrofa]				
gi|41323617|gb|AAR99966.1|	immunoglobulin kappa variable region [Sus scrofa] >gi|41323533|gb|AAR99924.1| immunoglobulin kappa variable region [Sus scrofa]				
gi|41323621|gb|AAR99968.1|	immunoglobulin kappa variable region [Sus scrofa] >gi|41323589|gb|AAR99952.1| immunoglobulin kappa variable region [Sus scrofa] >gi|41323573|gb|AAR99944.1| immunoglobulin kappa variable region [Sus scrofa] >gi|41323611|gb|AAR99963.1| immunoglobulin				
gi|41323655|gb|AAR99985.1|	immunoglobulin kappa variable region [Sus scrofa] >gi|41323651|gb|AAR99983.				

	1| immunoglobulin kappa variable region [Sus scrofa]				
gi|41323565|gb|AAR99940.1|	immunoglobulin kappa variable region [Sus scrofa]				
gi|41323667|gb|AAR99991.1|	immunoglobulin kappa variable region [Sus scrofa]				
gi|350580067|ref|XP_003122733.3|	PREDICTED: protein PAT1 homolog 1 [Sus scrofa]	---	---	---	---
gi|147906270|ref|NP_001090905.1|	E3 ubiquitin-protein ligase XIAP [Sus scrofa] >gi|119567669|gb|ABL84299.1| X-linked inhibitor of apoptosis [Sus scrofa]	---	---	---	---
gi|311249593|ref|XP_003123724.1|	PREDICTED: vesicular integral-membrane protein VIP36 [Sus scrofa]	0.559	0.214	0.214	0.626
gi|270289750|ref|NP_001161887.1|	non-specific lipid-transfer protein [Sus scrofa] >gi|262263195|dbj|BAI48100.1| sterol carrier protein 2 [Sus scrofa]	---	---	---	---
gi|335298382|ref|XP_003358265.1|	PREDICTED: thioredoxin domain-containing protein 17-like isoform 2 [Sus scrofa] >gi|311268187|ref|XP_003131925.1| PREDICTED: thioredoxin domain-containing protein 17-like isoform 1 [Sus scrofa]	---	---	---	---
gi|350584189|ref|XP_003481688.1|	PREDICTED: phosphatidylinositol-5-phosphate 4-kinase type-2 gamma-like [Sus scrofa]	---	---	---	---
gi|194042810|ref|XP_001928060.1|	PREDICTED: serine/threonine-protein phosphatase 2B catalytic subunit beta isoform isoform 2 [Sus scrofa]	---	---	---	---
gi|326368169|emb|CAZ65757.1|	protein phosphatase 3, catalytic subunit, beta isoform [Phacochoerus africanus]				
gi|311271387|ref|XP_001928050.2|	PREDICTED: serine/threonine-protein phosphatase 2B catalytic subunit beta isoform isoform 1 [Sus scrofa]				
gi|326368171|emb|CAZ6575	protein phosphatase 3, catalytic				

8.1|	subunit, beta isoform [Babyrousa babyrussa]				
gi|326368167|emb|CAZ65756.1|	protein phosphatase 3, catalytic subunit, beta isoform [Sus scrofa]				
gi|147225127|emb|CAN13255.1|	novel protein similar to butyrophilin family proteins [Sus scrofa]	0.673	0.418	0.595	0.502
gi|350586516|ref|XP_003128364.3|	PREDICTED: butyrophilin subfamily 1 member A1-like [Sus scrofa]				
gi|147225163|emb|CAN13303.1|	novel protein similar to butyrophilin family proteins [Sus scrofa]				
gi|194035115|ref|XP_001925615.1|	PREDICTED: ubiquitin carboxyl-terminal hydrolase 45 [Sus scrofa]	1.763	---	---	0.168
gi|350591061|ref|XP_003132153.3|	PREDICTED: nuclease EXOG, mitochondrial [Sus scrofa]	---	---	---	---
gi|194035837|ref|XP_001928904.1|	PREDICTED: peroxisomal biogenesis factor 19 isoform 1 [Sus scrofa]	---	---	---	---
gi|311254007|ref|XP_003125714.1|	PREDICTED: peroxisomal biogenesis factor 19 isoform 2 [Sus scrofa]				
gi|148747288|ref|NP_001092072.1|	sideroflexin-1 [Sus scrofa] >gi|166977631|sp|A5A761.1|SFXN1_PIG RecName: Full=Sideroflexin-1 >gi|146741302|dbj|BAF62306.1| sideroflexin 1 [Sus scrofa]	0.951	0.642	0.587	0.663
gi|166796069|ref|NP_001107760.1|	signal peptidase complex subunit 1 [Sus scrofa] >gi|187611464|sp|B0FWK4.1|SPCS1_PIG RecName: Full=Signal peptidase complex subunit 1; AltName: Full=Microsomal signal peptidase 12 kDa subunit; Short=SPase 12 kDa subunit >gi|164598074|gb|ABY61324.1| s	---	---	---	---
gi|311274550|ref|XP_001924843.2|	PREDICTED: 28S ribosomal protein S26, mitochondrial [Sus scrofa]	---	---	---	---
gi|350585199|ref|XP_003355972.2|	PREDICTED: rRNA 2~-O-methyltransferase fibrillarin-like isoform 1 [Sus scrofa]	0.703	0.823	0.753	1.083

gi|350585201|ref|XP_003481901.1|	PREDICTED: rRNA 2~-O-methyltransferase fibrillarin-like isoform 2 [Sus scrofa]				
gi|47522616|ref|NP_999102.1|	ubiquitin-like protein FUBI precursor [Sus scrofa] >gi|1628628|gb|AAB52915.1| ubiquitin-like/S30 ribosomal fusion protein [Sus scrofa]	0.111	0.123	0.101	0.111
gi|51338624|sp|P62863.1|RS30_PIG	RecName: Full=40S ribosomal protein S30				
gi|350589515|ref|XP_003482862.1|	PREDICTED: hypothetical protein LOC100738725 [Sus scrofa]				
gi|75056777|sp|Q8HZV3.1|TFR1_PIG	RecName: Full=Transferrin receptor protein 1; Short=TR; Short=TfR; Short=TfR1; Short=Trfr; AltName: CD_antigen=CD71 >gi|47522828|ref|NP_999166.1| transferrin receptor protein 1 [Sus scrofa] >gi|22854661|gb|AAN09749.1|AF416763_1 transferrin receptor	0.648	0.447	0.379	0.547
gi|298162731|gb|ADI59732.1|	transferrin receptor protein [Sus scrofa]				
gi|311259609|ref|XP_003128180.1|	PREDICTED: alpha-1B-glycoprotein-like [Sus scrofa]	---	---	---	---
gi|350585516|ref|XP_003356167.2|	PREDICTED: cyclin-dependent kinase 11 [Sus scrofa]	---	---	---	---
gi|194042021|ref|XP_001927084.1|	PREDICTED: programmed cell death protein 4 isoform 1 [Sus scrofa]	2.4	0.92	0.975	1.571
gi|335302236|ref|XP_003359415.1|	PREDICTED: programmed cell death protein 4 isoform 2 [Sus scrofa]				
gi|350586098|ref|XP_003482113.1|	PREDICTED: PDZ domain-containing protein GIPC2-like isoform 2 [Sus scrofa]	---	---	---	---
gi|350586100|ref|XP_003356469.2|	PREDICTED: PDZ domain-containing protein GIPC2-like isoform 1 [Sus scrofa]				
gi|335297132|ref|XP_003131142.2|	PREDICTED: cysteinyl-tRNA synthetase, mitochondrial-like [Sus scrofa]	---	---	---	---
gi|148724904|emb|CAN87692.1|	RD RNA binding protein [Sus scrofa]	---	---	---	---

gi|156120154|ref|NP_001095295.1|	negative elongation factor E [Sus scrofa] >gi|148724907|emb|CAN87695.1| RD RNA binding protein [Sus scrofa]				
gi|148724908|emb|CAN87696.1|	RD RNA binding protein [Sus scrofa]				
gi|148724905|emb|CAN87693.1|	RD RNA binding protein [Sus scrofa]				
gi|148724906|emb|CAN87694.1|	RD RNA binding protein [Sus scrofa]				
gi|350579603|ref|XP_003480647.1|	PREDICTED: ribosome-recycling factor, mitochondrial-like [Sus scrofa]	---	---	---	---
gi|350579605|ref|XP_003122165.3|	PREDICTED: ribosome-recycling factor, mitochondrial-like isoform 2 [Sus scrofa]				
gi|345199333|ref|NP_001230853.1|	SEC24 family, member A [Sus scrofa]	---	---	---	---
gi|194038534|ref|XP_001929109.1|	PREDICTED: prostaglandin reductase 2 isoform 1 [Sus scrofa] >gi|311261328|ref|XP_003128705.1| PREDICTED: prostaglandin reductase 2 isoform 2 [Sus scrofa]	---	---	---	---
gi|335288570|ref|XP_003126608.2|	PREDICTED: NADH dehydrogenase [ubiquinone] 1 alpha subcomplex subunit 9, mitochondrial-like [Sus scrofa]	1.076	0.863	0.648	0.825
gi|305855162|ref|NP_001182263.1|	ELAV-like protein 1 [Sus scrofa] >gi|285818484|gb|ADC38915.1| ELAV (embryonic lethal, abnormal vision, Drosophila)-like 1 [Sus scrofa]	0.766	0.611	0.609	0.783
gi|346716187|ref|NP_001231241.1|	WAS/WASL-interacting protein family member 2 [Sus scrofa]	---	---	---	---
gi|47522624|ref|NP_999098.1|	arylsulfatase A precursor [Sus scrofa] >gi|45686371|gb|AAL58668.2|AF316108_1 arylsulfatase A [Sus scrofa]	---	---	---	---
gi|335281873|ref|XP_003353914.1|	PREDICTED: 182 kDa tankyrase-1-binding protein [Sus scrofa]	0.975	0.921	1.108	0.694
gi|350586715|ref|XP_001925	PREDICTED: CD2-associated	---	---	---	---

766.4|	protein-like, partial [Sus scrofa]				
gi|343790936|ref|NP_001230525.1|	KH domain containing, RNA binding, signal transduction associated 1 [Sus scrofa]	---	---	---	---
gi|264681474|ref|NP_001161108.1|	sterol-4-alpha-carboxylate 3-dehydrogenase, decarboxylating [Sus scrofa] >gi|262204910|dbj|BAI48035.1| NAD(P) dependent steroid dehydrogenase-like [Sus scrofa]	---	---	---	---
gi|194042179|ref|XP_001929427.1|	PREDICTED: 40S ribosomal protein S24 isoform 4 [Sus scrofa]	---	---	---	---
gi|335301826|ref|XP_003359293.1|	PREDICTED: 40S ribosomal protein S24 [Sus scrofa] >gi|335301828|ref|XP_003359294.1| PREDICTED: 40S ribosomal protein S24 [Sus scrofa] >gi|335301824|ref|XP_003359292.1| PREDICTED: 40S ribosomal protein S24 [Sus scrofa]				
gi|311271421|ref|XP_001929422.2|	PREDICTED: 40S ribosomal protein S24 isoform 3 [Sus scrofa] >gi|194042181|ref|XP_001929415.1| PREDICTED: 40S ribosomal protein S24 isoform 1 [Sus scrofa]				
gi|335306028|ref|XP_003360371.1|	PREDICTED: 40S ribosomal protein S24-like [Sus scrofa]				
gi|335301836|ref|XP_003359297.1|	PREDICTED: 40S ribosomal protein S24 [Sus scrofa] >gi|311271423|ref|XP_001929420.2| PREDICTED: 40S ribosomal protein S24 isoform 2 [Sus scrofa]				
gi|335301831|ref|XP_003359295.1|	PREDICTED: 40S ribosomal protein S24 [Sus scrofa]				
gi|311273577|ref|XP_003133934.1|	PREDICTED: 40S ribosomal protein S24-like [Sus scrofa]				
gi|227430316|ref|NP_001153064.1|	cytochrome b5 type B [Sus scrofa]	---	---	---	---
gi|311266655|ref|XP_003131200.1|	PREDICTED: charged multivesicular body protein 6-like [Sus scrofa]	---	---	---	---
gi|335299053|ref|XP_003132249.2|	PREDICTED: inosine-5~-monophosphate dehydrogenase 2-like isoform 1 [Sus	2.258	1.474	1.885	1.826

	scrofa]				
gi|335299055|ref|XP_003358476.1|	PREDICTED: inosine-5~-monophosphate dehydrogenase 2-like isoform 2 [Sus scrofa]				
gi|335297344|ref|XP_003131243.2|	PREDICTED: integrin beta-4-like [Sus scrofa]	0.73	1.151	1.519	1.199
gi|335282293|ref|XP_003354026.1|	PREDICTED: UPF0449 protein C19orf25 homolog [Sus scrofa]	---	---	---	---
gi|346421411|ref|NP_001231080.1|	proteasome (prosome, macropain) 26S subunit, ATPase, 1 [Sus scrofa]	---	---	---	---
gi|335290010|ref|XP_003127394.2|	PREDICTED: splicing factor, arginine/serine-rich 19 [Sus scrofa]	0.441	0.39	0.467	0.395
gi|350579829|ref|XP_003480693.1|	PREDICTED: LOW QUALITY PROTEIN: cysteinyl-tRNA synthetase, cytoplasmic-like [Sus scrofa]	---	---	---	---
gi|350592384|ref|XP_001927025.4|	PREDICTED: golgin subfamily A member 3 [Sus scrofa]	---	---	---	---
gi|350597062|ref|XP_003362000.2|	PREDICTED: golgin subfamily A member 3-like, partial [Sus scrofa]				
gi|335300274|ref|XP_003132696.2|	PREDICTED: hematopoietic lineage cell-specific protein [Sus scrofa]	---	---	---	---
gi|311256155|ref|XP_003126523.1|	PREDICTED: DNA-binding protein A-like isoform 1 [Sus scrofa]	---	---	---	---
gi|335288453|ref|XP_003355623.1|	PREDICTED: DNA-binding protein A-like isoform 2 [Sus scrofa]				
gi|350578568|ref|XP_003480395.1|	PREDICTED: beta-1,3-galactosyl-O-glycosyl-glycoprotein beta-1,6-N-acetylglucosaminyltransferase 3-like [Sus scrofa] >gi|350578566|ref|XP_003121521.3| PREDICTED: beta-1,3-galactosyl-O-glycosyl-glycoprotein beta-1,6-N-acetylglucosaminyltransferase 3-l	---	---	---	---
gi|343403767|ref|NP_001230312.1|	solute carrier family 46, member 1 [Sus scrofa]	---	---	---	---
gi|350590667|ref|XP_003483116.1|	PREDICTED: proton-coupled folate transporter-like [Sus scrofa]				

gi|116175263|ref|NP_001070687.1|	ubiquitin-conjugating enzyme E2 J1 [Sus scrofa] >gi|115371729|gb|ABI96189.1| UB2J1 [Sus scrofa]	---	---	---	---
gi|350579496|ref|XP_003353657.2|	PREDICTED: proteasome-associated protein ECM29 homolog [Sus scrofa]	1.214	1.076	1.216	1.332
gi|194474050|ref|NP_001124003.1|	disintegrin and metalloproteinase domain-containing protein 10 precursor [Sus scrofa] >gi|190610714|gb|ACE80208.1| disintegrin and metallopeptidase domain 10 [Sus scrofa]	---	---	---	---
gi|350581743|ref|XP_003481100.1|	PREDICTED: phosphomannomutase 2-like, partial [Sus scrofa]	---	---	---	---
gi|3023900|sp|P79382.3|MGST1_PIG	RecName: Full=Microsomal glutathione S-transferase 1; Short=Microsomal GST-1; AltName: Full=Microsomal GST-I >gi|47523668|ref|NP_999465.1| microsomal glutathione S-transferase 1 [Sus scrofa] >gi|1840393|dbj|BAA19201.1| glutathione S-transferase [Sus	---	---	---	---
gi|350583937|ref|XP_003355432.2|	PREDICTED: LIM domain and actin-binding protein 1-like [Sus scrofa]	---	---	---	---
gi|350584488|ref|XP_003126654.3|	PREDICTED: kinesin family member 21A [Sus scrofa]	---	---	---	---
gi|350595170|ref|XP_003484054.1|	PREDICTED: UPF0562 protein C7orf55 homolog [Sus scrofa]	---	---	---	---
gi|350595172|ref|XP_003484055.1|	PREDICTED: UPF0562 protein C7orf55 homolog [Sus scrofa]				
gi|350595174|ref|XP_003484056.1|	PREDICTED: UPF0562 protein C7orf55 homolog [Sus scrofa]				
gi|335310367|ref|XP_003361999.1|	PREDICTED: heterogeneous nuclear ribonucleoprotein H2-like [Sus scrofa]	0.699	0.928	0.744	0.843
gi|194036098|ref|XP_001929432.1|	PREDICTED: SHC-transforming protein 1 isoform 1 [Sus scrofa]	---	---	---	---
gi|335286795|ref|XP_003355185.1|	PREDICTED: SHC-transforming protein 1 [Sus scrofa]				
gi|3913338|sp|O46658.3|CP2	RecName: Full=Vitamin D(3)	---	---	---	---

DP_PIG	25-hydroxylase; AltName: Full=CYPIID25; AltName: Full=Cytochrome P450 2D25 >gi|47523842|ref|NP_999559.1| vitamin D(3) 25-hydroxylase [Sus scrofa] >gi|2956688|emb|CAA76205.1| vitamin D3 25-Hydroxylase (CYP2D25) [Sus scrofa]				
gi|94490432|gb|ABF29435.1|	cytochrome P450 family 2 subfamily D polypeptide 6 [Sus scrofa]				
gi|10179958|gb|AAG13962.1|	vitamin D-25-hydroxylase [Sus scrofa]				
gi|47523696|ref|NP_999480.1|	hormone-sensitive lipase [Sus scrofa] >gi|2764520|emb|CAA04121.1| Hormone-sensitive lipase [Sus scrofa] >gi|3169109|emb|CAA04122.1| hormone-sensitive lipase [Sus scrofa]	---	---	---	---
gi|302372518|gb|ADL28431.1|	hormone-sensitive lipase [Sus scrofa]				
gi|75071455|sp|Q68J42.1|LIPS_PIG	RecName: Full=Hormone-sensitive lipase; Short=HSL >gi|51101229|gb|AAT95417.1| hormone-sensitive lipase [Sus scrofa]				
gi|51101227|gb|AAT95416.1|	hormone-sensitive lipase [Sus scrofa]				
gi|345110630|ref|NP_001230796.1|	RuvB-like 2 [Sus scrofa]	1.554	0.962	0.722	1.409
gi|335296521|ref|XP_003130802.2|	PREDICTED: acyl-CoA-binding domain-containing protein 5 [Sus scrofa]	---	---	---	---
gi|350586271|ref|XP_003482146.1|	PREDICTED: E3 ubiquitin-protein ligase HECTD3-like, partial [Sus scrofa]	---	---	---	---
gi|343478171|ref|NP_001230355.1|	small nuclear ribonucleoprotein Sm D3 [Sus scrofa]	0.583	0.802	0.423	0.689
gi|16798651|gb|AAL29467.1|AF435591_1	ribosomal protein L19 [Sus scrofa]	---	---	---	---
gi|311267438|ref|XP_003131557.1|	PREDICTED: 60S ribosomal protein L19 [Sus scrofa]				
gi|347300387|ref|NP_001231487.1|	60S ribosomal protein L17 [Sus scrofa]	0.669	0.697	0.429	0.549

gi|1839536|gb|AAB47145.1|	dipeptidyl peptidase II, DPP II, dipeptidyl aminopeptidase II {N-terminal} [swine, seminal plasma, Peptide Partial, 41 aa] >gi|1881845|gb|AAB49532.1| dipeptidyl-peptidase II, DPP II {N-terminal} {EC 3.4.14.2} [swine, seminal plasma, Peptide Partial,	---	---	---	---
gi|157427709|ref|NP_001098761.1|	leucyl-cystinyl aminopeptidase [Sus scrofa] >gi|156763828|emb|CAC84519.1| insulin-regulated aminopeptidase [Sus scrofa] >gi|156778063|emb|CAC88117.1| insulin-regulated aminopeptidase [Sus scrofa]	---	---	---	---
gi|195562223|gb|ACG50182.1|	actin related protein 2/3 complex subunit 2 [Sus scrofa]	1.277	0.509	0.891	1.037
gi|298160991|ref|NP_001177168.1|	actin-related protein 2/3 complex subunit 2 [Sus scrofa]				
gi|350582201|ref|XP_003125018.3|	PREDICTED: hypothetical protein LOC100524080 [Sus scrofa]	---	---	---	---
gi|350579136|ref|XP_003480530.1|	PREDICTED: protein phosphatase 1A-like [Sus scrofa]	0.838	0.943	1.301	0.886
gi|335284548|ref|XP_003354635.1|	PREDICTED: transmembrane channel-like 5 [Sus scrofa]	---	---	---	---
gi|311248055|ref|XP_003122952.1|	PREDICTED: protein lin-7 homolog C-like [Sus scrofa]	0.802	0.753	0.669	0.898
gi|350584725|ref|XP_003126793.3|	PREDICTED: protein lin-7 homolog A-like [Sus scrofa]				
gi|346644810|ref|NP_001231161.1|	protein lin-7 homolog B [Sus scrofa]				
gi|350578494|ref|XP_003480379.1|	PREDICTED: methyl-CpG-binding domain protein 2-like, partial [Sus scrofa]	---	---	---	---
gi|343478269|ref|NP_001230399.1|	RNA 3~-terminal phosphate cyclase [Sus scrofa]	---	---	---	---
gi|223509|prf||0810207A	glucagon 37	---	---	---	---
gi|279613|pir||GCPG	glucagon precursor - pig (fragment)				
gi|46397418|sp|P01274.4|GLUC_PIG	RecName: Full=Glucagon; Contains: RecName: Full=Glicentin; Contains: RecName: Full=Glicentin-related polypeptide; Short=GRPP; Contains:				

	RecName: Full=Oxyntomodulin; Short=OXM; Short=OXY; Contains: RecName: Full=Glucagon; Contains: RecName: Full=Gluc				
gi|509403|emb|CAA84355.1|	BAT1 [Sus scrofa]	---	---	---	---
gi|55742824|ref|NP_001005157.1|	spliceosome RNA helicase DDX39B [Sus scrofa] >gi|47117788|sp|Q29024.2|DX39B_PIG RecName: Full=Spliceosome RNA helicase DDX39B; AltName: Full=56 kDa U2AF65-associated protein; AltName: Full=DEAD box protein UAP56 >gi|162138207|gb|ABX82809.1| HLA-B-as				
gi|350582986|ref|XP_001925424.4|	PREDICTED: eukaryotic translation initiation factor 3 subunit E [Sus scrofa]	---	---	---	---
gi|350529421|ref|NP_001231942.1|	mitochondrial ribosomal protein L49 [Sus scrofa]	---	---	---	---
gi|311250199|ref|XP_003124018.1|	PREDICTED: heterogeneous nuclear ribonucleoprotein A0-like [Sus scrofa]	---	---	---	---
gi|335298275|ref|XP_003131863.2|	PREDICTED: glyoxalase domain-containing protein 4-like [Sus scrofa]	1.799	1.22	1.084	1.553
gi|335310707|ref|XP_003362158.1|	PREDICTED: 39S ribosomal protein L37, mitochondrial-like, partial [Sus scrofa]	---	---	---	---
gi|335286179|ref|XP_003125563.2|	PREDICTED: eukaryotic translation initiation factor 3 subunit H-like isoform 1 [Sus scrofa] >gi|335286181|ref|XP_003355036.1| PREDICTED: eukaryotic translation initiation factor 3 subunit H-like isoform 2 [Sus scrofa]	---	---	---	---
gi|311251198|ref|XP_003124496.1|	PREDICTED: coiled-coil-helix-coiled-coil-helix domain-containing protein 2, mitochondrial-like [Sus scrofa]	---	---	---	---
gi|311273437|ref|XP_003133863.1|	PREDICTED: coiled-coil-helix-coiled-coil-helix domain-containing protein 2,				

	mitochondrial-like [Sus scrofa]				
gi|311262316|ref|XP_003129121.1|	PREDICTED: mps one binder kinase activator-like 1A-like [Sus scrofa]	---	---	---	---
gi|350582248|ref|XP_003481231.1|	PREDICTED: mps one binder kinase activator-like 1B-like [Sus scrofa]				
gi|350581864|ref|XP_003481142.1|	PREDICTED: protein disulfide-isomerase A2-like [Sus scrofa]	0.828	0.529	0.616	0.75
gi|194042794|ref|XP_001925861.1|	PREDICTED: calcium uniporter protein, mitochondrial [Sus scrofa]	---	---	---	---
gi|311245746|ref|XP_001925192.2|	PREDICTED: endophilin-A1-like [Sus scrofa]	---	---	---	---
gi|350583507|ref|XP_003481532.1|	PREDICTED: cold shock domain-containing protein E1-like [Sus scrofa]	---	---	---	---
gi|75065380|sp|Q8MJ49.1|OSTF1_PIG	RecName: Full=Osteoclast-stimulating factor 1 >gi|47522836|ref|NP_999170.1| osteoclast-stimulating factor 1 [Sus scrofa] >gi|21930129|gb|AAM82161.1|AF523268_1 osteoclast stimulating factor [Sus scrofa]	---	---	---	---
gi|350590012|ref|XP_003482975.1|	PREDICTED: FERM, RhoGEF and pleckstrin domain-containing protein 1 [Sus scrofa]	---	---	---	---
gi|350596332|ref|XP_003361056.2|	PREDICTED: FERM, RhoGEF and pleckstrin domain-containing protein 1-like, partial [Sus scrofa]				
gi|350580442|ref|XP_003123420.3|	PREDICTED: trans-2,3-enoyl-CoA reductase-like [Sus scrofa]	---	---	---	---
gi|349732216|ref|NP_001231858.1|	maspardin [Sus scrofa]	---	---	---	---
gi|350578512|ref|XP_003353370.2|	PREDICTED: maspardin-like [Sus scrofa]				
gi|350581893|ref|XP_003481146.1|	PREDICTED: RNA-binding protein with serine-rich domain 1-like isoform 1 [Sus scrofa] >gi|350581895|ref|XP_003481147.1| PREDICTED: RNA-binding protein with serine-rich domain 1-like isoform 2 [Sus scrofa]	---	---	---	---
gi|220900279|gb|ACL82340.	galectin-related protein [Sus scrofa]	---	---	---	---

1|					
gi|350582342|ref|XP_003481250.1|	PREDICTED: galectin-related protein A [Sus scrofa]				
gi|350596839|ref|XP_003484324.1|	PREDICTED: galectin-related protein A-like [Sus scrofa]				
gi|350582694|ref|XP_003481333.1|	PREDICTED: ATP-dependent RNA helicase DDX1-like [Sus scrofa]	---	---	---	---
gi|311248402|ref|XP_003123126.1|	PREDICTED: hepatoma-derived growth factor-related protein 2-like [Sus scrofa]	---	---	---	---
gi|54039371|sp|P62272.3|RS18_PIG	RecName: Full=40S ribosomal protein S18 >gi|47522714|ref|NP_999105.1| 40S ribosomal protein S18 [Sus scrofa] >gi|1841304|dbj|BAA19211.1| ribosomal protein [Sus scrofa]	0.629	0.226	0.178	0.7
gi|194038457|ref|XP_001928802.1|	PREDICTED: retinol dehydrogenase 11 [Sus scrofa]	---	---	---	---
gi|350581931|ref|XP_003354725.2|	PREDICTED: isoleucyl-tRNA synthetase, cytoplasmic-like [Sus scrofa]	---	---	---	---
gi|350581941|ref|XP_003354723.2|	PREDICTED: isoleucyl-tRNA synthetase, cytoplasmic-like [Sus scrofa]				
gi|147832430|emb|CAN59682.1|	HLA-B associated transcript 2 [Sus scrofa]	---	---	---	---
gi|167908789|ref|NP_001108147.1|	HLA-B associated transcript 2 [Sus scrofa] >gi|158537754|gb|ABW73319.1| HLA-B associated transcript 2 [Sus scrofa]				
gi|311250094|ref|XP_003123955.1|	PREDICTED: histidine triad nucleotide-binding protein 1-like [Sus scrofa]	0.766	1.029	0.941	0.7
gi|119416776|emb|CAL92193.1|	elongation initiation factor 4E [Sus scrofa]	---	---	---	---
gi|311262801|ref|XP_003129362.1|	PREDICTED: eukaryotic translation initiation factor 4E [Sus scrofa]				
gi|21703161|gb|AAM76078.1|AF334740_1	immunoglobulin kappa light chain VJ region [Sus scrofa]	---	---	---	---
gi|41323513|gb|AAR99914.1|	immunoglobulin kappa variable region [Sus scrofa]				
gi|41323531|gb|AAR99923.1|	immunoglobulin kappa variable				

	region [Sus scrofa]				
gi|345199326|ref|NP_001230850.1|	sperm associated antigen 7 [Sus scrofa]	---	---	---	---
gi|343432672|ref|NP_001230350.1|	carbohydrate kinase domain-containing protein isoform 1 [Sus scrofa]	1.276	1.35	0.875	0.768
gi|343432674|ref|NP_001230351.1|	carbohydrate kinase domain-containing protein isoform 2 [Sus scrofa]				
gi|350578316|ref|XP_003121377.3|	PREDICTED: absent in melanoma 1 protein [Sus scrofa]	1.747	1.189	1.311	1.295
gi|223950631|ref|NP_001138859.1|	guanine nucleotide-binding protein G(I)/G(S)/G(O) subunit gamma-12 [Sus scrofa] >gi|166915512|gb|ABZ03970.1| guanine nucleotide-binding protein gamma 12 subunit [Sus scrofa]	0.982	0.646	1.014	0.841
gi|194039313|ref|XP_001929257.1|	PREDICTED: kinesin light chain 4 [Sus scrofa]	0.607	0.471	0.544	0.599
gi|311255064|ref|XP_003126064.1|	PREDICTED: mitochondrial import receptor subunit TOM22 homolog [Sus scrofa]	0.746	0.648	0.642	0.877
gi|171948776|gb|ACB59245.1|	cathepsin B [Sus scrofa]	6.941	11.882	9.941	13.412
gi|187470655|sp|A1E295.1|CATB_PIG	RecName: Full=Cathepsin B; Contains: RecName: Full=Cathepsin B light chain; Contains: RecName: Full=Cathepsin B heavy chain; Flags: Precursor >gi|147906534|ref|NP_001090927.1| cathepsin B precursor [Sus scrofa] >gi|118490058|gb|ABK96810.1| cathepsin				
gi|350591026|ref|XP_003132136.3|	PREDICTED: dolichyl-diphosphooligosaccharide--protein glycosyltransferase subunit STT3B [Sus scrofa]	---	---	---	---
gi|335299677|ref|XP_003358644.1|	PREDICTED: U2 snRNP-associated SURP motif-containing protein [Sus scrofa]	---	---	---	---
gi|194018716|ref|NP_001123450.1|	cytochrome b-c1 complex subunit 9 [Sus scrofa] >gi|87047640|gb|ABD18453.1| ubiquinol-cytochrome c reductase	---	---	---	---

	complex [Sus scrofa]				
gi|47575891|ref|NP_001001260.1|	peptidoglycan recognition protein 1 precursor [Sus scrofa] >gi|47028067|gb|AAT09052.1| peptidoglycan recognition protein short isoform 1 [Sus scrofa]	---	---	---	---
gi|350585327|ref|XP_003356027.2|	PREDICTED: peptidoglycan recognition protein 1-like [Sus scrofa]				
gi|343432658|ref|NP_001230343.1|	protein phosphatase 1, regulatory (inhibitor) subunit 8 [Sus scrofa]	---	---	---	---
gi|350591508|ref|XP_003483287.1|	PREDICTED: DNA replication licensing factor MCM2 [Sus scrofa]	---	---	---	---
gi|194035343|ref|XP_001928597.1|	PREDICTED: heme-binding protein 2 [Sus scrofa]	---	---	---	---
gi|350580713|ref|XP_003480884.1|	PREDICTED: hypothetical protein LOC100737370 [Sus scrofa]	---	---	---	---
gi|311275781|ref|XP_003134908.1|	PREDICTED: drebrin-like protein-like [Sus scrofa]	---	---	---	---
gi|264681458|ref|NP_001161123.1|	2-acylglycerol O-acyltransferase 2 [Sus scrofa] >gi|262204904|dbj|BAI48032.1| monoacylglycerol O-acyltransferase 2 [Sus scrofa]	0.608	0.314	0.222	0.311
gi|223950623|ref|NP_001138857.1|	dynein light chain Tctex-type 1 [Sus scrofa] >gi|219563062|gb|ACL27890.1| Tctex-type 1 dynein light chain [Sus scrofa]	---	---	---	---
gi|350591965|ref|XP_003132730.3|	PREDICTED: autophagy-related 3 isoform 2 [Sus scrofa]	---	---	---	---
gi|335283921|ref|XP_003354461.1|	PREDICTED: single Ig IL-1-related receptor [Sus scrofa]	---	---	---	---
gi|335307722|ref|XP_003360950.1|	PREDICTED: single Ig IL-1-related receptor-like [Sus scrofa]				
gi|365796117|dbj|BAL43001.1|	single immunoglobulin IL-1R-related molecule [Sus scrofa]				
gi|350588343|ref|XP_003129759.3|	PREDICTED: disks large homolog 2-like [Sus scrofa]	---	---	---	---
gi|350591860|ref|XP_003358811.2|	PREDICTED: disks large homolog 1, partial [Sus scrofa]				
gi|335289860|ref|XP_003127290.2|	PREDICTED: LOW QUALITY PROTEIN: zinc finger CCCH domain-containing protein 4-like [Sus	---	---	---	---

	scrofa]				
gi|350579966|ref|XP_003122620.3|	PREDICTED: tRNA methyltransferase 112 homolog [Sus scrofa]	---	---	---	---
gi|335282788|ref|XP_003123414.2|	PREDICTED: PDZ domain-containing protein GIPC1-like, partial [Sus scrofa]	---	---	---	---
gi|311271301|ref|XP_001926034.2|	PREDICTED: vacuolar protein sorting-associated protein 26A isoform 1 [Sus scrofa]	1.278	0.537	0.55	1.032
gi|350591121|ref|XP_003358453.2|	PREDICTED: phosphatidylinositide phosphatase SAC1-like [Sus scrofa]	---	---	---	---
gi|350591135|ref|XP_003483215.1|	PREDICTED: phosphatidylinositide phosphatase SAC1-like [Sus scrofa]				
gi|1399282|gb|AAB03248.1|	hypoxanthine phosphoribosyltransferase, partial [Sus scrofa]	1.786	0.932	1.113	1.596
gi|4809241|gb|AAD30159.1|	hypoxanthine phosphoribosyltransferase [Sus scrofa]				
gi|74024903|ref|NP_001027548.1|	hypoxanthine-guanine phosphoribosyltransferase [Sus scrofa] >gi|85542050|sp|Q45FY6.3|HPRT_PIG RecName: Full=Hypoxanthine-guanine phosphoribosyltransferase; Short=HGPRT; Short=HGPRTase >gi|71842219|gb|AAZ43258.1| hypoxanthine phosphoribosyltransferas				
gi|1661110|gb|AAB18389.1|	hypoxanthine phosphoribosyltransferase [Sus scrofa]				
gi|264681434|ref|NP_001161112.1|	asparagine synthetase [Sus scrofa] >gi|262036930|dbj|BAI47600.1| asparagine synthetase [Sus scrofa]	---	---	---	---
gi|350579892|ref|XP_003122508.3|	PREDICTED: coronin-1B-like [Sus scrofa]	1.087	1.264	1.046	1.222
gi|147225114|emb|CAN13231.1|	dolichyl-phosphate mannosyltransferase polypeptide 1, catalytic subunit [Sus scrofa] >gi|147223388|emb|CAN13123.1| dolichyl-phosphate	---	---	---	---

	mannosyltransferase polypeptide 1, catalytic subunit [Sus scrofa]				
gi|167001855|ref|NP_001095290.1|	dolichol-phosphate mannosyltransferase [Sus scrofa] >gi|158513309|sp|A5GFZ5.1|DPM1_PIG RecName: Full=Dolichol-phosphate mannosyltransferase; AltName: Full=Dolichol-phosphate mannose synthase; Short=DPM synthase; AltName: Full=Dolichyl-phosphate beta				
gi|262072949|dbj|BAI47782.1|	dolichyl-phosphate mannosyltransferase polypeptide 1, catalytic subunit [Sus scrofa]				
gi|350593701|ref|XP_003483747.1|	PREDICTED: hypothetical protein LOC100737189 [Sus scrofa]	---	---	---	---
gi|350580832|ref|XP_003123718.3|	PREDICTED: clathrin light chain B-like isoform 1 [Sus scrofa]	0.561	1.155	0.995	0.586
gi|311275636|ref|XP_003134838.1|	PREDICTED: septin-7-like isoform 1 [Sus scrofa]	1.268	0.852	0.765	1.169
gi|350595354|ref|XP_003134840.2|	PREDICTED: septin-7-like isoform 3 [Sus scrofa]				
gi|335305478|ref|XP_003360219.1|	PREDICTED: septin-7-like [Sus scrofa]				
gi|311251345|ref|XP_003124567.1|	PREDICTED: rab GTPase-binding effector protein 2-like [Sus scrofa]	---	---	---	---
gi|350583690|ref|XP_003125999.3|	PREDICTED: tetratricopeptide repeat protein 38-like [Sus scrofa]	2.899	1.326	1.043	1.622
gi|350593076|ref|XP_001925759.4|	PREDICTED: vesicle transport through interaction with t-SNAREs homolog 1A-like, partial [Sus scrofa]	---	---	---	---
gi|350593078|ref|XP_001926059.4|	PREDICTED: vesicle transport through interaction with t-SNAREs homolog 1A-like, partial [Sus scrofa]				
gi|311253520|ref|XP_003125581.1|	PREDICTED: 60S ribosomal protein L35a-like [Sus scrofa]	---	---	---	---
gi|335300197|ref|XP_003358821.1|	PREDICTED: 60S ribosomal protein L35a-like isoform 1 [Sus scrofa] >gi|335294628|ref|XP_003357274.1| PREDICTED: 60S ribosomal protein L35a-like [Sus				

	scrofa] >gi|335300199|ref|XP_003358822.1| PREDICTED: 60S ribosomal protein L35a-like isoform 2 [Sus s				
gi|350580587|ref|XP_003123193.3|	PREDICTED: neuropathy target esterase isoform 1 [Sus scrofa]	---	---	---	---
gi|350580589|ref|XP_003480855.1|	PREDICTED: neuropathy target esterase [Sus scrofa]				
gi|311269349|ref|XP_003132451.1|	PREDICTED: histone H1x-like [Sus scrofa]	---	---	---	---
gi|350584300|ref|XP_003355577.2|	PREDICTED: FYVE, RhoGEF and PH domain-containing protein 4-like [Sus scrofa]	---	---	---	---
gi|178056175|ref|NP_001116558.1|	basigin precursor [Sus scrofa] >gi|166244451|gb|ABY86570.1| CD147 [Sus scrofa]	0.412	0.438	0.408	0.371
gi|194033639|ref|XP_001927971.1|	PREDICTED: prostaglandin E synthase 2 [Sus scrofa]	---	---	---	---
gi|246580|gb|AAB21636.1|	zona pellucida-binding protein, AWN-1=C12 fragment [swine, sperm, Peptide Partial, 22 aa]	0.19		0.186	0.22
gi|335309560|ref|XP_003361682.1|	PREDICTED: DNA-directed RNA polymerase I subunit RPA34-like [Sus scrofa]	---	---	---	---
gi|311275477|ref|XP_003134759.1|	PREDICTED: staphylococcal nuclease domain-containing protein 1-like [Sus scrofa]	---	---	---	---
gi|61214649|sp|Q767K9.1|PP1RA_PIG	RecName: Full=Serine/threonine-protein phosphatase 1 regulatory subunit 10; AltName: Full=MHC class I region proline-rich protein CAT53; AltName: Full=Protein FB19 >gi|178056844|ref|NP_001116637.1| serine/threonine-protein phosphatase 1 regulatory s	0.821	0.933	1.391	0.92
gi|311271051|ref|XP_003133041.1|	PREDICTED: synaptosomal-associated protein 29-like [Sus scrofa]	---	---	---	---
gi|350594395|ref|XP_003359859.2|	PREDICTED: transportin-1 [Sus scrofa]	0.539	0.288	1.011	0.628
gi|195539480|ref|NP_001124203.1|	bystin [Sus scrofa] >gi|155964243|gb|ABU40181	---	---	---	---

	.1| bystin-like [Sus scrofa]				
gi|350595768|ref|XP_003135230.3|	PREDICTED: phosphorylase b kinase regulatory subunit alpha, skeletal muscle isoform-like, partial [Sus scrofa]	---	---	---	---
gi|296531513|ref|NP_001171875.1|	lymphocyte antigen 75 precursor [Sus scrofa] >gi|283854885|gb|ACV89989.1| CD205 [Sus scrofa]	---	---	---	---
gi|50657386|ref|NP_001002801.1|	apolipoprotein C-III precursor [Sus scrofa] >gi|416627|sp|P27917.2|APOC3_PIG RecName: Full=Apolipoprotein C-III; Short=Apo-CIII; Short=ApoC-III; AltName: Full=Apolipoprotein C3; Flags: Precursor >gi|164361|gb|AAA30993.1| apolipoprotein C-III [Sus sc	0.204	0.24	0.217	0.237
gi|311249451|ref|XP_003123641.1|	PREDICTED: uncharacterized protein UNQ511/PRO1026 homolog isoform 1 [Sus scrofa]	---	---	---	---
gi|335282983|ref|XP_003354207.1|	PREDICTED: uncharacterized protein UNQ511/PRO1026 homolog isoform 2 [Sus scrofa]				
gi|350582720|ref|XP_003481337.1|	PREDICTED: isoamyl acetate-hydrolyzing esterase 1 homolog [Sus scrofa]	---	---	---	---
gi|951375|gb|AAA74655.1|	apolipoprotein B [Sus scrofa]	---	---	---	---
gi|350597081|ref|XP_003484354.1|	PREDICTED: LOW QUALITY PROTEIN: apolipoprotein B-100 [Sus scrofa]				
gi|6013139|gb|AAF01257.1|AF109646_1	coxsackie-adenovirus-receptor homolog [Sus scrofa]	---	---	---	---
gi|350592092|ref|XP_001924589.4|	PREDICTED: coxsackievirus and adenovirus receptor homolog isoform 1 [Sus scrofa]				
gi|350586377|ref|XP_003128223.3|	PREDICTED: thioredoxin domain-containing protein 5-like, partial [Sus scrofa]	---	---	---	---
gi|190360629|ref|NP_001121936.1|	stromal membrane-associated protein 2 [Sus scrofa] >gi|183223973|dbj|BAG24503.1| stromal membrane-associated protein 1-like [Sus scrofa]	---	---	---	---

gi|335300981|ref|XP_001926354.2|	PREDICTED: 60S acidic ribosomal protein P1-like [Sus scrofa]	---	---	---	---
gi|311248936|ref|XP_003123385.1|	PREDICTED: phenylalanyl-tRNA synthetase alpha chain-like [Sus scrofa]	---	---	---	---
gi|335308639|ref|XP_003361313.1|	PREDICTED: probable hydrolase PNKD-like, partial [Sus scrofa]	0.744	0.459	0.519	0.559
gi|350587090|ref|XP_001926625.4|	PREDICTED: serine palmitoyltransferase 2 [Sus scrofa]	---	---	---	---
gi|350589269|ref|XP_003357681.2|	PREDICTED: LOW QUALITY PROTEIN: MOSC domain-containing protein 1, mitochondrial-like [Sus scrofa]	---	---	---	---
gi|350589272|ref|XP_003482825.1|	PREDICTED: MOSC domain-containing protein 1, mitochondrial-like [Sus scrofa]				
gi|335296836|ref|XP_003357876.1|	PREDICTED: hypothetical protein LOC100624456 [Sus scrofa]	---	---	---	---
gi|335301996|ref|XP_001925416.3|	PREDICTED: insulin-degrading enzyme isoform 1 [Sus scrofa]	---	---	---	---
gi|335301998|ref|XP_003359343.1|	PREDICTED: insulin-degrading enzyme isoform 2 [Sus scrofa]				
gi|350581169|ref|XP_003124100.2|	PREDICTED: UPF0467 protein C5orf32 homolog [Sus scrofa]	---	---	---	---
gi|227336237|gb|ACP20834.1|	selenocysteine lyase [Sus scrofa]	---	---	---	---
gi|311273333|ref|XP_003133817.1|	PREDICTED: selenocysteine lyase [Sus scrofa]				
gi|311267394|ref|XP_003131544.1|	PREDICTED: 26S proteasome non-ATPase regulatory subunit 3-like [Sus scrofa]	---	---	---	---
gi|335307890|ref|XP_003131560.2|	PREDICTED: 26S proteasome non-ATPase regulatory subunit 3-like, partial [Sus scrofa]				
gi|50401716|sp|Q6J1I8.1|RN114_PIG	RecName: Full=RING finger protein 114; AltName: Full=Zinc finger protein 313 >gi|49274651|ref|NP_001001869.1| RING finger protein 114 [Sus scrofa] >gi|47607447|gb|AAT36620.1| zinc finger protein 313 [Sus scrofa]	---	---	---	---
gi|335298143|ref|XP_003131	PREDICTED: zinc finger protein 207	---	---	---	---

778.2|	isoform 1 [Sus scrofa]				
gi|335298147|ref|XP_003131780.2|	PREDICTED: zinc finger protein 207 isoform 3 [Sus scrofa]				
gi|335298145|ref|XP_003131779.2|	PREDICTED: zinc finger protein 207 isoform 2 [Sus scrofa]				
gi|335307509|ref|XP_003360867.1|	PREDICTED: lipoma-preferred partner-like, partial [Sus scrofa]	0.795	1.317	1.276	1.036
gi|350591812|ref|XP_003483339.1|	PREDICTED: lipoma-preferred partner-like [Sus scrofa]				
gi|335294600|ref|XP_003129756.2|	PREDICTED: pre-mRNA cleavage complex 2 protein Pcf11 [Sus scrofa]	0.728	1.004	1.125	0.759
gi|28373591|pdb|1M3E|C	Chain C, Succinyl-Coa:3-Ketoacid Coa Transferase From Pig Heart (Selenomethionine) >gi|28373589|pdb|1M3E|A Chain A, Succinyl-Coa:3-Ketoacid Coa Transferase From Pig Heart (Selenomethionine) >gi|28373592|pdb|1M3E|D Chain D, Succinyl-Coa:3-Ketoacid Co	---	---	---	---
gi|300193176|pdb|3K6M|B	Chain B, Dynamic Domains Of Succinyl-Coa:3-Ketoacid-Coenzyme A Transferase From Pig Heart. >gi|300193174|pdb|3K6M|D Chain D, Dynamic Domains Of Succinyl-Coa:3-Ketoacid-Coenzyme A Transferase From Pig Heart. >gi|300193175|pdb|3K6M|C Chain C, Dynamic				
gi|395398464|sp|Q29551.2|SCOT1_PIG	RecName: Full=Succinyl-CoA:3-ketoacid coenzyme A transferase 1, mitochondrial; AltName: Full=3-oxoacid CoA-transferase 1; AltName: Full=Somatic-type succinyl-CoA:3-oxoacid CoA-transferase; Short=SCOT-s; Flags: Precursor				
gi|47522610|ref|NP_999103.1|	succinyl-CoA:3-ketoacid coenzyme A transferase 1, mitochondrial precursor [Sus scrofa] >gi|164423|gb|AAA31019.1| succinyl-CoA:alpha-ketoacid				

	coenzyme A transferase [Sus scrofa]				
gi|158429140|pdb|2NRC|D	Chain D, C28a Mutant Of Succinyl-Coa:3-Ketoacid Coa Transferase From Pig Heart >gi|158429138|pdb|2NRC|B Chain B, C28a Mutant Of Succinyl-Coa:3-Ketoacid Coa Transferase From Pig Heart >gi|158429139|pdb|2NRC|C Chain C, C28a Mutant Of Succinyl-Coa:3-Ke				
gi|48425138|pdb|1OPE|A	Chain A, Deletion Mutant Of Succinyl-Coa:3-Ketoacid Coa Transferase From Pig Heart >gi|48425136|pdb|1OOZ|A Chain A, Deletion Mutant Of Succinyl-Coa:3-Ketoacid Coa Transferase From Pig Heart >gi|48425139|pdb|1OPE|B Chain B, Deletion Mutant Of Succiny				
gi|158429135|pdb|2NRB|C	Chain C, C28s Mutant Of Succinyl-Coa:3-Ketoacid Coa Transferase From Pig Heart >gi|158429133|pdb|2NRB|A Chain A, C28s Mutant Of Succinyl-Coa:3-Ketoacid Coa Transferase From Pig Heart >gi|158429136|pdb|2NRB|D Chain D, C28s Mutant Of Succinyl-Coa:3-Ke				
gi|350591891|ref|XP_003132672.3|	PREDICTED: uridine 5~-monophosphate synthase-like [Sus scrofa]	1.043	0.583	0.734	0.766
gi|311243865|ref|XP_003121220.1|	PREDICTED: costars family protein C6orf115-like isoform 2 [Sus scrofa]	---	---	---	---
gi|335278956|ref|XP_003353243.1|	PREDICTED: costars family protein C6orf115-like [Sus scrofa] >gi|335278958|ref|XP_003353244.1| PREDICTED: costars family protein C6orf115-like [Sus scrofa]				
gi|350596336|ref|XP_003361058.2|	PREDICTED: desmocollin-2 [Sus scrofa]	1.338	1.363	1.878	1.045
gi|350579705|ref|XP_003122	PREDICTED: TBC1 domain family	---	---	---	---

274.3|	member 13 [Sus scrofa]				
gi|311254945|ref|XP_003126007.1|	PREDICTED: nuclear pore complex protein Nup50 [Sus scrofa]	---	---	---	---
gi|350596555|ref|XP_003484289.1|	PREDICTED: nuclear pore complex protein Nup50-like [Sus scrofa]				
gi|350591479|ref|XP_003483280.1|	PREDICTED: acyl-CoA dehydrogenase family member 9, mitochondrial-like, partial [Sus scrofa]	---	---	---	---
gi|335289868|ref|XP_003127303.2|	PREDICTED: coiled-coil domain-containing protein 9 [Sus scrofa]	---	---	---	---
gi|350593239|ref|XP_003483642.1|	PREDICTED: UBX domain-containing protein 4-like [Sus scrofa]	---	---	---	---
gi|350587143|ref|XP_001927117.4|	PREDICTED: legumain [Sus scrofa]	---	---	---	---
gi|350589300|ref|XP_003130593.3|	PREDICTED: hypothetical protein LOC100525245 [Sus scrofa]	0.586	0.804	0.667	0.852
gi|335300577|ref|XP_003358945.1|	PREDICTED: ubiquitin carboxyl-terminal hydrolase 16-like isoform 1 [Sus scrofa]	---	---	---	---
gi|311270664|ref|XP_001924462.2|	PREDICTED: V-set and immunoglobulin domain-containing protein 10 [Sus scrofa]	---	---	---	---
gi|335307295|ref|XP_003360785.1|	PREDICTED: tetraspanin-31-like [Sus scrofa]	0.264	0.17		0.255
gi|350588836|ref|XP_003357506.2|	PREDICTED: tetraspanin-13-like [Sus scrofa]				
gi|194044687|ref|XP_001928924.1|	PREDICTED: acyl-coenzyme A thioesterase 8 [Sus scrofa]	---	---	---	---
gi|335284373|ref|XP_003354586.1|	PREDICTED: LOW QUALITY PROTEIN: E3 ubiquitin-protein ligase BRE1B-like [Sus scrofa]	---	---	---	---
gi|126302599|sp|P04163.2|S10AA_PIG	RecName: Full=Protein S100-A10; AltName: Full=Calpactin I light chain; AltName: Full=Calpactin-1 light chain; AltName: Full=Cellular ligand of annexin II; AltName: Full=S100 calcium-binding protein A10; AltName: Full=p10 protein; AltName: Full=p11	0.619	1.123	1.869	1.304

gi|335286919|ref|XP_003355216.1|	PREDICTED: protein S100-A10-like isoform 4 [Sus scrofa] >gi|335286915|ref|XP_003355214.1| PREDICTED: protein S100-A10-like isoform 2 [Sus scrofa] >gi|335286917|ref|XP_003355215.1| PREDICTED: protein S100-A10-like isoform 3 [Sus scrofa] >gi|311254303				
gi|335288415|ref|XP_003355612.1|	PREDICTED: serine-threonine kinase receptor-associated protein-like [Sus scrofa]	---	---	---	---
gi|350591920|ref|XP_003483358.1|	PREDICTED: poly [ADP-ribose] polymerase 14 [Sus scrofa]	---	---	---	---
gi|158514031|sp|A1XQU5.1|RL27_PIG	RecName: Full=60S ribosomal protein L27 >gi|148226949|ref|NP_001090948.1| 60S ribosomal protein L27 [Sus scrofa] >gi|117661046|gb|ABK55651.1| RPL27 [Sus scrofa]	---	---	---	---
gi|47523714|ref|NP_999490.1|	mitochondrial Rho GTPase 2 [Sus scrofa] >gi|75072868|sp|Q864R5.1|MIRO2_PIG RecName: Full=Mitochondrial Rho GTPase 2; Short=MIRO-2; AltName: Full=Ras homolog gene family member T2 >gi|30025664|gb|AAP04408.1| rho GTPase [Sus scrofa]	---	---	---	---
gi|342187294|ref|NP_001230119.1|	syndecan-1 precursor [Sus scrofa]	---	---	---	---
gi|194042152|ref|XP_001924391.1|	PREDICTED: WD repeat-containing protein 11 [Sus scrofa]	---	---	---	---
gi|5915886|sp|O46427.1|CATH_PIG	RecName: Full=Pro-cathepsin H; Contains: RecName: Full=Cathepsin H mini chain; Contains: RecName: Full=Cathepsin H; Contains: RecName: Full=Cathepsin H heavy chain; Contains: RecName: Full=Cathepsin H light chain; Flags: Precursor >gi|47522632|ref|N	---	---	---	---
gi|156046107|gb|ABU42573.1|	cathepsin H variant 2 [Sus scrofa]				
gi|172050735|gb|ACB70169.	cathepsin H transcript variant 3 [Sus				

1|	scrofa]				
gi|28948787|pdb|1NB3|C	Chain C, Crystal Structure Of Stefin A In Complex With Cathepsin H: N-Terminal Residues Of Inhibitors Can Adapt To The Active Sites Of Endo-And Exopeptidases >gi|28948781|pdb|1NB3|A Chain A, Crystal Structure Of Stefin A In Complex With Cathepsin H:				
gi|171948778|gb|ACB59246.1|	cathepsin H [Sus scrofa]				
gi|350588133|ref|XP_003482569.1|	PREDICTED: LOW QUALITY PROTEIN: interferon-induced very large GTPase 1, partial [Sus scrofa]	---	---	---	---
gi|194041277|ref|XP_001924306.1|	PREDICTED: TATA element modulatory factor [Sus scrofa]	---	---	---	---
gi|350590398|ref|XP_003131591.3|	PREDICTED: CDK5 regulatory subunit-associated protein 3-like [Sus scrofa]	---	---	---	---
gi|350582111|ref|XP_003124939.2|	PREDICTED: LOW QUALITY PROTEIN: eukaryotic translation initiation factor 5B [Sus scrofa]	---	---	---	---
gi|335309033|ref|XP_003121764.2|	PREDICTED: organic solute transporter subunit beta-like [Sus scrofa]	---	---	---	---
gi|335310602|ref|XP_003362108.1|	PREDICTED: echinoderm microtubule-associated protein-like 2-like, partial [Sus scrofa]	---	---	---	---
gi|350594364|ref|XP_003134050.3|	PREDICTED: protein LAP2 [Sus scrofa]	---	---	---	---
gi|343488514|ref|NP_001230416.1|	ubiquitin-related modifier 1 homolog [Sus scrofa]	---	---	---	---
gi|350582717|ref|XP_003125434.3|	PREDICTED: rho-associated protein kinase 2, partial [Sus scrofa]	---	---	---	---
gi|350580947|ref|XP_003123849.3|	PREDICTED: endoplasmic reticulum aminopeptidase 2-like, partial [Sus scrofa]	0.801	0.635	0.615	0.908
gi|212549621|ref|NP_001131100.1|	growth factor receptor-bound protein 2 [Sus scrofa] >gi|208612619|gb|ACI29749.1| growth factor receptor bound	---	---	---	---

	protein 2 [Sus scrofa]				
gi|311248892|ref|XP_003123362.1|	PREDICTED: lysosomal alpha-mannosidase-like isoform 1 [Sus scrofa]	---	---	---	---
gi|311275439|ref|XP_003134730.1|	PREDICTED: transportin-3 isoform 1 [Sus scrofa]	---	---	---	---
gi|335305352|ref|XP_003360189.1|	PREDICTED: transportin-3 isoform 2 [Sus scrofa]				
gi|335305354|ref|XP_003360190.1|	PREDICTED: transportin-3 isoform 3 [Sus scrofa]				
gi|116175267|ref|NP_001070690.1|	U1 small nuclear ribonucleoprotein A [Sus scrofa] >gi|122131847|sp|Q06AA4.1|SNRPA_PIG RecName: Full=U1 small nuclear ribonucleoprotein A; Short=U1 snRNP A; Short=U1-A; Short=U1A >gi|115371743|gb|ABI96196.1| SNRPA [Sus scrofa]	---	---	---	---
gi|116175273|ref|NP_001070696.1|	uncharacterized protein LOC768116 [Sus scrofa] >gi|115371759|gb|ABI96204.1| RU2B [Sus scrofa]				
gi|311248398|ref|XP_003123116.1|	PREDICTED: chromatin assembly factor 1 subunit A-like [Sus scrofa]	---	---	---	---
gi|350578878|ref|XP_003353487.2|	PREDICTED: tight junction protein ZO-1 [Sus scrofa]	---	---	---	---
gi|194042423|ref|XP_001926989.1|	PREDICTED: cleavage stimulation factor subunit 2 tau variant isoform 1 [Sus scrofa]	---	---	---	---
gi|311276614|ref|XP_003135279.1|	PREDICTED: cleavage stimulation factor subunit 2-like isoform 1 [Sus scrofa]				
gi|335306285|ref|XP_003360436.1|	PREDICTED: cleavage stimulation factor subunit 2-like isoform 2 [Sus scrofa]				
gi|350593080|ref|XP_001927062.4|	PREDICTED: LOW QUALITY PROTEIN: uncharacterized protein C10orf118 [Sus scrofa]	---	---	---	---
gi|311249343|ref|XP_003123584.1|	PREDICTED: coatomer subunit epsilon-like isoform 1 [Sus scrofa]	---	---	---	---
gi|311249345|ref|XP_003123586.1|	PREDICTED: coatomer subunit epsilon-like isoform 3 [Sus scrofa]				

gi|350595741|ref|XP_003484170.1|	PREDICTED: disks large homolog 3 isoform 2 [Sus scrofa]	---	---	---	---
gi|350595743|ref|XP_003135199.3|	PREDICTED: disks large homolog 3 isoform 1 [Sus scrofa]				
gi|350595745|ref|XP_003484171.1|	PREDICTED: disks large homolog 3 isoform 3 [Sus scrofa]				
gi|311276931|ref|XP_003135414.1|	PREDICTED: U3 small nucleolar RNA-associated protein 14 homolog A-like isoform 1 [Sus scrofa]	0.44	0.533	0.443	0.578
gi|311276933|ref|XP_003135415.1|	PREDICTED: U3 small nucleolar RNA-associated protein 14 homolog A-like isoform 2 [Sus scrofa]				
gi|350582038|ref|XP_003124891.3|	PREDICTED: U5 small nuclear ribonucleoprotein 200 kDa helicase-like [Sus scrofa]	---	---	---	---
gi|311257842|ref|XP_003127319.1|	PREDICTED: glutamate-rich WD repeat-containing protein 1-like [Sus scrofa]	---	---	---	---
gi|335291465|ref|XP_003128033.2|	PREDICTED: UPF0587 protein C1orf123 homolog [Sus scrofa]	---	---	---	---
gi|1717797|sp|P52552.1|PRDX2_PIG	RecName: Full=Peroxiredoxin-2; AltName: Full=Thiol-specific antioxidant protein; Short=TSA; AltName: Full=Thioredoxin peroxidase 1; AltName: Full=Thioredoxin-dependent peroxide reductase 1	2.762	1.141	0.737	1.769
gi|347300176|ref|NP_001231403.1|	peroxiredoxin-2 [Sus scrofa]				
gi|264681442|ref|NP_001161116.1|	dynactin subunit 6 [Sus scrofa] >gi|262072943|dbj|BAI47779.1| dynactin 6 [Sus scrofa]	---	---	---	---
gi|31074212|gb|AAP37947.1|	cell cycle progression 2 protein [Sus scrofa]	---	---	---	---
gi|178056484|ref|NP_001116573.1|	protein TBRG4 [Sus scrofa] >gi|31074402|gb|AAP37946.1| cell cycle progression 2 protein [Sus scrofa]				
gi|124053394|sp|Q7YS91.2|TBRG4_PIG	RecName: Full=Protein TBRG4; AltName: Full=Cell cycle progression protein 2; AltName: Full=Transforming growth factor beta				

	regulator 4				
gi|335310523|ref|XP_003362072.1|	PREDICTED: protein TBRG4-like, partial [Sus scrofa]				
gi|350593043|ref|XP_001929355.3|	PREDICTED: alpha-internexin [Sus scrofa]	0.589	0.71	0.753	0.56
gi|350583051|ref|XP_003125610.3|	PREDICTED: protein virilizer homolog [Sus scrofa]	---	---	---	---
gi|311277128|ref|XP_003135516.1|	PREDICTED: high mobility group protein B3-like [Sus scrofa]	---	---	---	---
gi|350588037|ref|XP_003482541.1|	PREDICTED: high mobility group protein B3-like [Sus scrofa] >gi|350588033|ref|XP_003482539.1| PREDICTED: high mobility group protein B3-like [Sus scrofa]				
gi|350594590|ref|XP_003483925.1|	PREDICTED: golgin subfamily A member 7-like isoform 1 [Sus scrofa] >gi|350594586|ref|XP_003483923.1| PREDICTED: golgin subfamily A member 7 [Sus scrofa] >gi|350594592|ref|XP_003483926.1| PREDICTED: golgin subfamily A member 7-like isoform 2 [Sus scr	---	---	---	---
gi|350583523|ref|XP_003481536.1|	PREDICTED: putative helicase MOV-10 [Sus scrofa]	---	---	---	---
gi|350580612|ref|XP_003123174.3|	PREDICTED: cdc42-interacting protein 4-like isoform 1 [Sus scrofa]	---	---	---	---
gi|343478257|ref|NP_001230394.1|	peptidase M20 domain containing 1 [Sus scrofa]	---	---	---	---
gi|335307003|ref|XP_001926406.3|	PREDICTED: LanC lantibiotic synthetase component C-like 1 [Sus scrofa]	2.656	0.591	0.618	1.642
gi|298160936|ref|NP_001177146.1|	transmembrane protein C9orf46 [Sus scrofa]	0.545	0.35	0.364	0.601
gi|350595661|ref|XP_003360337.2|	PREDICTED: RNA-binding protein 10-like, partial [Sus scrofa]	---	---	---	---
gi|350590442|ref|XP_003483060.1|	PREDICTED: epsin-3-like [Sus scrofa] >gi|311267546|ref|XP_003131620.1| PREDICTED: epsin-3 [Sus scrofa]	---	---	---	---
gi|350578717|ref|XP_003121581.3|	PREDICTED: eukaryotic translation initiation factor 3 subunit J-like	---	---	---	---

	isoform 1 [Sus scrofa]				
gi|350592596|ref|XP_003133009.3|	PREDICTED: merlin isoform 1 [Sus scrofa]	---	---	---	---
gi|350592600|ref|XP_003483495.1|	PREDICTED: merlin [Sus scrofa]				
gi|350592602|ref|XP_003483496.1|	PREDICTED: merlin [Sus scrofa]				
gi|350592606|ref|XP_003483497.1|	PREDICTED: merlin [Sus scrofa]				
gi|350592598|ref|XP_003483494.1|	PREDICTED: merlin [Sus scrofa]				
gi|350592604|ref|XP_003133012.3|	PREDICTED: merlin isoform 4 [Sus scrofa]				
gi|311251005|ref|XP_003124395.1|	PREDICTED: TSC22 domain family protein 4-like [Sus scrofa] >gi|311251027|ref|XP_003124405.1| PREDICTED: TSC22 domain family protein 4-like [Sus scrofa]	---	---	---	---
gi|350529368|ref|NP_001231920.1|	synaptojanin 2 binding protein [Sus scrofa]	---	---	---	---
gi|350584802|ref|XP_003481825.1|	PREDICTED: neighbor of COX4-like [Sus scrofa]	---	---	---	---
gi|350584804|ref|XP_003355777.2|	PREDICTED: neighbor of COX4-like, partial [Sus scrofa]				
gi|350591308|ref|XP_003132335.3|	PREDICTED: bis(5~-adenosyl)-triphosphatase-like [Sus scrofa]	---	---	---	---
gi|34978340|sp|P36887.4|KAPCA_PIG	RecName: Full=cAMP-dependent protein kinase catalytic subunit alpha; Short=PKA C-alpha	---	---	---	---
gi|190589912|gb|ACE79215.1|	cyclic AMP-dependent protein kinase A catalytic subunit [Sus scrofa]				
gi|334878385|pdb|1CDK|A	Chain A, Camp-Dependent Protein Kinase Catalytic Subunit (E.C.2.7.1.37) (Protein Kinase A) Complexed With Protein Kinase Inhibitor Peptide Fragment 5-24 (Pki(5-24) Isoelectric Variant Ca) And Mn2+ Adenylyl Imidodiphosphate (Mnamp-Pnp) At Ph 5.6 And				
gi|305677665|pdb|3AGM|A	Chain A, Complex Of Pka With The				

	Bisubstrate Protein Kinase Inhibitor Arc-670				
gi|334878402|pdb|1CTP|E	Chain E, Structure Of The Mammalian Catalytic Subunit Of Camp-Dependent Protein Kinase And An Inhibitor Peptide Displays An Open Conformation				
gi|157838448|pdb|1CMK|E	Chain E, Crystal Structures Of The Myristylated Catalytic Subunit Of Camp- Dependent Protein Kinase Reveal Open And Closed Conformations				
gi|311248983|ref|XP_003123401.1|	PREDICTED: cAMP-dependent protein kinase catalytic subunit alpha isoform 1 [Sus scrofa]				
gi|335282773|ref|XP_003354151.1|	PREDICTED: cAMP-dependent protein kinase catalytic subunit alpha [Sus scrofa]				
gi|311250750|ref|XP_003124275.1|	PREDICTED: 26S proteasome non-ATPase regulatory subunit 13-like [Sus scrofa]	---	---	---	---
gi|194039474|ref|XP_001924848.1|	PREDICTED: DNA replication licensing factor MCM3 [Sus scrofa]	---	---	---	---
gi|350534920|ref|NP_001233134.1|	interferon-induced protein 44 [Sus scrofa] >gi|332113311|gb|AEE02032.1| interferon-inducible microtubular aggregate protein 44 [Sus scrofa]	---	---	---	---
gi|346716263|ref|NP_001231270.1|	protein tyrosine phosphatase type IVA 2 [Sus scrofa]	---	---	---	---
gi|335308977|ref|XP_003361445.1|	PREDICTED: cytoplasmic FMR1-interacting protein 1-like, partial [Sus scrofa]	---	---	---	---
gi|350594468|ref|XP_003134162.3|	PREDICTED: cytoplasmic FMR1-interacting protein 2-like [Sus scrofa]				
gi|38569755|gb|AAR24396.1|	vimentin [Sus scrofa]	---	---	---	---
gi|346716275|ref|NP_001231275.1|	dnaJ homolog subfamily B member 11 precursor [Sus scrofa]	---	---	---	---
gi|350594138|ref|XP_001927044.4|	PREDICTED: signal transducing adapter molecule 2 [Sus scrofa]	---	---	---	---
gi|231467|sp|P29700.1|FETUA_PIG	RecName: Full=Alpha-2-HS-glycoprotein;	---	---	---	---

	AltName: Full=Fetuin-A; Flags: Precursor >gi|3980229|emb|CAA39498.1| fetuin [Sus scrofa]				
gi|311269753|ref|XP_003132624.1|	PREDICTED: alpha-2-HS-glycoprotein [Sus scrofa]				
gi|122131855|sp|Q06AB3.1|UCHL3_PIG	RecName: Full=Ubiquitin carboxyl-terminal hydrolase isozyme L3; Short=UCH-L3; AltName: Full=Ubiquitin thioesterase L3 >gi|116175277|ref|NP_001070695.1| ubiquitin carboxyl-terminal hydrolase isozyme L3 [Sus scrofa] >gi|115371725|gb|ABI96187.1| UCHL3	---	---	---	---
gi|350583835|ref|XP_003355399.2|	PREDICTED: ADP-ribosylation factor-binding protein GGA1 [Sus scrofa]	---	---	---	---
gi|350578554|ref|XP_003480390.1|	PREDICTED: talin-2 [Sus scrofa]	---	---	---	---
gi|343403848|ref|NP_001230297.1|	phosphopantothenoylcysteine decarboxylase [Sus scrofa]	---	---	---	---
gi|311265237|ref|XP_003130554.1|	PREDICTED: LOW QUALITY PROTEIN: rab3 GTPase-activating protein non-catalytic subunit-like [Sus scrofa]	---	---	---	---
gi|350587371|ref|XP_003482396.1|	PREDICTED: cytosol aminopeptidase-like [Sus scrofa]	2.89	2.142	2.61	1.252
gi|350596353|ref|XP_003361080.2|	PREDICTED: gamma-soluble NSF attachment protein-like [Sus scrofa]	0.767	0.636	0.846	0.758
gi|38569721|gb|AAR24381.1|	isopentyl-diphosphate delta isomerase [Sus scrofa]	---	---	---	---
gi|350589674|ref|XP_003482895.1|	PREDICTED: isopentenyl-diphosphate Delta-isomerase 1-like [Sus scrofa] >gi|335296666|ref|XP_003357836.1| PREDICTED: isopentenyl-diphosphate Delta-isomerase 1-like [Sus scrofa]				
gi|350587135|ref|XP_003482353.1|	PREDICTED: cleavage and polyadenylation specificity factor subunit 2-like [Sus scrofa]	---	---	---	---
gi|194034586|ref|XP_001925948.1|	PREDICTED: 3-ketodihydrosphingosine	---	---	---	---

<table>
<tr><td></td><td>reductase-like [Sus scrofa]</td><td></td><td></td><td></td><td></td></tr>
<tr><td>gi|297591981|ref|NP_001172068.1|</td><td>sialic acid synthase [Sus scrofa] >gi|262204906|dbj|BAI48033.1| N-acetylneuraminic acid synthase [Sus scrofa]</td><td>---</td><td>---</td><td>---</td><td>---</td></tr>
<tr><td>gi|311276241|ref|XP_003135104.1|</td><td>PREDICTED: NADH dehydrogenase [ubiquinone] 1 beta subcomplex subunit 11, mitochondrial-like isoform 1 [Sus scrofa]</td><td>---</td><td>---</td><td>---</td><td>---</td></tr>
<tr><td>gi|311276243|ref|XP_003135105.1|</td><td>PREDICTED: NADH dehydrogenase [ubiquinone] 1 beta subcomplex subunit 11, mitochondrial-like isoform 2 [Sus scrofa]</td><td></td><td></td><td></td><td></td></tr>
<tr><td>gi|335284154|ref|XP_003354525.1|</td><td>PREDICTED: solute carrier family 12 member 9-like [Sus scrofa]</td><td>---</td><td>---</td><td>---</td><td>---</td></tr>
<tr><td>gi|335298303|ref|XP_003131887.2|</td><td>PREDICTED: protein KIAA0664-like [Sus scrofa]</td><td>0.999</td><td>1.392</td><td>1.554</td><td>1.051</td></tr>
<tr><td>gi|311276865|ref|XP_003135390.1|</td><td>PREDICTED: 60S ribosomal protein L39-like [Sus scrofa] >gi|335306463|ref|XP_003360477.1| PREDICTED: 60S ribosomal protein L39-like [Sus scrofa] >gi|335284542|ref|XP_003354632.1| PREDICTED: 60S ribosomal protein L39-like [Sus scrofa] >gi|311247842|re</td><td>0.308</td><td>0.286</td><td>0.279</td><td>0.265</td></tr>
<tr><td>gi|335306465|ref|XP_003360478.1|</td><td>PREDICTED: 60S ribosomal protein L39-like [Sus scrofa]</td><td></td><td></td><td></td><td></td></tr>
<tr><td>gi|357580498|sp|C5HGF3.1|TMCO1_PIG</td><td>RecName: Full=Transmembrane and coiled-coil domains protein 1 >gi|262399400|ref|NP_001161057.1| transmembrane and coiled-coil domains protein 1 precursor [Sus scrofa] >gi|225200247|gb|ACN82430.1| transmembrane and coiled-coil domains 1 [Sus scrofa]</td><td>---</td><td>---</td><td>---</td><td>---</td></tr>
<tr><td>gi|350585688|ref|XP_003482025.1|</td><td>PREDICTED: LOW QUALITY PROTEIN: protein RCC2 [Sus scrofa]</td><td>---</td><td>---</td><td>---</td><td>---</td></tr>
<tr><td>gi|270289756|ref|NP_001161893.1|</td><td>endophilin-B1 [Sus scrofa] >gi|262263199|dbj|BAI48102.1| SH3-domain GRB2-like endophilin B1 [Sus scrofa]</td><td>---</td><td>---</td><td>---</td><td>---</td></tr>
</table>

gi|346986269|ref|NP_001231292.1|	interferon-induced protein with tetratricopeptide repeats 1 [Sus scrofa]	---	---	---	---
gi|311274743|ref|XP_003134451.1|	PREDICTED: RNA-binding protein Raly isoform 2 [Sus scrofa]	0.735	0.569	0.566	0.682
gi|335304688|ref|XP_003359995.1|	PREDICTED: RNA-binding protein Raly [Sus scrofa] >gi|311274741|ref|XP_001929139.2| PREDICTED: RNA-binding protein Raly isoform 1 [Sus scrofa]				
gi|66866411|gb|AAY57853.1|	DJ-1 protein, partial [Sus scrofa]	---	---	---	---
gi|194043105|ref|XP_001929229.1|	PREDICTED: cob(I)yrinic acid a,c-diamide adenosyltransferase, mitochondrial isoform 1 [Sus scrofa]	---	---	---	---
gi|311270785|ref|XP_003132971.1|	PREDICTED: cob(I)yrinic acid a,c-diamide adenosyltransferase, mitochondrial isoform 2 [Sus scrofa]				
gi|350596216|ref|XP_003360918.2|	PREDICTED: sorting nexin-2, partial [Sus scrofa]	---	---	---	---
gi|311272760|ref|XP_001926651.2|	PREDICTED: zinc finger CCCH domain-containing protein 15 [Sus scrofa]	---	---	---	---
gi|11055649|gb|AAG28195.1|AF304201_6	ATPase 6 [Sus scrofa]	---	---	---	---
gi|45826189|gb|AAS77709.1|	ATP synthase F0 subunit 6 [Sus scrofa]				
gi|209166162|gb|ACI41537.1|	ATP synthase F0 subunit 6 [Sus scrofa]				
gi|118135679|gb|ABK62690.1|	ATPase subunit 6 [Sus scrofa]				
gi|223976084|ref|YP_002600782.1|	ATP synthase F0 subunit 6 [Sus scrofa domesticus] >gi|5835868|ref|NP_008639.1|ATP6_15069 ATP synthase F0 subunit 6 [Sus scrofa] >gi|347448701|gb|AEO93016.1| ATP synthase F0 subunit 6 (mitochondrion) [Sus scrofa] >gi|347448645|gb|AEO92964.1| ATP synt				
gi|251829655|gb|ACT21208.1|	ATP synthase F0 subunit 6 [Sus scrofa]				

gi|33320871|gb|AAQ06154.1|	ATPase subunit 6 [Sus scrofa]				
gi|33320927|gb|AAQ06206.1|	ATPase subunit 6 [Sus scrofa] >gi|33320689|gb|AAQ05985.1| ATPase subunit 6 [Sus scrofa]				
gi|145315588|gb|ABP63137.1|	ATP synthase F0 subunit 6 [Sus scrofa]				
gi|145315644|gb|ABP63189.1|	ATP synthase F0 subunit 6 [Sus scrofa] >gi|145315630|gb|ABP63176.1| ATP synthase F0 subunit 6 [Sus scrofa]				
gi|157056526|gb|ABV02158.1|	ATP synthase F0 subunit 6 [Sus scrofa]				
gi|11055677|gb|AAG28221.1|AF304203_6	ATPase 6 [Sus scrofa]				
gi|145315686|gb|ABP63228.1|	ATP synthase F0 subunit 6 [Sus scrofa]				
gi|290750183|gb|ADD52030.1|	ATP synthase subunit 6 [Sus scrofa]				
gi|145315658|gb|ABP63202.1|	ATP synthase F0 subunit 6 [Sus scrofa]				
gi|342316094|gb|ABF56565.2|	ATPase subunit 6 [Sus scrofa]				
gi|312233369|ref|YP_004021580.1|	ATP synthase F0 subunit 6 [Sus scrofa taiwanensis] >gi|347448729|gb|AEO93042.1| ATP synthase F0 subunit 6 (mitochondrion) [Sus scrofa] >gi|347448673|gb|AEO92990.1| ATP synthase F0 subunit 6 (mitochondrion) [Sus scrofa] >gi|347448743|gb|AEO93055.1| A				
gi|145315448|gb|ABP63007.1|	ATP synthase F0 subunit 6 [Sus scrofa]				
gi|37719398|gb|AAR01785.1|	ATP synthase F0 subunit 6 [Sus scrofa]				
gi|209166176|gb|ACI41550.1|	ATP synthase F0 subunit 6 [Sus scrofa]				
gi|80972998|gb|ABB53229.1|	ATP synthase F0 subunit 6 [Sus scrofa]				
gi|209166246|gb|ACI41615.1|	ATP synthase F0 subunit 6 [Sus scrofa] >gi|209166218|gb|ACI41589.				

	1| ATP synthase F0 subunit 6 [Sus scrofa] >gi|209166134|gb|ACI41511.1| ATP synthase F0 subunit 6 [Sus scrofa] >gi|209166106|gb|ACI41485.1| ATP synthase F0 subunit 6 [Sus scrofa] >gi|20				
gi|76262555|gb|ABA41441.1|	ATP synthase F0 subunit 6 [Sus scrofa]				
gi|157056541|gb|ABV02172.1|	ATP synthase F0 subunit 6 [Sus scrofa]				
gi|45826217|gb|AAS77735.1|	ATP synthase F0 subunit 6 [Sus scrofa]				
gi|315057191|gb|ADT71729.1|	ATP synthase subunit 6 [Sus scrofa]				
gi|94981326|gb|ABF49554.1|	ATPase subunit 6 [Sus scrofa] >gi|124249813|gb|ABM92886.1| ATPase subunit 6 [Sus scrofa]				
gi|83779137|gb|ABC47381.1|	ATP synthase F0 subunit 6 [Sus scrofa]				
gi|311257281|ref|XP_003127042.1|	PREDICTED: dnaJ homolog subfamily A member 2-like [Sus scrofa]	---	---	---	---
gi|350587021|ref|XP_003356786.2|	PREDICTED: MAGUK p55 subfamily member 5 [Sus scrofa]	---	---	---	---
gi|350591244|ref|XP_003483235.1|	PREDICTED: RNA-binding protein 6 isoform 2 [Sus scrofa]	---	---	---	---
gi|350591246|ref|XP_003132268.3|	PREDICTED: RNA-binding protein 6 isoform 1 [Sus scrofa]				
gi|350592445|ref|XP_003483465.1|	PREDICTED: hypothetical protein LOC100154492 [Sus scrofa]	---	---	---	---
gi|350592447|ref|XP_003483466.1|	PREDICTED: hypothetical protein LOC100154492 [Sus scrofa]				
gi|335304534|ref|XP_003134355.2|	PREDICTED: 5~-3~ exoribonuclease 2 [Sus scrofa]	---	---	---	---
gi|194034833|ref|XP_001928153.1|	PREDICTED: microfibrillar-associated protein 1 [Sus scrofa]	0.905	1.801	1.48	1.208
gi|350578774|ref|XP_003480447.1|	PREDICTED: EH domain-containing protein 4, partial [Sus scrofa]	---	---	---	---
gi|350585748|ref|XP_003127751.3|	PREDICTED: hypothetical protein LOC100521629 isoform 1 [Sus scrofa]	0.638	0.605	0.671	0.624

gi|350585750|ref|XP_003482044.1|	PREDICTED: hypothetical protein LOC100521629 isoform 2 [Sus scrofa]				
gi|350585912|ref|XP_003127855.3|	PREDICTED: microtubule-actin cross-linking factor 1 isoform 1, partial [Sus scrofa]	---	---	---	---
gi|6166237|sp|O19175.1|KC1A_PIG	RecName: Full=Casein kinase I isoform alpha; Short=CKI-alpha; AltName: Full=CK1	---	---	---	---
gi|311250475|ref|XP_003124136.1|	PREDICTED: casein kinase I isoform alpha isoform 1 [Sus scrofa]				
gi|350582213|ref|XP_003481224.1|	PREDICTED: 60S ribosomal protein L7a-like [Sus scrofa] >gi|350582215|ref|XP_003481225.1| PREDICTED: 60S ribosomal protein L7a-like [Sus scrofa] >gi|335285208|ref|XP_003125024.2| PREDICTED: 60S ribosomal protein L7a-like isoform 1 [Sus scrofa]	---	---	---	---
gi|311266801|ref|XP_003131259.1|	PREDICTED: nuclear pore complex protein Nup85-like [Sus scrofa]	---	---	---	---
gi|350592132|ref|XP_003358973.2|	PREDICTED: splicing factor, arginine/serine-rich 15 [Sus scrofa]	---	---	---	---
gi|350578880|ref|XP_003480471.1|	PREDICTED: tight junction protein ZO-1-like, partial [Sus scrofa]	---	---	---	---
gi|16506695|gb|AAL23907.1|AF339885_1	mannose-6-phosphate/insulin-like growth factor II receptor [Sus scrofa]	---	---	---	---
gi|350577990|ref|XP_003480266.1|	PREDICTED: cation-independent mannose-6-phosphate receptor-like [Sus scrofa]				
gi|347300273|ref|NP_001231402.1|	cation-independent mannose-6-phosphate receptor precursor [Sus scrofa] >gi|334853107|gb|AEH05671.1| insulin-like growth factor 2 receptor precursor [Sus scrofa]				
gi|374305556|gb|AEZ06359.1|	insulin-like growth factor II receptor [Sus scrofa]				
gi|350588656|ref|XP_003357409.2|	PREDICTED: amyloid beta (A4) precursor-like protein 2 [Sus scrofa]	---	---	---	---
gi|311259534|ref|XP_003128	PREDICTED:	---	---	---	---

149.1|	phosphopantothenate--cysteine ligase-like [Sus scrofa]				
gi|194038370|ref|XP_001929110.1|	PREDICTED: poly(A) polymerase alpha isoform 1 [Sus scrofa]	0.607	1.121	0.874	0.836
gi|343403833|ref|NP_001230293.1|	coiled-coil domain containing 167 [Sus scrofa]	---	---	---	---
gi|350586346|ref|XP_003482166.1|	PREDICTED: transmembrane and coiled-coil domain-containing protein C6orf129-like, partial [Sus scrofa]				
gi|350594977|ref|XP_001929182.3|	PREDICTED: nuclear receptor coactivator 5 [Sus scrofa]	---	---	---	---
gi|350596467|ref|XP_003129178.3|	PREDICTED: fibrinogen alpha chain, partial [Sus scrofa]	---	---	---	---
gi|346716281|ref|NP_001231278.1|	eukaryotic translation initiation factor 1A, Y-linked [Sus scrofa]	---	---	---	---
gi|350586900|ref|XP_003128597.3|	PREDICTED: transmembrane 9 superfamily member 1 [Sus scrofa]	---	---	---	---
gi|305855210|ref|NP_001182286.1|	DNA mismatch repair protein Msh2 [Sus scrofa] >gi|285818416|gb|ADC38881.1| MutS-like protein 2 [Sus scrofa]	---	---	---	---
gi|335299797|ref|XP_003358684.1|	PREDICTED: 1-phosphatidylinositol-4,5-bisphosphate phosphodiesterase eta-1 [Sus scrofa]	---	---	---	---
gi|350587061|ref|XP_003482336.1|	PREDICTED: iron-sulfur cluster assembly 2 homolog, mitochondrial-like [Sus scrofa]	---	---	---	---
gi|217314881|gb|ACK36975.1|	phosphatidic acid phosphatase type 2C isoform 3 [Sus scrofa]	---	---	---	---
gi|219522016|ref|NP_001137195.1|	lipid phosphate phosphohydrolase 2 [Sus scrofa] >gi|217314877|gb|ACK36973.1| phosphatidic acid phosphatase type 2C isoform 1 [Sus scrofa]				
gi|346421308|ref|NP_001231011.1|	trans-2-enoyl-CoA reductase, mitochondrial [Sus scrofa]	---	---	---	---
gi|113205616|ref|NP_001038008.1|	60S ribosomal protein L10 [Sus scrofa] >gi|85681889|sp|Q29195.3|RL10_PIG RecName: Full=60S ribosomal protein L10; AltName: Full=Protein QM	---	---	---	---

	homolog >gi|56384249|gb|AAV85773.1| ribosomal protein L10 [Sus scrofa]				
gi|311245451|ref|XP_003121835.1|	PREDICTED: 60S ribosomal protein L10-like [Sus scrofa]				
gi|350579589|ref|XP_003353682.2|	PREDICTED: erythrocyte band 7 integral membrane protein-like [Sus scrofa]	---	---	---	---
gi|350578478|ref|XP_003121483.3|	PREDICTED: myosin-Vb-like, partial [Sus scrofa]	---	---	---	---
gi|335282227|ref|XP_003123029.2|	PREDICTED: switch-associated protein 70-like [Sus scrofa]	---	---	---	---
gi|147901025|ref|NP_001090949.1|	40S ribosomal protein S21 [Sus scrofa] >gi|52783791|sp|P63221.1|RS21_PIG RecName: Full=40S ribosomal protein S21 >gi|311261595|ref|XP_003128784.1| PREDICTED: 40S ribosomal protein S21-like isoform 1 [Sus scrofa] >gi|117661139|gb|ABK55656.1| RPS21 [S	1.084	1.406	0.892	1.171
gi|335281869|ref|XP_003353912.1|	PREDICTED: FACT complex subunit SSRP1 [Sus scrofa]	---	---	---	---
gi|2833354|sp|Q29223.2|RL34_PIG	RecName: Full=60S ribosomal protein L34	0.879	0.523	0.566	0.941
gi|311262701|ref|XP_003129308.1|	PREDICTED: 60S ribosomal protein L34-like isoform 2 [Sus scrofa] >gi|311262697|ref|XP_003129307.1| PREDICTED: 60S ribosomal protein L34-like isoform 1 [Sus scrofa] >gi|311262699|ref|XP_003129309.1| PREDICTED: 60S ribosomal protein L34-like isoform 3				
gi|350592681|ref|XP_003483514.1|	PREDICTED: ubiquitin fusion degradation protein 1 homolog, partial [Sus scrofa]	---	---	---	---
gi|297632410|ref|NP_001172089.1|	ADP-ribosylation-like factor 6 interacting protein 4 isoform a [Sus scrofa]	---	---	---	---
gi|297632413|ref|NP_001172090.1|	ADP-ribosylation-like factor 6 interacting protein 4 isoform b [Sus scrofa]				
gi|335298923|ref|XP_003132	PREDICTED: anoctamin-10 [Sus	---	---	---	---

196.2|	scrofa]				
gi|350591798|ref|XP_003483335.1|	PREDICTED: replication factor C (activator 1) 4, 37kDa [Sus scrofa]	---	---	---	---
gi|335308196|ref|XP_003361137.1|	PREDICTED: cell division cycle protein 123 homolog [Sus scrofa]	---	---	---	---
gi|345091069|ref|NP_001230752.1|	cell division cycle 123 homolog [Sus scrofa]				
gi|350592375|ref|XP_001927668.3|	PREDICTED: LOW QUALITY PROTEIN: probable ATP-dependent RNA helicase DDX60 [Sus scrofa]	---	---	---	---
gi|335293236|ref|XP_003356909.1|	PREDICTED: ADP-ribosyl cyclase 2-like [Sus scrofa]	---	---	---	---
gi|311273371|ref|XP_003133833.1|	PREDICTED: NADH dehydrogenase [ubiquinone] 1 alpha subcomplex subunit 10, mitochondrial-like [Sus scrofa]	0.942	0.553	0.566	0.856
gi|350594832|ref|XP_003134443.3|	PREDICTED: uncharacterized protein C20orf112 homolog [Sus scrofa]	0.779	0.697	0.945	0.869
gi|343780920|ref|NP_001230477.1|	polyadenylate-binding protein 2 [Sus scrofa]	---	---	---	---
gi|335293623|ref|XP_003357010.1|	PREDICTED: immunoglobulin J chain-like isoform 2 [Sus scrofa] >gi|335293621|ref|XP_003357009.1| PREDICTED: immunoglobulin J chain-like isoform 1 [Sus scrofa]	---	---	---	---
gi|350579839|ref|XP_003353781.2|	PREDICTED: 7-dehydrocholesterol reductase-like [Sus scrofa]	---	---	---	---
gi|194044529|ref|XP_001925282.1|	PREDICTED: RNA-binding protein 39 isoform 2 [Sus scrofa]	---	---	---	---
gi|335304749|ref|XP_003360015.1|	PREDICTED: RNA-binding protein 39 [Sus scrofa]				
gi|335304742|ref|XP_003360012.1|	PREDICTED: RNA-binding protein 39 [Sus scrofa]				
gi|335304745|ref|XP_003360013.1|	PREDICTED: RNA-binding protein 39 [Sus scrofa]				
gi|335280058|ref|XP_003121747.2|	PREDICTED: vacuolar protein sorting-associated protein 4B-like [Sus scrofa]	---	---	---	---
gi|335303022|ref|XP_003359611.1|	PREDICTED: LOW QUALITY PROTEIN: WAS/WASL-interacting	---	---	---	---

	protein family member 1-like [Sus scrofa]				
gi|311262216|ref|XP_003129070.1|	PREDICTED: LOW QUALITY PROTEIN: signal recognition particle 72 kDa protein-like [Sus scrofa]	1.184	1.064	1.23	1.278
gi|311270305|ref|XP_001926790.2|	PREDICTED: exportin-7 [Sus scrofa]	---	---	---	---
gi|335308535|ref|XP_003361271.1|	PREDICTED: N(2),N(2)-dimethylguanosine tRNA methyltransferase-like, partial [Sus scrofa]	0.874	1.076	1.253	1.203
gi|415463|gb|AAB28132.1|	cytochrome c oxidase subunit VIIc {N-terminal} [swine, intestine, Peptide, 47 aa]	---	---	---	---
gi|91694325|gb|ABE41807.1|	cytochrome c oxidase subunit VIIc [Sus scrofa] >gi|90819632|gb|ABD98461.1| cytochrome c oxidase subunit VIIc [Sus scrofa]				
gi|110278917|sp|Q1W0Y2.1|COX7C_PIG	RecName: Full=Cytochrome c oxidase subunit 7C, mitochondrial; AltName: Full=Cytochrome c oxidase polypeptide VIIc; Flags: Precursor >gi|147900047|ref|NP_001090943.1| cytochrome c oxidase subunit 7C, mitochondrial precursor [Sus scrofa] >gi|117660817				
gi|335309123|ref|XP_003361503.1|	PREDICTED: cytochrome c oxidase subunit 7C, mitochondrial-like [Sus scrofa]				
gi|350578030|ref|XP_003121180.3|	PREDICTED: cytochrome c oxidase subunit 7C, mitochondrial-like [Sus scrofa]				
gi|194039547|ref|XP_001928898.1|	PREDICTED: LDLR chaperone MESD [Sus scrofa]	---	---	---	---
gi|350593111|ref|XP_001927517.4|	PREDICTED: SEC23-interacting protein [Sus scrofa]	---	---	---	---
gi|350581370|ref|XP_003124307.3|	PREDICTED: MICAL-like protein 2 [Sus scrofa]	0.502	1.128	1.227	0.912
gi|350592753|ref|XP_001929357.4|	PREDICTED: ankyrin-3 isoform 1 [Sus scrofa]	---	---	---	---
gi|350592755|ref|XP_003483	PREDICTED: ankyrin-3 isoform 2				

528.1|	[Sus scrofa]				
gi|198282001|ref|NP_001123683.1|	transcription factor A, mitochondrial precursor [Sus scrofa] >gi|75052621|sp|Q5D144.1|TFAM_PIG RecName: Full=Transcription factor A, mitochondrial; Short=mtTFA; Flags: Precursor >gi|60326706|gb|AAX18878.1| mitochondrial transcription factor A [Sus s	0.487	0.264	0.384	0.359
gi|194042017|ref|XP_001926614.1|	PREDICTED: structural maintenance of chromosomes protein 3 [Sus scrofa]	---	---	---	---
gi|350584221|ref|XP_003126398.3|	PREDICTED: exportin-T [Sus scrofa]	---	---	---	---
gi|5835872|ref|NP_008643.1|ND4_15069	NADH dehydrogenase subunit 4 [Sus scrofa] >gi|20455496|sp|O79881.2|NU4M_PIG RecName: Full=NADH-ubiquinone oxidoreductase chain 4; AltName: Full=NADH dehydrogenase subunit 4 >gi|4958961|gb|AAD34194.1|AF034253_10 NADH dehydrogenase subunit 4 [Sus scro	---	---	---	---
gi|76262531|gb|ABA41419.1|	NADH dehydrogenase subunit 4 [Sus scrofa]				
gi|251829659|gb|ACT21212.1|	NADH dehydrogenase subunit 4 [Sus scrofa]				
gi|45826193|gb|AAS77713.1|	NADH dehydrogenase subunit 4 [Sus scrofa]				
gi|145315786|gb|ABP63321.1|	NADH dehydrogenase subunit 4 [Sus scrofa]				
gi|118135683|gb|ABK62694.1|	NADH dehydrogenase subunit 4 [Sus scrofa]				
gi|95116714|gb|ABF56569.1|	NADH dehydrogenase subunit 4 [Sus scrofa]				
gi|312233373|ref|YP_004021584.1|	NADH dehydrogenase subunit 4 [Sus scrofa taiwanensis] >gi|347448733|gb|AEO93046.1| NADH dehydrogenase subunit 4 (mitochondrion) [Sus scrofa] >gi|347448677|gb|AEO92994.1| NADH dehydrogenase subunit 4				

	(mitochondrion) [Sus scrofa] >gi|347448747|gb|AEO9				
gi|223976088|ref|YP_002600786.1|	NADH dehydrogenase subunit 4 [Sus scrofa domesticus] >gi|347448705|gb|AEO93020.1| NADH dehydrogenase subunit 4 (mitochondrion) [Sus scrofa] >gi|347448649|gb|AEO92968.1| NADH dehydrogenase subunit 4 (mitochondrion) [Sus scrofa] >gi|347448621|gb|AEO92				
gi|45826179|gb|AAS77700.1|	NADH dehydrogenase subunit 4 [Sus scrofa]				
gi|94981330|gb|ABF49558.1|	NADH dehydrogenase subunit 4 [Sus scrofa]				
gi|76262559|gb|ABA41445.1|	NADH dehydrogenase subunit 4 [Sus scrofa]				
gi|162279942|ref|YP_001023772.2|	NADH dehydrogenase subunit 4 [Phacochoerus africanus]				
gi|157056530|gb|ABV02162.1|	NADH dehydrogenase subunit 4 [Sus scrofa]				
gi|145315774|gb|ABP63310.1|	NADH dehydrogenase subunit 4 [Sus scrofa]				
gi|162423277|gb|ABX89418.1|	NADH dehydrogenase subunit 4 [Sus scrofa]				
gi|76262545|gb|ABA41432.1|	NADH dehydrogenase subunit 4 [Sus scrofa]				
gi|37962878|gb|AAR05791.1|	NADH dehydrogenase subunit 4 [Sus scrofa]				
gi|80973002|gb|ABB53233.1|	NADH dehydrogenase subunit 4 [Sus scrofa]				
gi|238625892|gb|ACR48218.1|	NADH dehydrogenase subunit 4 [Sus scrofa]				
gi|159162014|gb|ABM92890.2|	NADH dehydrogenase subunit 4 [Sus scrofa]				
gi|45826207|gb|AAS77726.1|	NADH dehydrogenase subunit 4 [Sus scrofa]				
gi|37719402|gb|AAR01789.1|	NADH dehydrogenase subunit 4 [Sus scrofa]				
gi|83779155|gb|ABC47398.1|	NADH dehydrogenase subunit 4 [Sus scrofa]				
gi|45826221|gb|AAS77739.1|	NADH dehydrogenase subunit 4 [Sus				

	scrofa]				
gi|161777647|gb|ABX79190.1|	N-acetylneuraminic acid hydroxylase variant 3 [Sus scrofa]	---	---	---	---
gi|73918051|sp|O19074.1|CMAH_PIG	RecName: Full=Cytidine monophosphate-N-acetylneuraminic acid hydroxylase; Short=CMP-N-acetylneuraminic acid hydroxylase; AltName: Full=CMP-N-acetylneuraminate monooxygenase; AltName: Full=CMP-Neu5Ac hydroxylase; AltName: Full=CMP-NeuAc hydroxylase >				
gi|163915137|ref|NP_001106486.1|	cytidine monophosphate-N-acetylneuraminic acid hydroxylase [Sus scrofa] >gi|158726707|gb|ABW80749.1| CMP-N-acetylneuraminic acid hydroxylase [Sus scrofa]				
gi|350585957|ref|XP_003482084.1|	PREDICTED: LOW QUALITY PROTEIN: AFG3-like protein 2, partial [Sus scrofa]	---	---	---	---
gi|47522752|ref|NP_999126.1|	optineurin [Sus scrofa] >gi|62286962|sp|Q7YS99.1|OPTN_PIG RecName: Full=Optineurin >gi|31324103|gb|AAP47176.1|AF513722_1 optineurin [Sus scrofa]	---	---	---	---
gi|350584686|ref|XP_003481803.1|	PREDICTED: polypeptide N-acetylgalactosaminyltransferase 4 isoform 2 [Sus scrofa]	---	---	---	---
gi|350596113|ref|XP_003360781.2|	PREDICTED: polypeptide N-acetylgalactosaminyltransferase 4-like [Sus scrofa] >gi|350584684|ref|XP_003481802.1| PREDICTED: polypeptide N-acetylgalactosaminyltransferase 4 isoform 1 [Sus scrofa]				
gi|346421398|ref|NP_001231071.1|	sorcin isoform 1 [Sus scrofa]	---	---	---	---
gi|346644814|ref|NP_001231072.1|	sorcin isoform 2 [Sus scrofa]				
gi|68534998|ref|NP_0010203	nuclear receptor coactivator 1 [Sus	---	---	---	---

99.1|	scrofa] >gi|75075065|sp|Q4PJW2.1|NCOA1_PIG RecName: Full=Nuclear receptor coactivator 1; Short=NCoA-1; AltName: Full=Steroid receptor coactivator 1; Short=SRC-1 >gi|67866953|gb|AAY82453.1| nuclear receptor coactiv				
gi|164523392|gb|ABY60786.1|	steroid receptor coactivator 1 isoform 2 [Sus scrofa]				
gi|164523390|gb|ABY60785.1|	steroid receptor coactivator 1 isoform 1 [Sus scrofa]				
gi|350578786|ref|XP_003121657.3|	PREDICTED: RNA polymerase-associated protein RTF1 homolog [Sus scrofa]	---	---	---	---
gi|350581632|ref|XP_003124588.3|	PREDICTED: hypothetical protein LOC100521783 isoform 1 [Sus scrofa]	---	---	---	---
gi|311276906|ref|XP_001924366.2|	PREDICTED: THO complex subunit 2 [Sus scrofa]	---	---	---	---
gi|350587373|ref|XP_003128918.3|	PREDICTED: condensin complex subunit 3 [Sus scrofa]	---	---	---	---
gi|194041854|ref|XP_001929564.1|	PREDICTED: biogenesis of lysosome-related organelles complex-1 subunit 2 [Sus scrofa]	---	---	---	---
gi|350595685|ref|XP_003135150.3|	PREDICTED: E3 ubiquitin-protein ligase HUWE1-like [Sus scrofa]	---	---	---	---
gi|311269675|ref|XP_003132593.1|	PREDICTED: actin-like 6A isoform 1 [Sus scrofa]	---	---	---	---
gi|343962595|ref|NP_001230634.1|	thioredoxin, mitochondrial [Sus scrofa]	1.024	1.646	1.288	1.379
gi|350591320|ref|XP_003132344.3|	PREDICTED: 26S proteasome non-ATPase regulatory subunit 6 [Sus scrofa]	1.844	1.187	0.979	1.468
gi|298160941|ref|NP_001177148.1|	pterin-4 alpha-carbinolamine dehydratase/dimerization cofactor of hepatocyte nuclear factor 1 alpha [Sus scrofa]	1.246	1.287	1.274	1.312
gi|350585782|ref|XP_003127781.3|	PREDICTED: AT-rich interactive domain-containing protein 1A, partial [Sus scrofa]	---	---	---	---
gi|38488989|tpg|DAA01973.1|	TPA_inf: RTN4-Aw [Sus scrofa]	---	---	---	---

gi|194018686|ref|NP_001123435.1|	reticulon-4 [Sus scrofa] >gi|38327590|tpg|DAA01967.1| TPA_inf: RTN4-C [Sus scrofa]				
gi|219521986|ref|NP_001137180.1|	glycerol kinase [Sus scrofa] >gi|217314913|gb|ACK36991.1| glycerol kinase [Sus scrofa]	---	---	---	---
gi|311273120|ref|XP_003133723.1|	PREDICTED: glycerol kinase-like [Sus scrofa]				
gi|311264064|ref|XP_003129981.1|	PREDICTED: Na(+)/H(+) exchange regulatory cofactor NHE-RF4 isoform 1 [Sus scrofa]	---	---	---	---
gi|311264066|ref|XP_003129982.1|	PREDICTED: Na(+)/H(+) exchange regulatory cofactor NHE-RF4 isoform 2 [Sus scrofa]				
gi|3288634|emb|CAA04598.1|	Bak protein [Sus scrofa]	---	---	---	---
gi|194040311|ref|XP_001928182.1|	PREDICTED: putative Bcl-2 homologous antagonist/killer 2 [Sus scrofa]				
gi|335294984|ref|XP_003357369.1|	PREDICTED: trehalase [Sus scrofa]	---	---	---	---
gi|221325626|ref|NP_001138314.1|	NADH dehydrogenase [ubiquinone] 1 beta subcomplex subunit 8, mitochondrial [Sus scrofa] >gi|219563064|gb|ACL27891.1| NADH dehydrogenase (ubiquinone) 1 beta subcomplex 8 [Sus scrofa]	---	---	---	---
gi|335283923|ref|XP_003354462.1|	PREDICTED: plakophilin-3-like [Sus scrofa]	---	---	---	---
gi|311268067|ref|XP_003131870.1|	PREDICTED: replication protein A 70 kDa DNA-binding subunit [Sus scrofa]	---	---	---	---
gi|335293447|ref|XP_003129021.2|	PREDICTED: ubiquitin carboxyl-terminal hydrolase 46 [Sus scrofa]	---	---	---	---
gi|335296741|ref|XP_003130951.2|	PREDICTED: ubiquitin carboxyl-terminal hydrolase 12 [Sus scrofa]				
gi|335295199|ref|XP_003357427.1|	PREDICTED: zinc finger CCCH domain-containing protein 11A isoform 2 [Sus	---	---	---	---

	scrofa] >gi|335295197|ref|XP_003357426.1| PREDICTED: zinc finger CCCH domain-containing protein 11A isoform 1 [Sus scrofa]				
gi|222136636|ref|NP_001138402.1|	nuclear transcription factor Y subunit beta [Sus scrofa]	---	---	---	---
gi|335310220|ref|XP_003361935.1|	PREDICTED: elongation factor Ts, mitochondrial-like, partial [Sus scrofa]	---	---	---	---
gi|311258370|ref|XP_003127581.1|	PREDICTED: pleckstrin homology domain-containing family G member 5 [Sus scrofa]	---	---	---	---
gi|343790928|ref|NP_001230520.1|	mini-chromosome maintenance complex-binding protein [Sus scrofa]	---	---	---	---
gi|311266774|ref|XP_003131239.1|	PREDICTED: WW domain-binding protein 2-like [Sus scrofa]	---	---	---	---
gi|345091084|ref|NP_001230758.1|	RNA binding motif protein 25 [Sus scrofa]	---	---	---	---
gi|350596600|ref|XP_003128714.3|	PREDICTED: RNA-binding protein 25, partial [Sus scrofa]				
gi|229892828|ref|NP_001153565.1|	protein arginine N-methyltransferase 5 [Sus scrofa] >gi|197692948|gb|ACH71262.1| protein arginine methyltransferase 5 [Sus scrofa]	---	---	---	---
gi|194036657|ref|XP_001928045.1|	PREDICTED: brefeldin A-inhibited guanine nucleotide-exchange protein 1 [Sus scrofa]	---	---	---	---
gi|55741851|ref|NP_001001264.1|	Wilms tumor protein homolog [Sus scrofa] >gi|3915298|sp|O62651.1|WT1_PIG RecName: Full=Wilms tumor protein homolog >gi|3132272|dbj|BAA28147.1| unnamed protein product [Sus scrofa]	---	---	---	---
gi|98283612|gb|ABF58000.1|	mitochondrial NAD+isocitrate dehydrogenase 3 beta variant 1 [Sus scrofa]	---	---	---	---
gi|98283616|gb|ABF58003.1|	mitochondrial NAD+isocitrate dehydrogenase 3 beta variant 1 [Sus scrofa]				
gi|98283613|gb|ABF58001.1|	mitochondrial NAD+isocitrate				

	dehydrogenase 3 beta variant 2 [Sus scrofa] >gi|98283614|gb|ABF58002.1| mitochondrial NAD+isocitrate dehydrogenase 3 beta variant 3 [Sus scrofa]				
gi|113205754|ref|NP_001038040.1|	isocitrate dehydrogenase [NAD] subunit beta, mitochondrial [Sus scrofa] >gi|98283620|gb|ABF58005.1| mitochondrial NAD+isocitrate dehydrogenase 3 beta variant 3 [Sus scrofa] >gi|98283618|gb|ABF58004.1| mitochondrial NAD+isocitrate dehydrogenase 3 bet				
gi|335309458|ref|XP_003361645.1|	PREDICTED: isocitrate dehydrogenase [NAD] subunit beta, mitochondrial-like [Sus scrofa]				
gi|350583819|ref|XP_003126103.3|	PREDICTED: brain-specific angiogenesis inhibitor 1-associated protein 2-like protein 2-like, partial [Sus scrofa]	---	---	---	---
gi|350584473|ref|XP_003126641.3|	PREDICTED: V-type proton ATPase subunit E 1 isoform 2 [Sus scrofa]	---	---	---	---
gi|350584475|ref|XP_003481754.1|	PREDICTED: V-type proton ATPase subunit E 1 [Sus scrofa]				
gi|39646090|emb|CAE83881.1|	unnamed protein product [Sus scrofa]	---	---	---	---
gi|47523636|ref|NP_999446.1|	complement factor H precursor [Sus scrofa] >gi|21464594|emb|CAC81999.1| complement regulator factor H [Sus scrofa]				
gi|346644842|ref|NP_001231175.1|	NOP2 nucleolar protein homolog [Sus scrofa]	---	---	---	---
gi|347922122|ref|NP_001231672.1|	NADH dehydrogenase (ubiquinone) 1, subcomplex unknown, 2, 14.5kDa [Sus scrofa] >gi|350588320|ref|XP_003482623.1| PREDICTED: NADH dehydrogenase [ubiquinone] 1 subunit C2-like isoform 1 [Sus scrofa]	---	---	---	---
gi|350588322|ref|XP_003482624.1|	PREDICTED: NADH dehydrogenase [ubiquinone] 1 subunit C2-like isoform 2 [Sus scrofa]				

gi|187470928|sp|A5A779.1|PGTA_PIG	RecName: Full=Geranylgeranyl transferase type-2 subunit alpha; AltName: Full=Geranylgeranyl transferase type II subunit alpha; AltName: Full=Rab geranyl-geranyltransferase subunit alpha; Short=Rab GG transferase alpha; Short=Rab GGTase alpha; AltNam	---	---	---	---
gi|297747277|ref|NP_001172107.1|	NADH dehydrogenase (ubiquinone) 1 alpha subcomplex, 6, 14kDa [Sus scrofa] >gi|350583744|ref|XP_003481577.1| PREDICTED: NADH dehydrogenase [ubiquinone] 1 alpha subcomplex subunit 6-like [Sus scrofa]	---	---	---	---
gi|335310969|ref|XP_001928237.3|	PREDICTED: NADH dehydrogenase [ubiquinone] 1 alpha subcomplex subunit 6-like, partial [Sus scrofa]				
gi|346986447|ref|NP_001231373.1|	ribonuclease H2 subunit A [Sus scrofa]	0.934	1.495	0.612	0.901
gi|335282567|ref|XP_003123239.2|	PREDICTED: evolutionarily conserved signaling intermediate in Toll pathway, mitochondrial-like [Sus scrofa]	---	---	---	---
gi|311267157|ref|XP_003131429.1|	PREDICTED: coiled-coil domain-containing protein 56-like [Sus scrofa]	---	---	---	---
gi|350583671|ref|XP_003125970.3|	PREDICTED: interferon-induced guanylate-binding protein 2-like isoform 2, partial [Sus scrofa]	---	---	---	---
gi|311264070|ref|XP_003129980.1|	PREDICTED: E3 ubiquitin-protein ligase CBL [Sus scrofa]	---	---	---	---
gi|335296877|ref|XP_003357884.1|	PREDICTED: cytidine and dCMP deaminase domain-containing protein 1-like [Sus scrofa]	---	---	---	---
gi|335292471|ref|XP_001927834.3|	PREDICTED: signal recognition particle 54 kDa protein isoform 1 [Sus scrofa]	---	---	---	---
gi|335292473|ref|XP_003356739.1|	PREDICTED: signal recognition particle 54 kDa protein [Sus scrofa]				
gi|350589889|ref|XP_003482942.1|	PREDICTED: TSC22 domain family protein 1-like [Sus scrofa]	---	---	---	---

gi|311260236|ref|XP_001925503.2|	PREDICTED: tapasin-like [Sus scrofa]	---	---	---	---
gi|350589164|ref|XP_003357646.2|	PREDICTED: acyl-CoA:lysophosphatidylglycerol acyltransferase 1-like [Sus scrofa]	---	---	---	---
gi|311246039|ref|XP_003122046.1|	PREDICTED: golgi-associated plant pathogenesis-related protein 1-like [Sus scrofa]	---	---	---	---
gi|350579373|ref|XP_003480598.1|	PREDICTED: golgi-associated plant pathogenesis-related protein 1-like, partial [Sus scrofa]				
gi|148222591|ref|NP_001090955.1|	ATPase inhibitor, mitochondrial precursor [Sus scrofa] >gi|148887342|sp|Q29307.2|ATIF1_PIG RecName: Full=ATPase inhibitor, mitochondrial; AltName: Full=Inhibitor of F(1)F(o)-ATPase; Short=IF(1); Short=IF1; Flags: Precursor >gi|117661240|gb|ABK55664.	---	---	---	---
gi|350590642|ref|XP_003131789.3|	PREDICTED: 26S proteasome non-ATPase regulatory subunit 11-like [Sus scrofa]	---	---	---	---
gi|335310105|ref|XP_003361889.1|	PREDICTED: acyl-CoA dehydrogenase family member 10-like, partial [Sus scrofa]	---	---	---	---
gi|148233298|ref|NP_001090954.1|	heat shock factor-binding protein 1 [Sus scrofa] >gi|117661226|gb|ABK55663.1| HSBP1 [Sus scrofa]	---	---	---	---
gi|311247522|ref|XP_003122684.1|	PREDICTED: succinate dehydrogenase assembly factor 2, mitochondrial-like [Sus scrofa]	---	---	---	---
gi|335289736|ref|XP_003127196.2|	PREDICTED: inositol-trisphosphate 3-kinase C [Sus scrofa]	---	---	---	---
gi|335280193|ref|XP_003121819.2|	PREDICTED: cutaneous T-cell lymphoma-associated antigen 5-like isoform 1 [Sus scrofa] >gi|335280187|ref|XP_003353518.1| PREDICTED: cutaneous T-cell lymphoma-associated antigen 5 isoform 2 [Sus scrofa]	0.991	0.752	0.959	0.889
gi|335280195|ref|XP_003353	PREDICTED: cutaneous T-cell				

520.1|	lymphoma-associated antigen 5-like isoform 2 [Sus scrofa] >gi|335280191|ref|XP_003353519.1| PREDICTED: cutaneous T-cell lymphoma-associated antigen 5 isoform 3 [Sus scrofa]				
gi|211578376|ref|NP_001129984.1|	beta,beta-carotene 15,15~-monooxygenase [Sus scrofa] >gi|209571749|gb|ACI62530.1| beta-carotene 15,15~-monooxygenase 1 [Sus scrofa]	1.281	1.155	1.164	1.667
gi|311247444|ref|XP_003122650.1|	PREDICTED: reticulon-3 isoform 1 [Sus scrofa]	---	---	---	---
gi|47523756|ref|NP_999514.1|	pyruvate carboxylase, mitochondrial [Sus scrofa] >gi|300632138|emb|CBU89073.1| unnamed protein product [Sus scrofa] >gi|300628626|emb|CBU85988.1| unnamed protein product [Sus scrofa] >gi|300641281|emb|CBU93285.1| unnamed protein product [Sus scrofa]	---	---	---	---
gi|335292522|ref|XP_001924480.2|	PREDICTED: proteasome subunit beta type-5 [Sus scrofa]	---	---	---	---
gi|335310829|ref|XP_003362211.1|	PREDICTED: ubiquitin carboxyl-terminal hydrolase 14-like [Sus scrofa]	1.053	0.697	1.262	0.778
gi|311244988|ref|XP_003121641.1|	PREDICTED: regulator of microtubule dynamics protein 3-like [Sus scrofa]	---	---	---	---
gi|311260254|ref|XP_003128393.1|	PREDICTED: inositol 1,4,5-trisphosphate receptor type 3 [Sus scrofa]	---	---	---	---
gi|163915153|ref|NP_001106524.1|	NF-kappa-B essential modulator [Sus scrofa] >gi|161137769|gb|ABX57883.1| inhibitor of kappa light polypeptide gene enhancer in B-cells kinase gamma [Sus scrofa]	---	---	---	---
gi|166079195|gb|ABY81299.1|	inhibitor of kappa light polypeptide gene enhancer in B-cells kinase gamma transcript variant 1 [Sus scrofa]				

gi|34810486|pdb|1NX2|A	Chain A, Calpain Domain Vi >gi|34810482|pdb|1NX1|A Chain A, Calpain Domain Vi Complexed With Calpastatin Inhibitory Domain C (Dic) >gi|34810478|pdb|1NX0|B Chain B, Structure Of Calpain Domain 6 In Complex With Calpastatin Dic >gi|3319069|pdb|1ALV|B	0.843	0.93	1.21	1.038
gi|47523694|ref|NP_999483.1|	calpain small subunit 1 [Sus scrofa] >gi|115613|sp|P04574.1|CPNS1_PIG RecName: Full=Calpain small subunit 1; Short=CSS1; AltName: Full=Calcium-activated neutral proteinase small subunit; Short=CANP small subunit; AltName: Full=Calcium-dependent prot				
gi|219521950|ref|NP_001137167.1|	acetyl-coenzyme A synthetase, cytoplasmic [Sus scrofa] >gi|217039101|gb|ACJ76839.1| acyl-CoA synthetase short-chain family member 2 [Sus scrofa]	---	---	---	---
gi|350583332|ref|XP_001927031.4|	PREDICTED: pre-B-cell leukemia transcription factor-interacting protein 1-like [Sus scrofa]	---	---	---	---
gi|6174950|sp|P79324.1|RL15_PIG	RecName: Full=60S ribosomal protein L15	---	---	---	---
gi|346227216|ref|NP_001230994.1|	ribosomal protein L15 [Sus scrofa] >gi|335298742|ref|XP_003358383.1| PREDICTED: 60S ribosomal protein L15-like isoform 1 [Sus scrofa] >gi|335298744|ref|XP_003358384.1| PREDICTED: 60S ribosomal protein L15-like isoform 2 [Sus scrofa]				
gi|311252561|ref|XP_003125153.1|	PREDICTED: prefoldin subunit 1-like [Sus scrofa]	---	---	---	---
gi|350581165|ref|XP_003480975.1|	PREDICTED: prefoldin subunit 1-like [Sus scrofa]				
gi|350584178|ref|XP_003481687.1|	PREDICTED: prolow-density lipoprotein receptor-related protein 1-like [Sus scrofa]	---	---	---	---

gi|7716549|gb|AAF68430.1|AF239165_1	serine hydroxymethyltransferase, partial [Sus scrofa]	---	---	---	---
gi|47523070|ref|NP_999298.1|	pantetheinase precursor [Sus scrofa] >gi|46577140|sp|Q9BDJ5.1|VNN1_PIG RecName: Full=Pantetheinase; AltName: Full=Pantetheine hydrolase; AltName: Full=Vascular non-inflammatory molecule 1; Short=Vanin-1; Flags: Precursor >gi|13655618|gb|AAK29437.2|A	---	---	---	---
gi|194035771|ref|XP_001927964.1|	PREDICTED: guanylate-binding protein 4 [Sus scrofa]	1.152	1.559	1.072	0.948
gi|311256822|ref|XP_003126824.1|	PREDICTED: histone deacetylase 10-like [Sus scrofa]	---	---	---	---
gi|335310624|ref|XP_003362118.1|	PREDICTED: histone deacetylase 10-like [Sus scrofa]				
gi|164414802|pdb|2RME|A	Chain A, Stressin	---	---	---	---
gi|311248590|ref|XP_003123248.1|	PREDICTED: mitochondrial import inner membrane translocase subunit TIM44 [Sus scrofa]	---	---	---	---
gi|335298785|ref|XP_003132130.2|	PREDICTED: COX assembly mitochondrial protein homolog [Sus scrofa]	---	---	---	---
gi|335308716|ref|XP_003361344.1|	PREDICTED: selenide, water dikinase 1, partial [Sus scrofa]	---	---	---	---
gi|335281875|ref|XP_003353915.1|	PREDICTED: proteoglycan 3-like [Sus scrofa]	---	---	---	---
gi|311272131|ref|XP_003133314.1|	PREDICTED: aspartyl-tRNA synthetase, cytoplasmic-like isoform 1 [Sus scrofa]	0.967	0.599	0.349	0.865
gi|335302484|ref|XP_003359474.1|	PREDICTED: aspartyl-tRNA synthetase, cytoplasmic-like isoform 2 [Sus scrofa]				
gi|350587769|ref|XP_003129218.3|	PREDICTED: ATP-binding cassette sub-family E member 1 [Sus scrofa]	---	---	---	---
gi|311275848|ref|XP_003134937.1|	PREDICTED: biliverdin reductase A-like [Sus scrofa]	---	---	---	---
gi|311271149|ref|XP_003133065.1|	PREDICTED: uncharacterized protein C1orf31 homolog [Sus scrofa]	---	---	---	---
gi|335301594|ref|XP_003359	PREDICTED: uncharacterized				

243.1|	protein C1orf31 homolog [Sus scrofa] >gi|335301592|ref|XP_003359242.1| PREDICTED: uncharacterized protein C1orf31 homolog [Sus scrofa]				
gi|346716287|ref|NP_001231279.1|	carboxymethylenebutenolidase homolog [Sus scrofa]	---	---	---	---
gi|47522956|ref|NP_999236.1|	N-acetylneuraminate lyase [Sus scrofa] >gi|75049298|sp|Q9BEC7.1|NPL_PIG RecName: Full=N-acetylneuraminate lyase; Short=NALase; AltName: Full=N-acetylneuraminate pyruvate-lyase; AltName: Full=N-acetylneuraminic acid aldolase; AltName: Full=Sialate ly	---	---	---	---
gi|335292737|ref|XP_001927469.2|	PREDICTED: serine/arginine-rich splicing factor 5 isoform 2 [Sus scrofa] >gi|335292735|ref|XP_001927454.2| PREDICTED: serine/arginine-rich splicing factor 5 isoform 1 [Sus scrofa]	---	---	---	---
gi|335307313|ref|XP_003360793.1|	PREDICTED: serine/arginine-rich splicing factor 5-like [Sus scrofa]				
gi|346421328|ref|NP_001231038.1|	synapse-associated protein 1 isoform 2 [Sus scrofa]	---	---	---	---
gi|346421345|ref|NP_001231036.1|	synapse-associated protein 1 isoform 1 [Sus scrofa]				
gi|350580558|ref|XP_003354114.2|	PREDICTED: dynamin-2-like [Sus scrofa]	---	---	---	---
gi|178057329|ref|NP_001116638.1|	protein phosphatase 1 regulatory subunit 11 [Sus scrofa] >gi|194039988|ref|XP_001929404.1| PREDICTED: protein phosphatase 1 regulatory subunit 11-like [Sus scrofa] >gi|211926939|dbj|BAG82680.1| protein phosphatase 1, regulatory subunit 11 [Sus scrof	---	---	---	---
gi|350584858|ref|XP_003126900.3|	PREDICTED: craniofacial development protein 1, partial [Sus scrofa]	---	---	---	---
gi|347800629|ref|NP_001231	synaptosomal-associated protein,	---	---	---	---

648.1|	23kDa [Sus scrofa]				
gi|298104112|ref|NP_001177119.1|	charged multivesicular body protein 4c [Sus scrofa]	---	---	---	---
gi|311264732|ref|XP_003130306.1|	PREDICTED: synaptophysin-like protein 1-like [Sus scrofa]	---	---	---	---
gi|148230691|ref|NP_001090958.1|	Krueppel-like factor 5 [Sus scrofa] >gi|110007347|gb|ABG49105.1| kruppel-like factor 5 [Sus scrofa]	---	---	---	---
gi|335282891|ref|XP_003123558.2|	PREDICTED: myosin-IXb-like [Sus scrofa]	---	---	---	---
gi|194044882|ref|XP_001926942.1|	PREDICTED: 26S proteasome non-ATPase regulatory subunit 10 isoform 1 [Sus scrofa] >gi|335309762|ref|XP_003361757.1| PREDICTED: 26S proteasome non-ATPase regulatory subunit 10-like isoform 1 [Sus scrofa]	---	---	---	---
gi|343887438|ref|NP_001230620.1|	plasma glutamate carboxypeptidase precursor [Sus scrofa]	0.432	0.694	0.732	0.475
gi|350579791|ref|XP_003480684.1|	PREDICTED: endothelial differentiation-related factor 1-like isoform 2 [Sus scrofa] >gi|350579789|ref|XP_003480683.1| PREDICTED: endothelial differentiation-related factor 1-like isoform 1 [Sus scrofa] >gi|149132072|gb|ABR20898.1| multiprotein bridg	---	---	---	---
gi|350586171|ref|XP_003356500.2|	PREDICTED: ubiquitin carboxyl-terminal hydrolase 24 [Sus scrofa]	---	---	---	---
gi|232142|sp|P29797.1|GNAS_PIG	RecName: Full=Guanine nucleotide-binding protein G(s) subunit alpha; AltName: Full=Adenylate cyclase-stimulating G alpha protein >gi|1958|emb|CAA45355.1| alpha-stimulatory subunit of GTP-binding protein [Sus scrofa]	---	---	---	---
gi|147223308|emb|CAN13201.1|	GNAS complex locus [Sus scrofa] >gi|147223420|emb|CAN13152.1| GNAS complex locus [Sus scrofa]				

gi|147223417|emb|CAN13149.1|	GNAS complex locus [Sus scrofa] >gi|147223306|emb|CAN13199.1| GNAS complex locus [Sus scrofa]				
gi|285002235|ref|NP_999477.2|	guanine nucleotide-binding protein G(s) subunit alpha GNASL [Sus scrofa] >gi|147223423|emb|CAN13155.1| GNAS complex locus [Sus scrofa] >gi|147223313|emb|CAN13206.1| GNAS complex locus [Sus scrofa]				
gi|285002237|ref|NP_001165454.1|	guanine nucleotide-binding protein G(s) subunit alpha GNASS [Sus scrofa]				
gi|285002241|ref|NP_001165456.1|	guanine nucleotide-binding protein G(s) subunit alpha isoform g [Sus scrofa] >gi|147223316|emb|CAN13209.1| GNAS complex locus [Sus scrofa] >gi|147223424|emb|CAN13156.1| GNAS complex locus [Sus scrofa]				
gi|138752630|emb|CAM32247.1|	guanine nucleotide-binding protein, alpha-stimulating activity polypeptide 1 [Sus scrofa]				
gi|147223418|emb|CAN13150.1|	GNAS complex locus [Sus scrofa] >gi|147223307|emb|CAN13200.1| GNAS complex locus [Sus scrofa]				
gi|285002239|ref|NP_001165455.1|	guanine nucleotide-binding protein G(s) subunit alpha isoform f [Sus scrofa] >gi|147223425|emb|CAN13157.1| GNAS complex locus [Sus scrofa] >gi|147223315|emb|CAN13208.1| GNAS complex locus [Sus scrofa]				
gi|350585712|ref|XP_003482033.1|	PREDICTED: eukaryotic translation initiation factor 4 gamma 3-like [Sus scrofa]	---	---	---	---
gi|350579849|ref|XP_003480696.1|	PREDICTED: src substrate cortactin-like [Sus scrofa]	---	---	---	---
gi|343790902|ref|NP_001230508.1|	thymidylate synthetase [Sus scrofa]	---	---	---	---
gi|350596976|ref|XP_003361	PREDICTED: methionyl-tRNA	---	---	---	---

857.2|	synthetase, cytoplasmic-like [Sus scrofa]				
gi|343488509|ref|NP_001230414.1|	valacyclovir hydrolase precursor [Sus scrofa]	---	---	---	---
gi|227430403|ref|NP_001153085.1|	NAD(P)H dehydrogenase [quinone] 1 [Sus scrofa]	---	---	---	---
gi|350586151|ref|XP_003128006.3|	PREDICTED: LOW QUALITY PROTEIN: inaD-like protein [Sus scrofa]	---	---	---	---
gi|335284763|ref|XP_003124756.2|	PREDICTED: E3 ubiquitin-protein ligase CHIP-like [Sus scrofa]	---	---	---	---
gi|335297742|ref|XP_003131565.2|	PREDICTED: protein C17orf37 homolog [Sus scrofa]	---	---	---	---
gi|335300657|ref|XP_003358980.1|	PREDICTED: protein SON isoform 1 [Sus scrofa]	---	---	---	---
gi|335300659|ref|XP_003358981.1|	PREDICTED: protein SON isoform 2 [Sus scrofa]				
gi|311264544|ref|XP_003130216.1|	PREDICTED: replication protein A 14 kDa subunit-like [Sus scrofa]	---	---	---	---
gi|335307995|ref|XP_003361061.1|	PREDICTED: replication protein A 14 kDa subunit-like [Sus scrofa]				
gi|55926205|ref|NP_001007520.1|	interferon-related developmental regulator 1 [Sus scrofa] >gi|68565361|sp|Q5S1U6.1|IFRD1_PIG RecName: Full=Interferon-related developmental regulator 1 >gi|55709896|gb|AAV58829.1| interferon-related developmental regulator 1 [Sus scrofa]	---	---	---	---
gi|350590514|ref|XP_003131675.3|	PREDICTED: retinoid-inducible serine carboxypeptidase [Sus scrofa]	---	---	---	---
gi|335290950|ref|XP_003356343.1|	PREDICTED: tyrosyl-tRNA synthetase, cytoplasmic [Sus scrofa]	---	---	---	---
gi|350592546|ref|XP_001925361.4|	PREDICTED: TP53-regulated inhibitor of apoptosis 1-like, partial [Sus scrofa]	---	---	---	---
gi|350584124|ref|XP_003481674.1|	PREDICTED: melanocyte protein PMEL, partial [Sus scrofa]	---	---	---	---
gi|350584130|ref|XP_003126312.3|	PREDICTED: proliferation-associated protein 2G4 [Sus scrofa]				

gi|311276400|ref|XP_003135190.1|	PREDICTED: LOW QUALITY PROTEIN: hephaestin-like [Sus scrofa]	---	---	---	---
gi|350588022|ref|XP_003482536.1|	PREDICTED: protein transport protein Sec31A-like [Sus scrofa]	---	---	---	---
gi|347300255|ref|NP_001231435.1|	DAZ-associated protein 1 [Sus scrofa]	---	---	---	---
gi|311270533|ref|XP_003132904.1|	PREDICTED: splicing factor, suppressor of white-apricot homolog [Sus scrofa]	---	---	---	---
gi|335297337|ref|XP_003131233.2|	PREDICTED: signal recognition particle 68 kDa protein-like [Sus scrofa]	---	---	---	---
gi|350584672|ref|XP_003126766.2|	PREDICTED: transmembrane and coiled-coil domains protein 3 [Sus scrofa]	---	---	---	---
gi|350585412|ref|XP_003356115.2|	PREDICTED: receptor-type tyrosine-protein phosphatase H, partial [Sus scrofa]	---	---	---	---
gi|311248328|ref|XP_003123084.1|	PREDICTED: nicalin [Sus scrofa]	---	---	---	---
gi|194038528|ref|XP_001929053.1|	PREDICTED: acyl-coenzyme A thioesterase 6-like [Sus scrofa]	---	---	---	---
gi|194042067|ref|XP_001929334.1|	PREDICTED: actin-binding LIM protein 1 isoform 1 [Sus scrofa]	---	---	---	---
gi|311271869|ref|XP_003133239.1|	PREDICTED: actin-binding LIM protein 1 isoform 3 [Sus scrofa]				
gi|311271871|ref|XP_003133238.1|	PREDICTED: actin-binding LIM protein 1 isoform 2 [Sus scrofa]				
gi|311249377|ref|XP_003123620.1|	PREDICTED: transcriptional repressor p66-alpha [Sus scrofa]	---	---	---	---
gi|194043684|ref|XP_001926541.1|	PREDICTED: inward rectifier potassium channel 13 isoform 1 [Sus scrofa]	---	---	---	---
gi|16033755|gb|AAL13316.1|AF424780_1	cytochrome P450 3A [Sus scrofa]	---	---	---	---
gi|19071856|dbj|BAB85672.1|	cytochrome P450 3A46v1 [Sus scrofa]				
gi|19071846|dbj|BAB85667.1|	cytochrome P450 3A29v3 [Sus scrofa]				
gi|196475656|gb|ACG76385.1|	cytochrome P450 CYP3A [Sus scrofa]				

gi|47523900|ref|NP_999588.1|	cytochrome P450 3A29 [Sus scrofa] >gi|5921921|sp|P79401.1|CP3AT_PIG RecName: Full=Cytochrome P450 3A29; AltName: Full=CYPIIIA29 >gi|1903317|emb|CAB07513.1| cytochrome P450 [Sus scrofa]				
gi|19071852|dbj|BAB85670.1|	cytochrome P450 3A39v3 [Sus scrofa]				
gi|19071844|dbj|BAB85666.1|	cytochrome P450 3A29v2 [Sus scrofa]				
gi|19071854|dbj|BAB85671.1|	cytochrome P450 3A39v4 [Sus scrofa]				
gi|198282077|ref|NP_001128296.1|	cytochrome P450 3A46 [Sus scrofa] >gi|197791176|gb|ABS58488.2| cytochrome P450 [Sus scrofa]				
gi|47523898|ref|NP_999587.1|	cytochrome P450 3A39 [Sus scrofa] >gi|4151927|gb|AAD04628.1| cytochrome P450 [Sus scrofa]				
gi|19071848|dbj|BAB85668.1|	cytochrome P450 3A29v4 [Sus scrofa]				
gi|154101336|gb|ABS58494.1|	cytochrome P450 [Sus scrofa]				
gi|19071858|dbj|BAB85673.1|	cytochrome P450 3A46v2 [Sus scrofa]				
gi|343488540|ref|NP_001230425.1|	GSK3-beta interaction protein [Sus scrofa]	1.315	1.569	1.714	0.976
gi|335308872|ref|XP_003361404.1|	PREDICTED: serine hydroxymethyltransferase, cytosolic [Sus scrofa]	---	---	---	---
gi|335309568|ref|XP_003130716.2|	PREDICTED: ubiquitin-associated protein 2-like, partial [Sus scrofa]	---	---	---	---
gi|350589476|ref|XP_003482855.1|	PREDICTED: ubiquitin-associated protein 2-like [Sus scrofa]				
gi|346986410|ref|NP_001231355.1|	glycine cleavage system T-protein [Sus scrofa]	---	---	---	---
gi|350587512|ref|XP_003129026.3|	PREDICTED: pre-mRNA 3~-end-processing factor FIP1-like [Sus scrofa]	---	---	---	---
gi|313570009|ref|NP_001186292.1|	calcium-binding mitochondrial carrier protein SCaMC-1-like [Sus scrofa] >gi|186886352|gb|ACC93575.	---	---	---	---

	1| small calcium-binding mitochondrial carrier 1 [Sus scrofa]				
gi|350591284|ref|XP_003132316.2|	PREDICTED: sarcolemmal membrane-associated protein [Sus scrofa]	---	---	---	---
gi|350580065|ref|XP_003122766.3|	PREDICTED: syntaxin-3-like, partial [Sus scrofa]	0.474	0.757	0.71	0.552
gi|335296874|ref|XP_003131019.2|	PREDICTED: calcium-binding protein 39-like [Sus scrofa]	---	---	---	---
gi|335310350|ref|XP_003361992.1|	PREDICTED: carbonyl reductase [NADPH] 1-like, partial [Sus scrofa]	---	---	---	---
gi|350580387|ref|XP_003480810.1|	PREDICTED: calcium homeostasis endoplasmic reticulum protein-like [Sus scrofa]	---	---	---	---
gi|335296376|ref|XP_003357763.1|	PREDICTED: probable gluconokinase-like [Sus scrofa]	---	---	---	---
gi|335297595|ref|XP_003358072.1|	PREDICTED: interferon-induced 35 kDa protein-like [Sus scrofa]	---	---	---	---
gi|350595403|ref|XP_003134876.3|	PREDICTED: hypothetical protein LOC100516841 [Sus scrofa]	---	---	---	---
gi|350594194|ref|XP_003359791.2|	PREDICTED: hypothetical protein LOC100620294 [Sus scrofa]	---	---	---	---
gi|350582696|ref|XP_003354965.2|	PREDICTED: ATP-dependent RNA helicase DDX1 [Sus scrofa]	---	---	---	---
gi|297747306|ref|NP_001177083.1|	zinc finger, CCHC domain containing 8 [Sus scrofa]	---	---	---	---
gi|350596491|ref|XP_003484281.1|	PREDICTED: zinc finger CCHC domain-containing protein 8-like, partial [Sus scrofa]				
gi|298104144|ref|NP_001177137.1|	S100 calcium binding protein A16 [Sus scrofa] >gi|298104142|ref|NP_001177136.1| S100 calcium binding protein A16 [Sus scrofa]	---	---	---	---
gi|350583616|ref|XP_003481553.1|	PREDICTED: lipoamide acyltransferase component of branched-chain alpha-keto acid dehydrogenase complex, mitochondrial isoform 2 [Sus scrofa]	---	---	---	---
gi|350583618|ref|XP_001924875.4|	PREDICTED: lipoamide acyltransferase component of branched-chain alpha-keto acid				

	dehydrogenase complex, mitochondrial isoform 1 [Sus scrofa]				
gi|343488551|ref|NP_001230428.1|	mitochondrial intermembrane space import and assembly protein 40 [Sus scrofa]	---	---	---	---
gi|311272864|ref|XP_003133619.1|	PREDICTED: uncharacterized protein C2orf47 homolog, mitochondrial-like [Sus scrofa]	---	---	---	---
gi|350596869|ref|XP_003361751.2|	PREDICTED: uncharacterized protein C2orf47 homolog, mitochondrial-like [Sus scrofa]				
gi|194043356|ref|XP_001929473.1|	PREDICTED: SWI/SNF-related matrix-associated actin-dependent regulator of chromatin subfamily B member 1 isoform 2 [Sus scrofa]	---	---	---	---
gi|194043360|ref|XP_001929472.1|	PREDICTED: SWI/SNF-related matrix-associated actin-dependent regulator of chromatin subfamily B member 1 isoform 1 [Sus scrofa]				
gi|350583576|ref|XP_003125920.3|	PREDICTED: sortilin [Sus scrofa]	---	---	---	---
gi|373248672|emb|CCF70742.1|	sortilin 1, partial [Sus scrofa] >gi|373248668|emb|CCF70740.1| sortilin 1, partial [Sus scrofa] >gi|373248674|emb|CCF70743.1| sortilin 1, partial [Sus scrofa] >gi|373248670|emb|CCF70741.1| sortilin 1, partial [Sus scrofa]				
gi|350588230|ref|XP_003129648.3|	PREDICTED: nuclear pore complex protein Nup98-Nup96-like [Sus scrofa]	---	---	---	---
gi|121195|sp|P01281.1|GIP_PIG	RecName: Full=Gastric inhibitory polypeptide; Short=GIP; AltName: Full=Glucose-dependent insulinotropic polypeptide	---	---	---	---
gi|388255283|gb|AFK25156.1|	gastric inhibitory polypeptide precursor [Sus scrofa]				
gi|415514|gb|AAB28179.1|	diazepam-binding inhibitor (32-86), DBI(32-86)=antibacterial peptide [swine, small intestine, Peptide Partial, 36 aa]				
gi|335297830|ref|XP_003358130.1|	PREDICTED: gastric inhibitory polypeptide-like [Sus scrofa]				

gi|350592229|ref|XP_003359063.2|	PREDICTED: lanosterol synthase-like [Sus scrofa]	---	---	---	---
gi|311246600|ref|XP_001926112.2|	PREDICTED: serine/threonine-protein phosphatase 2A activator-like isoform 1 [Sus scrofa]	---	---	---	---
gi|335281070|ref|XP_003353729.1|	PREDICTED: serine/threonine-protein phosphatase 2A activator-like isoform 2 [Sus scrofa]				
gi|147900197|ref|NP_001090953.1|	cytochrome c oxidase subunit 7A2, mitochondrial [Sus scrofa] >gi|117661207|gb|ABK55662.1| COX7A2 [Sus scrofa]	---	---	---	---
gi|311270525|ref|XP_003132902.1|	PREDICTED: tRNA pseudouridine synthase A, mitochondrial [Sus scrofa] >gi|194043580|ref|XP_001928625.1| PREDICTED: tRNA pseudouridine synthase A, mitochondrial isoform 2 [Sus scrofa]	---	---	---	---
gi|311245687|ref|XP_003121922.1|	PREDICTED: uncharacterized protein C9orf82 homolog [Sus scrofa]	---	---	---	---
gi|350587407|ref|XP_003482407.1|	PREDICTED: hypothetical protein LOC100737378 [Sus scrofa]	---	---	---	---
gi|335307979|ref|XP_003361054.1|	PREDICTED: abhydrolase domain-containing protein 10, mitochondrial-like [Sus scrofa]	---	---	---	---
gi|189232885|emb|CAQ34905.1|	signal recognition particle receptor B subunit [Sus scrofa]	---	---	---	---
gi|311269414|ref|XP_003132477.1|	PREDICTED: signal recognition particle receptor subunit beta-like [Sus scrofa]				
gi|350594246|ref|XP_003133954.3|	PREDICTED: disabled homolog 2 [Sus scrofa]	---	---	---	---
gi|147903209|ref|NP_001090934.1|	small nuclear ribonucleoprotein E [Sus scrofa] >gi|166233313|sp|A1XQR9.1|RUXE_PIG RecName: Full=Small nuclear ribonucleoprotein E; Short=snRNP-E; AltName: Full=Sm protein E; Short=Sm-E; Short=SmE >gi|117660560|gb|ABK55626.1| NDUFB2 [Sus scrofa] >gi|	---	---	---	---

gi|350594505|ref|XP_003359894.2|	PREDICTED: annexin A6-like [Sus scrofa]	---	---	---	---
gi|348041218|ref|NP_001231706.1|	Ena-vasodilator stimulated phosphoprotein [Sus scrofa]	---	---	---	---
gi|350587198|ref|XP_003482364.1|	PREDICTED: ena/VASP-like protein-like [Sus scrofa]				
gi|156779005|gb|ABU95648.1|	sirtuin 2 [Sus scrofa]	---	---	---	---
gi|166796035|ref|NP_001107743.1|	NAD-dependent deacetylase sirtuin-2 [Sus scrofa] >gi|164653929|gb|ABY65333.1| sirtuin 2 [Sus scrofa]				
gi|227057425|gb|ACP18879.1|	truncated sirtuin 2 [Sus scrofa]				
gi|227057441|gb|ACP18880.1|	sirtuin 2 [Sus scrofa]				
gi|311258366|ref|XP_003127578.1|	PREDICTED: espin [Sus scrofa]	---	---	---	---
gi|350585555|ref|XP_003481987.1|	PREDICTED: espin-like [Sus scrofa]				
gi|350578468|ref|XP_003121482.3|	PREDICTED: myosin-Vb [Sus scrofa]	---	---	---	---
gi|350581059|ref|XP_003123938.3|	PREDICTED: phosphorylated adapter RNA export protein-like [Sus scrofa]	---	---	---	---
gi|350584132|ref|XP_003481675.1|	PREDICTED: proliferation-associated protein 2G4-like [Sus scrofa]	---	---	---	---
gi|347800675|ref|NP_001231654.1|	molybdenum cofactor synthesis 2 [Sus scrofa]	---	---	---	---
gi|63139103|gb|AAY33861.1|	centrin 2 [Sus scrofa]	---	---	---	---
gi|194044860|ref|XP_001927244.1|	PREDICTED: centrin-2 [Sus scrofa]				
gi|33320702|gb|AAQ05997.1|	ATPase subunit 8 [Sus scrofa]	---	---	---	---
gi|90969048|gb|ABE02601.1|	ATP synthase F0 subunit 8 [Sus scrofa]				
gi|94981325|gb|ABF49553.1|	ATPase subunit 8 [Sus scrofa] >gi|145315685|gb|ABP63227.1| ATP synthase F0 subunit 8 [Sus scrofa] >gi|145315447|gb|ABP63006.1| ATP synthase F0 subunit 8 [Sus scrofa] >gi|124249812|gb|ABM92885				

	.1| ATPase subunit 8 [Sus scrofa]				
gi|124358981|ref|YP_001023767.1|	ATP synthase F0 subunit 8 [Phacochoerus africanus] >gi|88766365|gb|ABD49697.1| ATP synthase F0 subunit 8 [Phacochoerus africanus]				
gi|33320870|gb|AAQ06153.1|	ATPase subunit 8 [Sus scrofa]				
gi|312233368|ref|YP_004021579.1|	ATP synthase F0 subunit 8 [Sus scrofa taiwanensis] >gi|347448742|gb|AEO93054.1| ATP synthase F0 subunit 8 (mitochondrion) [Sus scrofa] >gi|347448686|gb|AEO93002.1| ATP synthase F0 subunit 8 (mitochondrion) [Sus scrofa] >gi|347448728|gb|AEO93041.1| A				
gi|347802434|gb|AEP22018.1|	ATP synthase F0 subunit 8 (mitochondrion) [Potamochoerus porcus]				
gi|45826174|gb|AAS77695.1|	ATP synthase F0 subunit 8 [Sus scrofa]				
gi|335282277|ref|XP_003354019.1|	PREDICTED: NADH dehydrogenase [ubiquinone] iron-sulfur protein 7, mitochondrial-like isoform 1 [Sus scrofa]	---	---	---	---
gi|335282279|ref|XP_003354020.1|	PREDICTED: NADH dehydrogenase [ubiquinone] iron-sulfur protein 7, mitochondrial-like isoform 2 [Sus scrofa]				
gi|157427724|ref|NP_001098770.1|	COP9 signalosome complex subunit 5 [Sus scrofa] >gi|148800597|gb|ABR13016.1| COP9 constitutive photomorphogenic-like subunit 5 [Sus scrofa]	---	---	---	---
gi|226709210|sp|A1XQT2.1|COX6C_PIG	RecName: Full=Cytochrome c oxidase subunit 6C; AltName: Full=Cytochrome c oxidase polypeptide VIc >gi|148225574|ref|NP_001090942.1| cytochrome c oxidase subunit 6C [Sus	0.532	0.35	0.448	0.376

	scrofa] >gi|117660798|gb|ABK55638.1| COX6C [Sus scrofa]				
gi|335297915|ref|XP_003358154.1|	PREDICTED: ankyrin repeat domain-containing protein 40-like [Sus scrofa]	---	---	---	---
gi|194043190|ref|XP_001928943.1|	PREDICTED: protein NipSnap homolog 1 isoform 1 [Sus scrofa]	---	---	---	---
gi|335301406|ref|XP_003359199.1|	PREDICTED: protein NipSnap homolog 1 isoform 2 [Sus scrofa]				
gi|56404321|sp|Q71LE2.3|H33_PIG	RecName: Full=Histone H3.3 >gi|47522626|ref|NP_999095.1| histone H3.3 [Sus scrofa] >gi|311266778|ref|XP_003131242.1| PREDICTED: histone H3.3-like [Sus scrofa] >gi|18643343|gb|AAL76273.1|AF469469_1 histone H3.3A [Sus scrofa]	---	---	---	---
gi|335291702|ref|XP_003356567.1|	PREDICTED: hypothetical protein LOC100627410 [Sus scrofa]				
gi|311259879|ref|XP_003128260.1|	PREDICTED: histone H3.1-like [Sus scrofa] >gi|194039818|ref|XP_001927801.1| PREDICTED: histone H3.1-like [Sus scrofa] >gi|336092201|gb|AEI00724.1| histone cluster 1 [Sus scrofa]				
gi|194039886|ref|XP_001928622.1|	PREDICTED: histone H3.1-like [Sus scrofa]				
gi|350583434|ref|XP_003481517.1|	PREDICTED: histone H3.2 [Sus scrofa] >gi|350583419|ref|XP_003481513.1| PREDICTED: histone H3.2-like [Sus scrofa] >gi|335287027|ref|XP_003355255.1| PREDICTED: histone H3.2-like [Sus scrofa] >gi|347582608|ref|NP_001231571.1| histone H3.2 [Sus scrofa]				
gi|19919838|gb|AAM08402.1|AF490841_1	glutaminase [Sus scrofa]	---	---	---	---
gi|350593725|ref|XP_003133597.3|	PREDICTED: glutaminase kidney isoform, mitochondrial [Sus scrofa]				
gi|335289253|ref|XP_003126970.2|	PREDICTED: nuclear transport factor 2-like [Sus scrofa]	---	---	---	---

gi|335310392|ref|XP_003362011.1|	PREDICTED: nuclear transport factor 2-like [Sus scrofa]				
gi|311276597|ref|XP_003135272.1|	PREDICTED: hypothetical protein LOC100511612 [Sus scrofa]	---	---	---	---
gi|335301488|ref|XP_003359221.1|	PREDICTED: phosphatidylinositol 4-kinase alpha-like [Sus scrofa]	---	---	---	---
gi|335292819|ref|XP_003356805.1|	PREDICTED: activator of 90 kDa heat shock protein ATPase homolog 1-like [Sus scrofa] >gi|194038625|ref|XP_001928925.1| PREDICTED: activator of 90 kDa heat shock protein ATPase homolog 1 [Sus scrofa]	---	---	---	---
gi|298104110|ref|NP_001177118.1|	tricarboxylate transport protein, mitochondrial [Sus scrofa]	---	---	---	---
gi|343790961|ref|NP_001230534.1|	calcium uptake protein 1, mitochondrial [Sus scrofa]	---	---	---	---
gi|350593895|ref|XP_003483787.1|	PREDICTED: E3 ubiquitin-protein ligase RNF25 [Sus scrofa]	---	---	---	---
gi|350596061|ref|XP_003484224.1|	PREDICTED: E3 ubiquitin-protein ligase RNF25-like [Sus scrofa]				
gi|106073338|gb|ABF81978.1|	muscle glycogen phosphorylase [Sus scrofa]	---	---	---	---
gi|335281566|ref|XP_003122636.2|	PREDICTED: glycogen phosphorylase, muscle form isoform 1 [Sus scrofa]				
gi|335281570|ref|XP_003353833.1|	PREDICTED: glycogen phosphorylase, muscle form isoform 3 [Sus scrofa]				
gi|335281568|ref|XP_003353832.1|	PREDICTED: glycogen phosphorylase, muscle form isoform 2 [Sus scrofa]				
gi|300119711|gb|ABF81977.2|	muscle glycogen phosphorylase [Sus scrofa]				
gi|311254844|ref|XP_001929459.2|	PREDICTED: serine/threonine-protein kinase N2 isoform 1 [Sus scrofa]	---	---	---	---
gi|335305150|ref|XP_003134640.2|	PREDICTED: zyxin [Sus scrofa]	---	---	---	---
gi|350591506|ref|XP_003132459.3|	PREDICTED: ruvB-like 1-like, partial [Sus scrofa]	---	---	---	---
gi|311274870|ref|XP_003134	PREDICTED:	---	---	---	---

490.1|	1-phosphatidylinositol-4,5-bisphosphate phosphodiesterase gamma-1-like [Sus scrofa]				
gi|311243834|ref|XP_003121200.1|	PREDICTED: splicing factor 3B subunit 5-like [Sus scrofa]	---	---	---	---
gi|350578376|ref|XP_001924454.2|	PREDICTED: phosphoacetylglucosamine mutase [Sus scrofa]	---	---	---	---
gi|298104097|ref|NP_001177113.1|	dnaJ homolog subfamily C member 3 precursor [Sus scrofa]	---	---	---	---
gi|308208140|gb|ADO20319.1|	P58IPK [Sus scrofa]				
gi|67468552|gb|AAY67906.1|	centrin 3 [Sus scrofa]	---	---	---	---
gi|311249828|ref|XP_003123825.1|	PREDICTED: centrin-3 [Sus scrofa]				
gi|349732208|ref|NP_001231856.1|	mitochondrial ribosomal protein S35 [Sus scrofa]	---	---	---	---
gi|350583519|ref|XP_003125889.2|	PREDICTED: round spermatid basic protein 1-like [Sus scrofa]	---	---	---	---
gi|12229602|sp|Q9XS72.1|CD1A_PIG	RecName: Full=T-cell surface glycoprotein CD1a; AltName: CD_antigen=CD1a; Flags: Precursor >gi|47523190|ref|NP_998996.1| T-cell surface glycoprotein CD1a precursor [Sus scrofa] >gi|6822270|gb|AAF28737.1| CD1 antigen [Sus scrofa] >gi|4678984|gb|AAD26983.1|AF059492_1 CD1 antigen [Sus scrofa]	---	---	---	---
gi|118764570|dbj|BAF38758.1|	CD1A antigen [Sus scrofa]				
gi|118764597|dbj|BAF38778.1|	CD1A1 antigen [Sus scrofa]				
gi|47523884|ref|NP_999578.1|	cytochrome P450 2B22 precursor [Sus scrofa] >gi|19071836|dbj|BAB85662.1| cytochrome P450 2B22 [Sus scrofa]	---	---	---	---
gi|335290417|ref|XP_003356174.1|	PREDICTED: protein RER1-like [Sus scrofa]	---	---	---	---
gi|350585528|ref|XP_003481	PREDICTED: protein RER1-like				

980.1|	[Sus scrofa]				
gi|311260790|ref|XP_003128538.1|	PREDICTED: serine/threonine-protein kinase ULK3 [Sus scrofa]	---	---	---	---
gi|68565351|sp|Q767L8.1|MDC1_PIG	RecName: Full=Mediator of DNA damage checkpoint protein 1 >gi|178056868|ref|NP_001116610.1| mediator of DNA damage checkpoint protein 1 [Sus scrofa] >gi|41529174|dbj|BAD08434.1| NFBD1 [Sus scrofa]	---	---	---	---
gi|311246626|ref|XP_003122268.1|	PREDICTED: uncharacterized protein C9orf78-like [Sus scrofa]	---	---	---	---
gi|350589666|ref|XP_003482893.1|	PREDICTED: malignant T cell-amplified sequence 1-like [Sus scrofa] >gi|350589658|ref|XP_003482889.1| PREDICTED: malignant T cell-amplified sequence 1-like [Sus scrofa]	---	---	---	---
gi|148747342|ref|NP_001092055.1|	AP-3 complex subunit beta-1 [Sus scrofa] >gi|146741314|dbj|BAF62312.1| adaptor-related protein complex 3, beta-1 subunit [Sus scrofa]	---	---	---	---
gi|335287423|ref|XP_001929441.3|	PREDICTED: transcription initiation factor IIB [Sus scrofa]	---	---	---	---
gi|346716308|ref|NP_001231150.1|	RNA-binding protein 14 [Sus scrofa]	---	---	---	---
gi|335308902|ref|XP_003361415.1|	PREDICTED: heterogeneous nuclear ribonucleoprotein L-like [Sus scrofa]	---	---	---	---
gi|350579679|ref|XP_003480661.1|	PREDICTED: niban-like protein 1-like [Sus scrofa]	---	---	---	---
gi|194034889|ref|XP_001929437.1|	PREDICTED: zinc finger FYVE domain-containing protein 19 [Sus scrofa]	---	---	---	---
gi|350589849|ref|XP_003357888.2|	PREDICTED: fibronectin type-III domain-containing protein 3A-like [Sus scrofa]	---	---	---	---
gi|350585300|ref|XP_003127257.3|	PREDICTED: protein ETHE1, mitochondrial-like isoform 1 [Sus scrofa]	---	---	---	---
gi|47522794|ref|NP_999149.1|	small ubiquitin-related modifier 2 precursor [Sus	---	---	---	---

	scrofa] >gi|48429127|sp|P61958.1|SUMO2_PIG RecName: Full=Small ubiquitin-related modifier 2; Short=SUMO-2; AltName: Full=MIF2 suppressor; AltName: Full=SMT3 homolog 2; AltName: Full=Sentrin-2; AltNam				
gi|335300844|ref|XP_003359058.1|	PREDICTED: small ubiquitin-related modifier 3-like [Sus scrofa]				
gi|194039401|ref|XP_001929628.1|	PREDICTED: alanyl-tRNA synthetase, mitochondrial [Sus scrofa]	---	---	---	---
gi|109675377|gb|ABG37112.1|	ataxia-telangiectasia mutated protein splice variant 7 [Sus scrofa]	---	---	---	---
gi|178056824|ref|NP_001116552.1|	serine-protein kinase ATM [Sus scrofa] >gi|152031564|sp|Q6PQD5.2|ATM_PIG RecName: Full=Serine-protein kinase ATM; AltName: Full=Ataxia telangiectasia mutated homolog; Short=A-T mutated homolog >gi|115433705|gb|AAT01608.2| ataxia-telangiectasia mutat				
gi|335302143|ref|XP_003359391.1|	PREDICTED: nucleoplasmin-3-like [Sus scrofa]	---	---	---	---
gi|118403762|ref|NP_001072156.1|	extracellular superoxide dismutase precursor [Sus scrofa] >gi|115522015|gb|ABJ09397.1| extracellular superoxide dismutase [Sus scrofa]	---	---	---	---
gi|58332866|ref|NP_001011509.1|	heme-binding protein 1 [Sus scrofa] >gi|75040507|sp|Q5ISC6.1|HEBP1_PIG RecName: Full=Heme-binding protein 1 >gi|51847749|gb|AAU10512.1| heme-binding protein [Sus scrofa]	---	---	---	---
gi|350594366|ref|XP_003134051.3|	PREDICTED: splicing regulatory glutamine/lysine-rich protein 1, partial [Sus scrofa]	---	---	---	---
gi|311247104|ref|XP_003122481.1|	PREDICTED: V-type proton ATPase 116 kDa subunit a isoform 3 [Sus scrofa]	---	---	---	---
gi|346986279|ref|NP_001231	serine/arginine-rich splicing factor 10	---	---	---	---

297.1|	isoform 2 [Sus scrofa]				
gi|346986281|ref|NP_001231298.1|	serine/arginine-rich splicing factor 10 isoform 1 [Sus scrofa] >gi|338784425|gb|AEI98875.1| serine/arginine-rich splicing factor 10 isoform 2 [Sus scrofa]				
gi|343183422|ref|NP_001230269.1|	proteasome inhibitor PI31 subunit [Sus scrofa]	---	---	---	---
gi|350594770|ref|XP_003483974.1|	PREDICTED: proteasome inhibitor PI31 subunit-like [Sus scrofa]				
gi|335300014|ref|XP_003358759.1|	PREDICTED: von Willebrand factor A domain-containing protein 5B2 [Sus scrofa]	---	---	---	---
gi|335304240|ref|XP_003359898.1|	PREDICTED: tRNA (cytosine(34)-C(5))-methyltransferase isoform 2 [Sus scrofa]	---	---	---	---
gi|335304242|ref|XP_003134206.2|	PREDICTED: tRNA (cytosine(34)-C(5))-methyltransferase isoform 1 [Sus scrofa]				
gi|350596463|ref|XP_003484278.1|	PREDICTED: high mobility group protein 20A-like [Sus scrofa] >gi|350586820|ref|XP_003482285.1| PREDICTED: high mobility group protein 20A [Sus scrofa]	---	---	---	---
gi|194038051|ref|XP_001926686.1|	PREDICTED: nuclear pore complex protein Nup153 [Sus scrofa]	---	---	---	---
gi|347300376|ref|NP_001231482.1|	28S ribosomal protein S9, mitochondrial [Sus scrofa]	---	---	---	---
gi|350583688|ref|XP_003355353.2|	PREDICTED: TBC1 domain family member 22A-like [Sus scrofa]	---	---	---	---
gi|335290628|ref|XP_003356226.1|	PREDICTED: LOW QUALITY PROTEIN: heterochromatin protein 1-binding protein 3 [Sus scrofa]	---	---	---	---
gi|350582787|ref|XP_003481357.1|	PREDICTED: low molecular weight phosphotyrosine protein phosphatase-like [Sus scrofa]	---	---	---	---
gi|350596121|ref|XP_003125455.3|	PREDICTED: hypothetical protein LOC100516434 [Sus scrofa]				
gi|297747311|ref|NP_001177084.1|	coiled-coil-helix-coiled-coil-helix domain containing 1 [Sus scrofa]	---	---	---	---
gi|311247991|ref|XP_003122917.1|	PREDICTED: pyruvate dehydrogenase protein X	---	---	---	---

	component-like isoform 1 [Sus scrofa]				
gi|335282040|ref|XP_003353955.1|	PREDICTED: pyruvate dehydrogenase protein X component-like isoform 2 [Sus scrofa]				
gi|350595129|ref|XP_003360127.2|	PREDICTED: ATP-binding cassette sub-family B member 8, mitochondrial-like [Sus scrofa]	---	---	---	---
gi|1827456|dbj|BAA19177.1|	metallothionein isoform [Sus scrofa]	---	---	---	---
gi|350596343|ref|XP_003361065.2|	PREDICTED: myomegalin-like, partial [Sus scrofa]	---	---	---	---
gi|55776192|gb|AAV65283.1|	cytochrome P450 2C34 [Sus scrofa]	---	---	---	---
gi|335291123|ref|XP_003356402.1|	PREDICTED: receptor-type tyrosine-protein phosphatase mu-like [Sus scrofa]	---	---	---	---
gi|350580860|ref|XP_003123760.3|	PREDICTED: IQ motif containing GTPase activating protein 2 [Sus scrofa]	---	---	---	---
gi|311246904|ref|XP_003122389.1|	PREDICTED: torsin family protein C9orf167-like [Sus scrofa]	---	---	---	---
gi|14269035|gb|AAK57999.1|	immunoglobulin lambda chain [Sus scrofa]	0.318	0.138	0.11	0.073
gi|335310412|ref|XP_003362020.1|	PREDICTED: centrin-2-like [Sus scrofa]	---	---	---	---
gi|194035504|ref|XP_001927493.1|	PREDICTED: vacuolar protein sorting-associated protein 28 homolog [Sus scrofa]	---	---	---	---
gi|39931643|sp|P83686.1|NB5R3_PIG	RecName: Full=NADH-cytochrome b5 reductase 3; Short=B5R; Short=Cytochrome b5 reductase; AltName: Full=Diaphorase-1 >gi|157832094|pdb|1NDH|A Chain A, Crystal Structure Of Nadh-Cytochrome B5 Reductase From Pig Liver At 2.4 Angstroms Resolution	---	---	---	---
gi|311254975|ref|XP_003126030.1|	PREDICTED: NADH-cytochrome b5 reductase 3-like [Sus scrofa]				
gi|1827460|dbj|BAA19179.1|	metallothionein isoform [Sus scrofa]	---	---	---	---
gi|18202364|sp|P79377.2|MT1D_PIG	RecName: Full=Metallothionein-1D; Short=MT-1D; AltName:				

	Full=Metallothionein-ID; Short=MT-ID				
gi|18202365|sp|P79378.1|MT1F_PIG	RecName: Full=Metallothionein-1F; Short=MT-1F; AltName: Full=Metallothionein-IF; Short=MT-IF >gi|1827466|dbj|BAA19182.1| metallothionein isoform [Sus scrofa]				

Data file 2. Differentially expressed proteins

Gene	W0d vs W0d	W1d vs W0d	W3d vs W0d	W5d vs W0d	W7d vs W0d
gi|14269035|gb|AAK57999.1| immunoglobulin lambda chain [Sus scrofa]	1.000	0.318	0.138	0.110	0.073
gi|47522616|ref|NP_999102.1| ubiquitin-like protein FUBI precursor [Sus scrofa] >gi|1628628|gb|AAB52915.1| ubiquitin-like/S30 ribosomal fusion protein [Sus scrofa]	1.000	0.111	0.123	0.101	0.111
gi|164505|gb|AAA51294.1| immunoglobulin gamma-chain, partial [Sus scrofa]	1.000	0.237	0.099	0.217	0.183
gi|311252239|ref|XP_003124992.1| PREDICTED: all-trans-retinol 13,14-reductase [Sus scrofa]	1.000	0.520	0.200	0.307	0.231
gi|50657386|ref|NP_001002801.1| apolipoprotein C-III precursor [Sus scrofa] >gi|416627|sp|P27917.2|APOC3_PIG RecName: Full=Apolipoprotein C-III; Short=Apo-CIII; Short=ApoC-III; AltName: Full=Apolipoprotein C3; Flags: Precursor >gi|164361|gb|AAA30993.1| apolipoprotein C-III [Sus sc	1.000	0.204	0.240	0.217	0.237
gi|47522712|ref|NP_999108.1| pyridoxal kinase [Sus scrofa] >gi|67470293|sp|O46560.1|PDXK_PIG RecName: Full=Pyridoxal kinase; AltName: Full=Pyridoxine kinase >gi|2773404|gb|AAB96794.1| pyridoxal kinase [Sus scrofa]	1.000	0.708	0.261	0.275	0.245
gi|194039884|ref|XP_001928592.1| PREDICTED: histone H2A type 1-like [Sus scrofa]	1.000	0.354	0.344	0.267	0.246
gi|350529356|ref|NP_001231917.1| NADH dehydrogenase (ubiquinone) Fe-S protein 5, 15kDa (NADH-coenzyme Q reductase) [Sus scrofa]	1.000	0.217	0.436	0.519	0.262
gi|311248247|ref|XP_003123039.1| PREDICTED: ATP synthase subunit delta, mitochondrial-like isoform 1 [Sus scrofa]	1.000	0.280	0.385	0.346	0.274

gi|311259100|ref|XP_003127931.1| PREDICTED: meprin A subunit beta [Sus scrofa]	1.000	0.371	0.316	0.293	0.284
gi|311254039|ref|XP_003125724.1| PREDICTED: mucosal pentraxin-like [Sus scrofa]	1.000	0.420	0.286	0.227	0.291
gi|264681458|ref|NP_001161123.1| 2-acylglycerol O-acyltransferase 2 [Sus scrofa] >gi|262204904|dbj|BAI48032.1| monoacylglycerol O-acyltransferase 2 [Sus scrofa]	1.000	0.608	0.314	0.222	0.311
gi|298160993|ref|NP_001177169.1| glycerol-3-phosphate dehydrogenase [NAD+], cytoplasmic [Sus scrofa]	1.000	0.658	0.354	0.279	0.313
gi|346421378|ref|NP_001231061.1| serpin H1 precursor [Sus scrofa]	1.000	0.667	0.740	0.490	0.320
gi|347300323|ref|NP_001231460.1| thioredoxin-dependent peroxide reductase, mitochondrial [Sus scrofa]	1.000	0.606	0.676	0.513	0.331
gi|198282001|ref|NP_001123683.1| transcription factor A, mitochondrial precursor [Sus scrofa] >gi|75052621|sp|Q5D144.1|TFAM_PIG RecName: Full=Transcription factor A, mitochondrial; Short=mtTFA; Flags: Precursor >gi|60326706|gb|AAX18878.1| mitochondrial transcription factor A [Sus s	1.000	0.487	0.264	0.384	0.359
gi|350586831|ref|XP_003482287.1| PREDICTED: cytochrome c oxidase subunit 5A, mitochondrial-like isoform 1 [Sus scrofa] >gi|350586833|ref|XP_003482288.1| PREDICTED: cytochrome c oxidase subunit 5A, mitochondrial-like isoform 2 [Sus scrofa]	1.000	0.260	0.519	0.473	0.375
gi|226709210|sp|A1XQT2.1|COX6C_PIG RecName: Full=Cytochrome c oxidase subunit 6C; AltName: Full=Cytochrome c oxidase polypeptide VIc >gi|148225574|ref|NP_001090942.1| cytochrome c oxidase subunit 6C [Sus scrofa] >gi|117660798|gb|ABK55638.1| COX6C [Sus scrofa]	1.000	0.532	0.350	0.448	0.376
gi|350594035|ref|XP_003483822.1| PREDICTED: UDP-glucuronosyltransferase 1-10 isoform 3 [Sus scrofa]	1.000	0.561	0.348	0.404	0.378

gi|55926217|ref|NP_001007518.1| cytochrome c oxidase subunit 5B, mitochondrial precursor [Sus scrofa] >gi|75042739|sp|Q5S3G4.1|COX5B_PIG RecName: Full=Cytochrome c oxidase subunit 5B, mitochondrial; AltName: Full=Cytochrome c oxidase polypeptide Vb; Flags: Precursor >gi|55583747|g	1.000	0.467	0.331	0.397	0.379
gi|350538593|ref|NP_001233247.1| phosphoglucomutase-1 [Sus scrofa] >gi|321574209|gb|ADW94631.1| phosphoglucomutase 1 [Sus scrofa]	1.000	1.170	0.359	0.319	0.381
gi|350582932|ref|XP_003125549.2| PREDICTED: annexin A13 isoform 2 [Sus scrofa]	1.000	0.380	0.322	0.384	0.386
gi|335290692|ref|XP_003356250.1| PREDICTED: UDP-glucose 4-epimerase [Sus scrofa]	1.000	0.766	0.240	0.268	0.393
gi|350580438|ref|XP_003480823.1| PREDICTED: NADH dehydrogenase [ubiquinone] 1 beta subcomplex subunit 7-like [Sus scrofa]	1.000	0.407	0.396	0.258	0.394
gi|350584800|ref|XP_003355778.2| PREDICTED: cytochrome c oxidase subunit 4 isoform 1, mitochondrial-like [Sus scrofa]	1.000	0.500	0.278	0.305	0.408
gi|51317314|sp|P62802.2|H4_PIG RecName: Full=Histone H4 >gi|350583428|ref|XP_003481515.1| PREDICTED: histone H4-like [Sus scrofa] >gi|335291880|ref|XP_003356614.1| PREDICTED: histone H4-like [Sus scrofa] >gi|335287016|ref|XP_003355252.1| PREDICTED: histone H4-like [Sus scrofa] >g	1.000	0.435	0.532	0.445	0.417
gi|311259388|ref|XP_003128071.1| PREDICTED: cytochrome b-c1 complex subunit 6, mitochondrial-like [Sus scrofa]	1.000	0.606	0.434	0.581	0.418
gi|335281280|ref|XP_003122383.2| PREDICTED: apical endosomal glycoprotein [Sus scrofa]	1.000	0.502	0.431	0.468	0.419
gi|311246132|ref|XP_003122091.1| PREDICTED: 39S ribosomal protein L50, mitochondrial-like [Sus scrofa]	1.000	0.428	0.347	0.475	0.429
gi|335281047|ref|XP_003353724.1| PREDICTED: LOW QUALITY PROTEIN:	1.000	0.817	0.381	0.401	0.431

long-chain fatty acid transport protein 4 [Sus scrofa]					
gi|219521964|ref|NP_001137170.1| long-chain-fatty-acid--CoA ligase 3 [Sus scrofa] >gi|217314893|gb|ACK36981.1| acyl-CoA synthetase long-chain family member 3 [Sus scrofa]	1.000	0.559	0.389	0.335	0.432
gi|345090979|ref|NP_001230725.1| NADH dehydrogenase (ubiquinone) Fe-S protein 3, 30kDa (NADH-coenzyme Q reductase) [Sus scrofa]	1.000	0.510	0.285	0.248	0.434
gi|335299760|ref|XP_003358667.1| PREDICTED: neprilysin isoform 2 [Sus scrofa] >gi|311269573|ref|XP_003132549.1| PREDICTED: neprilysin isoform 1 [Sus scrofa]	1.000	0.571	0.385	0.373	0.435
gi|311264036|ref|XP_003129968.1| PREDICTED: 40S ribosomal protein S25-like isoform 2 [Sus scrofa] >gi|311264034|ref|XP_003129967.1| PREDICTED: 40S ribosomal protein S25-like isoform 1 [Sus scrofa]	1.000	0.408	0.174	0.194	0.439
gi|164507|gb|AAA51295.1| immunoglobulin gamma-chain [Sus scrofa]	1.000	0.635	0.488	0.566	0.442
gi|335284501|ref|XP_003124603.2| PREDICTED: cytochrome b-c1 complex subunit 2, mitochondrial-like [Sus scrofa]	1.000	0.886	0.408	0.423	0.451
gi|3913046|sp|O46409.1|APOA4_PIG RecName: Full=Apolipoprotein A-IV; Short=Apo-AIV; Short=ApoA-IV; AltName: Full=Apolipoprotein A4; Flags: Precursor >gi|47523830|ref|NP_999553.1| apolipoprotein A-IV precursor [Sus scrofa] >gi|2695742|emb|CAA11020.1| apolipoprotein A-IV [Sus scrofa]	1.000	0.558	0.390	0.478	0.451
gi|3746946|gb|AAC64131.1| phosphoglucomutase 1 [Sus scrofa] >gi|3746944|gb|AAC64130.1| phosphoglucomutase 1 [Sus scrofa]	1.000	0.855	0.665	0.452	0.453
gi|335297609|ref|XP_003358079.1| PREDICTED: synaptic vesicle membrane protein VAT-1 homolog [Sus scrofa]	1.000	0.788	0.386	0.515	0.459
gi|85687558|sp|Q4A3R3.1|DMBT1_PIG RecName: Full=Deleted in malignant brain	1.000	0.397	0.523	0.555	0.462

tumors 1 protein; AltName: Full=Hensin; Flags: Precursor >gi|73531021|emb|CAJ27171.1| DMBT1 protein [Sus scrofa]					
gi|335279750|ref|XP_003353424.1| PREDICTED: normal mucosa of esophagus-specific gene 1 protein-like isoform 2 [Sus scrofa] >gi|194034801|ref|XP_001925712.1| PREDICTED: normal mucosa of esophagus-specific gene 1 protein-like isoform 1 [Sus scrofa]	1.000	0.512	0.430	0.425	0.466
gi|346986456|ref|NP_001231378.1| nucleoside diphosphate kinase 3 isoform 1 precursor [Sus scrofa]	1.000	0.489	0.953	0.592	0.467
gi|51701269|sp|Q95334.1|AMPE_PIG RecName: Full=Glutamyl aminopeptidase; Short=EAP; AltName: Full=Aminopeptidase A; Short=AP-A; AltName: CD_antigen=CD249 >gi|47522856|ref|NP_999182.1| glutamyl aminopeptidase [Sus scrofa] >gi|1518865|gb|AAB07141.1| aminopeptidase A [Sus scrofa]	1.000	0.519	0.634	0.513	0.467
gi|264681460|ref|NP_001161124.1| acyl carrier protein, mitochondrial [Sus scrofa] >gi|262204908|dbj|BAI48034.1| NADH dehydrogenase (ubiquinone) 1, alpha/beta subcomplex, 1, 8kDa [Sus scrofa]	1.000	0.350	0.688	0.513	0.468
gi|347658971|ref|NP_001231613.1| ATP synthase, H+ transporting, mitochondrial Fo complex, subunit d [Sus scrofa]	1.000	0.489	0.543	0.446	0.473
gi|350581874|ref|XP_003124716.3| PREDICTED: rab11 family-interacting protein 3-like [Sus scrofa]	1.000	0.509	0.444	0.527	0.474
gi|136192|sp|P09571.2|TRFE_PIG RecName: Full=Serotransferrin; Short=Transferrin; AltName: Full=Beta-1 metal-binding globulin; AltName: Full=Siderophilin >gi|18655907|pdb|1H76|A Chain A, The Crystal Structure Of Diferric Porcine Serum Transferrin	1.000	0.803	0.460	0.442	0.476
gi|347300360|ref|NP_001231474.1| serine/arginine repetitive matrix protein 2 [Sus scrofa]	1.000	0.351	0.966	0.517	0.477
gi|311247022|ref|XP_003122449.1|	1.000	0.425	0.525	0.890	0.481

PREDICTED: cadherin-related family member 5 [Sus scrofa]					
gi|311248380|ref|XP_003123108.1| PREDICTED: cyclic AMP-responsive element-binding protein 3-like protein 3-like isoform 1 [Sus scrofa]	1.000	1.073	0.511	0.489	0.485
gi|194036463|ref|XP_001928684.1| PREDICTED: ras-related protein Rap-1A isoform 1 [Sus scrofa] >gi|335287176|ref|XP_003355289.1| PREDICTED: ras-related protein Rap-1A isoform 2 [Sus scrofa]	1.000	0.726	0.228	0.197	0.487
gi|47523730|ref|NP_999500.1| tropomyosin alpha-4 chain [Sus scrofa] >gi|54039746|sp|P67937.3|TPM4_PIG RecName: Full=Tropomyosin alpha-4 chain; AltName: Full=Tropomyosin-4 >gi|3661527|gb|AAC61744.1| tropomyosin 4 [Sus scrofa] >gi|262263207|dbj|BAI48106.1| tropomyosin 4 [Sus scro	1.000	0.496	0.857	0.634	0.489
gi|222090420|gb|ACM42418.1| carnitine palmitoyltransferase II [Sus scrofa]	1.000	1.253	0.307	0.223	0.492
gi|311257850|ref|XP_003127328.1| PREDICTED: 17-beta-hydroxysteroid dehydrogenase 14-like [Sus scrofa]	1.000	1.285	0.541	0.462	0.492
gi|114689|sp|P13618.1|ATP5J_PIG RecName: Full=ATP synthase-coupling factor 6, mitochondrial; Short=ATPase subunit F6	1.000	0.351	0.632	0.768	0.496
gi|121118|sp|P20305.1|GELS_PIG RecName: Full=Gelsolin; AltName: Full=Actin-depolymerizing factor; Short=ADF; AltName: Full=Brevin; Flags: Precursor >gi|164472|gb|AAA31042.1| plasma gelsolin precursor, partial [Sus scrofa] >gi|758306|emb|CAA32077.1| gelsolin [Sus scrofa]	1.000	0.313	0.654	0.571	0.497
gi|335310428|ref|XP_003362028.1| PREDICTED: surfeit locus protein 4-like, partial [Sus scrofa]	1.000	0.968	0.242	0.240	0.501
gi|147225127|emb|CAN13255.1| novel protein similar to butyrophilin family proteins [Sus scrofa]	1.000	0.673	0.418	0.595	0.502

gi|350592201|ref|XP_003359029.2| PREDICTED: hypothetical protein LOC100620809 isoform 1, partial [Sus scrofa]	1.000	0.604	0.664	0.798	0.503
gi|311252952|ref|XP_003125350.1| PREDICTED: ketohexokinase-like isoform 3 [Sus scrofa]	1.000	0.538	0.556	0.633	0.506
gi|297747368|ref|NP_001172101.1| ubiquinol-cytochrome c reductase binding protein [Sus scrofa]	1.000	0.352	0.887	1.053	0.506
gi|311275469|ref|XP_003134753.1| PREDICTED: calumenin isoform 2 [Sus scrofa]	1.000	0.129	1.092	0.556	0.512
gi|356614808|gb|AET25525.1| mitochondrial NADH dehydrogenase Fe-S protein 4 [Sus scrofa]	1.000	0.432	0.564	0.552	0.513
gi|344259276|gb|AEN03335.1| proteasome subunit beta type 6 [Sus scrofa] >gi|344259274|gb|AEN03334.1| proteasome subunit beta type 6 [Sus scrofa]	1.000	2.594	0.743	1.107	0.517
gi|350593495|ref|XP_003483700.1| PREDICTED: LOW QUALITY PROTEIN: glycerol-3-phosphate dehydrogenase, mitochondrial [Sus scrofa]	1.000	0.709	0.479	0.534	0.518
gi|311253020|ref|XP_003125380.1| PREDICTED: putative peptidyl-tRNA hydrolase PTRHD1-like [Sus scrofa]	1.000	0.406	0.392	0.647	0.521
gi|335290029|ref|XP_003356052.1| PREDICTED: nuclear pore glycoprotein p62 [Sus scrofa]	1.000	0.611	0.516	0.685	0.524
gi|47523548|ref|NP_999398.1| glutaredoxin-1 [Sus scrofa] >gi|121442|sp|P12309.2|GLRX1_PIG RecName: Full=Glutaredoxin-1; AltName: Full=Thioltransferase-1; Short=TTase-1 >gi|332368592|gb|AEE61370.1| glutaredoxin [Sus scrofa] >gi|326632057|gb|ADZ99031.1| glutaredoxin [Sus scrofa]	1.000	0.250	0.475	0.406	0.527
gi|350591601|ref|XP_003132498.3| PREDICTED: sodium/potassium-transporting ATPase subunit beta-3-like [Sus scrofa]	1.000	0.747	0.364	0.396	0.531
gi|350585425|ref|XP_003127517.3| PREDICTED: isochorismatase domain-containing protein 2, mitochondrial-like	1.000	0.409	1.462	1.207	0.534

isoform 1, partial [Sus scrofa]					
gi|311255413|ref|XP_001927240.2| PREDICTED: keratin, type II cytoskeletal 79 [Sus scrofa]	1.000	0.422	0.675	0.754	0.534
gi|148887343|sp|Q9MYT8.4|ATP5I_PIG RecName: Full=ATP synthase subunit e, mitochondrial; Short=ATPase subunit e	1.000	0.728	0.624	0.575	0.535
gi|350596379|ref|XP_003484266.1| PREDICTED: vesicle-trafficking protein SEC22b-like [Sus scrofa] >gi|350583458|ref|XP_003481524.1| PREDICTED: vesicle-trafficking protein SEC22b [Sus scrofa]	1.000	0.749	0.447	0.474	0.538
gi|4033507|sp|P08132.2|ANXA4_PIG RecName: Full=Annexin A4; AltName: Full=35-beta calcimedin; AltName: Full=Annexin IV; AltName: Full=Annexin-4; AltName: Full=Chromobindin-4; AltName: Full=Endonexin I; AltName: Full=Lipocortin IV; AltName: Full=P32.5; AltName: Full=PP4-X; AltName: F	1.000	0.597	0.527	0.325	0.539
gi|89216|pir||PT0220 Ig lambda chain V-C region PLC3 - pig (fragment)	1.000	0.534	0.840	0.664	0.541
gi|47522760|ref|NP_999131.1| long-chain 3-ketoacyl-CoA thiolase [Sus scrofa] >gi|6165556|gb|AAF04612.1|AF028007_1 long-chain 3-ketoacyl-CoA thiolase [Sus scrofa]	1.000	0.787	0.430	0.440	0.542
gi|194038067|ref|XP_001927944.1| PREDICTED: protein DEK isoform 1 [Sus scrofa]	1.000	0.564	0.737	1.077	0.544
gi|350583970|ref|XP_003126216.3| PREDICTED: keratin, type II cytoskeletal 75-like [Sus scrofa]	1.000	0.448	0.680	0.760	0.546
gi|350583385|ref|XP_001927905.3| PREDICTED: tuftelin, partial [Sus scrofa]	1.000	0.927	0.974	1.298	0.546
gi|350586576|ref|XP_001929450.4| PREDICTED: LOW QUALITY PROTEIN: peptidyl-prolyl cis-trans isomerase FKBP5 [Sus scrofa]	1.000	0.774	0.520	0.632	0.547
gi|347922148|ref|NP_001231673.1| charged multivesicular body protein 5 [Sus scrofa]	1.000	0.442	0.792	1.053	0.549
gi|347446687|ref|NP_001231527.1| glucosidase 2	1.000	0.456	0.593	0.593	0.549

subunit beta precursor [Sus scrofa]					
gi|350276134|ref|NP_001231877.1| LETM1 and EF-hand domain-containing protein 1, mitochondrial [Sus scrofa]	1.000	0.568	0.701	0.726	0.558
gi|350586356|ref|XP_003482168.1| PREDICTED: ribosyldihydronicotinamide dehydrogenase [quinone]-like [Sus scrofa]	1.000	1.234	0.977	1.294	0.558
gi|311245734|ref|XP_003121939.1| PREDICTED: hypothetical protein LOC100038023 [Sus scrofa] >gi|311245732|ref|XP_003121938.1| PREDICTED: hypothetical protein LOC100038023 [Sus scrofa]	1.000	0.464	0.315	0.345	0.559
gi|335291231|ref|XP_003356440.1| PREDICTED: desmoglein-2 [Sus scrofa]	1.000	0.805	0.477	0.561	0.564
gi|385862179|ref|NP_001245364.1| protein diaphanous homolog 1 [Sus scrofa]	1.000	0.625	0.552	0.764	0.564
gi|311276931|ref|XP_003135414.1| PREDICTED: U3 small nucleolar RNA-associated protein 14 homolog A-like isoform 1 [Sus scrofa]	1.000	0.440	0.533	0.443	0.578
gi|113205854|ref|NP_001038066.1| 40S ribosomal protein S3 [Sus scrofa] >gi|115502828|sp|Q0Z8U2.1|RS3_PIG RecName: Full=40S ribosomal protein S3 >gi|110006645|gb|ABG48767.1| ribosomal protein S3 [Sus scrofa]	1.000	0.590	0.430	0.367	0.585
gi|335308509|ref|XP_003361258.1| PREDICTED: microsomal glutathione S-transferase 2-like, partial [Sus scrofa]	1.000	0.711	0.282	0.335	0.585
gi|122142619|sp|Q19S50.1|STAT3_PIG RecName: Full=Signal transducer and activator of transcription 3 >gi|113205774|ref|NP_001038045.1| signal transducer and activator of transcription 3 [Sus scrofa] >gi|315273320|gb|ADU03227.1| signal transducer and activator of transcription 3 isofor	1.000	0.697	0.816	0.729	0.586
gi|54873401|gb|AAV40980.1| heat shock 60 kDa protein 1 [Sus scrofa]	1.000	1.229	0.463	0.450	0.587
gi|335310769|ref|XP_003362184.1| PREDICTED: THO complex subunit 4-like, partial [Sus scrofa]	1.000	0.639	0.688	0.742	0.587
gi|335309593|ref|XP_003361696.1|	1.000	0.419	0.930	0.562	0.587

PREDICTED: 39S ribosomal protein L12, mitochondrial-like, partial [Sus scrofa]					
gi|118675|sp|P09623.1|DLDH_PIG RecName: Full=Dihydrolipoyl dehydrogenase, mitochondrial; AltName: Full=Dihydrolipoamide dehydrogenase; Flags: Precursor >gi|47522940|ref|NP_999227.1| dihydrolipoyl dehydrogenase, mitochondrial precursor [Sus scrofa] >gi|164539|gb|AAA31069.1| lipoam	1.000	0.665	0.684	0.543	0.589
gi|311274155|ref|XP_003134215.1| PREDICTED: NADH dehydrogenase [ubiquinone] iron-sulfur protein 6, mitochondrial-like [Sus scrofa]	1.000	0.891	0.494	1.079	0.589
gi|148235351|ref|NP_001090944.1| NADH dehydrogenase ubiquinone flavoprotein 2 [Sus scrofa] >gi|117660947|gb|ABK55646.1| NDUFV2 [Sus scrofa]	1.000	0.501	0.531	0.567	0.591
gi|308081427|ref|NP_001183950.1| hydroxysteroid dehydrogenase-like protein 2 [Sus scrofa]	1.000	0.789	0.489	0.551	0.593
gi|350590640|ref|XP_003131788.3| PREDICTED: myosin-Id [Sus scrofa]	1.000	0.750	0.444	0.530	0.597
gi|116551|sp|P04404.2|CMGA_PIG RecName: Full=Chromogranin-A; Short=CgA; Contains: RecName: Full=Pancreastatin; Contains: RecName: Full=Parastatin; Contains: RecName: Full=WE-14; Flags: Precursor >gi|164417|gb|AAA31016.1| chromogranin A precursor, partial [Sus scrofa]	1.000	0.486	0.982	0.552	0.599
gi|46396062|sp|Q865F1.1|MTP_PIG RecName: Full=Microsomal triglyceride transfer protein large subunit; Flags: Precursor >gi|47523450|ref|NP_999350.1| microsomal triglyceride transfer protein large subunit precursor [Sus scrofa] >gi|28974486|gb|AAO61497.1| microsomal triglyceride tr	1.000	0.965	0.461	0.435	0.601
gi|298160936|ref|NP_001177146.1| transmembrane protein C9orf46 [Sus scrofa]	1.000	0.545	0.350	0.364	0.601
gi|47522662|ref|NP_999076.1| membrane-associated progesterone receptor component 1 [Sus	1.000	0.562	0.373	0.608	0.603

scrofa] >gi|6647580|sp|Q95250.3|PGRC1_PIG RecName: Full=Membrane-associated progesterone receptor component 1 >gi|1657409|emb|CAA68050.1| steroid membrane binding protein [Sus scrofa]					
gi|350585373|ref|XP_003127406.3| PREDICTED: myosin-14-like [Sus scrofa]	1.000	0.667	0.776	0.842	0.605
gi|194037554|ref|XP_001929445.1| PREDICTED: ATP synthase subunit beta, mitochondrial [Sus scrofa]	1.000	0.748	0.423	0.355	0.607
gi|299611|gb|AAB26236.1| cerebroside sulfate activator, CS-Act [swine, kidneys, Peptide, 79 aa]	1.000	0.957	0.947	1.163	0.607
gi|47523768|ref|NP_999520.1| epoxide hydrolase 1 precursor [Sus scrofa] >gi|75069447|sp|P79381.1|HYEP_PIG RecName: Full=Epoxide hydrolase 1; AltName: Full=Epoxide hydratase; AltName: Full=Microsomal epoxide hydrolase >gi|1840391|dbj|BAA19200.1| epoxide hydrolase [Sus scrofa]	1.000	0.733	0.492	0.311	0.608
gi|350582175|ref|XP_003481218.1| PREDICTED: mitochondrial inner membrane protein, partial [Sus scrofa]	1.000	0.546	0.513	0.778	0.610
gi|350584002|ref|XP_003126228.3| PREDICTED: keratin, type I cytoskeletal 18 [Sus scrofa]	1.000	0.903	0.753	0.534	0.611
gi|311258550|ref|XP_003127676.1| PREDICTED: EF-hand domain-containing protein D2-like [Sus scrofa]	1.000	0.429	0.804	0.768	0.614
gi|2258465|gb|AAB94003.1| succinyl-CoA synthetase alpha subunit [Sus scrofa]	1.000	0.610	0.582	0.448	0.616
gi|311253799|ref|XP_001926739.2| PREDICTED: ras-related protein Rab-2A [Sus scrofa]	1.000	1.027	0.390	0.427	0.617
gi|349732227|ref|NP_001231859.1| heterogeneous nuclear ribonucleoprotein M [Sus scrofa]	1.000	0.574	0.507	0.477	0.618
gi|5542425|pdb|1QPW|D Chain D, Crystal Structure Determination Of Porcine Hemoglobin At 1.8a Resolution >gi|5542423|pdb|1QPW|B Chain B, Crystal Structure Determination Of Porcine Hemoglobin At 1.8a	1.000	0.842	0.728	0.606	0.619

Resolution >gi|809285|pdb|2PGH|D Chain D, Structure Determination Of Aquo					
gi|297632426|ref|NP_001172096.1| signal sequence receptor, alpha [Sus scrofa]	1.000	0.756	0.387	0.391	0.624
gi|47523792|ref|NP_999532.1| catenin beta-1 [Sus scrofa] >gi|167834655|gb|ACA03158.1| beta-catenin [Sus scrofa] >gi|18146999|dbj|BAB82984.1| beta-catenin [Sus scrofa]	1.000	0.615	0.612	0.533	0.625
gi|311254317|ref|XP_001927973.2| PREDICTED: cingulin-like [Sus scrofa]	1.000	0.549	0.991	0.920	0.625
gi|194038586|ref|XP_001926746.1| PREDICTED: transmembrane emp24 domain-containing protein 10 [Sus scrofa]	1.000	0.582	0.399	0.461	0.628
gi|47522864|ref|NP_999186.1| unconventional myosin-VI [Sus scrofa] >gi|75039721|sp|Q29122.1|MYO6_PIG RecName: Full=Unconventional myosin-VI; AltName: Full=Unconventional myosin-6 >gi|516155|emb|CAA84559.1| unconventional myosin [Sus scrofa]	1.000	0.847	0.744	0.911	0.630
gi|343780950|ref|NP_001230488.1| 39S ribosomal protein L40, mitochondrial isoform 1 [Sus scrofa]	1.000	0.573	0.577	0.466	0.630
gi|297747302|ref|NP_001172115.1| aspartyl aminopeptidase [Sus scrofa]	1.000	0.913	2.217	1.765	0.631
gi|311257294|ref|XP_003127050.1| PREDICTED: cytochrome b-c1 complex subunit Rieske, mitochondrial-like [Sus scrofa]	1.000	0.723	0.545	0.722	0.632
gi|347582660|ref|NP_001231584.1| mesencephalic astrocyte-derived neurotrophic factor precursor [Sus scrofa] >gi|350591263|ref|XP_003483237.1| PREDICTED: mesencephalic astrocyte-derived neurotrophic factor [Sus scrofa] >gi|306563669|gb|ADN03186.1| mesencephalic astrocyte-derived neu	1.000	0.557	0.812	0.705	0.639
gi|350579385|ref|XP_001925894.4| PREDICTED: glyoxylate reductase/hydroxypyruvate reductase [Sus scrofa]	1.000	0.895	0.493	0.623	0.640
gi|47523892|ref|NP_999584.1| epithelial cell adhesion molecule precursor [Sus	1.000	0.935	0.504	0.553	0.642

scrofa] >gi|75045149|sp|Q75QW1.1|EPCAM_PIG RecName: Full=Epithelial cell adhesion molecule; Short=Ep-CAM; AltName: Full=Tumor-associated calcium signal transducer 1; AltName: CD_antigen=CD326; Flags:					
gi|350591864|ref|XP_001926353.4| PREDICTED: LOW QUALITY PROTEIN: melanotransferrin [Sus scrofa]	1.000	0.828	0.796	1.070	0.643
gi|335285948|ref|XP_003131991.2| PREDICTED: very long-chain specific acyl-CoA dehydrogenase, mitochondrial-like isoform 1 [Sus scrofa]	1.000	1.314	0.725	0.661	0.646
gi|262072813|dbj|BAI47714.1| hydroxysteroid (17-beta) dehydrogenase 12 [Sus scrofa]	1.000	0.728	0.376	0.498	0.648
gi|335292368|ref|XP_001926489.3| PREDICTED: calcium and integrin-binding protein 1 [Sus scrofa]	1.000	0.454	0.700	0.817	0.648
gi|350587465|ref|XP_003128987.3| PREDICTED: RNA-binding protein 47-like isoform 1 [Sus scrofa]	1.000	0.543	0.435	0.259	0.650
gi|311273235|ref|XP_003133777.1| PREDICTED: intestinal-type alkaline phosphatase-like isoform 2 [Sus scrofa]	1.000	0.879	1.307	2.392	0.652
gi|256838109|ref|NP_001157984.1| phosphate carrier protein, mitochondrial [Sus scrofa] >gi|255964672|gb|ACU44653.1| solute carrier family 25 member 3 [Sus scrofa]	1.000	0.704	0.387	0.381	0.659
gi|350596559|ref|XP_003361362.2| PREDICTED: ectonucleotide pyrophosphatase/phosphodiesterase family member 3-like, partial [Sus scrofa]	1.000	0.727	0.735	0.828	0.662
gi|350582036|ref|XP_003481179.1| PREDICTED: U5 small nuclear ribonucleoprotein 200 kDa helicase [Sus scrofa]	1.000	0.686	0.793	0.968	0.662
gi|359465576|ref|NP_001240753.1| A-kinase anchor protein 9 [Sus scrofa]	1.000	0.772	1.137	0.982	0.663
gi|350276172|ref|NP_001231880.1| leucine-rich repeat-containing protein 59 [Sus scrofa]	1.000	0.495	0.761	0.604	0.665
gi|335302040|ref|XP_001927564.2| PREDICTED: delta-1-pyrroline-5-carboxylate synthase isoform 1 [Sus scrofa]	1.000	0.834	0.466	0.373	0.665
gi|350589440|ref|XP_003130687.3|	1.000	0.268	0.679	0.831	0.666

PREDICTED: Golgi membrane protein 1-like [Sus scrofa]					
gi|164359|gb|AAA30992.1| apolipoprotein A-I [Sus scrofa]	1.000	1.067	0.411	0.499	0.666
gi|54020966|ref|NP_001005726.1| annexin A2 [Sus scrofa] >gi|148876771|sp|P19620.4|ANXA2_PIG RecName: Full=Annexin A2; AltName: Full=Annexin II; AltName: Full=Annexin-2; AltName: Full=Calpactin I heavy chain; AltName: Full=Calpactin-1 heavy chain; AltName: Full=Chromobindin-8; AltN	1.000	0.905	0.473	0.453	0.667
gi|311273928|ref|XP_003134106.1| PREDICTED: nucleophosmin-like isoform 2 [Sus scrofa]	1.000	0.919	1.022	1.439	0.668
gi|305855176|ref|NP_001182260.1| cleavage and polyadenylation specificity factor subunit 6 [Sus scrofa] >gi|285818464|gb|ADC38905.1| cleavage and polyadenylation specific factor 6 [Sus scrofa]	1.000	0.757	1.403	1.440	0.672
gi|194038359|ref|XP_001929028.1| PREDICTED: glutaredoxin-related protein 5, mitochondrial-like [Sus scrofa]	1.000	1.169	0.703	0.919	0.677
gi|335297415|ref|XP_003358038.1| PREDICTED: 60S ribosomal protein L38-like isoform 2 [Sus scrofa] >gi|311266862|ref|XP_003131289.1| PREDICTED: 60S ribosomal protein L38-like isoform 1 [Sus scrofa]	1.000	0.659	0.354	0.290	0.677
gi|350594715|ref|XP_001927122.3| PREDICTED: adipocyte plasma membrane-associated protein-like [Sus scrofa]	1.000	0.721	0.215	0.200	0.678
gi|147899784|ref|NP_001090887.1| elongation factor 1-alpha 1 [Sus scrofa] >gi|223019599|emb|CAX36487.1| eukaryotic translation elongation factor 1 alpha 1 [Sus scrofa] >gi|223019597|emb|CAX36486.1| eukaryotic translation elongation factor 1 alpha 1 [Sus scrofa] >gi|110287842|gb|ABG	1.000	0.764	0.570	0.438	0.680
gi|346986400|ref|NP_001231351.1| kunitz-type protease inhibitor 1 precursor [Sus scrofa]	1.000	0.573	0.736	0.762	0.681
gi|311259030|ref|XP_003127902.1| PREDICTED: small nuclear ribonucleoprotein Sm D1-like [Sus scrofa]	1.000	1.410	1.528	1.301	0.682

gi|37962872|gb|AAR05785.1| cytochrome c oxidase subunit 2 [Sus scrofa] >gi|162423271|gb|ABX89412.1| cytochrome c oxidase subunit 2 [Sus scrofa]	1.000	0.868	0.423	0.338	0.684
gi|335310669|ref|XP_003362140.1| PREDICTED: hypothetical protein LOC100622909 [Sus scrofa]	1.000	0.605	0.902	0.685	0.684
gi|349501107|ref|NP_001231795.1| ribosomal protein, large, P2 [Sus scrofa]	1.000	0.630	1.484	1.372	0.685
gi|47523888|ref|NP_999583.1| ADP/ATP translocase 3 [Sus scrofa] >gi|52000613|sp|Q6QRN9.3|ADT3_PIG RecName: Full=ADP/ATP translocase 3; AltName: Full=ADP,ATP carrier protein 3; AltName: Full=Adenine nucleotide translocator 3; Short=ANT 3; AltName: Full=Solute carrier family 25 m	1.000	0.649	0.471	0.325	0.687
gi|350594057|ref|XP_003359743.2| PREDICTED: leucine-rich repeat flightless-interacting protein 2-like [Sus scrofa]	1.000	0.725	0.781	1.048	0.688
gi|148222268|ref|NP_001090952.1| tropomyosin alpha-1 chain [Sus scrofa] >gi|158931149|sp|P42639.2|TPM1_PIG RecName: Full=Tropomyosin alpha-1 chain; AltName: Full=Alpha-tropomyosin; AltName: Full=Tropomyosin-1 >gi|117661180|gb|ABK55659.1| TPM1 [Sus scrofa]	1.000	0.641	1.115	1.002	0.688
gi|290463427|sp|Q0QF01.2|DHSA_PIG RecName: Full=Succinate dehydrogenase [ubiquinone] flavoprotein subunit, mitochondrial; AltName: Full=Flavoprotein subunit of complex II; Short=Fp; Flags: Precursor	1.000	0.678	0.605	0.674	0.695
gi|311268489|ref|XP_003132080.1| PREDICTED: fatty aldehyde dehydrogenase-like [Sus scrofa]	1.000	0.804	0.424	0.401	0.695
gi|311251336|ref|XP_003124563.1| PREDICTED: elongation factor Tu, mitochondrial-like [Sus scrofa]	1.000	0.973	0.501	0.508	0.696
gi|164302|gb|AAA30983.1| alpha-1 acid glycoprotein, partial [Sus scrofa]	1.000	0.263	0.696	0.494	0.699
gi|54039371|sp|P62272.3|RS18_PIG RecName: Full=40S ribosomal protein S18 >gi|47522714|ref|NP_999105.1| 40S	1.000	0.629	0.226	0.178	0.700

ribosomal protein S18 [Sus scrofa] >gi|1841304|dbj|BAA19211.1| ribosomal protein [Sus scrofa]					
gi|350593231|ref|XP_003359478.2| PREDICTED: lactase-phlorizin hydrolase-like [Sus scrofa]	1.000	0.691	0.999	0.733	0.700
gi|345100481|pdb|1C9P|A Chain A, Complex Of Bdellastasin With Porcine Trypsin >gi|3318722|pdb|1AN1|E Chain E, Leech-Derived Tryptase InhibitorTRYPSIN COMPLEX	1.000	0.612	0.894	0.765	0.701
gi|156120140|ref|NP_001095287.1| helicase SKI2W [Sus scrofa] >gi|148724903|emb|CAN87691.1| superkiller viralicidic activity 2-like (S. cerevisiae) [Sus scrofa]	1.000	0.943	1.061	1.130	0.702
gi|335304027|ref|XP_003134070.2| PREDICTED: 28S ribosomal protein S36, mitochondrial-like [Sus scrofa]	1.000	0.469	0.658	0.771	0.704
gi|335288150|ref|XP_003126347.2| PREDICTED: prolow-density lipoprotein receptor-related protein 1-like [Sus scrofa]	1.000	0.776	0.558	0.530	0.707
gi|47523726|ref|NP_999498.1| dolichyl-diphosphooligosaccharide--protein glycosyltransferase subunit 1 precursor [Sus scrofa] >gi|75056164|sp|Q9GMB0.1|RPN1_PIG RecName: Full=Dolichyl-diphosphooligosaccharide--protein glycosyltransferase subunit 1; AltName: Full=Dolichyl-diphosph	1.000	0.625	0.627	0.547	0.709
gi|78172717|gb|ABB29449.1| transporter associated with antigen processing 1 [Sus scrofa]	1.000	0.541	0.382	0.447	0.711
gi|164318|gb|AAA30988.1| albumin, partial [Sus scrofa]	1.000	1.129	0.611	0.569	0.712
gi|346644799|ref|NP_001231157.1| thymopoietin [Sus scrofa]	1.000	0.723	0.984	0.854	0.716
gi|335299041|ref|XP_001926664.3| PREDICTED: cytochrome b-c1 complex subunit 1, mitochondrial [Sus scrofa]	1.000	0.764	0.572	0.812	0.720
gi|350589278|ref|XP_003130571.3| PREDICTED: melanoma inhibitory activity protein 3-like, partial [Sus scrofa]	1.000	0.752	1.073	0.757	0.720
gi|164382|gb|AAA31002.1| Na+, K+-ATPase	1.000	0.797	0.422	0.473	0.725

alpha-subunit precursor [Sus scrofa]					
gi|347300243|ref|NP_001231430.1| glutamate dehydrogenase 1, mitochondrial [Sus scrofa]	1.000	0.826	0.563	0.527	0.727
gi|47522750|ref|NP_999125.1| voltage-dependent anion-selective channel protein 1 [Sus scrofa] >gi|75050406|sp|Q9MZ16.3|VDAC1_PIG RecName: Full=Voltage-dependent anion-selective channel protein 1; Short=VDAC-1 >gi|8745552|gb|AAF78963.1|AF268461_1 voltage-dependent anion channel	1.000	0.661	0.450	0.434	0.729
gi|8569334|pdb|1EIO|A Chain A, Ileal Lipid Binding Protein In Complex With Glycocholate	1.000	1.497	1.823	0.959	0.734
gi|448581|prf||1917268B pyruvate dehydrogenase:SUBUNIT=beta	1.000	1.030	0.518	0.699	0.734
gi|350584479|ref|XP_003126643.3| PREDICTED: bcl-2-like protein 13 [Sus scrofa]	1.000	0.678	1.003	0.721	0.739
gi|347300207|ref|NP_001231417.1| nucleobindin-1 precursor [Sus scrofa]	1.000	0.672	1.095	1.007	0.739
gi|343072|gb|AAA32030.1| ATPase 8, partial (mitochondrion) [Sus scrofa]	1.000	0.683	0.285	0.431	0.745
gi|1346670|sp|P04175.2|NCPR_PIG RecName: Full=NADPH--cytochrome P450 reductase; Short=CPR; Short=P450R	1.000	0.722	0.432	0.709	0.752
gi|311270597|ref|XP_003132922.1| PREDICTED: 26S proteasome non-ATPase regulatory subunit 9-like [Sus scrofa]	1.000	0.599	1.217	1.425	0.755
gi|178056556|ref|NP_001116620.1| probable aminopeptidase NPEPL1 [Sus scrofa] >gi|147223400|emb|CAN13197.1| aminopeptidase-like 1 [Sus scrofa]	1.000	1.378	2.150	2.596	0.755
gi|12231040|sp|Q29048.2|VATA_PIG RecName: Full=V-type proton ATPase catalytic subunit A; Short=V-ATPase subunit A; AltName: Full=V-ATPase 69 kDa subunit; AltName: Full=Vacuolar proton pump subunit alpha	1.000	0.868	0.558	0.642	0.755
gi|311274849|ref|XP_001925742.2| PREDICTED: regulation of nuclear pre-mRNA domain-containing protein 1B [Sus scrofa]	1.000	0.672	1.313	0.928	0.755
gi|113205886|ref|NP_001038075.1| nucleoside diphosphate kinase B [Sus scrofa] >gi|115311824|sp|Q2EN76.1|NDKB_PIG RecName: Full=Nucleoside diphosphate kinase	1.000	0.829	1.823	1.107	0.757

B; Short=NDK B; Short=NDP kinase B; AltName: Full=Histidine protein kinase NDKB >gi|87047646|gb|ABD18456.1| nm23-H2 [Sus scro					
gi|350580184|ref|XP_003353965.2| PREDICTED: reticulocalbin-1-like isoform 2 [Sus scrofa]	1.000	0.631	0.986	1.263	0.758
gi|350596353|ref|XP_003361080.2| PREDICTED: gamma-soluble NSF attachment protein-like [Sus scrofa]	1.000	0.767	0.636	0.846	0.758
gi|342349319|ref|NP_001230139.1| calnexin precursor [Sus scrofa]	1.000	0.685	0.549	0.700	0.759
gi|194036296|ref|XP_001926524.1| PREDICTED: splicing factor 3B subunit 4 [Sus scrofa]	1.000	0.862	1.231	1.161	0.760
gi|350580651|ref|XP_003480867.1| PREDICTED: scaffold attachment factor B1-like isoform 2 [Sus scrofa]	1.000	0.687	1.076	0.762	0.763
gi|335278800|ref|XP_003121142.2| PREDICTED: afadin [Sus scrofa]	1.000	0.568	1.225	1.062	0.765
gi|350582773|ref|XP_003481353.1| PREDICTED: 40S ribosomal protein S7-like isoform 2 [Sus scrofa] >gi|350582771|ref|XP_003481352.1| PREDICTED: 40S ribosomal protein S7-like isoform 1 [Sus scrofa]	1.000	0.624	0.404	0.255	0.765
gi|346421419|ref|NP_001231084.1| lysosome membrane protein 2 precursor [Sus scrofa]	1.000	0.696	0.520	0.332	0.768
gi|335285324|ref|XP_003354827.1| PREDICTED: prenylcysteine oxidase-like [Sus scrofa]	1.000	1.016	0.403	0.147	0.770
gi|47522680|ref|NP_999069.1| neutral alpha-glucosidase AB precursor [Sus scrofa] >gi|54037147|sp|P79403.1|GANAB_PIG RecName: Full=Neutral alpha-glucosidase AB; AltName: Full=Alpha-glucosidase 2; AltName: Full=Glucosidase II subunit alpha; Flags: Precursor >gi|1890664|gb|AAB4975	1.000	0.917	0.548	0.547	0.771
gi|304365428|ref|NP_001182041.1| protein disulfide-isomerase A3 precursor [Sus scrofa] >gi|301016769|dbj|BAJ11757.1| glucose regulated protein 58 [Sus scrofa]	1.000	0.492	1.192	1.152	0.772
gi|1710093|sp|P50121.2|RET2_PIG RecName:	1.000	1.386	0.766	0.800	0.777

Full=Retinol-binding protein 2; AltName: Full=Cellular retinol-binding protein II; Short=CRBP-II >gi|55741711|ref|NP_999616.1| retinol-binding protein 2 [Sus scrofa] >gi|455523|emb|CAA54729.1| cellular retinol binding protein II [Sus scrofa					
gi|305855170|ref|NP_001182250.1| long-chain-fatty-acid--CoA ligase 5 [Sus scrofa] >gi|285818418|gb|ADC38882.1| acyl-CoA synthetase long-chain family member 5 [Sus scrofa]	1.000	0.820	0.530	0.574	0.782
gi|178056623|ref|NP_001116685.1| vesicle-associated membrane protein-associated protein B [Sus scrofa] >gi|160210890|sp|A5GFS8.1|VAPB_PIG RecName: Full=Vesicle-associated membrane protein-associated protein B; Short=VAMP-B; Short=VAMP-associated protein B; Short=VAP-B >gi|147223393	1.000	0.473	0.849	0.623	0.782
gi|47522846|ref|NP_999175.1| ATP-binding cassette sub-family G member 2 [Sus scrofa] >gi|68051987|sp|Q8MIB3.1|ABCG2_PIG RecName: Full=ATP-binding cassette sub-family G member 2; AltName: Full=Brain multidrug resistance protein; AltName: CD_antigen=CD338 >gi|21530795|emb|CAD1278	1.000	1.247	0.674	0.582	0.785
gi|1921|emb|CAA33169.1| unnamed protein product [Sus scrofa]	1.000	0.735	0.558	0.573	0.786
gi|47522608|ref|NP_999106.1| dolichyl-diphosphooligosaccharide--protein glycosyltransferase subunit 2 precursor [Sus scrofa] >gi|75056105|sp|Q9GL01.1|RPN2_PIG RecName: Full=Dolichyl-diphosphooligosaccharide--protein glycosyltransferase subunit 2; AltName: Full=Dolichyl-diphosph	1.000	0.966	0.550	0.412	0.788
gi|335293186|ref|XP_003356895.1| PREDICTED: grpE protein homolog 1, mitochondrial-like isoform 1 [Sus scrofa]	1.000	0.674	0.743	0.608	0.791
gi|311267276|ref|XP_003131485.1| PREDICTED: keratin, type I cytoskeletal 19-like [Sus scrofa]	1.000	0.627	0.634	0.765	0.792

gi|230506|pdb|2FBP|A Chain A, Structure Refinement Of Fructose-1,6-Bisphosphatase And Its Fructose 2,6-Bisphosphate Complex At 2.8 Angstroms Resolution >gi|230507|pdb|2FBP|B Chain B, Structure Refinement Of Fructose-1,6-Bisphosphatase And Its Fructose 2,6-Bisphosphate C	1.000	1.622	1.109	0.814	0.793
gi|335281686|ref|XP_003353872.1| PREDICTED: UBX domain-containing protein 1-like [Sus scrofa]	1.000	0.843	1.119	0.973	0.796
gi|340007402|ref|NP_001229989.1| RAB10, member RAS oncogene family [Sus scrofa]	1.000	0.916	0.388	0.456	0.797
gi|343790888|ref|NP_001230502.1| uncharacterized protein LOC100627352 [Sus scrofa]	1.000	0.852	0.333	0.465	0.797
gi|194037097|ref|XP_001927840.1| PREDICTED: carbonic anhydrase 2 [Sus scrofa]	1.000	0.798	1.380	1.495	0.798
gi|297591975|ref|NP_001172071.1| ATP synthase subunit alpha, mitochondrial [Sus scrofa] >gi|187370717|gb|ACD02421.1| cardiac muscle ATP synthase H+ transporting mitochondrial F1 complex alpha subunit 1 [Sus scrofa]	1.000	0.768	0.706	0.723	0.799
gi|350594669|ref|XP_001926037.4| PREDICTED: ribosome-binding protein 1 [Sus scrofa]	1.000	0.571	1.008	0.992	0.800
gi|194042439|ref|XP_001928012.1| PREDICTED: ATPase family AAA domain-containing protein 1 isoform 1 [Sus scrofa]	1.000	0.481	1.189	1.064	0.800
gi|346644830|ref|NP_001231167.1| sulfide:quinone oxidoreductase, mitochondrial [Sus scrofa]	1.000	0.889	0.519	0.467	0.803
gi|335309807|ref|XP_003361778.1| PREDICTED: delta-aminolevulinic acid dehydratase-like, partial [Sus scrofa]	1.000	0.853	1.284	1.316	0.803
gi|148747594|ref|NP_001092068.1| 60S acidic ribosomal protein P0 [Sus scrofa] >gi|182705234|sp|Q29214.2|RLA0_PIG RecName: Full=60S acidic ribosomal protein P0; AltName: Full=60S ribosomal protein L10E >gi|83854099|gb|ABC47963.1| ribosomal phosphoprotein large PO subunit [Sus scrofa	1.000	0.785	0.612	0.651	0.807

gi|1236646|gb|AAC48775.1| ch4 and secrete domains of swine IgM [Sus scrofa]	1.000	0.779	0.684	0.478	0.807
gi|311277261|ref|XP_003135572.1| PREDICTED: 55 kDa erythrocyte membrane protein-like isoform 1 [Sus scrofa]	1.000	0.801	0.558	0.535	0.811
gi|47522948|ref|NP_999231.1| D-amino-acid oxidase [Sus scrofa] >gi|129305|sp|P00371.2|OXDA_PIG RecName: Full=D-amino-acid oxidase; Short=DAAO; Short=DAMOX; Short=DAO >gi|164306|gb|AAA30985.1| D-amino acid oxidase [Sus scrofa] >gi|48425898|pdb|1VE9|A Chain A, Porcine Kidney D-Am	1.000	1.109	0.677	0.555	0.816
gi|335299628|ref|XP_001926139.3| PREDICTED: coatomer subunit beta~ [Sus scrofa]	1.000	0.808	0.654	0.856	0.817
gi|311247240|ref|XP_003122553.1| PREDICTED: splicing factor 3B subunit 2 [Sus scrofa]	1.000	0.716	1.012	0.897	0.818
gi|194037681|ref|XP_001926390.1| PREDICTED: early endosome antigen 1 [Sus scrofa]	1.000	0.749	1.226	1.334	0.818
gi|335310208|ref|XP_003361930.1| PREDICTED: NADH dehydrogenase [ubiquinone] 1 beta subcomplex subunit 4-like [Sus scrofa]	1.000	1.201	0.436	0.449	0.822
gi|311254645|ref|XP_003125912.1| PREDICTED: EPS8-like 3 [Sus scrofa]	1.000	0.870	0.538	0.696	0.824
gi|194042318|ref|XP_001928917.1| PREDICTED: putative hexokinase HKDC1 [Sus scrofa]	1.000	0.716	0.557	0.679	0.825
gi|335283403|ref|XP_003123930.2| PREDICTED: lamin-B1 [Sus scrofa]	1.000	0.698	0.956	0.966	0.827
gi|343887442|ref|NP_001230623.1| heterogeneous nuclear ribonucleoprotein C (C1/C2) 2 [Sus scrofa]	1.000	0.738	0.645	0.614	0.827
gi|47523672|ref|NP_999468.1| 2~-5~-oligoadenylate synthase 1 [Sus scrofa] >gi|6226834|sp|Q29599.3|OAS1_PIG RecName: Full=2~-5~-oligoadenylate synthase 1; Short=(2-5~)oligo(A) synthase 1; Short=2-5A synthase 1; AltName: Full=p42	1.000	0.885	0.462	0.342	0.831

OAS >gi|3451331|emb|CAA12397.1| 2~-5~ oligoadenyla					
gi|353819|prf||1106188C cytochrome b5	1.000	0.588	0.840	0.666	0.833
gi|350587379|ref|XP_003482398.1| PREDICTED: putative aminopeptidase C13A11.05-like [Sus scrofa]	1.000	2.105	2.722	3.623	0.835
gi|194042189|ref|XP_001924213.1| PREDICTED: annexin A11 [Sus scrofa]	1.000	1.218	0.642	0.780	0.835
gi|311247118|ref|XP_003122486.1| PREDICTED: NADH dehydrogenase [ubiquinone] flavoprotein 1, mitochondrial-like isoform 1 [Sus scrofa]	1.000	1.053	0.587	0.656	0.836
gi|387912908|sp|P28491.3|CALR_PIG RecName: Full=Calreticulin; AltName: Full=CRP55; AltName: Full=Calregulin; AltName: Full=Endoplasmic reticulum resident protein 60; Short=ERp60; AltName: Full=HACBP; Flags: Precursor >gi|291622246|ref|NP_001167604.1| calreticulin precursor [Sus scro	1.000	0.910	0.589	0.581	0.836
gi|183228163|gb|ACC59785.1| PRP19/PSO4 pre-mRNA processing factor 19-like protien [Sus scrofa]	1.000	1.040	1.003	0.467	0.837
gi|350588303|ref|XP_003129732.3| PREDICTED: serine/threonine-protein kinase PAK 1-like [Sus scrofa]	1.000	1.086	0.799	0.761	0.840
gi|335306763|ref|XP_003360560.1| PREDICTED: ubiquitin-like protein 4A-like isoform 1 [Sus scrofa]	1.000	0.800	0.778	0.894	0.843
gi|311269853|ref|XP_003132661.1| PREDICTED: choline-phosphate cytidylyltransferase A [Sus scrofa]	1.000	0.689	0.563	0.584	0.844
gi|37934198|gb|AAP68768.1| 2,4-dienoyl-CoA reductase [Sus scrofa]	1.000	1.330	0.905	0.922	0.844
gi|311259162|ref|XP_003127965.1| PREDICTED: far upstream element-binding protein 1 [Sus scrofa]	1.000	0.590	0.988	0.965	0.846
gi|194039403|ref|XP_001929632.1| PREDICTED: CDC5 cell division cycle 5-like [Sus scrofa]	1.000	0.541	0.668	0.720	0.847
gi|1165145|emb|CAA64477.1| annexin I [Sus scrofa]	1.000	0.423	0.578	0.912	0.848

gi|349585085|ref|NP_001231804.1| RAB11B, member RAS oncogene family [Sus scrofa]	1.000	1.031	0.664	0.520	0.848
gi|311260817|ref|XP_001925607.2| PREDICTED: protein PML isoform 1 [Sus scrofa]	1.000	0.605	0.668	0.609	0.849
gi|350586335|ref|XP_003356548.2| PREDICTED: nuclease-sensitive element-binding protein 1-like [Sus scrofa]	1.000	0.357	1.959	0.998	0.852
gi|158514030|sp|A1XQU3.1|RL14_PIG RecName: Full=60S ribosomal protein L14 >gi|148237282|ref|NP_001090947.1| 60S ribosomal protein L14 [Sus scrofa] >gi|117661006|gb|ABK55649.1| RPL14 [Sus scrofa]	1.000	1.034	0.626	0.479	0.855
gi|311273371|ref|XP_003133833.1| PREDICTED: NADH dehydrogenase [ubiquinone] 1 alpha subcomplex subunit 10, mitochondrial-like [Sus scrofa]	1.000	0.942	0.553	0.566	0.856
gi|146741296|dbj|BAF62303.1| keratin 1 [Sus scrofa]	1.000	0.933	1.375	0.846	0.856
gi|345090993|ref|NP_001230731.1| RAB8A, member RAS oncogene family [Sus scrofa]	1.000	0.701	0.514	0.564	0.856
gi|343887420|ref|NP_001230609.1| transcription elongation factor A protein 1 [Sus scrofa]	1.000	0.935	1.433	1.330	0.857
gi|311275164|ref|XP_003134601.1| PREDICTED: amiloride-sensitive amine oxidase [copper-containing] [Sus scrofa]	1.000	0.807	0.699	0.984	0.858
gi|171986635|gb|ACB59309.1| intelectin 2 [Sus scrofa]	1.000	1.352	0.631	0.358	0.858
gi|311276040|ref|XP_003135024.1| PREDICTED: LOW QUALITY PROTEIN: acyl-coenzyme A thioesterase 9, mitochondrial-like [Sus scrofa]	1.000	1.339	0.598	0.958	0.858
gi|350584106|ref|XP_003481667.1| PREDICTED: SWI/SNF complex subunit SMARCC2 [Sus scrofa]	1.000	0.755	0.898	0.717	0.863
gi|132566481|gb|ABO34136.1| bactericidal permeability increasing protein [Sus scrofa]	1.000	1.007	0.321	0.341	0.863
gi|311267326|ref|XP_003131510.1| PREDICTED: keratin, type I cytoskeletal 20-like [Sus scrofa]	1.000	0.640	0.647	0.745	0.864
gi|350581449|ref|XP_003481037.1|	1.000	1.102	0.709	0.749	0.867

PREDICTED: guanine nucleotide-binding protein G(I)/G(S)/G(T) subunit beta-2-like [Sus scrofa] >gi|311251041|ref|XP_003124417.1| PREDICTED: guanine nucleotide-binding protein G(I)/G(S)/G(T) subunit beta-2-like isoform 2 [Sus scrofa]					
gi|350539577|ref|NP_001233167.1| coatomer subunit gamma-2 [Sus scrofa] >gi|301087607|gb|ADK56184.1| coatomer protein complex subunit gamma 2 [Sus scrofa]	1.000	1.059	0.472	0.381	0.867
gi|238018148|ref|NP_001153899.1| ADP-ribosylation factor 1 [Sus scrofa] >gi|212725657|gb|ACJ38117.1| ADP-ribosylation factor 1 [Sus scrofa] >gi|237638742|gb|ACR07973.1| ADP-ribosylation factor 1 [Sus scrofa]	1.000	0.779	0.435	0.407	0.868
gi|345441771|ref|NP_001230857.1| aldolase C, fructose-bisphosphate [Sus scrofa]	1.000	1.123	0.698	1.148	0.868
gi|350595677|ref|XP_003360362.2| PREDICTED: GRIP1-associated protein 1 [Sus scrofa]	1.000	0.784	1.938	1.963	0.870
gi|335296241|ref|XP_003357722.1| PREDICTED: ladinin-1-like [Sus scrofa]	1.000	0.587	0.680	0.710	0.870
gi|350591788|ref|XP_003358782.2| PREDICTED: hypothetical protein LOC448846 [Sus scrofa]	1.000	1.439	0.714	0.869	0.874
gi|335310107|ref|XP_003361890.1| PREDICTED: non-POU domain-containing octamer-binding protein [Sus scrofa]	1.000	1.266	0.594	0.636	0.877
gi|194043083|ref|XP_001928729.1| PREDICTED: malectin-like [Sus scrofa]	1.000	0.792	0.388	0.530	0.878
gi|194043861|ref|XP_001928233.1| PREDICTED: tubulin alpha-1D chain [Sus scrofa]	1.000	0.839	0.202	0.171	0.878
gi|70986480|gb|AAZ16514.1| hepatic nuclear factor 1 [Sus scrofa]	1.000	0.768	1.128	1.780	0.882
gi|640352|pdb|3MDE|A Chain A, Crystal Structures Of Medium Chain Acyl-Coa Dehydrogenase From Pig Liver Mitochondria With And Without Substrate >gi|640350|pdb|3MDD|A Chain A, Crystal Structures Of Medium Chain Acyl-Coa	1.000	1.391	0.684	0.685	0.883

Dehydrogenase From Pig Liver Mitochondria With And W					
gi|311265664|ref|XP_003130763.1| PREDICTED: LOW QUALITY PROTEIN: kinesin-1 heavy chain [Sus scrofa]	1.000	0.823	0.710	1.111	0.887
gi|85542092|sp|Q2YGT9.3|RL6_PIG RecName: Full=60S ribosomal protein L6 >gi|113205608|ref|NP_001038007.1| 60S ribosomal protein L6 [Sus scrofa] >gi|56384243|gb|AAV85770.1| ribosomal protein L6 [Sus scrofa]	1.000	0.806	0.295	0.432	0.890
gi|350595461|ref|XP_003134939.3| PREDICTED: LOW QUALITY PROTEIN: 2-oxoglutarate dehydrogenase, mitochondrial-like [Sus scrofa]	1.000	0.912	0.602	0.409	0.891
gi|298104076|ref|NP_001177104.1| enoyl-CoA hydratase, mitochondrial [Sus scrofa]	1.000	1.176	0.473	0.430	0.891
gi|335289671|ref|XP_003355951.1| PREDICTED: transcription elongation factor SPT5 isoform 3 [Sus scrofa] >gi|335289667|ref|XP_003355949.1| PREDICTED: transcription elongation factor SPT5 isoform 1 [Sus scrofa] >gi|335289669|ref|XP_003355950.1| PREDICTED: transcription elongation fac	1.000	0.921	1.108	1.490	0.892
gi|194036302|ref|XP_001926704.1| PREDICTED: histone H2A type 2-B-like [Sus scrofa]	1.000	0.779	1.057	1.179	0.893
gi|335309199|ref|XP_003361534.1| PREDICTED: AP-1 complex subunit sigma-1A-like [Sus scrofa]	1.000	0.824	0.539	0.476	0.893
gi|47523628|ref|NP_999442.1| aminopeptidase N [Sus scrofa] >gi|1703286|sp|P15145.3|AMPN_PIG RecName: Full=Aminopeptidase N; Short=AP-N; Short=pAPN; AltName: Full=Alanyl aminopeptidase; AltName: Full=Aminopeptidase M; Short=AP-M; AltName: Full=Microsomal aminopeptidase; AltName:	1.000	0.377	0.514	0.611	0.898
gi|350593858|ref|XP_003133682.3| PREDICTED: carbamoyl-phosphate synthase [ammonia], mitochondrial isoform 1 [Sus scrofa]	1.000	1.256	0.640	0.507	0.899

gi|346986447|ref|NP_001231373.1| ribonuclease H2 subunit A [Sus scrofa]	1.000	0.934	1.495	0.612	0.901
gi|311244862|ref|XP_001925828.2| PREDICTED: huntingtin-interacting protein K-like [Sus scrofa]	1.000	0.776	1.342	1.280	0.902
gi|335293615|ref|XP_003357006.1| PREDICTED: UDP-glucuronosyltransferase 2A1 [Sus scrofa]	1.000	0.731	0.513	0.642	0.911
gi|350581370|ref|XP_003124307.3| PREDICTED: MICAL-like protein 2 [Sus scrofa]	1.000	0.502	1.128	1.227	0.912
gi|350591926|ref|XP_003358845.2| PREDICTED: golgin subfamily B member 1 [Sus scrofa]	1.000	0.766	0.971	0.959	0.915
gi|334305778|sp|D2SW95.1|COPB_PIG RecName: Full=Coatomer subunit beta; AltName: Full=Beta-coat protein; Short=Beta-COP >gi|288860138|ref|NP_001165837.1| coatomer subunit beta [Sus scrofa] >gi|268633765|gb|ACZ15983.1| coatomer protein subunit beta 1 [Sus scrofa]	1.000	1.192	0.661	0.623	0.916
gi|169139278|gb|ACA48587.1| liver fatty acid binding protein [Sus scrofa]	1.000	0.903	1.628	1.294	0.917
gi|343790893|ref|NP_001230504.1| creatine kinase, brain [Sus scrofa]	1.000	1.616	0.641	0.774	0.917
gi|311258794|ref|XP_003127785.1| PREDICTED: sphingomyelin phosphodiesterase, acid-like 3B [Sus scrofa]	1.000	0.849	1.649	1.632	0.918
gi|350579853|ref|XP_003480698.1| PREDICTED: liprin-alpha-1-like [Sus scrofa]	1.000	1.347	1.462	1.395	0.919
gi|350580953|ref|XP_003354274.2| PREDICTED: 60S acidic ribosomal protein P1-like [Sus scrofa]	1.000	0.421	1.608	1.595	0.920
gi|47523794|ref|NP_999534.1| voltage-dependent anion-selective channel protein 2 [Sus scrofa] >gi|75050405|sp|Q9MZ15.1|VDAC2_PIG RecName: Full=Voltage-dependent anion-selective channel protein 2; Short=VDAC-2 >gi|8745554|gb|AAF78964.1|AF268462_1 voltage-dependent anion channel	1.000	0.872	0.478	0.433	0.922
gi|350582129|ref|XP_003354780.2| PREDICTED: ig kappa chain V-II region RPMI 6410-like [Sus scrofa]	1.000	0.919	1.388	1.364	0.923

gi|343791015|ref|NP_001230554.1| carboxylesterase 3 precursor [Sus scrofa]	1.000	0.942	0.662	0.829	0.924
gi|297747346|ref|NP_001177097.1| S100 calcium binding protein A14 [Sus scrofa]	1.000	0.868	1.211	1.289	0.925
gi|190360663|ref|NP_001121949.1| FYVE and coiled-coil domain-containing protein 1 [Sus scrofa] >gi|41688293|dbj|BAD08647.1| FYVE and coiled-coil domain containing 1 [Sus scrofa]	1.000	0.991	0.625	1.214	0.925
gi|1262435|emb|CAA61864.1| put. 26S protease subunit [Sus scrofa]	1.000	0.765	0.820	0.599	0.929
gi|1332717|gb|AAB36056.1| acylamino acid-releasing enzyme, AARE {EC 3.4.19.1} [swine, liver, Peptide, 732 aa] >gi|1583737|prf||2121361A acyl-AA releasing enzyme	1.000	1.103	0.791	0.977	0.930
gi|335286637|ref|XP_001927783.3| PREDICTED: nitrilase homolog 1 isoform 2 [Sus scrofa]	1.000	1.142	1.595	1.118	0.932
gi|325652134|ref|NP_001191701.1| bromodomain-containing protein 4 [Sus scrofa] >gi|321172828|gb|ADW77216.1| bromodomain-containing protein 4 [Sus scrofa]	1.000	0.575	1.145	0.883	0.932
gi|218664491|ref|NP_001136314.1| galectin-3 [Sus scrofa] >gi|215254098|gb|ACJ64061.1| lectin galactoside-binding soluble 2 protein [Sus scrofa]	1.000	1.047	1.249	0.849	0.939
gi|2833354|sp|Q29223.2|RL34_PIG RecName: Full=60S ribosomal protein L34	1.000	0.879	0.523	0.566	0.941
gi|350591034|ref|XP_003483187.1| PREDICTED: glycerol-3-phosphate dehydrogenase 1-like protein-like [Sus scrofa]	1.000	1.304	0.697	0.704	0.941
gi|350587641|ref|XP_003129114.3| PREDICTED: UDP-glucuronosyltransferase 2C1-like [Sus scrofa]	1.000	0.934	0.430	0.670	0.942
gi|335287882|ref|XP_003126232.2| PREDICTED: LOW QUALITY PROTEIN: sterol O-acyltransferase 2-like [Sus scrofa]	1.000	1.336	0.918	1.402	0.942
gi|346227222|ref|NP_001230997.1| ribosomal protein L13a isoform 1 [Sus scrofa]	1.000	0.802	0.636	0.500	0.943
gi|346421386|ref|NP_001231066.1| ATP synthase subunit g, mitochondrial [Sus scrofa]	1.000	0.905	0.362	0.382	0.946
gi|113205668|ref|NP_001038021.1| microsomal	1.000	1.689	0.870	0.986	0.950

glutathione S-transferase 3 [Sus scrofa] >gi|120564453|gb|ABM30152.1| microsomal glutathione S-transferase 3 [Sus scrofa] >gi|87047644|gb|ABD18455.1| microsomal glutathione S-transferase 3 [Sus scrofa]					
gi|311260243|ref|XP_001927987.2| PREDICTED: protein CutA isoform 1 [Sus scrofa]	1.000	0.621	2.449	1.975	0.951
gi|350597193|ref|XP_003484383.1| PREDICTED: LOW QUALITY PROTEIN: peptidyl-prolyl cis-trans isomerase B [Sus scrofa]	1.000	0.394	1.723	0.922	0.958
gi|311255888|ref|XP_003126410.1| PREDICTED: cullin-associated NEDD8-dissociated protein 1-like [Sus scrofa]	1.000	1.430	0.839	0.627	0.959
gi|311250237|ref|XP_003124024.1| PREDICTED: stress-70 protein, mitochondrial [Sus scrofa]	1.000	0.877	0.886	0.721	0.961
gi|45269029|gb|AAS55927.1| cytoskeletal beta actin, partial [Sus scrofa]	1.000	1.089	0.585	0.609	0.964
gi|350587231|ref|XP_003482373.1| PREDICTED: LOW QUALITY PROTEIN: cytoplasmic dynein 1 heavy chain 1 [Sus scrofa]	1.000	1.192	0.769	0.690	0.967
gi|335281560|ref|XP_003353830.1| PREDICTED: splicing factor 1 isoform 2 [Sus scrofa]	1.000	1.140	1.611	1.992	0.968
gi|343098455|ref|NP_001230202.1| ras-related protein Rab-6A [Sus scrofa]	1.000	1.156	0.719	0.616	0.970
gi|350582040|ref|XP_003481180.1| PREDICTED: U5 small nuclear ribonucleoprotein 200 kDa helicase-like [Sus scrofa]	1.000	1.299	0.968	0.982	0.974
gi|312283580|ref|NP_999292.2| superoxide dismutase [Mn], mitochondrial [Sus scrofa]	1.000	0.970	1.800	0.677	0.974
gi|335293826|ref|XP_003357065.1| PREDICTED: LOW QUALITY PROTEIN: short coiled-coil protein-like [Sus scrofa]	1.000	0.372	3.119	1.888	0.975
gi|385648282|ref|NP_001245314.1| glucosidase, beta (bile acid) 2 [Sus scrofa]	1.000	0.636	0.760	0.737	0.977
gi|124857|sp|P00998.1|ISK1_PIG RecName: Full=Pancreatic secretory trypsin inhibitor; AltName: Full=Serine protease inhibitor	1.000	0.546	1.059	0.297	0.980

Kazal-type 1 >gi|230351|pdb|1TGS|I Chain I, Three-Dimensional Structure Of The Complex Between Pancreatic Secretory Inhibitor (Kazal Type) And Trypsinogen					
gi|350592528|ref|XP_003483480.1| PREDICTED: endoplasmic reticulum resident protein 29 [Sus scrofa]	1.000	0.787	1.304	1.172	0.982
gi|350579445|ref|XP_003480606.1| PREDICTED: protein NipSnap homolog 3A-like [Sus scrofa]	1.000	0.842	1.427	1.801	0.984
gi|347543818|ref|NP_001231565.1| cytochrome P450, family 4, subfamily F, polypeptide 55 precursor [Sus scrofa]	1.000	0.959	0.472	0.472	0.986
gi|113205892|ref|NP_001038077.1| tubulin beta chain [Sus scrofa] >gi|75045190|sp|Q767L7.1|TBB5_PIG RecName: Full=Tubulin beta chain; AltName: Full=Tubulin beta-5 chain >gi|81174748|gb|ABB58916.1| beta 5-tubulin [Sus scrofa] >gi|41529175|dbj|BAD08435.1| beta 5-tubulin [Sus scrofa]	1.000	0.788	0.442	0.456	0.987
gi|45268981|gb|AAS55903.1| 60S ribosomal protein L12, partial [Sus scrofa]	1.000	0.878	0.609	0.594	0.987
gi|164543|gb|AAA31072.1| malate dehydrogenase (EC 1.1.1.37), partial [Sus scrofa]	1.000	0.973	0.715	0.601	0.988
gi|239504558|ref|NP_001155112.1| caspase-10 [Sus scrofa] >gi|229365449|dbj|BAH57972.1| caspase 10 [Sus scrofa]	1.000	1.116	0.668	0.738	0.988
gi|462384|sp|P33198.1|IDHP_PIG RecName: Full=Isocitrate dehydrogenase [NADP], mitochondrial; Short=IDH; AltName: Full=ICD-M; AltName: Full=IDP; AltName: Full=NADP(+)-specific ICDH; AltName: Full=Oxalosuccinate decarboxylase; Flags: Precursor >gi|294223|gb|AAA31089.1| NADPH-specif	1.000	1.284	0.588	0.640	0.990
gi|157427728|ref|NP_001098772.1| ATP-citrate synthase isoform 2 [Sus scrofa] >gi|380509279|gb|AFD64642.1| ATP citrate lyase short isoform [Sus scrofa] >gi|156140098|gb|ABU51323.1| ATP citrate lyase [Sus scrofa]	1.000	0.960	0.574	0.853	0.991

gi|346716326|ref|NP_001231181.1| SWI/SNF-related matrix-associated actin-dependent regulator of chromatin subfamily D member 2 [Sus scrofa]	1.000	0.627	0.547	0.905	0.991
gi|47522800|ref|NP_999152.1| 60S ribosomal protein L22 [Sus scrofa] >gi|54039123|sp|P67985.2|RL22_PIG RecName: Full=60S ribosomal protein L22; AltName: Full=Heparin-binding protein HBp15 >gi|409074|dbj|BAA04547.1| heparin binding protein [Sus scrofa]	1.000	0.815	0.549	0.479	0.997
gi|343478210|ref|NP_001230371.1| syntaxin 12 [Sus scrofa]	1.000	0.949	1.837	2.884	1.001
gi|311249251|ref|XP_003123542.1| PREDICTED: 6-phosphogluconolactonase-like [Sus scrofa]	1.000	1.405	0.796	0.756	1.009
gi|350587377|ref|XP_003356918.2| PREDICTED: cytosol aminopeptidase-like [Sus scrofa]	1.000	1.687	2.747	3.579	1.011
gi|350586674|ref|XP_001929500.4| PREDICTED: exportin-5 [Sus scrofa]	1.000	0.936	0.699	0.653	1.018
gi|351738777|gb|AEQ61487.1| ribokinase [Sus scrofa]	1.000	1.083	1.493	1.603	1.019
gi|350580256|ref|XP_003480773.1| PREDICTED: nucleobindin-2 [Sus scrofa]	1.000	0.608	1.207	1.564	1.020
gi|350593062|ref|XP_003133228.3| PREDICTED: xaa-Pro aminopeptidase 1 isoform 2 [Sus scrofa]	1.000	1.117	0.832	0.607	1.023
gi|335298834|ref|XP_003358406.1| PREDICTED: leucine-rich repeat flightless-interacting protein 2 [Sus scrofa]	1.000	0.894	1.546	1.044	1.027
gi|47522844|ref|NP_999174.1| complement C3 precursor [Sus scrofa] >gi|11869931|gb|AAG40565.1|AF154933_1 complement component C3 [Sus scrofa] >gi|27801787|emb|CAD38823.2| complement C3 precursor [Sus scrofa]	1.000	0.640	0.917	0.806	1.028
gi|342672022|ref|NP_001230144.1| exportin-2 [Sus scrofa]	1.000	1.359	0.529	0.497	1.030
gi|335284313|ref|XP_003124517.2| PREDICTED: RNA-binding protein FUS-like isoform 1 [Sus scrofa]	1.000	0.713	0.899	0.633	1.032

gi|350583346|ref|XP_003125776.3| PREDICTED: ubiquitin associated protein 2-like isoform 3 [Sus scrofa]	1.000	0.946	1.471	1.696	1.033
gi|335307509|ref|XP_003360867.1| PREDICTED: lipoma-preferred partner-like, partial [Sus scrofa]	1.000	0.795	1.317	1.276	1.036
gi|335297068|ref|XP_001927259.3| PREDICTED: CLYBL protein [Sus scrofa]	1.000	0.888	1.271	1.636	1.036
gi|311276332|ref|XP_003135175.1| PREDICTED: 3-hydroxyacyl-CoA dehydrogenase type-2-like isoform 2 [Sus scrofa]	1.000	1.185	0.750	0.745	1.037
gi|311246780|ref|XP_003122339.1| PREDICTED: haloacid dehalogenase-like hydrolase domain-containing protein 2-like [Sus scrofa]	1.000	0.868	1.466	1.718	1.043
gi|350591495|ref|XP_003132454.3| PREDICTED: coatomer subunit gamma [Sus scrofa]	1.000	1.377	0.929	0.721	1.045
gi|311258000|ref|XP_003127386.1| PREDICTED: AP-2 complex subunit alpha-1 isoform 1 [Sus scrofa]	1.000	1.207	0.648	0.815	1.052
gi|311259466|ref|XP_003128111.1| PREDICTED: putative hydroxypyruvate isomerase-like isoform 1 [Sus scrofa]	1.000	1.019	2.107	1.793	1.053
gi|350582315|ref|XP_003481243.1| PREDICTED: NFU1 iron-sulfur cluster scaffold homolog, mitochondrial-like [Sus scrofa]	1.000	0.809	1.818	1.689	1.055
gi|212549623|ref|NP_001131101.1| endoplasmic reticulum resident protein 44 precursor [Sus scrofa] >gi|187480160|gb|ACD13000.1| thioredoxin domain-containing 4 [Sus scrofa]	1.000	1.194	0.716	0.968	1.059
gi|350586216|ref|XP_003128039.3| PREDICTED: thioredoxin domain-containing protein 12-like [Sus scrofa]	1.000	0.456	1.098	1.272	1.059
gi|350584903|ref|XP_003126945.3| PREDICTED: AP-1 complex subunit gamma-1 isoform 1 [Sus scrofa]	1.000	1.115	0.685	0.787	1.059
gi|346421347|ref|NP_001231022.1| glucosamine-6-phosphate isomerase 1 [Sus scrofa]	1.000	0.958	1.649	1.438	1.064
gi|113205878|ref|NP_001038072.1| delta(3,5)-Delta(2,4)-dienoyl-CoA isomerase,	1.000	1.299	1.408	0.971	1.065

mitochondrial [Sus scrofa] >gi|90024980|gb|AAZ82038.2| peroxisomal enoyl coenzyme A hydratase 1 [Sus scrofa]					
gi|311275226|ref|XP_003134622.1| PREDICTED: glutathione S-transferase kappa 1-like isoform 2 [Sus scrofa]	1.000	0.864	0.619	0.577	1.072
gi|57528035|ref|NP_001009582.1| stathmin [Sus scrofa] >gi|75043336|sp|Q6DUB7.3|STMN1_PIG RecName: Full=Stathmin >gi|49615355|gb|AAT66936.1| stathmin-1 [Sus scrofa]	1.000	0.699	1.231	1.255	1.081
gi|343488538|ref|NP_001230424.1| cytoplasmic dynein 1 intermediate chain 2 [Sus scrofa]	1.000	0.686	1.581	1.663	1.087
gi|346716298|ref|NP_001231025.1| heterogeneous nuclear ribonucleoprotein G [Sus scrofa]	1.000	0.958	1.657	1.159	1.090
gi|298104082|ref|NP_001177109.1| dynein light chain roadblock-type 1 [Sus scrofa]	1.000	0.847	2.213	3.050	1.090
gi|335310032|ref|XP_003126353.2| PREDICTED: dynactin subunit 2-like isoform 3 [Sus scrofa]	1.000	1.748	1.035	0.915	1.093
gi|311246990|ref|XP_003122442.1| PREDICTED: mucin-2, partial [Sus scrofa]	1.000	1.835	0.865	0.677	1.098
gi|335280113|ref|XP_003121789.2| PREDICTED: LOW QUALITY PROTEIN: 60S ribosomal protein L4 [Sus scrofa]	1.000	0.772	0.477	0.419	1.100
gi|350595475|ref|XP_003360255.2| PREDICTED: proteasome subunit alpha type-2-like [Sus scrofa]	1.000	0.713	2.004	2.133	1.102
gi|350586921|ref|XP_003482304.1| PREDICTED: LOW QUALITY PROTEIN: apoptotic chromatin condensation inducer in the nucleus-like [Sus scrofa]	1.000	0.842	1.424	0.894	1.102
gi|311261483|ref|XP_003128742.1| PREDICTED: LOW QUALITY PROTEIN: golgin subfamily A member 5-like [Sus scrofa]	1.000	1.092	1.630	1.604	1.104
gi|350594033|ref|XP_003483821.1| PREDICTED: UDP-glucuronosyltransferase 1-10 isoform 2 [Sus scrofa]	1.000	0.806	0.583	0.609	1.105
gi|347300356|ref|NP_001231473.1| uncharacterized protein LOC100689297 [Sus	1.000	1.224	1.686	1.919	1.106

scrofa]					
gi|350581286|ref|XP_003124146.3| PREDICTED: hypothetical protein LOC100516425 [Sus scrofa]	1.000	1.102	1.883	1.285	1.111
gi|297591969|ref|NP_001172062.1| ubiquitin-conjugating enzyme E2 L3 [Sus scrofa] >gi|219563058|gb|ACL27888.1| ubiquitin-conjugating enzyme E2L 3 [Sus scrofa]	1.000	0.729	1.377	1.106	1.111
gi|75051712|sp|Q9TUB5.1|CLCA1_PIG RecName: Full=Calcium-activated chloride channel regulator 1; AltName: Full=Calcium-activated chloride channel family member 1; AltName: Full=pCLCA1; Flags: Precursor >gi|47523388|ref|NP_999313.1| calcium-activated chloride channel regulator 1 precu	1.000	1.042	1.503	1.076	1.117
gi|194038540|ref|XP_001929175.1| PREDICTED: ectonucleoside triphosphate diphosphohydrolase 5 [Sus scrofa]	1.000	0.769	0.948	0.509	1.118
gi|346986324|ref|NP_001231316.1| tumor protein D52 [Sus scrofa]	1.000	0.720	0.882	1.075	1.124
gi|329663924|ref|NP_001192332.1| tubulin-folding cofactor B-like [Sus scrofa] >gi|24528346|emb|CAD56044.1| cytoskeleton-associated protein 1 [Sus scrofa]	1.000	1.001	1.310	1.194	1.124
gi|335310868|ref|XP_003362230.1| PREDICTED: T-complex protein 1 subunit epsilon, partial [Sus scrofa]	1.000	1.491	0.598	0.472	1.125
gi|122064583|sp|P04178.2|SODC_PIG RecName: Full=Superoxide dismutase [Cu-Zn] >gi|298677090|ref|NP_001177351.1| superoxide dismutase [Cu-Zn] [Sus scrofa] >gi|297593564|gb|ADI47520.1| superoxide dismutase 1 [Sus scrofa]	1.000	0.935	1.540	1.450	1.125
gi|325652138|ref|NP_001191702.1| A-kinase anchor protein 1, mitochondrial [Sus scrofa] >gi|321267428|dbj|BAJ72691.1| A kinase anchor protein 1 [Sus scrofa]	1.000	0.686	1.180	1.032	1.126
gi|350585075|ref|XP_003481870.1| PREDICTED: xaa-Pro dipeptidase-like [Sus scrofa]	1.000	1.176	1.013	1.704	1.131
gi|335300836|ref|XP_003359054.1| PREDICTED: ES1 protein homolog,	1.000	0.485	1.208	0.769	1.133

mitochondrial-like [Sus scrofa]					
gi|350592730|ref|XP_003483523.1| PREDICTED: heterogeneous nuclear ribonucleoprotein F [Sus scrofa] >gi|335301642|ref|XP_003359253.1| PREDICTED: heterogeneous nuclear ribonucleoprotein F [Sus scrofa] >gi|194042674|ref|XP_001929089.1| PREDICTED: heterogeneous nuclear ribonucleoprotei	1.000	1.215	1.290	1.078	1.133
gi|350587966|ref|XP_003482525.1| PREDICTED: PDZ and LIM domain protein 5 isoform 3 [Sus scrofa]	1.000	0.779	1.597	1.479	1.140
gi|194037005|ref|XP_001928988.1| PREDICTED: ribonuclease UK114-like isoform 1 [Sus scrofa]	1.000	1.117	2.581	2.299	1.141
gi|350581741|ref|XP_003124660.3| PREDICTED: phosphomannomutase 2-like, partial [Sus scrofa]	1.000	1.863	1.052	1.590	1.141
gi|350586779|ref|XP_003482273.1| PREDICTED: LOW QUALITY PROTEIN: IQ motif containing GTPase activating protein 1 [Sus scrofa]	1.000	1.126	0.971	0.793	1.146
gi|1900|emb|CAA27575.1| unnamed protein product [Sus scrofa]	1.000	1.005	0.503	0.769	1.152
gi|350582471|ref|XP_003125244.3| PREDICTED: echinoderm microtubule associated protein like 4 [Sus scrofa]	1.000	0.952	1.303	1.407	1.152
gi|83921635|ref|NP_001033089.1| FKBP1A-like [Sus scrofa] >gi|61098747|gb|AAX37547.1| FKBP1A-like [Sus scrofa]	1.000	1.011	1.849	1.720	1.154
gi|350584176|ref|XP_003481686.1| PREDICTED: myosin-Ia-like [Sus scrofa]	1.000	0.968	0.862	0.644	1.164
gi|311259195|ref|XP_003127983.1| PREDICTED: plasminogen activator inhibitor 1 RNA-binding protein-like isoform 2 [Sus scrofa]	1.000	1.114	1.411	1.334	1.164
gi|350583407|ref|XP_003481511.1| PREDICTED: cathepsin S [Sus scrofa]	1.000	0.807	3.109	2.841	1.171
gi|322510017|sp|Q007T2.2|CDC42_PIG RecName: Full=Cell division control protein 42 homolog; Flags: Precursor >gi|269994011|dbj|BAI50642.1| Cell division control protein 42 homolog [Sus scrofa]	1.000	1.125	0.764	0.921	1.172

gi|335291962|ref|XP_001928015.3| PREDICTED: E3 ubiquitin-protein ligase TRIM31 [Sus scrofa] >gi|211926941|dbj|BAG82682.1| tripartite motif-containing protein 31 [Sus scrofa]	1.000	0.990	0.644	1.319	1.179
gi|350590070|ref|XP_003131163.3| PREDICTED: paraspeckle component 1 [Sus scrofa]	1.000	1.256	0.892	0.844	1.179
gi|225450|prf||1303334A valosin precursor	1.000	1.408	0.778	0.889	1.181
gi|350590733|ref|XP_003131874.3| PREDICTED: pre-mRNA-processing-splicing factor 8, partial [Sus scrofa]	1.000	1.245	0.745	0.327	1.182
gi|343790906|ref|NP_001230510.1| calcineurin-like phosphoesterase domain containing 1 [Sus scrofa]	1.000	0.755	2.436	1.559	1.194
gi|178056532|ref|NP_001116688.1| Z-DNA-binding protein 1 [Sus scrofa] >gi|147223427|emb|CAN13344.1| Z-DNA binding protein 1 [Sus scrofa]	1.000	0.703	0.731	1.318	1.195
gi|350587405|ref|XP_003482406.1| PREDICTED: cytosolic beta-glucosidase-like, partial [Sus scrofa]	1.000	1.402	0.973	0.831	1.198
gi|335297344|ref|XP_003131243.2| PREDICTED: integrin beta-4-like [Sus scrofa]	1.000	0.730	1.151	1.519	1.199
gi|194038734|ref|XP_001929226.1| PREDICTED: protein transport protein Sec23A isoform 2 [Sus scrofa]	1.000	1.655	0.875	0.634	1.202
gi|311272700|ref|XP_003133549.1| PREDICTED: heterogeneous nuclear ribonucleoprotein A3-like isoform 1 [Sus scrofa]	1.000	1.347	0.704	0.642	1.203
gi|75053043|sp|Q6UAQ8.3|ETFB_PIG RecName: Full=Electron transfer flavoprotein subunit beta; Short=Beta-ETF >gi|328550534|ref|NP_001192208.1| electron transfer flavoprotein subunit beta [Sus scrofa] >gi|35384838|gb|AAQ84565.1| electron transfer flavoprotein beta subunit precursor [S	1.000	1.237	0.867	1.028	1.205
gi|350582454|ref|XP_003125231.3| PREDICTED: LOW QUALITY PROTEIN: leucine-rich PPR motif-containing protein, mitochondrial [Sus scrofa]	1.000	1.183	0.968	0.808	1.206
gi|356582293|ref|NP_001239141.1| DEAH	1.000	0.875	0.801	0.869	1.207

(Asp-Glu-Ala-His) box polypeptide 15 [Sus scrofa]					
gi|47523822|ref|NP_999549.1| glutamate carboxypeptidase 2 [Sus scrofa] >gi|20138100|sp|O77564.1|FOLH1_PIG RecName: Full=Glutamate carboxypeptidase 2; AltName: Full=Folate hydrolase 1; AltName: Full=Folylpoly-gamma-glutamate carboxypeptidase; Short=FGCP; AltName: Full=Glutamate	1.000	1.112	2.071	2.577	1.210
gi|194044822|ref|XP_001927404.1| PREDICTED: peroxiredoxin-4 [Sus scrofa]	1.000	0.450	1.472	1.707	1.213
gi|311250752|ref|XP_003124284.1| PREDICTED: NACHT, LRR and PYD domains-containing protein 6 [Sus scrofa]	1.000	1.000	2.178	1.782	1.213
gi|148231223|ref|NP_001090898.1| mitochondrial antiviral-signaling protein [Sus scrofa] >gi|156144883|gb|ABU53000.1| virus-induced signaling adapter [Sus scrofa] >gi|119507309|dbj|BAF42542.1| mitochondrial antiviral signaling protein [Sus scrofa]	1.000	0.771	1.333	1.416	1.219
gi|311247536|ref|XP_003122689.1| PREDICTED: bifunctional ATP-dependent dihydroxyacetone kinase/FAD-AMP lyase (cyclizing)-like [Sus scrofa]	1.000	1.863	0.874	0.935	1.222
gi|194036835|ref|XP_001924895.1| PREDICTED: 4-trimethylaminobutyraldehyde dehydrogenase [Sus scrofa]	1.000	1.684	0.809	0.783	1.223
gi|311250151|ref|XP_003123979.1| PREDICTED: heat shock 70 kDa protein 4 [Sus scrofa]	1.000	1.453	2.216	1.688	1.224
gi|148234672|ref|NP_001090881.1| porphobilinogen deaminase [Sus scrofa] >gi|112980813|gb|ABI29188.1| hydroxymethylbilane synthase [Sus scrofa]	1.000	0.860	2.163	1.615	1.225
gi|213021241|ref|NP_001132944.1| proteasome subunit alpha type-6 [Sus scrofa] >gi|210062872|gb|ACJ06406.1| proteasome subunit alpha type 6 [Sus scrofa]	1.000	1.055	1.229	1.076	1.231
gi|335303414|ref|XP_001928370.3| PREDICTED: tubulin alpha-4A chain [Sus scrofa]	1.000	1.239	0.760	0.683	1.233

gi|335296459|ref|XP_003130769.2| PREDICTED: vimentin-like [Sus scrofa]	1.000	0.816	1.537	1.283	1.234
gi|194033860|ref|XP_001927901.1| PREDICTED: UV excision repair protein RAD23 homolog B [Sus scrofa]	1.000	1.642	2.407	2.937	1.236
gi|164669|gb|AAA31120.1| succinyl-CoA synthetase beta-subunit, partial [Sus scrofa]	1.000	1.758	0.974	0.824	1.244
gi|148232214|ref|NP_001090964.1| ribose-5-phosphate isomerase [Sus scrofa] >gi|158512830|sp|A2TLM1.1|RPIA_PIG RecName: Full=Ribose-5-phosphate isomerase; AltName: Full=Phosphoriboisomerase >gi|124265188|gb|ABM98101.1| ribose 5-phosphate isomerase-like protein [Sus scrofa]	1.000	0.938	3.291	3.682	1.253
gi|194036975|ref|XP_001927782.1| PREDICTED: polyadenylate-binding protein 1 [Sus scrofa]	1.000	0.878	1.445	1.228	1.255
gi|190360593|ref|NP_001121945.1| interferon-induced guanylate-binding protein 1 [Sus scrofa] >gi|166202346|gb|ABY84597.1| guanylate binding protein 1 [Sus scrofa]	1.000	0.933	0.844	0.731	1.258
gi|47523692|ref|NP_999478.1| thioredoxin [Sus scrofa] >gi|20141821|sp|P82460.3|THIO_PIG RecName: Full=Thioredoxin; Short=Trx >gi|332368598|gb|AEE61373.1| thioredoxin [Sus scrofa] >gi|14326453|gb|AAK60272.1|AF382821_1 thioredoxin [Sus scrofa]	1.000	1.007	2.615	2.229	1.258
gi|343488474|ref|NP_001230453.1| elongation factor 1-beta [Sus scrofa]	1.000	0.927	1.277	2.426	1.258
gi|225735709|ref|NP_001139599.1| clathrin heavy chain 1 [Sus scrofa] >gi|224492556|emb|CAR65329.1| clathrin heavy chain [Sus scrofa]	1.000	1.218	0.807	0.717	1.260
gi|350582501|ref|XP_003481284.1| PREDICTED: atlastin-2-like isoform 2 [Sus scrofa]	1.000	1.596	1.146	0.929	1.273
gi|350584145|ref|XP_001929467.3| PREDICTED: nascent polypeptide-associated complex subunit alpha isoform 1 [Sus scrofa]	1.000	0.879	1.852	1.608	1.283
gi|335305558|ref|XP_003134901.2| PREDICTED: heterogeneous nuclear	1.000	1.127	0.765	0.745	1.284

ribonucleoproteins A2/B1 isoform 1 [Sus scrofa]					
gi|48675927|ref|NP_001001632.1| tropomyosin alpha-3 chain [Sus scrofa] >gi|45272586|gb|AAS57724.1| tropomyosin 3 [Sus scrofa]	1.000	0.426	1.649	1.820	1.290
gi|148225750|ref|NP_001090973.1| heat shock protein 105 kDa [Sus scrofa] >gi|141521428|gb|ABO88027.1| heat shock 105kDa/110kDa protein 1 [Sus scrofa]	1.000	0.908	1.023	0.959	1.296
gi|343790920|ref|NP_001230517.1| fumarate hydratase [Sus scrofa]	1.000	1.509	0.966	0.890	1.303
gi|60394813|sp|Q29201.4|RS16_PIG RecName: Full=40S ribosomal protein S16 >gi|212549659|ref|NP_001131092.1| 40S ribosomal protein S16 [Sus scrofa] >gi|335289675|ref|XP_003355953.1| PREDICTED: 40S ribosomal protein S16-like isoform 2 [Sus scrofa] >gi|335289677|ref|XP_003355954.1| PRE	1.000	1.010	0.711	0.563	1.304
gi|68532157|gb|AAY98813.1| proteasome subunit 10 [Sus scrofa] >gi|68532159|gb|AAY98814.1| proteasome subunit 10 [Sus scrofa]	1.000	1.961	1.767	3.009	1.305
gi|311244872|ref|XP_003121596.1| PREDICTED: creatine kinase U-type, mitochondrial-like isoform 2 [Sus scrofa] >gi|311244870|ref|XP_003121595.1| PREDICTED: creatine kinase U-type, mitochondrial-like isoform 1 [Sus scrofa]	1.000	1.676	1.552	1.153	1.306
gi|311276184|ref|XP_003135086.1| PREDICTED: FUN14 domain-containing protein 1-like [Sus scrofa]	1.000	0.718	1.337	1.354	1.308
gi|346421435|ref|NP_001231091.1| sorbitol dehydrogenase [Sus scrofa]	1.000	1.724	1.163	0.884	1.309
gi|122131841|sp|Q06A98.1|SRSF2_PIG RecName: Full=Serine/arginine-rich splicing factor 2; AltName: Full=Splicing component, 35 kDa; AltName: Full=Splicing factor SC35; Short=SC-35; AltName: Full=Splicing factor, arginine/serine-rich 2 >gi|116175283|ref|NP_001070697.1| serine/arginine-	1.000	1.196	1.380	1.786	1.313
gi|389618965|gb|AFK92990.1| U2 small nuclear	1.000	1.303	1.058	1.085	1.315

RNA auxiliary factor 2 [Sus scrofa]					
gi|335282599|ref|XP_003123290.2| PREDICTED: interleukin enhancer-binding factor 3 [Sus scrofa]	1.000	1.011	1.226	1.104	1.319
gi|335281715|ref|XP_003122673.2| PREDICTED: L-asparaginase-like [Sus scrofa]	1.000	1.844	2.260	2.082	1.320
gi|312062797|ref|NP_001185848.1| proactivator polypeptide precursor [Sus scrofa] >gi|310789269|gb|ADP24688.1| prosaposin variant 2 [Sus scrofa]	1.000	0.731	2.233	2.259	1.324
gi|335302965|ref|XP_001925033.2| PREDICTED: lupus La protein homolog [Sus scrofa]	1.000	1.032	0.677	0.571	1.326
gi|346644874|ref|NP_001231109.1| T-complex protein 1 subunit gamma [Sus scrofa]	1.000	1.406	0.959	0.898	1.328
gi|343790856|ref|NP_001230567.1| glutathione transferase zeta 1 [Sus scrofa]	1.000	1.644	0.741	0.595	1.333
gi|268607671|ref|NP_001161267.1| actin, alpha skeletal muscle [Sus scrofa] >gi|62287931|sp|P68137.1|ACTS_PIG RecName: Full=Actin, alpha skeletal muscle; AltName: Full=Alpha-actin-1; Flags: Precursor >gi|790202|gb|AAC48692.1| skeletal alpha actin [Sus scrofa]	1.000	1.216	0.517	0.418	1.334
gi|350585579|ref|XP_003481996.1| PREDICTED: alpha-enolase-like, partial [Sus scrofa]	1.000	1.597	1.466	1.565	1.341
gi|350577999|ref|XP_001928755.4| PREDICTED: ezrin-like [Sus scrofa]	1.000	1.848	0.828	1.314	1.341
gi|47523870|ref|NP_999573.1| peroxiredoxin-6 [Sus scrofa] >gi|75074817|sp|Q9TSX9.3|PRDX6_PIG RecName: Full=Peroxiredoxin-6; AltName: Full=Non-selenium glutathione peroxidase; Short=NSGPx >gi|6689393|emb|CAB65456.1| non-selenium glutathione phospholipid hydroperoxide peroxidase	1.000	1.132	2.008	1.577	1.343
gi|350593430|ref|XP_003483683.1| PREDICTED: glutathione reductase, mitochondrial isoform 1 [Sus scrofa]	1.000	0.713	1.184	1.473	1.349
gi|335280672|ref|XP_003353634.1| PREDICTED: aldehyde dehydrogenase X, mitochondrial [Sus scrofa]	1.000	1.944	0.802	0.591	1.353

gi|55583761|sp|Q64L94.1|PSME1_PIG RecName: Full=Proteasome activator complex subunit 1; AltName: Full=Proteasome activator 28 subunit alpha; Short=PA28a; Short=PA28alpha >gi|34978646|gb|AAQ83574.1| proteasome activator 28 alpha subunit [Sus scrofa]	1.000	1.531	1.257	1.253	1.363
gi|335281676|ref|XP_003353867.1| PREDICTED: heterogeneous nuclear ribonucleoprotein U-like 2 [Sus scrofa]	1.000	1.432	0.889	1.030	1.367
gi|335299026|ref|XP_003132223.2| PREDICTED: microtubule-associated protein 4-like [Sus scrofa]	1.000	0.901	1.857	1.532	1.377
gi|555827|gb|AAA65943.1| immunoglobulin alpha heavy chain constant region, partial [Sus scrofa] >gi|2136551|pir||I47175 Ig alpha chain C region - pig (fragment)	1.000	0.720	0.817	1.044	1.378
gi|350587586|ref|XP_003129077.3| PREDICTED: hypothetical protein LOC100522154 [Sus scrofa]	1.000	1.027	1.932	2.369	1.379
gi|120968|sp|P80147.2|GABT_PIG RecName: Full=4-aminobutyrate aminotransferase, mitochondrial; AltName: Full=(S)-3-amino-2-methylpropionate transaminase; AltName: Full=GABA aminotransferase; Short=GABA-AT; AltName: Full=Gamma-amino-N-butyrate transaminase; Short=GABA transaminase;	1.000	1.695	0.783	0.717	1.390
gi|62511144|sp|Q764M5.1|STAT1_PIG RecName: Full=Signal transducer and activator of transcription 1 >gi|47523306|ref|NP_998934.1| signal transducer and activator of transcription 1 [Sus scrofa] >gi|40363629|dbj|BAD06318.1| signal transducer and activator of transcription 1 [Sus scrof	1.000	1.669	1.216	1.570	1.391
gi|335309657|ref|XP_003361720.1| PREDICTED: 14-3-3 protein epsilon-like [Sus scrofa]	1.000	1.488	0.899	0.780	1.393
gi|335289143|ref|XP_003126912.2| PREDICTED: probable D-lactate dehydrogenase, mitochondrial [Sus scrofa]	1.000	1.998	1.083	2.112	1.394
gi|346644844|ref|NP_001231177.1| phosphotriesterase-related protein [Sus scrofa]	1.000	1.138	2.160	2.467	1.396

gi|345441750|ref|NP_001230836.1| heat shock 70kDa protein 8 [Sus scrofa]	1.000	1.383	0.961	0.980	1.398
gi|311271975|ref|XP_001926332.2| PREDICTED: short/branched chain specific acyl-CoA dehydrogenase, mitochondrial [Sus scrofa]	1.000	1.307	0.714	0.940	1.408
gi|350580243|ref|XP_003123005.3| PREDICTED: harmonin-like [Sus scrofa]	1.000	0.886	2.100	1.767	1.414
gi|197251934|ref|NP_001127826.1| actin-related protein 2 [Sus scrofa] >gi|195562213|gb|ACG50178.1| actin-related protein 2-like protein [Sus scrofa]	1.000	1.397	0.500	0.574	1.417
gi|350591605|ref|XP_003483304.1| PREDICTED: plastin-1-like [Sus scrofa]	1.000	1.460	1.001	0.985	1.418
gi|346716348|ref|NP_001231189.1| arginine--tRNA ligase, cytoplasmic [Sus scrofa]	1.000	1.610	0.986	0.841	1.429
gi|47523086|ref|NP_999309.1| peroxiredoxin-5, mitochondrial [Sus scrofa] >gi|10305336|gb|AAG13452.2|AF110735_1 peroxiredoxin 5 [Sus scrofa]	1.000	1.866	0.912	0.881	1.432
gi|297591979|ref|NP_001172070.1| ornithine aminotransferase, mitochondrial [Sus scrofa] >gi|166244455|gb|ABY86572.1| mitochondrial ornithine aminotransferase [Sus scrofa]	1.000	1.701	1.067	0.856	1.434
gi|56788496|gb|AAW29969.1| unknown [Sus scrofa] >gi|56792880|gb|AAW30632.1| unknown [Sus scrofa]	1.000	0.647	1.212	0.768	1.437
gi|47117721|sp|P83884.2|RL36A_PIG RecName: Full=60S ribosomal protein L36a; AltName: Full=60S ribosomal protein L44 >gi|47522650|ref|NP_999082.1| 60S ribosomal protein L36a [Sus scrofa] >gi|335303998|ref|XP_003134062.2| PREDICTED: 60S ribosomal protein L36a-like [Sus scrofa] >gi|335	1.000	1.132	0.672	0.743	1.439
gi|1170740|sp|P00339.3|LDHA_PIG RecName: Full=L-lactate dehydrogenase A chain; Short=LDH-A; AltName: Full=LDH muscle subunit; Short=LDH-M >gi|473571|gb|AAA50436.1| lactate dehydrogenase-A [Sus scrofa domesticus]	1.000	1.799	0.825	0.705	1.440
gi|118403852|ref|NP_001072139.1| UBC1 [Sus	1.000	1.670	1.155	1.259	1.448

scrofa] >gi|115371727|gb|ABI96188.1| UBC1 [Sus scrofa]					
gi|311254260|ref|XP_001929592.2| PREDICTED: interleukin enhancer-binding factor 2 [Sus scrofa]	1.000	1.471	1.176	0.872	1.452
gi|155369760|ref|NP_001094498.1| peroxisomal acyl-coenzyme A oxidase 1 [Sus scrofa] >gi|112292673|gb|ABI14815.1| palmitoyl acyl-CoA oxidase 1 [Sus scrofa]	1.000	1.021	0.945	1.126	1.452
gi|194038542|ref|XP_001929208.1| PREDICTED: methylmalonate-semialdehyde dehydrogenase [acylating], mitochondrial [Sus scrofa]	1.000	1.593	0.928	0.929	1.458
gi|335296662|ref|XP_003130891.2| PREDICTED: 6-phosphofructokinase type C [Sus scrofa]	1.000	1.462	0.995	1.326	1.464
gi|194036227|ref|XP_001929678.1| PREDICTED: selenium-binding protein 1 [Sus scrofa]	1.000	1.132	1.756	2.005	1.465
gi|311248177|ref|XP_003123013.1| PREDICTED: proteasome subunit alpha type-1-like [Sus scrofa]	1.000	0.627	1.485	1.428	1.467
gi|350591320|ref|XP_003132344.3| PREDICTED: 26S proteasome non-ATPase regulatory subunit 6 [Sus scrofa]	1.000	1.844	1.187	0.979	1.468
gi|51592135|ref|NP_001004043.1| cofilin-1 [Sus scrofa] >gi|116850|sp|P10668.3|COF1_PIG RecName: Full=Cofilin-1; AltName: Full=Cofilin, non-muscle isoform >gi|164425|gb|AAA31020.1| cofilin [Sus scrofa]	1.000	1.814	1.452	0.952	1.469
gi|113205888|ref|NP_001038076.1| aldehyde dehydrogenase, mitochondrial precursor [Sus scrofa] >gi|122138098|sp|Q2XQV4.1|ALDH2_PIG RecName: Full=Aldehyde dehydrogenase, mitochondrial; AltName: Full=ALDH class 2; AltName: Full=ALDH-E2; Flags: Precursor >gi|81295909|gb|ABB70228.1| mit	1.000	1.732	0.778	0.734	1.475
gi|194034199|ref|XP_001928042.1| PREDICTED: proteasome subunit alpha type-3 isoform 2 [Sus scrofa]	1.000	1.080	2.055	1.906	1.477
gi|45268975|gb|AAS55900.1| 60S ribosomal protein L11, partial [Sus scrofa]	1.000	2.079	1.202	2.070	1.478

gi|350595271|ref|XP_003134762.3| PREDICTED: hypothetical protein LOC100521760 [Sus scrofa]	1.000	1.787	1.246	1.200	1.484
gi|12055551|emb|CAC21172.1| diazepam binding inhibitor [Sus scrofa]	1.000	1.667	2.489	2.516	1.486
gi|350587882|ref|XP_003129304.3| PREDICTED: caspase-6-like [Sus scrofa]	1.000	0.925	2.035	1.475	1.488
gi|311255145|ref|XP_001926338.2| PREDICTED: thiosulfate sulfurtransferase [Sus scrofa]	1.000	1.947	0.819	1.357	1.494
gi|89573899|gb|ABD77175.1| ribosomal protein L18 [Sus scrofa]	1.000	0.920	0.907	0.779	1.498
gi|47523670|ref|NP_999471.1| peroxisomal multifunctional enzyme type 2 [Sus scrofa] >gi|499340|emb|CAA55037.1| 17beta-estradiol dehydrogenase [Sus scrofa]	1.000	1.407	1.131	1.217	1.501
gi|194018698|ref|NP_001123442.1| cytochrome c [Sus scrofa] >gi|119388070|sp|P62895.2|CYC_PIG RecName: Full=Cytochrome c >gi|62208258|gb|AAX77008.1| cytochrome c-like protein [Sus scrofa]	1.000	1.081	2.968	2.164	1.501
gi|335300802|ref|XP_003359038.1| PREDICTED: 6-phosphofructokinase, liver type-like [Sus scrofa]	1.000	1.026	0.859	0.933	1.510
gi|283993079|gb|ADB57036.1| L-gulonate 3-dehydrogenase [Sus scrofa]	1.000	2.065	0.888	1.278	1.510
gi|47169449|pdb|1VJD|A Chain A, Structure Of Pig Muscle Pgk Complexed With Atp >gi|47169448|pdb|1VJC|A Chain A, Structure Of Pig Muscle Pgk Complexed With Mgatp	1.000	1.879	0.606	0.764	1.515
gi|350579323|ref|XP_003122006.3| PREDICTED: retinal dehydrogenase 1-like, partial [Sus scrofa]	1.000	1.350	1.241	1.953	1.515
gi|335293690|ref|XP_003129163.2| PREDICTED: general vesicular transport factor p115-like [Sus scrofa]	1.000	0.836	2.522	2.176	1.516
gi|346986445|ref|NP_001231372.1| NSFL1 (p97) cofactor (p47) [Sus scrofa]	1.000	1.188	1.880	1.872	1.520
gi|47523818|ref|NP_999546.1| calcium/calmodulin-dependent protein kinase	1.000	1.253	1.013	0.852	1.523

type II subunit delta [Sus scrofa] >gi|75073672|sp|Q95266.1|KCC2D_PIG RecName: Full=Calcium/calmodulin-dependent protein kinase type II subunit delta; Short=CaM kinase II subunit delta; Short=CaMK-II subun					
gi|350592964|ref|XP_003483582.1| PREDICTED: PDZ and LIM domain protein 1 [Sus scrofa]	1.000	1.389	2.467	2.281	1.529
gi|350590394|ref|XP_003131590.3| PREDICTED: pyridoxine-5~-phosphate oxidase-like [Sus scrofa]	1.000	1.599	2.451	2.121	1.531
gi|330417958|ref|NP_001155225.1| phosphoenolpyruvate carboxykinase [GTP], mitochondrial [Sus scrofa] >gi|238863892|gb|ACR66222.1| phosphoenolpyruvate carboxykinase 2 [Sus scrofa]	1.000	2.689	1.250	0.996	1.532
gi|350593418|ref|XP_003359535.2| PREDICTED: hypothetical protein LOC100627320 [Sus scrofa]	1.000	1.522	1.994	2.167	1.535
gi|116175259|ref|NP_001070686.1| heterogeneous nuclear ribonucleoprotein A1 [Sus scrofa] >gi|115371763|gb|ABI96206.1| ROA1 [Sus scrofa]	1.000	1.325	0.865	0.712	1.539
gi|356460899|ref|NP_999466.2| catalase [Sus scrofa]	1.000	0.830	1.677	1.644	1.541
gi|118403904|ref|NP_001072131.1| protein DJ-1 [Sus scrofa] >gi|67038668|gb|AAY63803.1| DJ-1 protein [Sus scrofa]	1.000	1.060	2.156	1.948	1.543
gi|255683394|ref|NP_001157474.1| ornithine carbamoyltransferase, mitochondrial [Sus scrofa]	1.000	0.910	1.058	1.682	1.552
gi|219522018|ref|NP_001137196.1| Na(+)/H(+) exchange regulatory cofactor NHE-RF1 [Sus scrofa] >gi|217031226|gb|ACJ74160.1| solute carrier family 9 (sodium/hydrogen exchanger) member 3 regulator 1 [Sus scrofa]	1.000	1.571	2.608	2.407	1.557
gi|346716265|ref|NP_001231271.1| omega-amidase NIT2 [Sus scrofa]	1.000	1.536	1.113	0.832	1.558
gi|194035831|ref|XP_001928742.1| PREDICTED: coatomer subunit alpha isoform 2 [Sus scrofa]	1.000	1.561	1.003	0.878	1.562
gi|343780946|ref|NP_001230486.1| D-dopachrome decarboxylase [Sus scrofa]	1.000	1.248	1.873	1.422	1.566

gi|312062805|ref|NP_001185852.1| chloride intracellular channel protein 5 [Sus scrofa] >gi|197361201|gb|ACH70136.1| chloride intracellular channel 5 [Sus scrofa]	1.000	2.034	0.871	1.000	1.572
gi|194037373|ref|XP_001927584.1| PREDICTED: coatomer subunit zeta-1 isoform 1 [Sus scrofa]	1.000	1.781	1.371	1.598	1.578
gi|194040624|ref|XP_001929173.1| PREDICTED: plastin-2 isoform 1 [Sus scrofa]	1.000	1.786	1.252	1.164	1.583
gi|350591042|ref|XP_003132145.3| PREDICTED: programmed cell death 6-interacting protein [Sus scrofa]	1.000	1.284	0.954	1.220	1.590
gi|335310692|ref|XP_003362151.1| PREDICTED: protein LZIC-like [Sus scrofa]	1.000	1.219	2.177	2.930	1.599
gi|335309939|ref|XP_003133770.2| PREDICTED: nucleolin-like [Sus scrofa]	1.000	1.089	1.648	1.607	1.599
gi|47523784|ref|NP_999528.1| 40S ribosomal protein S12 [Sus scrofa] >gi|1173191|sp|P46405.2|RS12_PIG RecName: Full=40S ribosomal protein S12 >gi|872315|emb|CAA55946.1| 40S ribosomal protein S12 [Sus scrofa]	1.000	0.974	1.068	0.878	1.607
gi|335282345|ref|XP_003354038.1| PREDICTED: mitochondrial import inner membrane translocase subunit Tim13-like [Sus scrofa]	1.000	1.780	2.187	2.239	1.616
gi|21954744|gb|AAM83105.1|AF525487_1 Ran [Sus scrofa]	1.000	1.757	0.822	0.618	1.618
gi|348605274|ref|NP_001231762.1| alcohol dehydrogenase 5 (class III), chi polypeptide [Sus scrofa]	1.000	1.887	0.872	0.792	1.618
gi|112985|sp|P00506.2|AATM_PIG RecName: Full=Aspartate aminotransferase, mitochondrial; Short=mAspAT; AltName: Full=Fatty acid-binding protein; Short=FABP-1; AltName: Full=Glutamate oxaloacetate transaminase 2; AltName: Full=Plasma membrane-associated fatty acid-binding protein;	1.000	1.009	1.728	2.191	1.620
gi|350592969|ref|XP_003483583.1| PREDICTED: phosphoglycerate mutase 1-like [Sus scrofa]	1.000	1.569	0.987	1.190	1.621
gi|335292272|ref|XP_003356696.1| PREDICTED: fumarylacetoacetase-like [Sus	1.000	1.473	3.380	2.688	1.629

scrofa]					
gi|122064241|sp|Q06AT0.3|HPCL1_PIG RecName: Full=Hippocalcin-like protein 1 >gi|178056468|ref|NP_001116598.1| hippocalcin-like protein 1 [Sus scrofa] >gi|115394792|gb|ABI97190.1| HPCL1 [Sus scrofa]	1.000	1.122	1.907	2.156	1.642
gi|350582722|ref|XP_003481338.1| PREDICTED: 14-3-3 protein theta isoform 1 [Sus scrofa]	1.000	1.696	1.369	1.297	1.659
gi|335294002|ref|XP_003357106.1| PREDICTED: 3-hydroxybutyrate dehydrogenase type 2-like isoform 2 [Sus scrofa] >gi|335294000|ref|XP_003129333.2| PREDICTED: 3-hydroxybutyrate dehydrogenase type 2-like isoform 1 [Sus scrofa]	1.000	2.360	0.622	0.791	1.663
gi|335297787|ref|XP_003131575.2| PREDICTED: puromycin-sensitive aminopeptidase isoform 1 [Sus scrofa]	1.000	1.467	0.977	0.697	1.664
gi|211578376|ref|NP_001129984.1| beta,beta-carotene 15,15~-monooxygenase [Sus scrofa] >gi|209571749|gb|ACI62530.1| beta-carotene 15,15~-monooxygenase 1 [Sus scrofa]	1.000	1.281	1.155	1.164	1.667
gi|239977642|sp|Q29183.1|TFF3_PIG RecName: Full=Trefoil factor 3; AltName: Full=Intestinal trefoil factor; Flags: Precursor	1.000	0.853	4.328	3.069	1.667
gi|47523516|ref|NP_999381.1| inositol monophosphatase 1 [Sus scrofa] >gi|68568737|sp|O77591.1|IMPA1_PIG RecName: Full=Inositol monophosphatase 1; Short=IMP 1; Short=IMPase 1; AltName: Full=Inositol-1(or 4)-monophosphatase 1; AltName: Full=Lithium-sensitive myo-inositol monophos	1.000	1.006	2.592	2.528	1.676
gi|297591965|ref|NP_001172061.1| leukotriene A-4 hydrolase [Sus scrofa] >gi|262204898|dbj|BAI48029.1| leukotriene A4 hydrolase [Sus scrofa]	1.000	1.816	0.956	0.872	1.682
gi|47522738|ref|NP_999119.1| aconitate hydratase, mitochondrial precursor [Sus scrofa] >gi|113159|sp|P16276.1|ACON_PIG RecName: Full=Aconitate hydratase,	1.000	1.770	1.054	1.058	1.686

mitochondrial; Short=Aconitase; AltName: Full=Citrate hydro-lyase; Flags: Precursor >gi|164315|gb|AAA30987.1| heart aconitas					
gi|345441801|ref|NP_001230874.1| TNF receptor-associated protein 1 [Sus scrofa]	1.000	1.932	0.894	0.943	1.689
gi|84028320|sp|Q9GKX6.1|GALM_PIG RecName: Full=Aldose 1-epimerase; AltName: Full=Galactose mutarotase >gi|47523866|ref|NP_999571.1| aldose 1-epimerase [Sus scrofa] >gi|11611545|dbj|BAB18973.1| aldose 1-epimerase [Sus scrofa]	1.000	1.501	0.864	0.931	1.695
gi|335305925|ref|XP_003135119.2| PREDICTED: LOW QUALITY PROTEIN: ubiquitin-like modifier activating enzyme 1 [Sus scrofa]	1.000	1.971	0.991	0.967	1.697
gi|1352666|sp|P11493.2|PP2AB_PIG RecName: Full=Serine/threonine-protein phosphatase 2A catalytic subunit beta isoform; Short=PP2A-beta >gi|164298|gb|AAA30982.1| protein phosphatase 2A beta subunit, partial [Sus scrofa]	1.000	2.065	1.224	0.936	1.719
gi|194044626|ref|XP_001927289.1| PREDICTED: 14-3-3 protein beta/alpha isoform 1 [Sus scrofa]	1.000	2.234	1.272	0.948	1.724
gi|311250943|ref|XP_003124353.1| PREDICTED: 28 kDa heat- and acid-stable phosphoprotein-like [Sus scrofa]	1.000	0.855	1.997	1.660	1.733
gi|347543782|ref|NP_001231554.1| glutathione synthetase [Sus scrofa]	1.000	1.749	1.676	2.554	1.735
gi|162951821|ref|NP_001106165.1| heterogeneous nuclear ribonucleoprotein A/B [Sus scrofa] >gi|160858224|dbj|BAF93845.1| CArG-binding factor A [Sus scrofa]	1.000	0.998	2.227	1.941	1.736
gi|311268173|ref|XP_003131919.1| PREDICTED: myb-binding protein 1A [Sus scrofa]	1.000	0.608	2.053	1.081	1.739
gi|71361861|gb|AAZ30034.1| Mx1 [Sus scrofa]	1.000	0.967	1.069	0.893	1.743
gi|47522916|ref|NP_999215.1| glutathione S-transferase omega-1 [Sus scrofa] >gi|20141290|sp|Q9N1F5.2|GSTO1_PIG RecName: Full=Glutathione S-transferase	1.000	1.410	0.791	0.625	1.755

<table>
<tr><td>omega-1; Short=GSTO-1; AltName: Full=Glutathione S-transferase omega 1-1; Short=GSTO 1-1; AltName: Full=Glutathione-dependent d</td><td></td><td></td><td></td><td></td><td></td></tr>
<tr><td>gi|335310331|ref|XP_003361983.1| PREDICTED: poly(U)-binding-splicing factor PUF60-like [Sus scrofa]</td><td>1.000</td><td>1.293</td><td>1.065</td><td>0.792</td><td>1.760</td></tr>
<tr><td>gi|52000924|sp|Q29238.2|CLIC1_PIG RecName: Full=Chloride intracellular channel protein 1; AltName: Full=Nuclear chloride ion channel 27</td><td>1.000</td><td>1.730</td><td>0.733</td><td>0.748</td><td>1.761</td></tr>
<tr><td>gi|197251946|ref|NP_001127815.1| actin-related protein 3 [Sus scrofa] >gi|195562215|gb|ACG50179.1| actin-related protein 3-like protein [Sus scrofa]</td><td>1.000</td><td>1.498</td><td>1.237</td><td>1.031</td><td>1.763</td></tr>
<tr><td>gi|48675953|ref|NP_001001643.1| rab GDP dissociation inhibitor beta [Sus scrofa] >gi|75043802|sp|Q6Q7J2.1|GDIB_PIG RecName: Full=Rab GDP dissociation inhibitor beta; Short=Rab GDI beta; AltName: Full=Guanosine diphosphate dissociation inhibitor 2; Short=GDI-2 >gi|45758488|gb|AAS76</td><td>1.000</td><td>2.866</td><td>1.102</td><td>0.916</td><td>1.781</td></tr>
<tr><td>gi|335281104|ref|XP_003353734.1| PREDICTED: argininosuccinate synthase [Sus scrofa]</td><td>1.000</td><td>1.671</td><td>2.308</td><td>2.587</td><td>1.797</td></tr>
<tr><td>gi|194041937|ref|XP_001928912.1| PREDICTED: alpha-centractin [Sus scrofa]</td><td>1.000</td><td>1.787</td><td>1.406</td><td>1.284</td><td>1.802</td></tr>
<tr><td>gi|319401915|ref|NP_001188313.1| rho GDP-dissociation inhibitor 1 [Sus scrofa] >gi|315321426|gb|ADU04840.1| Rho GDP dissociation inhibitor alpha [Sus scrofa]</td><td>1.000</td><td>2.584</td><td>0.973</td><td>1.127</td><td>1.810</td></tr>
<tr><td>gi|311277231|ref|XP_003135559.1| PREDICTED: trimethyllysine dioxygenase, mitochondrial-like [Sus scrofa]</td><td>1.000</td><td>1.143</td><td>5.553</td><td>3.922</td><td>1.817</td></tr>
<tr><td>gi|311252247|ref|XP_003124998.1| PREDICTED: macrophage-capping protein-like isoform 4 [Sus scrofa] >gi|311252243|ref|XP_003124996.1| PREDICTED: macrophage-capping protein-like isoform 2 [Sus scrofa] >gi|311252249|ref|XP_003124997.1| PREDICTED: macrophage-capping protein-like isofor</td><td>1.000</td><td>1.498</td><td>2.400</td><td>4.476</td><td>1.818</td></tr>
</table>

gi|311252615|ref|XP_003125184.1| PREDICTED: ubiquitin-40S ribosomal protein S27a-like [Sus scrofa] >gi|311252617|ref|XP_003125185.1| PREDICTED: ubiquitin-40S ribosomal protein S27a-like [Sus scrofa]	1.000	1.316	3.017	2.609	1.820
gi|350582355|ref|XP_003354851.2| PREDICTED: exportin-1 [Sus scrofa]	1.000	2.597	2.292	1.444	1.821
gi|223046|prf||0410468A aminotransferase,Asp	1.000	1.169	1.787	1.918	1.829
gi|999620|pdb|1MLD|D Chain D, Refined Structure Of Mitochondrial Malate Dehydrogenase From Porcine Heart And The Consensus Structure For Dicarboxylic Acid Oxidoreductases >gi|999618|pdb|1MLD|B Chain B, Refined Structure Of Mitochondrial Malate Dehydrogenase From Porcine	1.000	2.014	1.057	0.879	1.832
gi|311252403|ref|XP_003125080.1| PREDICTED: sepiapterin reductase-like [Sus scrofa]	1.000	1.394	1.350	1.971	1.837
gi|148237564|ref|NP_001090977.1| adenylosuccinate synthetase isozyme 2 [Sus scrofa] >gi|189031715|sp|A4Z6H1.1|PURA2_PIG RecName: Full=Adenylosuccinate synthetase isozyme 2; Short=AMPSase 2; Short=AdSS 2; AltName: Full=Adenylosuccinate synthetase, acidic isozyme; AltName: Full=Adeny	1.000	2.151	1.756	2.876	1.842
gi|311253255|ref|XP_003125488.1| PREDICTED: alanine aminotransferase 1-like [Sus scrofa]	1.000	1.108	1.782	2.460	1.848
gi|212549619|ref|NP_001131099.1| ERO1-like protein alpha precursor [Sus scrofa] >gi|226741411|sp|B6CVD7.1|ERO1A_PIG RecName: Full=ERO1-like protein alpha; Short=ERO1-L; Short=ERO1-L-alpha; AltName: Full=Endoplasmic oxidoreductin-1-like protein; AltName: Full=Oxidoreductin-1-L-alpha	1.000	1.314	1.946	1.577	1.857
gi|335300581|ref|XP_003358947.1| PREDICTED: T-complex protein 1 subunit theta-like isoform 1 [Sus scrofa]	1.000	1.545	0.974	0.794	1.863
gi|164414678|emb|CAO81735.1| Alternative Pig Liver Esterase [Sus scrofa]	1.000	0.927	1.243	1.579	1.871
gi|311270662|ref|XP_003132938.1| PREDICTED:	1.000	1.826	3.013	2.036	1.876

phosphatidylethanolamine-binding protein 1-like [Sus scrofa]					
gi|281427370|ref|NP_001163993.1| T-complex protein 1 subunit eta [Sus scrofa] >gi|262036937|dbj|BAI47603.1| chaperonin containing TCP1, subunit 7 (eta) [Sus scrofa]	1.000	1.904	0.901	0.911	1.878
gi|222136590|ref|NP_001138373.1| proteasome subunit alpha type-5 [Sus scrofa]	1.000	1.041	2.413	2.229	1.879
gi|311245810|ref|XP_001924714.2| PREDICTED: GTP:AMP phosphotransferase, mitochondrial isoform 1 [Sus scrofa]	1.000	1.660	0.785	1.152	1.897
gi|350583020|ref|XP_003481419.1| PREDICTED: zinc finger protein 706-like [Sus scrofa]	1.000	1.271	1.945	2.395	1.900
gi|335288894|ref|XP_003126772.2| PREDICTED: ubiquitin-conjugating enzyme E2 N-like [Sus scrofa]	1.000	1.780	2.226	1.835	1.902
gi|347300325|ref|NP_001231461.1| dehydrogenase/reductase SDR family member 11 [Sus scrofa]	1.000	2.520	1.354	1.392	1.907
gi|350578507|ref|XP_003121503.3| PREDICTED: asparaginyl-tRNA synthetase, cytoplasmic-like [Sus scrofa]	1.000	1.923	1.266	1.484	1.920
gi|47523456|ref|NP_999349.1| serine/threonine-protein phosphatase PP1-beta catalytic subunit [Sus scrofa] >gi|47116968|sp|P61292.3|PP1B_PIG RecName: Full=Serine/threonine-protein phosphatase PP1-beta catalytic subunit; Short=PP-1B >gi|89257972|gb|ABD65256.1| protein phosphatase	1.000	1.843	1.251	1.257	1.937
gi|195539476|ref|NP_001124208.1| bifunctional purine biosynthesis protein PURH [Sus scrofa] >gi|192383834|gb|ACF04802.1| 5-aminoimidazole-4-carboxamide ribonucleotide formyltransferase/IMP cyclohydrolase [Sus scrofa]	1.000	1.674	0.855	1.284	1.949
gi|350582612|ref|XP_003481312.1| PREDICTED: protein phosphatase 1G-like [Sus scrofa]	1.000	1.641	2.347	2.165	1.949
gi|335282386|ref|XP_003354050.1| PREDICTED: elongation factor 2 [Sus scrofa]	1.000	1.833	1.100	0.974	1.955

gi|335293373|ref|XP_003356947.1| PREDICTED: UDP-glucose 6-dehydrogenase-like isoform 1 [Sus scrofa]	1.000	1.734	1.612	1.136	1.981
gi|311273061|ref|XP_001925202.2| PREDICTED: villin 1 [Sus scrofa]	1.000	1.437	1.059	0.998	1.990
gi|122114359|gb|AAY42145.2| cathepsin D [Sus scrofa]	1.000	1.729	1.191	1.125	1.992
gi|345199288|ref|NP_001230831.1| protein phosphatase 1, regulatory (inhibitor) subunit 7 [Sus scrofa]	1.000	1.888	1.653	0.854	2.001
gi|346986388|ref|NP_001231345.1| profilin-1 [Sus scrofa]	1.000	2.107	1.375	1.305	2.004
gi|350596362|ref|XP_003361090.2| PREDICTED: CD2-associated protein-like [Sus scrofa]	1.000	1.357	5.830	3.915	2.031
gi|350586543|ref|XP_003482212.1| PREDICTED: glutamate--cysteine ligase catalytic subunit isoform 2 [Sus scrofa]	1.000	2.588	1.351	1.762	2.034
gi|350585077|ref|XP_003481871.1| PREDICTED: hypothetical protein LOC100739300 [Sus scrofa]	1.000	0.914	1.823	1.807	2.044
gi|335309827|ref|XP_003361787.1| PREDICTED: nuclear autoantigenic sperm protein, partial [Sus scrofa]	1.000	1.435	2.468	1.368	2.088
gi|47523618|ref|NP_999441.1| citrate synthase, mitochondrial precursor [Sus scrofa] >gi|116470|sp|P00889.2|CISY_PIG RecName: Full=Citrate synthase, mitochondrial; Flags: Precursor >gi|164419|gb|AAA31017.1| citrate synthase precursor (EC 4.1.3.7) [Sus scrofa]	1.000	2.016	0.966	1.408	2.124
gi|112976|sp|P00503.3|AATC_PIG RecName: Full=Aspartate aminotransferase, cytoplasmic; AltName: Full=Glutamate oxaloacetate transaminase 1; AltName: Full=Transaminase A >gi|47522636|ref|NP_999092.1| aspartate aminotransferase, cytoplasmic [Sus scrofa] >gi|300557343|emb|CBV09643.1|	1.000	1.113	2.697	3.518	2.144
gi|262072939|dbj|BAI47777.1| CCHC-type zinc finger, nucleic acid binding protein [Sus scrofa]	1.000	1.380	2.221	1.925	2.168
gi|35384836|gb|AAQ84564.1| electron transfer flavoprotein alpha subunit precursor [Sus scrofa]	1.000	2.734	1.242	1.160	2.173

gi|24987656|pdb|1LXE|A Chain A, Crystal Structure Of The Cathelicidin Motif Of Protegrins >gi|24987482|pdb|1KWI|A Chain A, Crystal Structure Analysis Of The Cathelicidin Motif Of Protegrins	1.000	0.329	1.160	0.826	2.176
gi|311267406|ref|XP_003131549.1| PREDICTED: gasdermin-B-like [Sus scrofa]	1.000	1.558	1.337	1.747	2.213
gi|346644743|ref|NP_001231138.1| calmodulin 3 [Sus scrofa] >gi|346644746|ref|NP_001231139.1| calmodulin 1 [Sus scrofa] >gi|311252670|ref|XP_003125211.1| PREDICTED: calmodulin-like [Sus scrofa]	1.000	1.687	2.281	4.172	2.230
gi|194040450|ref|XP_001927992.1| PREDICTED: lactoylglutathione lyase isoform 1 [Sus scrofa]	1.000	1.245	1.591	3.199	2.250
gi|281500757|pdb|3FX4|A Chain A, Porcine Aldehyde Reductase In Ternary Complex With Inhibitor >gi|229597975|pdb|3H4G|A Chain A, Structure Of Aldehyde Reductase Holoenzyme In Complex With Potent Aldose Reductase Inhibitor Fidarestat: Implications For Inhibitor Binding And S	1.000	3.159	1.610	1.180	2.261
gi|311261988|ref|XP_003128958.1| PREDICTED: phosphoglucomutase-2 [Sus scrofa]	1.000	1.677	1.345	1.583	2.313
gi|194043292|ref|XP_001928111.1| PREDICTED: 14-3-3 protein eta [Sus scrofa]	1.000	3.227	1.275	1.172	2.330
gi|112980811|gb|ABI29187.1| glyceraldehyde-3-phosphate dehydrogenase [Sus scrofa]	1.000	2.033	1.297	0.982	2.414
gi|346986428|ref|NP_001231362.1| heat shock 90kD protein 1, beta [Sus scrofa]	1.000	1.860	1.323	0.967	2.418
gi|47523764|ref|NP_999518.1| peptidyl-prolyl cis-trans isomerase A [Sus scrofa] >gi|51702768|sp|P62936.2|PPIA_PIG RecName: Full=Peptidyl-prolyl cis-trans isomerase A; Short=PPIase A; AltName: Full=Cyclophilin A; AltName: Full=Cyclosporin A-binding protein; AltName: Full=Rotamas	1.000	2.721	1.273	0.985	2.422
gi|349732201|ref|NP_001231854.1| dpy-30 homolog [Sus scrofa]	1.000	0.546	1.038	2.141	2.438
gi|1845|emb|CAA48565.1| aminoacylase I [Sus scrofa]	1.000	1.855	2.955	3.909	2.438

gi|346644699|ref|NP_001231019.1| protein SET [Sus scrofa]	1.000	1.404	4.301	2.967	2.469
gi|237681312|ref|NP_001153744.1| protein S100-A12 [Sus scrofa] >gi|126302600|sp|P80310.2|S10AC_PIG RecName: Full=Protein S100-A12; AltName: Full=Calgranulin-C; Short=CAGC; AltName: Full=Extracellular newly identified RAGE-binding protein; Short=EN-RAGE; AltName: Full=S100 calcium-b	1.000	0.787	1.875	1.494	2.476
gi|122220|sp|P17630.1|TCO1_PIG RecName: Full=Transcobalamin-1; Short=TC-1; AltName: Full=Cobalophilin; AltName: Full=Haptocorrin; AltName: Full=Protein R; AltName: Full=Transcobalamin I; Short=TC I; Short=TCI; Flags: Precursor >gi|1964|emb|CAA36800.1| unnamed protein product [Sus	1.000	1.622	1.801	1.275	2.573
gi|335284301|ref|XP_003354567.1| PREDICTED: T-complex protein 1 subunit zeta-like isoform 2 [Sus scrofa]	1.000	2.957	1.740	1.338	2.699
gi|190360639|ref|NP_001121941.1| interferon-induced 17 kDa protein [Sus scrofa] >gi|182406743|gb|ACB87600.1| interferon stimulated gene 15 [Sus scrofa]	1.000	0.780	2.300	4.581	2.706
gi|1364248|emb|CAA82246.1| glucosephosphate isomerase [Sus scrofa]	1.000	2.652	1.874	1.372	2.710
gi|350583022|ref|XP_001927263.3| PREDICTED: 14-3-3 protein zeta/delta [Sus scrofa]	1.000	3.308	1.517	1.086	2.726
gi|343962597|ref|NP_001230636.1| cytosolic non-specific dipeptidase [Sus scrofa]	1.000	1.600	3.758	3.289	2.765
gi|335281222|ref|XP_003353764.1| PREDICTED: probable 2-ketogluconate reductase-like isoform 2 [Sus scrofa]	1.000	2.964	2.138	1.420	2.895
gi|194043017|ref|XP_001928354.1| PREDICTED: MGC159817 protein isoform 1 [Sus scrofa]	1.000	2.079	1.832	2.726	2.978
gi|311259408|ref|XP_003128090.1| PREDICTED: peroxiredoxin-1 isoform 5 [Sus scrofa]	1.000	2.943	1.704	1.309	3.046
gi|90200404|gb|ABD92704.1| triosephosphate isomerase 1 [Sus scrofa]	1.000	2.706	1.493	1.114	3.048
gi|219521990|ref|NP_001137182.1| granzyme B	1.000	1.167	3.528	4.173	3.057

precursor [Sus scrofa] >gi|216408333|gb|ACJ72857.1| granzyme B [Sus scrofa]					
gi|7288152|dbj|BAA92850.1| esterase D [Sus scrofa]	1.000	3.122	1.562	2.008	3.194
gi|229620|prf||770227A dehydrogenase H4,lactate	1.000	2.789	2.491	1.866	3.254
gi|264681436|ref|NP_001161113.1| copper transport protein ATOX1 [Sus scrofa] >gi|262036932|dbj|BAI47601.1| antioxidant protein 1 homolog (yeast) [Sus scrofa]	1.000	1.280	2.727	3.209	3.307
gi|335300686|ref|XP_003358992.1| PREDICTED: carbonyl reductase [NADPH] 1-like [Sus scrofa]	1.000	3.339	1.314	1.332	3.405
gi|47523158|ref|NP_999015.1| glutathione S-transferase A2 [Sus scrofa] >gi|1185280|emb|CAA93434.1| glutathione S-transferase [Sus scrofa]	1.000	2.229	1.726	1.798	3.489
gi|162952052|ref|NP_001106151.1| transketolase [Sus scrofa] >gi|159502444|gb|ABW97521.1| transketolase [Sus scrofa]	1.000	1.819	2.627	3.235	3.514
gi|116047849|gb|ABJ53147.1| glutathione S-transferase mu 2 [Sus scrofa]	1.000	2.290	1.598	1.589	3.519
gi|350597032|ref|XP_001925921.4| PREDICTED: aldehyde dehydrogenase family 1 member A3, partial [Sus scrofa]	1.000	2.310	4.200	3.338	3.713
gi|311251248|ref|XP_003124516.1| PREDICTED: apoptosis-associated speck-like protein containing a CARD-like isoform 1 [Sus scrofa]	1.000	3.139	6.012	6.925	3.814
gi|266018|gb|AAB25484.1| calmodulin=low affinity vasoactive intestinal polypeptide binding protein [swine, liver, Peptide Partial, 34 aa]	1.000	1.397	2.473	2.726	4.203
